# Rapid Access to Azabicyclo[3.3.1]nonanes by a Tandem Diverted Tsuji–Trost Process

**DOI:** 10.1002/chem.202003762

**Published:** 2020-10-06

**Authors:** Hannah G. Steeds, Jonathan P. Knowles, Wai L. Yu, Jeffery Richardson, Katie G. Cooper, Kevin I. Booker‐Milburn

**Affiliations:** ^1^ School of Chemistry University of Bristol Cantock' Close Bristol BS8 1TS UK; ^2^ Northumbria University Newcastle upon Tyne NE1 8ST UK; ^3^ DCRT European Innovation Hub, Lilly Erl Wood Manor Windlesham GU20 6PH UK; ^4^ Pharmaceutical Technology & Development AstraZeneca Macclesfield Campus Cheshire SK10 2NA UK

**Keywords:** heterocycles, morphan, Pd catalysis, rearrangements, Tsuji–Trost

## Abstract

A three‐step synthesis of the 2‐azabicyclo[3.3.1]nonane ring system from simple pyrroles, employing a combined photochemical/palladium‐catalysed approach is reported. Substrate scope is broad, allowing the incorporation of a wide range of functionality relevant to medicinal chemistry. Mechanistic studies demonstrate that the process occurs by acid‐assisted C−N bond cleavage followed by β‐hydride elimination to form a reactive diene, demonstrating that efficient control of what might be considered off‐cycle reactions can result in productive tandem catalytic processes. This represents a short and versatile route to the biologically important morphan scaffold.

## Graphical abstract

**Easily diverted**: A rapid entry to the biologically important morphan ring system highlights the powerful combination of photochemistry and catalysis. The key palladium‐catalysed transformation utilises a novel diverted Tsuji–Trost sequence. Mechanistic studies demonstrate this process occurs by acid‐assisted C−N bond cleavage followed by β‐hydride elimination to form a reactive diene.

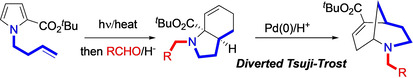

Since their discovery, palladium‐catalysed cross‐coupling reactions have seen increasing use in the synthesis of bioactive molecules.^[1]^ In particular, due to its reliability, the Suzuki cross‐coupling has become a key C−C bond forming reaction within medicinal chemistry.^[2]^ However, the resulting compounds are often relatively planar in nature, despite evidence that increased bioactivity might result from increased levels of sp^3^‐hybridized carbon.^[3]^ The Tsuji–Trost allylation represents a palladium‐catalysed process with potential to achieve more three‐dimensional molecules, necessarily connecting fragments via sp^3^‐hybridized centres.^[4]^ Recent work has added to this potential with increasingly effective systems for performing enantioselective Tsuji–Trost reactions.^[5]^ The power of such reactions within tandem processes has also been demonstrated, particularly in combination with photochemistry to create complex, three‐dimensional molecules from simple substrates (Scheme 1 a).^[6]^

**Scheme 1:**
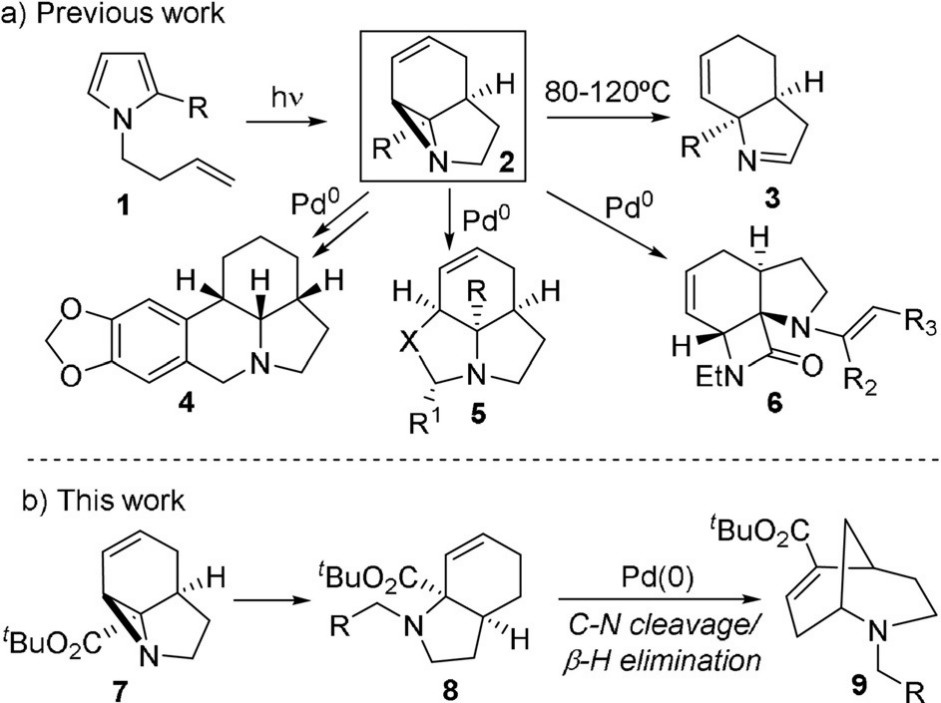
Previous synthetic utility of photochemically synthesized vinyl aziridines and their formation of azabicyclo[3.3.1]nonanes in a diverted Tsuji–Trost process.^[6]^

Tsuji–Trost reactions are also potentially less prone to side reactions, such as competing protodehalogenation encountered in Suzuki cross‐couplings.^[7]^ While competing β‐hydride elimination from intermediate π‐allyl Pd complexes to form dienes is known,^[8]^ this process is less reported and potentially reversible.^[9]^ However, dienes themselves frequently serve as useful synthetic intermediates,^[11]^ raising the possibility that their formation could form part of a productive catalytic cycle.^[11]^ Herein, we report a diverted Tsuji–Trost process, where β‐hydride elimination to form a reactive diene results in a novel tandem process, forming complex tertiary amines that represent the core of the biologically significant morphan ring‐system (Scheme 1 b).

Following our recently reported synthesis of lycorane alkaloid **4**,^[12]^ employing a key Heck cyclisation on a photochemically‐derived substrate, we were led to consider whether simple homologation of the carbon tether might lead directly to the homologated alkaloid series. However, initial investigation of the Heck reaction of iodide **10 a** in fact yielded deiodinated material **10 b** under the majority of conditions (Table 1). In no case was the desired Heck product detected, with use of previously successful phosphite ligands^[13]^ leading to the unexpected phosphonate ester **10 c** (Entry 4), presumably via reductive elimination to a phosphonium salt intermediate.^[14]^ However, the use of triphenylphosphine and dppf (Entries 7 and 8) led to the formation of bicyclic amine **11**. This process appeared to result from C−N bond cleavage with concurrent amine migration and reduction of the iodide moiety. Further screening of reaction conditions demonstrated that bicyclic amine **11** was formed in good yield through the use of DPEPhos (Entry 9), and that *i*Pr_2_NEt was required for this process to occur, with either no base or Et_3_N proving unsuccessful (Entries 10 and 11).

**Table 1:** Initial reaction screening.

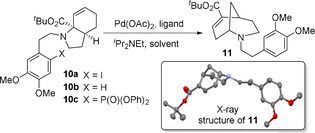				
Entry	Ligand	Solvent^[a]^	**10 b** [%]	**11** [%]
1	P(*o‐*tol)_3_	MeCN	<5	0
2	P(*o‐*tol)_3_	toluene	<5	0
3	P(*o‐*tol)_3_	dioxane	<5	0
4	P(OPh)_3_	MeCN	89^[b]^	0
5	XantPhos	dioxane	55	0
6	dppb	dioxane	0	3^[c]^
7	dppf	dioxane	0	21^[c]^
8	PPh_3_	dioxane	0	36^[c]^
9	DPEPhos	dioxane	0	76
10^d^	DPEPhos	dioxane	42	0
11^e^	DPEPhos	dioxane	0	0
12	CyDPEPhos	dioxane	0	6^[c]^

[a] All reactions were performed at reflux for 20 h. [b] Yield for phosphonate ester **10 c**, based on P(OPh)_3_. [c] Based on ^1^H NMR using 1,3,5‐trimethoxybenezene as an internal standard. [d] Et_3_N used instead of *i*Pr_2_NEt. [e] No amine added.

While this process was found to be relatively tolerant of variation of the aryl group (see SI for details), the inclusion of a sacrificial iodide moiety (i.e. X=I) proved essential for reactivity.^[15]^ As noted previously, the protodehalogenation of aryl halides is well documented within cross coupling reactions. Such a process has the potential to generate stoichiometric quantities of HX, which might then facilitate the observed cleavage of the C−N bond.^[16]^ Further evidence for this was obtained from a cross‐over reaction where a mixture of iodinated and non‐iodinated substrates led to product formation from both (see SI for details). We therefore investigated various additives (Table 2).

**Table 2:** Optimization study of reaction additives.

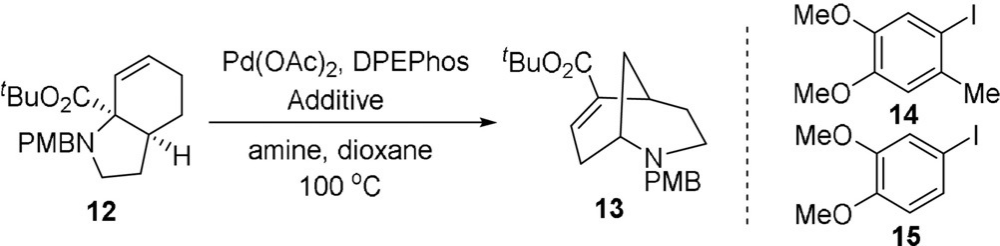			
Entry^[a]^	Amine (equiv)	Additive (equiv^[b]^)	**13** [%]
1	None	None	0
2	^*i*^Pr_2_NEt (2)	**14** (1)	50
3	^*i*^Pr_2_NEt (2)	**14** (0.5)	40
4	^*i*^Pr_2_NEt (2)	**15** (0.5)	26^[c]^
5	^*i*^Pr_2_NEt (2)	4‐iodoanisole (0.5)	23^[c]^
6	^*i*^Pr_2_NEt (2)	PhI (0.5)	19^[c]^
7	^i^Pr_2_NEt (2)	TBAI (1)	0
8	^*i*^Pr_2_NEt (2)	AcOH/TBAI (1)	0
9	none	^*i*^Pr_2_NEt.HI (1)	53
10	^*i*^Pr_2_NEt (0.2)	^*i*^Pr_2_NEt.HI (1)	39^[c]^
11	^*i*^Pr_2_NEt (1)	CSA (1)	43
12	^*i*^Pr_2_NEt (1)	MSA (1)	70

[a] All reactions were performed at reflux for 20 h. [b] Equivalents relate to molar quantity of starting material **12**. [c] Yield based on ^1^H NMR using 1,3,5‐trimethoxybenzene as internal standard. TBAI=tetrabutylammonium iodide. CSA=camphorsulfonic acid. MSA=methanesulfonic acid.

It can be seen that the use of an external electron‐rich aryl iodide led to efficient reaction (Entry 2). However stoichiometric quantities were required (Entry 3), and the use of simpler, less electron‐rich species was less effective (Entries 4–6). Use of iodide anion itself, either alone or in the presence of a weak acid proved ineffective (Entries 7 and 8). However, the use of the HI salt of *i*Pr_2_NEt proved a real breakthrough, obviating the need for a sacrificial aryl iodide (Entry 9). Exploring the required acid and amine stoichiometry led to further refinement, with a buffered system of 1 equiv. each of methanesulfonic acid and *i*Pr_2_NEt (Entry 12) proving optimal (see SI for complete acid study).

With these conditions in hand, we explored the scope of this reaction (Figure 1), the substrates being easily accessible via a simple two‐step process from pyrrole **1** (R=CO_2_ *t*Bu), involving photochemical conversion to tricyclic aziridine **7** followed by a one pot retro‐ene reaction/reductive amination sequence (see SI for details).[^6a^, ^6c^]

**Figure 1:**
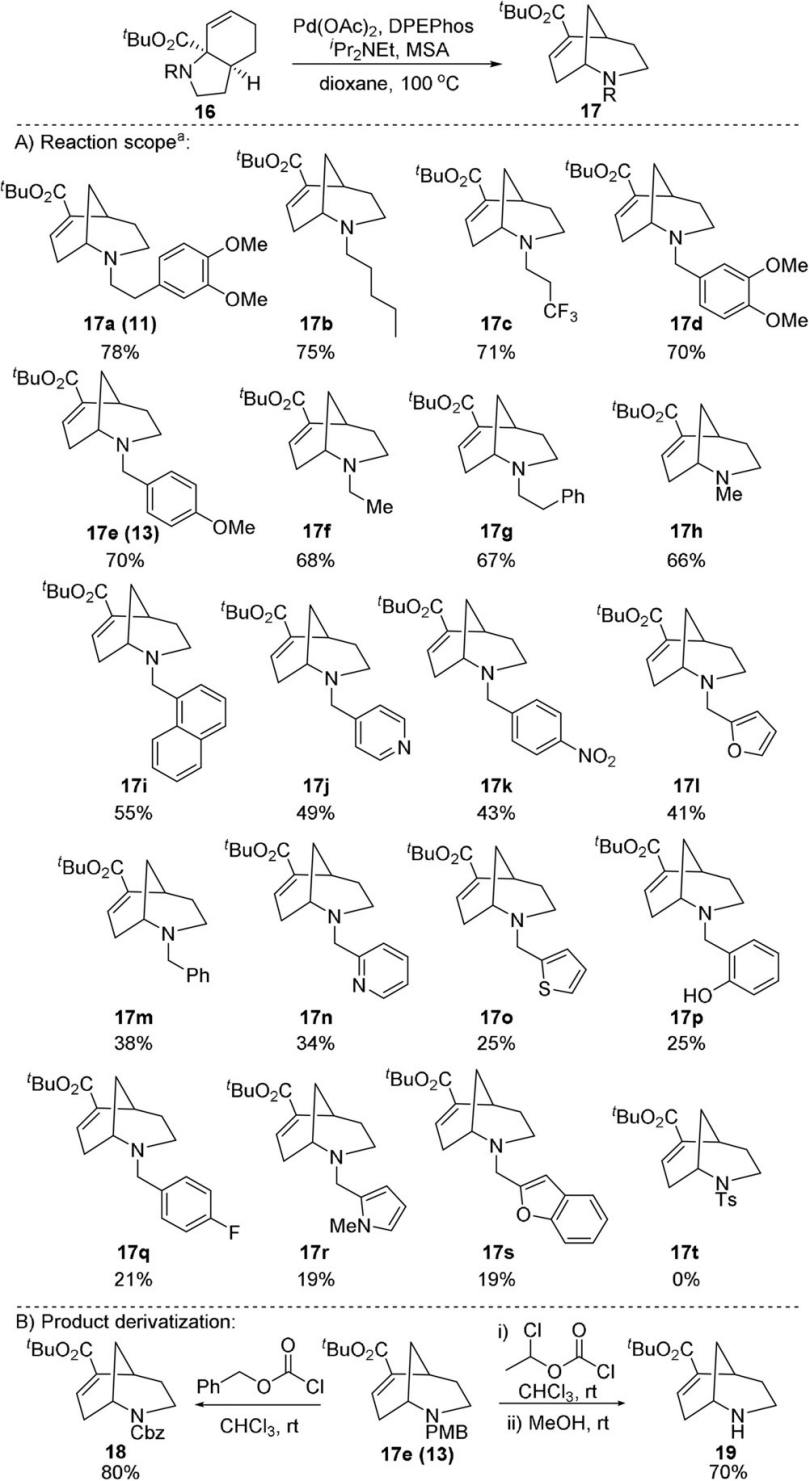
Reaction scope and product derivatization. [a] Pd‐catalysed reactions were performed using 10 mol % Pd(OAc)_2_, 20 mol % DPEPhos at 0.2 m concentration for 20 h. Amine to acid stoichiometry was 1:1.

The reaction proved very general, with a range of *N*‐alkyl, *N*‐benzyl and *N*‐homobenzyl substrates proceeding in good to moderate yield (**17 a–i**). Of particular note is the potential to include a simple methyl group (**17 h**), permitting access to *N*‐methyl morphan structures, and the medicinally important CF_3_ group (**17 c**).^[17]^ Given the importance of the morphan scaffold to medicinal chemistry,^[18]^ we also explored heterocyclic substituents. The reaction proved to tolerate a range of electron‐rich (**17 l**, **o**, **r**) and electron‐poor (**17 j**, **n**) heterocycles, albeit in reduced yield. *N*‐tosyl system (**17 t**) was also explored but proved unreactive.

The rapidity with which such complex, sp^3^‐rich aza‐systems can be reached from a single parent pyrrole is a significant highlight of the methodology, as is the ability to include reactive functional groups as in **17 p**. Importantly, *N*‐deprotection can be readily achieved to form **19**, permitting the installation of additional functionality on nitrogen in only two further steps. This could allow a practical approach to further expand the range of R groups in **17**. Exchange of PMB for the more versatile Cbz protecting group is conveniently achieved in a single step, as shown in the formation of **18**. This could be a significant advantage for a medicinal chemist wishing to prepare a 2D‐library of compounds by dual functionalization of the ester and amine moieties in **17**.

Having established the scope to be relatively broad, we turned our attention to the reaction mechanism. Formally a rearrangement, we considered that the process most likely involved acid‐assisted cleavage of the C−N bond forming a π‐allyl Pd intermediate, from which β‐hydride elimination formed a diene. This was tested by the addition of acetic anhydride to a reaction of substrate **12**, where uncyclized acetamide **20** was formed in good yield (Scheme 2). Stopping the reaction at an early stage also showed the presence of intermediate **21**, consistent with intramolecular 1,6‐addition to this diene. Re‐subjection of **21** to the reaction conditions showed conversion to **13** even in the absence of palladium. Furthermore, brief treatment of **21** to the optimized reaction conditions gave only **13** and no starting material **12** was detected. This latter experiment likely indicates that 1,6‐addition is not reversible.

**Scheme 2:**
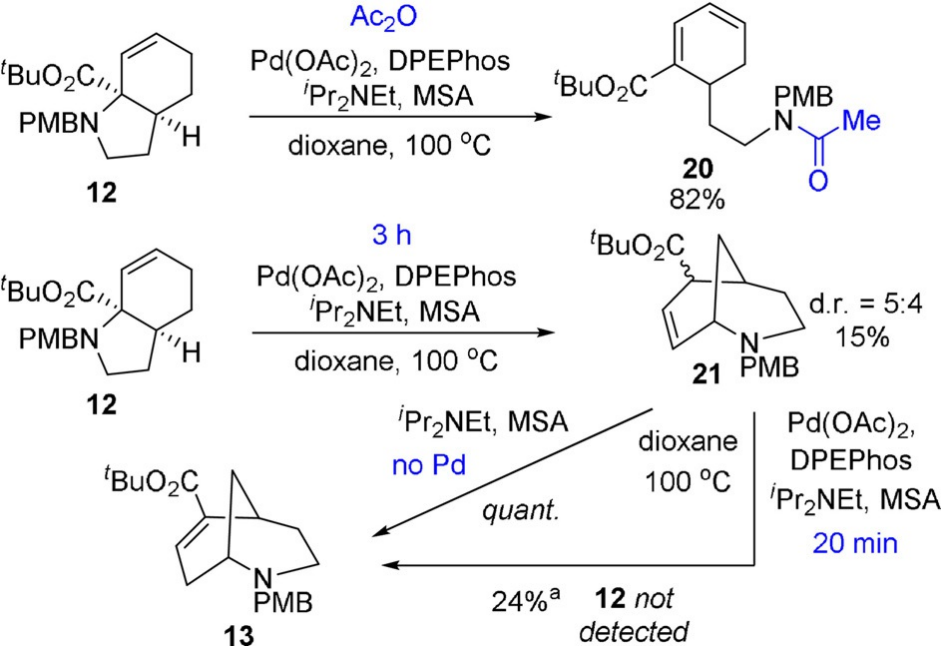
Investigation of trapping and intermediates. [a] Yield determined by ^1^H NMR using 1,3,5‐trimethoxybenzene as an internal standard. Reaction time of 20 h unless stated otherwise.

We then prepared deuterated compounds **22** and **23** and subjected these to the reaction conditions (Scheme 3). This led to a somewhat surprising results, with both compounds showing deuterium incorporation within the product; in fact, compound **24** showed a higher level of deuterium incorporation at the bridgehead (60 % vs. 35 %), despite an *anti*‐addition^[19]^/*syn*‐elimination^[20]^ mechanism being expected to result in selective cleavage of the C−D bond of **22** and the C−H bond of **23**. Assuming addition of palladium occurs *anti* to nitrogen, such behaviour suggests that facile equilibration of palladium between the *endo* and *exo* faces occurs within the π‐allyl Pd complex (vide infra). Further, a competition reaction between **22** and **12** (see Supporting Information for details) suggested no significant kinetic isotope effect was operating, although a secondary KIE, for instance during rate limiting π‐allyl complex formation, cannot be excluded.^[21]^

**Scheme 3:**
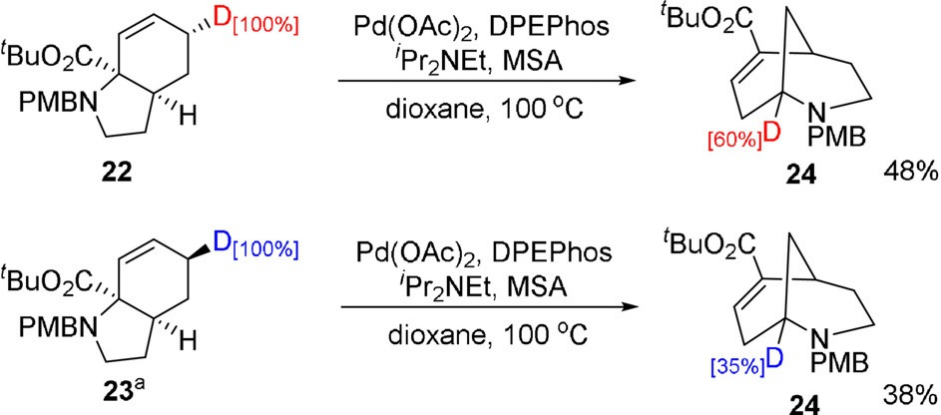
Deuterium‐labelling studies. [a] Substrate **24** contains a second remote deuterium atom (NCH_*endo*_ *D_exo_*) as a consequence of the synthetic route, which remained unchanged in the reaction (see the Supporting Information for full details).

Based on these results, a mechanism is proposed in Scheme 4. Initial acid‐promoted cleavage of the C−N bond by Pd^0^ forms π‐allyl Pd complex **25**. Based on the similar H/D ratios in the products of deuterated compounds **22** and **23**, this undergoes equilibration between faces, presumably by palladium *O*‐enolate **26**,^[22]^ with β‐hydride elimination thus being possible from either face to form diene **28**, and occurring somewhat preferentially from the *endo* face (i.e. from complex **27**). The exchange of Pd between the faces of the π‐allyl complex suggests this species has a significant lifetime, and this combined with the absence of the appreciable primary KIE generally associated with β‐hydride elimination,^[23]^ leaves open the possibility that this step to form diene **28** may be reversible. Trapping of this diene is possible through the inclusion of an electrophile such as acetic anhydride (Scheme 2), and otherwise this diene then undergoes irreversible 1,6‐conjugate addition to form intermediate **29** as a mixture of diastereomers. These species undergo acid/base‐promoted isomerization to the observed product. Related conjugated addition processes have been observed to occur under palladium catalysis.^[24]^

**Scheme 4:**
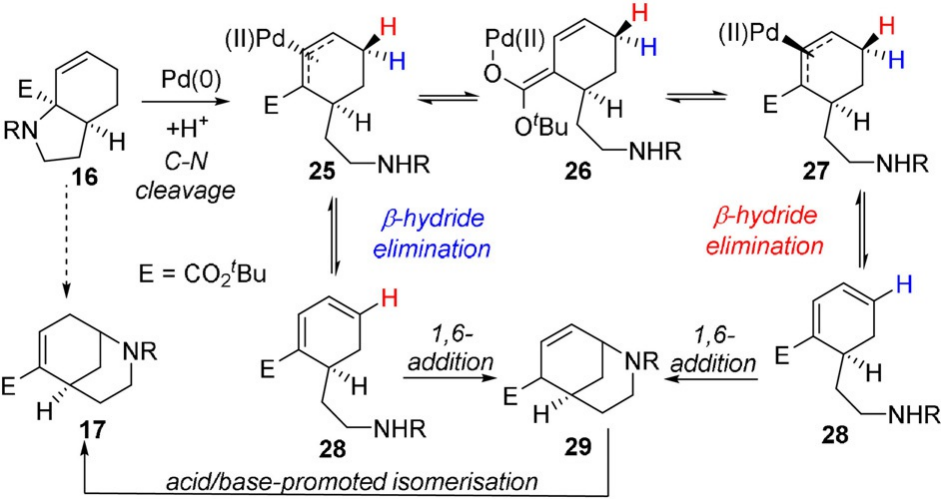
Proposed mechanism.

In conclusion, we have demonstrated that a diverted Tsuji–Trost process provides rapid access to biologically important ring systems. This occurs via an unusual Pd‐catalysed mechanism, exploiting processes often regarded as unwanted side reactions that is, proto‐dehalogenation, β‐hydride elimination and Pd *O*‐enolate equilibration. Overall, this methodology provides three‐step access to complex, biologically significant molecules from simple aromatic starting materials. The versatility of this chemistry could prove useful for medicinal chemists in the construction of 2D‐libraries based on the morphan scaffold, and once again highlights the power of combining photochemical synthesis with palladium catalysis.

## Conflict of interest

The authors declare no conflict of interest.

## Supporting information

As a service to our authors and readers, this journal provides supporting information supplied by the authors. Such materials are peer reviewed and may be re‐organized for online delivery, but are not copy‐edited or typeset. Technical support issues arising from supporting information (other than missing files) should be addressed to the authors.

Supplementary
