# Supplementary material for: Rapid Access to Azabicyclo[3.3.1]nonanes by a Tandem Diverted Tsuji–Trost Process
Source: Chemistry. 2020 Oct 6;26(63):14330–4. doi: 10.1002/chem.202003762 (PMC7702095; doi:10.1002/chem.202003762)
Supplement: Supplementary file 1 — Supplementary [file CHEM-26-14330-s001.pdf]

Chemistry–A European Journal

Supporting Information

Rapid Access to Azabicyclo[3.3.1]nonanes by a Tandem Diverted Tsuji–Trost Process

Hannah G. Steeds,[a] Jonathan P. Knowles,*[b] Wai L. Yu,[a] Jeffery Richardson,[c] Katie G. Cooper,[d] and Kevin I. Booker-Milburn*[a]

Table of Contents

General Information	S3
Experimental Procedures	S4
General Procedures	S4
Preliminary Scope of Halo-Aromatic Substituents	S4
Acid Screening Results	S4
Cross-over Experiment	S5
Competition Experiment	S5
Synthesis of Aziridine	S6
Synthesis of Aryl Iodide Substrates	S7
Synthesis of Aryl Triflate and Bromide	S10
Synthesis of Additives	S11
Synthesis of Substrates	S12
Synthesis of Morphan Products	S17
Intermediate Trapping	S23
Synthesis of Deuterated Materials	S24
Derivatization of Morphan	S28
Crystal Structure Data	S29
References	S30
Author Contributions	S30
NMR Spectra	S31

General Information

Chemicals were purchased and used without further purification. Dry solvents were obtained by passage through a column of anhydrous alumina using equipment from Anhydrous Engineering (University of Bristol) based on the Grubbs' design. Struass flasks fitted with a J. Youngs valve were used to collect anhydrous solvent. All other commercially available reagents were used as received. Reactions requiring anhydrous conditions were performed under N_2, glassware was flame dried immediately prior to use. Liquid reagents, solutions or solvents were added *via* syringe through rubber septa; solid reagents were added *via* Schlenk type adapters. Reaction mixtures were stirred magnetically. Reactions carried out at RT varied between 16-22 ºC depending on the season. The Pd-catalyzed rearrangement reactions were carried out in a glovebox.
Flash column chromatography was performed on Aldrich silica gel: 230-400 mesh (40-63 µm). Analytical thin layer chromatography was performed on aluminium backed 60 F_{254} silica plates. Visualisation was achieved by UV florescence (254 or 365 nm) and/or staining with $KMnO_4$ solution and heat. Extracts were concentrated *in vacuo* using both a Heidolph Hei-VAP Advantage rotary evaporator (bath temperatures up to 50 °C) at a pressure of 15 mmHg (diaphragm pump) and a high vacuum line at room temperature.
^{1}H-NMR and ^{13}C-NMR spectra were measured at 25 °C in the solvent specified with Varian, Jeol or Bruker spectrometers operating at field strengths listed. Chemical shifts (δ) are quoted in parts per million (ppm) with spectra referenced to the residual solvent peaks. Coupling constants (*J*) are reported in Hz and are reported as an average. Multiplicities are abbreviated as: s (singlet), d (doublet), t (triplet), q (quartet), qn (quintet), m (multiplet), br (broad), app (apparent) or combinations thereof. Assignments of ^{1}H-NMR and ^{13}C-NMR signals were made where possible, using COSY, HSQC and HMBC experiments. ^{1}H-NMR yields used 1,3,5-trimethoxy benzene as the internal standard.
Melting points were determined from a recrystallized material using Bibby Stuart SMP10 apparatus and are uncorrected.
Infra-red spectra were recorded in the range 4000-650 cm^{-1} on a Perkin Elmer Spectrum either as neat films or solids compressed onto a diamond window.
Mass spectra were determined by the University of Bristol mass spectrometry service by electrospray ionisation (ESI) mode or electron ionisation (EI) mode.
Numbering nomenclature in aziridine, imine and morphan systems are as shown below:

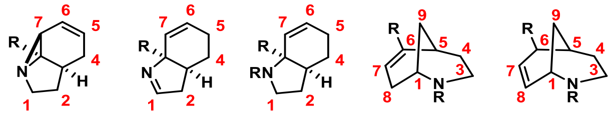

Experimental Procedures

General Procedures

General Procedure A: Reductive Amination
Sodium triacetoxyborohydride (3 equiv.) was added in one portion to a stirred solution of crude imine (1 equiv.) and aldehyde (1 equiv.) in CH_2Cl_2 (0.3 M) at 0 °C. The reaction was warmed to RT and stirred for 17 h, quenched with $NaHCO_3$ (saturated aqueous solution) and stirred for 10 min. The organic layer was extracted with CH_2Cl_2 (3 x) and the combined organic layers were dried over $MgSO_4$, filtered and concentrated *in vacuo*. The crude product was purified by SiO_2 flash chromatography.

General Procedure B: Reductive Amination with Oligomer Aldehydes (paraformaldehyde and 3,3,3-trifluoropropanal)
Sodium triacetoxyborohydride (3 equiv.) was added in one portion to a stirred solution of crude imine (1 equiv.) and aldehyde (3 equiv. paraformaldehyde, 1 equiv. 3,3,3-trifluoropropanal) in CH_2Cl_2 (0.3 M) at 0 °C. The reaction was warmed to 35 °C and stirred for 17 h, quenched with $NaHCO_3$ (saturated aqueous solution) and stirred for 10 min. The organic layer was extracted with CH_2Cl_2 (3 x) and the combined organic layers were dried over $MgSO_4$, filtered and concentrated *in vacuo*. The crude material was purified by SiO_2 flash chromatography.

General Procedure C: Pd catalysed 1,4 Rearrangement
Dioxane (0.06 M) and DIPEA (1 equiv.) were added to $Pd(OAc)_2$ (0.1 equiv.) and DPEPhos (0.15 equiv.), followed by the substrate (1 equiv.) in dioxane (0.06 M). The reaction mixture was stirred for 10 min, methanesulfonic acid was added (1 equiv.) and the reaction was heated under reflux. After 20 h the reaction mixture was cooled to RT and $NaHCO_3$ (sat. aqueous solution) was added. The organic layer was extracted with CH_2Cl_2 (3 x), dried over $MgSO_4$, filtered and concentrated *in vacuo*. The crude product was purified by SiO_2 flash chromatography.

Preliminary Reaction Scope of Halide Aromatic Precursors

TableS1. The effect of variation of aromatic substituent.

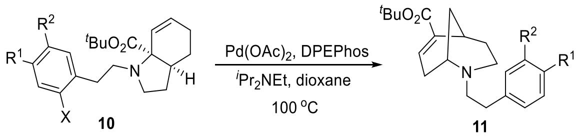

Entry[a]	X	R^1	R^2	Yield /%
1	I	MeO	MeO	76
2	I	H	H	74
3	I	F	H	33
4	Br	MeO	MeO	12[b]
5	H	MeO	MeO	0
6[c]	OTf	MeO	H	0

[a] All reactions carried out at reflux. [b] Yield based on ^{1}H-NMR using an internal standard. [c] Employed a single methylene unit within *N*-tether.

Acid Screening Results

Table S2. Optimization study of reaction additives.

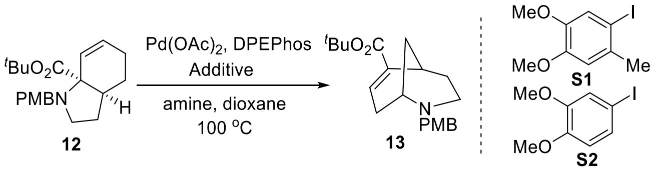

Entry[a]	Amine (eq)	Additive (eq.[b])	**13** /%

1	None	None	0
2	$^{i}Pr_2NEt$ (2)	**S1** (1)	50
3	$^{i}Pr_2NEt$ (2)	**S1** (0.5)	40
4	$^{i}Pr_2NEt$ (2)	**S1** (0.5)	26[c]
5	$^{i}Pr_2NEt$ (2)	4-iodoanisole (0.5)	23[c]
6	$^{i}Pr_2NEt$ (2)	PhI (0.5)	19[c]
7	$^{i}Pr_2NEt$ (2)	TBAI (1)	0
8	$^{i}Pr_2NEt$ (2)	AcOH/TBAI (1)	0
9	None	$^{i}Pr_2NEt$.HI (1)	53
10	None	$^{i}Pr_2NEt$.HI (2)	46c
11	$^{i}Pr_2NEt$ (0.2)	$^{i}Pr_2NEt$.HI (1)	39c
12	None	$^{i}Pr_2NEt$.HCl (1)	0
13	$^{i}Pr_2NEt$ (1)	Triisopropyl borate	0
14	$^{i}Pr_2NEt$ (1)	AcOH (1)	0
15	$^{i}Pr_2NEt$ (1)	TFA (1)	43[c]
16	$^{i}Pr_2NEt$ (1)	CSA (1)	43
17	$^{i}Pr_2NEt$ (1)	MSA (1)	70

[a] All reactions were performed at reflux for 20 h. [b] Equivalents relate to molar quantity of starting material **12**. [c] Yield based on ^{1}H-NMR using 1,3,5-trmethoxybenzne as an internal standard. TBAI = tetrabutylammonium iodide. TFA = trifluoroacetic acid. CSA = camphorsulfonic acid. MSA = methanesulfonic acid.

Cross-over Experiment

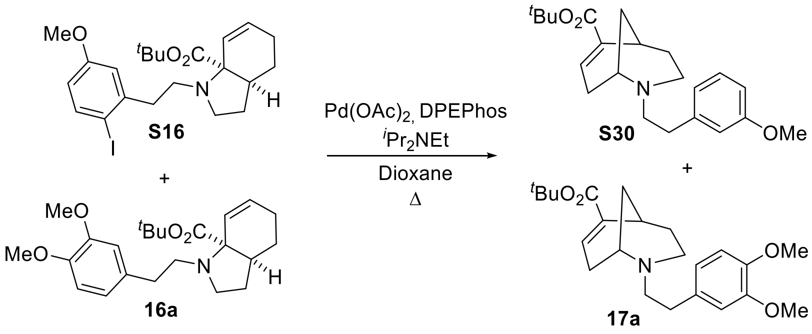

$^{i}PrNEt$ (0.070 mL, 0.4 mmol) was added to a stirred solution of $Pd(OAc)_2$ (5 mg, 0.02 mmol) and DPEPhos (19 mg, 0.04 mmol) in dioxane (0.5 mL). After 10 min, (±)-tert-butyl (3aS,7aR)-1-(2-iodo-5-methoxyphenethyl)-1,2,3,3a,4,5-hexahydro-7aH-indole-7a-carboxylate **S16** (69 mg, 0.14 mmol) and (±)-tert-butyl (3aS,7aR)-1-(3,4-dimethoxyphenethyl)-1,2,3,3a,4,5-hexahydro-7aH-indole-7a-carboxylate **16a** (53 mg, 0.14 mmol) as a solution in dioxane (1 mL) were added and the reaction mixture was heated under reflux for 17 h. The reaction was cooled to RT, $NaHCO_3$ (sat. aq. solution, 5 mL), was added and extracted with CH_2Cl_2 (3 x 5 mL). The combined organic layers were dried over $MgSO_4$, filtered and concentrated *in vacuo*. The crude material was purified by SiO_2 flash chromatography, eluting with 30-60% EtOAc/petrol to give the products as an inseparable mixture (47 mg, 1:0.3, **17a**:**S30**).

Competition Experiment

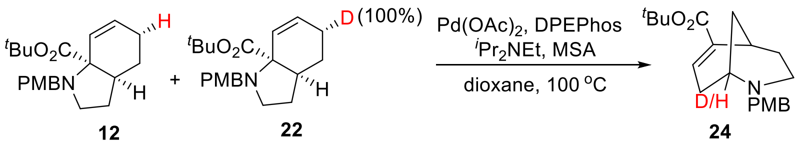

The lack of an appreciable kinetic isotope effect was determined by the reaction of an equimolar mixture of the deuterated compound 24 and non-deuterated compound 14. Stopping the reaction at several stages (Table S2) showed no significant change in the level of deuteration of either starting material or product. It can therefore be concluded that a primary kinetic isotope effect is likely not operating within the catalytic cycle, with kinetic isotope effect values for β-hydride elimination within π-ally palladium complexes reported as being in the range of 2.2 - 2.6.[1] More detailed kinetic studies of these processes are complicated by the presence of an induction period and by the formation of several intermediates over the course of the reaction. These studies were therefore performed as simple competition experiments, which as noted by Hartwig[2] may yield more information due to their ability to probe more than just the rate limiting step.

An equimolar solution of (±)-*tert*-butyl (3a*S*,5*R*,7a*R*)-1-(4-methoxybenzyl)-1,2,3,3a,4,5-hexahydro-7a*H*-indole-7a-carboxylate-5-*d* and (±)-*tert*-butyl (3a*S*,7a*R*)-1-(4-methoxybenzyl)-1,2,3,3a,4,5-hexahydro-7a*H*-indole-7a-carboxylate (0.075 mmol of each) in dioxane (0.6 mL) was added to a stirred solution of $Pd(OAc)_2$ (3 mg, 0.02 mmol), DPEPhos (12 mg, 0.02 mmol) and *N*,*N*-diisopropylethylamine (0.030 mL, 0.15 mmol) in dioxane (0.6 mL). After 10 min methanesulfonic acid (0.010 mL, 0.15 mmol) was added and the reaction was heated under reflux. After 30 mins, 60 mins and 120 mins the reaction mixture was cooled to RT and $NaHCO_3$ (sat. aqueous solution) was added. The organic layer was extracted with CH_2Cl_2 (3 x), dried over $MgSO_4$, filtered and concentrated *in vacuo*. The crude material was purified by SiO_2 flash chromatography, eluting with 10-20% EtOAc/petrol to separate the unreacted starting material and the morphan product.

Table S3. Competition experiment.

Conversion %[a]	H/D SM	H/D Product
41	1:1	1:1
78	1:1	1:1
84	1:1	1:1

[a] Percentage conversion is calculated from a determination of level of starting material based on crude ^{1}H-NMR.

Synthesis of Aziridine

1-(1-(but-3-en-1-yl)-1*H*-pyrrol-2-yl)-2,2,2-trichloroethan-1-one S3

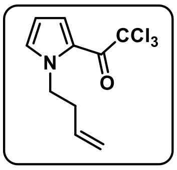

Diisopropyl azodicarboxylate (40.0 mL, 203 mmol) was added dropwise over 15 min to a stirred solution of triphenylphosphine (52.0 g, 199 mmol) in THF (600 mL) at -78 °C. The reaction mixture turned bright yellow after this addition. The mixture was stirred for 40 min and 3-buten-1-ol (19.0 mL, 221 mmol) was added dropwise over 5 min. After a further 1 h, 2-(trichloroacetyl)pyrrole (41.4 g, 194.86 mmol) was added in one portion and the reaction mixture was warmed to RT. After 20 h, the reaction mixture was concentrated *in vacuo* and the residue triturated in Et_2O/Petrol (2:3, 500 mL) at -10 °C and filtered. The residue was concentrated *in vacuo* and purified by SiO_2 flash chromatography, eluting with Petrol, to give the product as a light-yellow oil (46.87 g, 90%). ^{1}H-NMR (400 MHz, $CDCl_3$) δ 7.54 (dd, 1H, *J* = 4.4, 1.4 Hz, ArCH), 7.00 (t, 1H, *J* = 2.1 Hz, ArCH), 6.22 (dd, 1H, *J* = 4.4, 2.1 Hz, ArCH), 5.82-5.71 (m, 1H, $C\underline{H}CH_2$), 5.07-5.03 (m, 2H, $CHC\underline{H_2}$), 4.39 (t, 2H, *J* = 7.1 Hz, NCH_2), 2.51 (app q, 2H, $NCH_2C\underline{H_2}$); ^{13}C-NMR (100 MHz, $CDCl_3$) δ 172.6 (CO), 134.0 ($\underline{C}HCH_2$), 133.2 (ArCH), 124.7 (ArCH), 121.0 (ArC), 117.8 ($CH\underline{C}H_2$), 108.9 (ArCH), 96.5 (C), 50.1 (NCH_2), 35.4 ($NCH_2\underline{C}H_2$). ^{1}H-NMR an ^{13}C-NMR data is consistent with the literature.[3]

1-(buten-3-en-1-yl)-1*H*-pyrrole-2-carboxylic acid S4

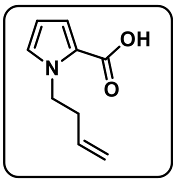

NaOH (2M aqueous solution, 190 mL) was added to a stirred solution of 1-(1-(but-3-en-1-yl)-1*H*-pyrrol-2-yl)-2,2,2-trichloroethan-1-one **S3** (31.59 g, 119.4 mmol) in THF (104 mL). After 17 the reaction mixture was cooled to 0 °C and quenched with HCl (3M aqueous solution, 150 mL). The mixture was extracted with Et_2O (2 x 150 mL) and the combined organic layers were dried over $MgSO_4$, filtered and concentrated *in vacuo*. The crude product was recrystallized from Petrol to give a yellow solid (17.79 g, 90%). ^{1}H-NMR (400 MHz, MeOD) δ 6.93-6.92 (m, 2H, ArCH), 6.07-6.05 (m, 1H, ArCH), 5.81-5.71 (m, 1H, $C\underline{H}$), 5.02-4.96 (m, 2H, CH), 4.89 (br s, 1H, OH), 4.36 (t, 2H, *J* = 7.1 Hz, NCH_2), 2.47 (app q, 2H, $CH_2C\underline{H_2}$); ^{13}C-NMR (100 MHz, MeOD) δ 164.2 (CO), 136.0 ($\underline{C}HCH_2$), 130.4 (ArCH), 122.8 (ArC), 119.9 (ArCH), 117.4 ($CH\underline{C}H_2$), 108.7 (ArCH), 49.5 (NCH_2), 37.2 ($NCH_2\underline{C}H_2$) υ_{max}/cm^{-1} 2919.38, 1659.83, 1532.80, 1428.44, 1326.06, 1256.79, 1107.77, 1073.59, 917.06, 739.51; *m/z* HRMS (Nanospray) found $[M - H]^-$ 164.0711 and $[M - 2H + Na]^-$ 186.1150, $[C_9H_{10}NO_2]^+$ requires 164.0712 and $[C_9H_9NO_2Na]^+$ requires 186.0530; m.p. 48 - 49 ˚C (Petrol).

tert-Butyl 1-(but-3-en-1-yl)-1*H*-pyrrole-2-carboxylate S5

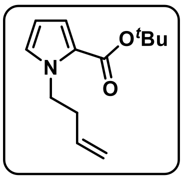

Oxalyl chloride (10.90 mL, 128.9 mmol) was added dropwise over 55 min to a stirred solution of 1-(buten-3-en-1-yl)-1*H*-pyrrole-2-carboxylic acid **S4** (17.75 g, 107.5 mmol) and DMF (6 drops) in CH_2Cl_2 (245 mL) at -10 °C. The reaction mixture was warmed to RT for 40 min then cooled to 0 °C and potassium *tert*-butoxide (35.17 g, 322.4 mmol) was added portion wise over 20 min. The reaction was warmed to RT, stirred for 2.5 h and quenched with H_2O (200 mL). The mixture was separated, extracted with CH_2Cl_2 (2 x 200 mL), washed with brine (400 mL), dried over $MgSO_4$, filtered and concentrated *in vacuo*. The crude material was purified by SiO_2 flash chromatography, eluting with 0-5% EtOAc/Petrol, to give the product as a light yellow oil (12.94 g, 54%). ^{1}H-NMR (400 MHz, $CDCl_3$) δ 6.89-6.87 (m, 1H, ArCH), 6.77 (br s, 1H, ArCH), 6.08-6.06 (m, 1H, ArCH), 5.81-5.71 (m, 1H, CHCH$_2$), 5.06-5.01 (m, 2H, CHCH$_2$), 4.34 (t, 2H, *J* = 7.0 Hz, NCH$_2$), 2.51 (app q, 2H, NCH$_2$CH$_2$), 1.55 (s, 9H, CH$_3$); ^{13}C-NMR (100 MHz, $CDCl_3$) δ 160.6 (CO), 134.7 (CHCH$_2$), 128.1 (ArCH), 123.2 (ArC), 117.9 (CHCH$_2$), 117.1 (ArCH), 107.4 (ArCH), 80.2 (C), 48.7 (NCH$_2$), 36.0 (NCH$_2$CH$_2$), 28.4 (CH$_3$). ^{1}H-NMR and ^{13}C-NMR data is consistent with the literature.[4]

(±)-*tert*-Butyl (3^1*R*,3aS,6a*S*)-1,3a,6,6a-terahydroazirino[2,3,1-*hi*]indole-3^1(2*H*)-carboxylate S6

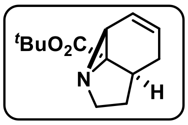

tert-Butyl 1-(but-3-en-1-yl)-1*H*-pyrrole-2-carboxylate **S5** (1.00 g, 4.52 mmol) in degassed cyclohexane:EtOAc (5.7:1, 470 mL) was irradiated using a, water cooled, 36 W low pressure Hg lamp. After 14 h the irradiation was stopped, and the reaction mixture was concentrated *in vacuo*. The crude material was purified by SiO_2 flash chromatography, eluting with 20-50% EtOAc/Petrol, to give a yellow oil (0.41 g, 41%). ^{1}H-NMR (400 MHz, $CDCl_3$) δ 6.24-6.19 (m, 1H, 5-CH), 5.78 (dt, 1H, *J* = 10, 3.5 Hz, 6-CH) 3.24-3.16 (m, 2H, 1-CH and 3-CH), 2.84 (d, *J* = 3.5 Hz, 1H, 7-CH), 2.60-2.47 (m, 2H, 1-CH and 2-CH), 2.30 (br d, *J* = 18.2 Hz, 1H, 4-CH), 1.91 (dd, *J* = 18.0, 6.1 Hz, 1H, 4-CH), 1.52 (m, 1H, 2-CH), 1.47 (s, 9 H, CH$_3$); ^{13}C-NMR (100 MHz, $CDCl_3$) δ 171.6 (CO), 135.0 (6-CH), 120.7 (5-CH), 81.2 (C), 52.4 (C), 49.8 (1-CH$_2$), 43.5 (7-CH), 41.2 (2-CH$_2$), 33.5 (3-CH), 29.5 (4-CH$_2$), 28.0 (CH$_3$). ^{1}H-NMR and ^{13}C-NMR data is consistent with literature.[4]

(±)-*tert*-Butyl (3a*S*,7a*R*)-3-3a,4,5-tetrahydro-7a*H*-indole-7a-carboxylate S7

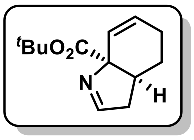

(±)-*tert*-Butyl (3^1*S*,3a*R*,6a*R*)-1,3a,6,6a-tetrahydoazirino[2,3,1-*hi*]indole-3^1(2H) carboxylate **S6** (0.70 g, 3.2 mmol) was stirred in PhMe (30 mL) at 100 °C for 15 h. The reaction mixture was concentrated *in vacuo* to give the crude material as a brown oil (0.70 g, 100%) which was used directly in the next step. ^{1}H-NMR (400 MHz, $CDCl_3$) δ 7.64 (s, 1H, 1-CH), 6.05-5.95 (m, 2H, 6-CH and 7-CH), 2.86-2.70 (m, 2H, 2-CH and 3-CH), 2.42-2.35 (m, 1H, 2-CH), 2.03-1.98 (m, 2H, 5-CH$_2$), 1.87-1.81 (m, 2H, 4-CH$_2$) 1.46 (s, 9H, CH$_3$). ^{1}H-NMR data is consistent with literature.[5]

***(±)-tert*-Butyl (3a*S*,7a*R*)-1,2,3,3a,4,5-hexahydro-7a*H*-indole-7a-carboxylate S8**

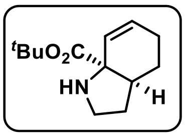

A stirred solution of aziridine **S6** (233 mg, 1.05 mmol) in toluene (12 mL) was heated to 100 °C under nitrogen. After 16 h the reaction was evaporated to give a brown oil which was redissolved in anhydrous DCM (3.5 mL) and cooled to 0 °C. AcOH (0.080 mL, 1.3 mmol) was added, followed by NaBH(OAc)$_3$ (260 mg, 1.23 mmol) in one portion. The reaction was allowed to warm to rt, stirred for 13 h and quenched by the addition of sat. aq. $NaHCO_3$ (10 mL). The mixture was stirred for 15 min and extracted with DCM (2 × 10 mL). Drying ($MgSO_4$) and evaporation gave a brown oil which was purified by silica gel chromatography (EtOAc/petrol, 1:1 to 9:1 as eluent) to afford the title compound (164 mg, 70% over two steps) as a light yellow oil. ν_{max}/cm^{-1} (film) 2929, 1717, 1367 and 1254; δ_H (500 MHz, $CDCl_3$) 1.47 (9H, s, *Me*$_3$C), 1.53 – 1.68 (2H, m, homoallylic CH*H* and NCH2CH*H*), 1.84 – 1.97 (2H, m, homoallylic C*H*H and NCH$_2$C*H*H), 1.97 – 2.13 (2H, m, allylic C*H*$_2$), 2.43 – 2.51 (1H, m, CH), 2.95 – 3.01 (2H, m, NC*H*$_2$), 5.55 (1H, dt, J 9.9, 2.1, C*H*=CH-CH) and 5.92 (1H, dt, J 10.0, 4.0, =C*H*-CH); δ_C (126 MHz, $CDCl_3$) 21.7 (allylic *C*H$_2$), 24.5 (homoallylic *C*H$_2$), 27.9 (*Me*$_3$C), 30.2 (NCH$_2$CH$_2$), 40.0 (CH), 44.4 (N*C*H$_2$), 67.0 (C$_q$-CO$_2$^{t}Bu), 81.0 (Me$_3$*C*O), 128.5 (*C*H=CH-CH), 129.5 (=*C*H-CH) and 175.1 (CO$_2$^{t}Bu); HRMS (ESI$^+$) 224.1652 ($C_{13}H_{22}NO_2$, [M+H]$^+$, requires 224.1645).

Synthesis of Aryl Iodide Substrates

(2-Iodo-4,5-dimethoxyphenyl)ethanol S9

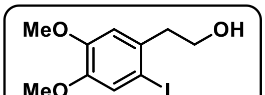

Iodomonochloride (2.50 g, 15.4 mmol) in CH_2Cl_2 (8 mL) was added dropwise over 10 min to a stirred solution of 2-(3,4-dimethoxyphenyl)ethanol (2.00 g, 11.0 mmol) in CH_2Cl_2 (34 mL). After 26.5 h the reaction mixture was quenched with $Na_2S_2O_3$ (1M aqueous solution, 30 mL) and extracted with CH_2Cl_2 (3 x 20 mL). The combined organic layers were dried over $MgSO_4$, filtered and concentrated *in vacuo*. The crude material was purified by SiO_2 flash chromatography, eluting with 10-40% EtOAc/Petrol, which

was recrystallised from Et_2O to give the product as a light pink solid (1.81 g, 54%). ^{1}H-NMR (400 MHz, $CDCl_3$) δ 7.22 (s, 1H, ArCH), 6.78 (s, 1H, ArCH), 3.85 (s, 3H, OCH_3), 3.84 (s, 3H, OCH_3), 3.82 (m, 2H, C$\underline{H_2}$OH), 2.94 (t, 2H, *J* = 6.7 Hz, $ArCH_2$), 1.48 (br s, 1H, OH); ^{13}C-NMR (100 MHz, $CDCl_3$) δ 149.3 (ArC), 148.2 (ArC), 133.43 (ArCH), 121.8 (ArC), 113.0 (ArCH), 88.2 (ArC), 62.5 (CH_2OH), 56.1 (OCH_3), 55.9 (OCH_3) 43.2 ($ArCH_2$); m.p. 58-59 ˚C (Et_2O), lit. 53-54˚C.[4] ^{1}H-NMR and ^{13}C-NMR data is consistent with literature.[4]

2-(2-Iodo-4,5-dimethoxyphenyl)acetaldehyde S10

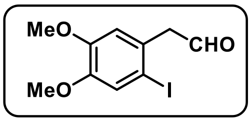

Dess Martin periodinane (2.94 g, 6.94 mmol) was added in one portion to a stirred solution of (2-iodo-4,5-dimethoxphenyl)ethanol **S9** (1.78 g, 5.78 mmol) in CH_2Cl_2 (24 mL). After 16.5 h the reaction was quenched with $Na_2S_2O_3$ (1M aqueous solution, 30 mL) and extracted with CH_2Cl_2 (3 x 10 mL). The combined organic layers were washed with $NaHCO_3$ (saturated aqueous solution, 30 mL) dried over $MgSO_4$, filtered and concentrated *in vacuo*. The crude product was purified by SiO_2 flash chromatography, eluting with 0-20% EtOAc/Petrol, to yield the product as a yellow solid (1.26 g, 72%). ^{1}H-NMR (400 MHz, $CDCl_3$) δ 9.73 (s, 1H, CHO), 7.26 (s, 1H, ArCH), 6.70 (s, 1H, ArCH), 3.86 (s, 3H, OCH_3), 3.84 (s, 3H, OCH_3), 3.81 (m, 2H, CH_2); ^{13}C-NMR (100 MHz, $CDCl_3$) δ 199.7 (CHO), 149.6 (ArC), 148.9 (ArC), 128.2 (ArC), 121.7 (ArCH), 113.3 (ArCH), 88.9 (ArC), 56.2 (OCH_3), 55.9 (OCH_3), 54.3 (CH_2); m.p. 70-71 ˚C (EtOAc/Petrol), lit. 53-56 ˚C.[6] ^{1}H-NMR data is consistent with literature.[6]

***(±)-tert*-Butyl (3a*S*,7a*R*)-1-(2-iodo-4,5-dimethoxyphenethyl)-1,2,3,3a,4,5-hexahydro-7a*H*-indole-7a-carboxylate 10a**

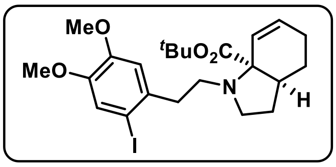

According to general procedure A, crude (±)-*tert*-butyl (3a*S*,7a*R*)-3-3a,4,5-tetrahydro-7a*H*-indole-7a-carboxylate **S7** (0.650 g, 2.92 mmol) and 2-(2-iodo-4,5-dimethoxyphenl)acetaldehyde **S10** (0.890 g, 2.92 mmol) were stirred with sodium triacetoxyborohydride (1.86 g, 8.76 mmol) in CH_2Cl_2 (11 mL). The crude product was purified by SiO_2 flash chromatography, 0-30% EtOAc/Petrol, to give a yellow oil (1.02 g, 68%). ^{1}H-NMR (400 MHz, $CDCl_3$) δ 7.18 (s, 1H, ArCH), 6.79 (s, 1H, ArCH), 5.93 (dt, 1H, *J* = 10.3, 3.8 Hz, 6-CH), 5.76 (br dt, 1H, *J* = 10.3, 1.8 Hz, 7-CH), 3.83 (s, 6H, 2 x OCH_3), 3.07 (m, 1H, 1-CH), 2.91-2.80 (m, 4H, 1-CH, NCH$\underline{H}$, $NCH_2C\underline{H_2}$), 2.72-2.66 (m, 1H, NC$\underline{H}$H), 2.57-2.50 (m, 1H, 3-CH), 2.14-1.93 (m, 3H, 2-CH and 4-CH_2), 1.75-1.68 (m, 1H, 5-CH), 1.62-1.54 (m, 2H, 2-CH and 5-CH), 1.45 (s, 9H, CH_3); ^{13}C-NMR (100 MHz, $CDCl_3$) δ 173.7 (CO), 149.2 (ArC), 147.8 (ArC), 135.9 (ArC), 130.3 (6-CH), 124.6 (7-CH), 121.5 (ArCH), 112.6 (ArCH), 88.1 (ArC), 80.8 ($\underline{C}(CH_3)_3$), 69.1 (C), 56.1 (OCH_3), 55.9 (OCH_3), 50.6 (NCH_2), 50.4 (1-CH), 40.5 (3-CH), 40.3 ($NCH_2\underline{C}H_2$), 28.2 (CH_3), 27.4 (5-CH_2), 25.3 (4-CH_2), 22.2 (2-CH_2); υ_{max}/cm^{-1} 2929.98, 2970.68, 1715.44, 1503.59, 1454.91, 1367.24, 1217.24, 1252.66, 1159.37, 1029.15; *m/z* HRMS (ESI$^+$) found [M + H]$^+$ 514.1441, [$C_{23}H_{33}INO_4$]$^+$ requires 514.1449.

(*E*)-1-iodo-2-(2-methoxyvinyl)benzene S11

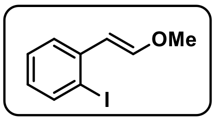

To a stirred suspension of methoxymethyl triphenylphosphonium bromide (3.4 g, 9.9 mmol) in dry THF (20 mL) at 0 °C under nitrogen was added KO^{t}Bu (1.08 g, 9.6 mmol) portionwise. The resulting deep orange solution was stirred for 15 min and substrate (1.00 g, 4.3 mmol) added portionwise. The reaction was stirred for 1 h, warmed to rt, stirred for a further 2 h and quenched with water (20 mL). The mixture was extracted with Et_2O (40 mL) and the organic phase washed with brine (25 mL), dried ($MgSO_4$) and evaporated to give an orange oil. Purification by silica gel chromatography (EtOAc/petrol, 5:95 to 1:9 as eluent) afforded the title compound (1.16 g, 100%) as a clear oil. ^{1}H-NMR data is consistent with literature.

2-(2-Iodophenyl)acetaldehyde S12

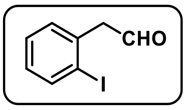

To a stirred solution of substrate **S11** (0.58 g, 2.2 mmol) in acetone (5 mL) at rt was added 2 M aq. HCl (0.5 mL) and the mixture heated to 60 °C. After 2.5 h the reaction was cool, evaporated, diluted with water (10 mL) and DCM (10 mL), sat. aq. $NaHCO_3$ (5 mL) added, the phases separated and the aqueous phase reextracted with DCM (10 mL). The combined organic phase was dried ($MgSO_4$) amd evaporated to give a light yellow oil. Purification by silica gel chromatography (EtOAc/petrol, 5:95 to 1:9 as eluent) afforded the title compound (338 mg, 62%) as a clear oil. ^{1}H-NMR data is consistent with literature.[7]

(±)-*tert*-Butyl (3a*S*,7a*R*)-1-(2-iodo-phenethyl)-1,2,3,3a,4,5-hexahydro-7a*H*-indole-7a-carboxylate S14

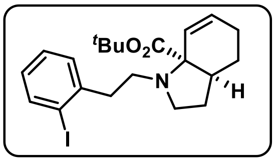

To a stirred solution of substrate **S7** (120 mg, 0.54 mmol) and aldehyde **S12** (140 mg, 0.57 mmol) in anhydrous DCM (5 mL) at 0 °C under nitrogen was added $NaBH(OAc)_3$ (360 mg, 1.7 mmol) in one portion. The reaction was warmed to rt and stirred overnight. After 13 h the reaction was quenched by the addition of sat. aq. $NaHCO_3$ (10 mL), stirred for 15 min and extracted with DCM (2 × 10 mL). Drying ($MgSO_4$) and evaporation gave a yellow oil. Purification by silica gel chromatography (EtOAc/petrol, 5:95 to 1:9 as eluent) afforded the title compound (168 mg, 69% over two steps) as a clear oil. ν_{max} /cm^{-1} (film) 2972, 1715, 1455, 1366, 1249 and 1154; δ_H (500 MHz, $CDCl_3$) 1.48 (9H, s, Me_3C), 1.55 – 1.66 (2H, m, NCH_2CH*H* and homoallylic CH*H*), 1.74 (1H, ddt, J 12.9, 7.9, 5.1, homoallylic C*H*H), 1.93 – 2.16 (3H, m, NCH_2C*H*H and allylic CH_2),

2.57 (1H, tt, J 9.8, 5.7, CH), 2.68 – 2.79 (1H, m, NCH*H*), 2.83 – 2.97 (4H, m, benzylic C*H*$_2$, NC*H*H and NCH*H*), 3.10 (1H, td, J 8.8, 4.6 NC*H*H), 5.77 (1H, dt, J 10.3, 2.1, C*H*=CH-CH), 5.95 (1H, dt, J 10.2, 3.8, =C*H*-CH), 6.89 (1H, ddd, J 7.9, 5.5, 3.5, CH_{Ar}), 7.24 – 7.30 (2H, m, 2 × CH_{Ar}) and 7.81 (1H, dt, J 8.0, 0.9, CH_{Ar})); δ_C (126 MHz, $CDCl_3$) 22.2 (allylic *C*H$_2$), 25.2 (homoallylic CH_2), 27.3 (NCH_2*C*H$_2$), 28.2 (*Me*$_3$C), 40.4 (CH), 40.8 (benzylic CH_2), 50.4 (NCH_2), 50.6 (NCH_2), 69.1 (C_q-$CO_2{}^tBu$), 80.8 (Me_3*C*O), 100.7 (CI_{Ar}), 124.5 (*C*H=CH-CH), 127.8 (CH_{Ar}), 128.2 (CH_{Ar}), 129.9 (CH_{Ar}), 130.4 (=*C*H-CH), 139.3 (CH_{Ar}), 143.4 (C_{qAr}) and 173.7 (*C*O$_2{}^t$Bu); HRMS (ESI^+) 454.1243 ($C_{21}H_{29}INO_2$, $[M+H]^+$, requires 454.1238).

2-Iodo-5-methoxybenzaldehyde S15

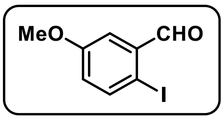

To a stirred solution of meta-anisaldehyde (2.2 mL, 18 mmol) and *N*-iodosuccinimide (4.85 g, 21.6 mmol) in anhydrous MeCN (30 mL) at rt under nitrogen was added TfOH (5 drops) and the stirred mixture heated to 80 °C. After 13 h the reaction was cooled to rt and quenched by the addition of 1M aq. sodium thiosulphate (20 mL). The mixture was stirred for 15 min, diluted with water (60 mL) and extracted with Et_2O (2 × 50 mL). Drying ($MgSO_4$) and evaporation gave a light yellow solid which was purified by silica gel chromatography (EtOAc/petrol, 1:9 as eluent) to afford the title compound (2.59 g, 55%) as a white solid. Further product (1.27 g) was also recovered but contaminated with di-iodide. ^1H-NMR data is consistent with literature.[7]

(*E*)-1-Iodo-4-methoxy-2-(2-methoxyvinyl)benzene S16

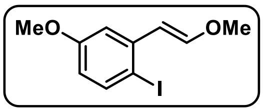

To a stirred suspension of methoxymethyl triphenylphosphonium bromide (7.54 g, 22 mmol) in dry THF (40 mL) at 0 °C under nitrogen was added KOtBu (2.39 g, 21.3 mmol) portionwise. The resulting deep orange solution was stirred for 15 min and a solution of substrate **S15** (2.50 g, 9.54 mmol) in anhydrous THF (20 mL) was added dropwise. The reaction was warmed to rt, stirred for a further 2 h and quenched with water (60 mL). The mixture was extracted with Et_2O (2 × 50 mL) and the combined organic phase washed with brine (70 mL), dried ($MgSO_4$) and evaporated to give an orange oil. Purification by silica gel chromatography (EtOAc/petrol, 4:96 to 6:94 as eluent) afforded the title compound (2.06 g, 74%) as a clear oil. ^1H-NMR data is consistent with literature.[7]

2-(2-Iodo-5-methoxyphenyl)acetaldehyde S17

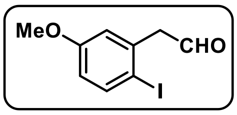

To a stirred solution of substrate **S16** (2.00 g, 6.90 mmol) in acetone (15 mL) at rt was added 2 M aq. HCl (2.0 mL) and the mixture heated to 60 °C. After 2.5 h the reaction was cooled to rt, evaporated and partitioned between DCM (40 mL) and half saturated $NaHCO_3$ (40 mL). The phases separated and the aqueous phase reextracted with DCM (20 mL). The combined organic phase was dried ($MgSO_4$) and evaporated to give a yellow oil. Purification by silica gel chromatography (EtOAc/petrol, 2:98 to 7:93 as eluent) afforded the title compound (1.42 g, 75%) as a clear oil. ^1H-NMR and ^{13}C-NMR data are consistent with literature.[7]

(±)-*tert*-Butyl (3a*S*,7a*R*)-1-(2-iodo-5-methoxyphenethyl)-1,2,3,3a,4,5-hexahydro-7a*H*-indole-7a-carboxylate S18

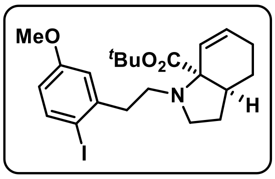

To a stirred solution of substrate **S7** (120 mg, 0.54 mmol) and aldehyde **S17** (150 mg, 0.54 mmol) in anhydrous DCM (5 mL) at 0 °C under nitrogen was added $NaBH(OAc)_3$ (360 mg, 1.7 mmol) in one portion. The reaction was warmed to rt and stirred overnight. After 13 h the reaction was quenched by the addition of sat. aq. $NaHCO_3$ (10 mL), stirred for 15 min and extracted with DCM (2 × 10 mL). Drying ($MgSO_4$) and evaporation gave a yellow oil. Purification by silica gel chromatography (EtOAc/petrol, 5:95 to 15:85 as eluent) afforded the title compound (203 mg, 78% over two steps) as a clear oil.ν_{max} /cm^{-1} (film) 2931, 1716, 1589, 1567, 1465 and 1236; ^1H-NMR (400 MHz, $CDCl_3$) δ 7.64 (d, 1H, *J* = 9.0 Hz, ArCH), 6.83 (br s, 1H, ArCH), 6.50 (dd, 1H, *J* = 9.0, 3.2 Hz, ArCH), 5.93 (br s, 1H, 6-CH), 5.75 (d, 1H, *J* = 10.5 Hz, 7-CH), 3.76 (s, 3H, OCH_3), 3.07 (br s, 1H, 1-CH), 2.90-2.83 (m, 4H, 1-CH, NC<u>H</u>H and $NCH_2C\underline{H}_2$), 2.75-2.71 (m, 1H, NCH<u>H</u>), 2.54 (br s, 1H, 3-CH), 2.14-1.94 (m, 3H, 2-CH and 4-CH_2), 1.74-1.68 (m, 1H, 5-CH), 1.62-1.53 (m, 2H, 2-CH and 5-CH), 1.46 (s, 9H, CH_3); HRMS (ESI^+) 484.1345 ($C_{22}H_{31}INO_3$, $[M+H]^+$, requires 484.1343).

(5-Fluoro-2-iodophenyl)methanol S19

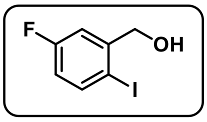

Borane tetrahydrofuran complex (1M solution, 11.3 mL, 11.3 mmol) was added dropwise over 20 min to a stirred solution of 5-fluoro-2-iodobenzoic acid (2.0 g, 7.5 mmol) in THF (15 mL) at 0 ˚C. The reaction mixture was warmed to RT, stirred for 6 hour then quenched with H_2O:THF (1:1, 15 mL) and solid K_2CO_3. The reaction was extracted with Et_2O (3 x 20 mL), dried over $MgSO_4$, filtered and concentrated *in vacuo*. The crude product was purified by SiO_2 flash chromatography, 20-40% EtOAc/Petrol, to give a colourless solid (0.58 g, 31%). ^1H-NMR (400 MHz, MeOD) δ 7.83 (dd, 1H, *J* = 8.5, 5.5 Hz, ArCH), 7.31 (dd, 1H, *J* = 9.9, 3.1 Hz, ArCH), 6.85 (td, 1H, *J* = 8.5, 3.1 Hz, ArCH), 4.55 (s, 2H, CH_2); ^{13}C-NMR (100 MHz, MeOD) δ 164.9 (d, *J* = 246.7 Hz, ArCF), 147.5 (d, *J* = 6.9 Hz, Ar<u>C</u>CH$_2$),

141.4 (d, J = 7.8 Hz, ArCH), 116.8 (d, J = 22.5 Hz, ArCH), 115.7 (d, J = 24.1 Hz, ArCH), 89.3 (ArCI), 68.9 (CH_2); $^{19}F\{^1H\}$-NMR (377 MHz, MeOD) δ -113.4; m.p. 106-108 ˚C (EtOAc/Petrol), lit. 107-108 ˚C.[8] ^1H-NMR and ^{13}C-NMR data is consistent with literature.[8]

5-Fluoro-2-iodobenzaldehyde S20

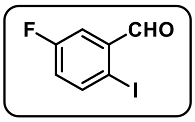

Dess Martin periodinane (0.64 g, 1.51 mmol) was added in one portion to a stirred solution of (5-fluoro-2-iodophenyl)methanol **S19** (0.57 g, 1.26 mmol) in CH_2Cl_2 (15 mL). After 18 h the reaction was quenched with $Na_2S_2O_3$ (1M aqueous solution, 15 mL) and extracted with CH_2Cl_2 (3 x 15 mL). The combined organic layers were washed with $NaHCO_3$ (saturated aqueous solution, 30 mL) dried over $MgSO_4$, filtered and concentrated *in vacuo*. The crude product was purified by SiO_2 flash chromatography, eluting with 0-5% EtOAc/Petrol, to yield the product as a yellow solid (0.36 g, 64%). ^1H-NMR (400 MHz, $CDCl_3$) δ 10.02 (d, 1H, J = 3.2 Hz, CHO), 7.93 (dd, 1H, J = 8.5, 5.0 Hz, ArCH), 7.61 (dd, 1H, J = 8.5, 3.2 Hz, ArCH), 7.08 (td, 1H , J = 8.5, 3.2 Hz, ArCH); ^{13}C-NMR (100 MHz, $CDCl_3$) δ 194.6 (d, J = 1.4 Hz, CO), 163.2 (d, J = 250.8 Hz, ArCF), 141.9 (d, J = 6.8 Hz, ArCH), 136.6 (d, J = 6.0 Hz, ArC), 123.0 (d, J = 22.1 Hz, ArCH), 116.9 (d, J = 23.3 Hz, ArCH), 93.3 (ArCI); $^{19}F\{^1H\}$-NMR (377 MHz, MeOD) δ -111.73; m.p. 93-95 ˚C (EtOAc/Petrol).

4-Fluoro-1-iodo-2-(methoxyvinyl)benzene S21

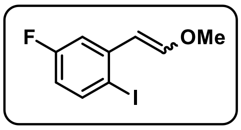

Potassium tert-butoxide (0.33 g, 2.9 mmol) was added portion wise to a stirred solution of (methoxymethyl)triphenylphosphonium chloride (1.04 g, 1.32 mmol) in THF (6 mL) at 0 ˚C. The reaction mixture turned bright orange upon this addition. After 15 min, 5-fluoro-2-iodobenzaldehyde **S20** (0.33 g, 1.32 mmol) in THF (3 mL) was added dropwise over 10 min to the reaction mixture at 0 ˚C. Upon this addition the reaction mixture turned pale yellow and was warmed to RT. After 21 h the reaction was quenched with H_2O and extracted with Et_2O (3 x 10 mL). The combined organic layers were washed with brine (20 mL), dried over $MgSO_4$, filtered and concentrated *in vacuo*. The crude product was purified by SiO_2 flash chromatography, eluting with 100% Petrol, to give a colourless oil as a 1:1 mixture of *E*:*Z* geometric isomers (0.30 g, 81%). ^1H-NMR (400 MHz, $CDCl_3$) δ 7.81-7.72 (m, 3H, ArCH), 7.03 (dd, 1H, J = 10.4, 2.8 Hz, ArCH), 6.93 (d, 1H, J = 12.9 Hz, ArCHCH), 6.65-6.59 (m, 2H, 2 x ArCH), 6.27 (d, 1H, J = 7.2 Hz, ArCHCH), 5.93 (d, 1H, J = 12.9 Hz, ArCHCH), 5.44 (d, 1H, J = 7.2 Hz, ArCHCH), 3.81 (s, 3H, OCH_3), 3.75 (s, 3H, OCH_3); ^{13}C-NMR (100 MHz, $CDCl_3$) δ 163.2 (d, J = 247.4 Hz, ArCF), 162.8 (d, J = 248.7 Hz, ArCF), 151.4, (ArCHCH) 150.1 (ArCHCH), 141.6 (d, J = 8.5 Hz, ArC), 140.4 (d, J = 8.4 Hz, ArCH), 139.9 (d, J = 7.8 Hz, ArCH), 116.4 (d, J = 23.2 Hz, ArCH), 114.8 (ArCH), 114.6 (ArCH) 111.8 (d, J = 24.0 Hz, ArCH), 108.9 (d, J = 2.5 Hz, ArCHCH), 108.4 (d, J = 2.5 Hz, ArCHCH), 93.9 (ArCI), 92.3 (ArCI), 61.0 (OCH_3), 56.8 (OCH_3); $^{19}F\{^1H\}$-NMR (377 MHz, $CDCl_3$) δ -114.24, -114.40.

2-(5-fluoro-2-iodophenyl)acetaldehyde S22

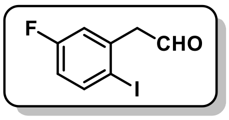

HCl (1M aqueous solution, 0.60 mL, 0.55 mmol) was added to a stirred solution of 4-fluoro-1-iodo-2-(methoxyvinyl)benzene **S21** (0.26 g, 0.94 mmol) in acetone (4 mL) and the reaction mixture was heated to 60 ˚C. After 2 hours the reaction was cooled to RT and concentrated in vacuo. The crude material was partitioned between CH_2Cl_2 (10 mL) and $NaHCO_3$ (saturated aqueous solution, 10 mL) and extracted with CH_2Cl_2 (2 x 10 mL). The combined organic phases were dried over $MgSO_4$, filtered and concentrated *in vacuo*. The crude product was purified by SiO_2 flash chromatography, eluting with 0-5% EtOAc/Petrol, to give a yellow oil (0.17 g, 68%). ^1H-NMR (400 MHz, $CDCl_3$) δ 9.78 (br t, 1H, J = 1.3 Hz, CHO), 7.82 (dd, 1H, J = 8.6, 5.6 Hz, ArCH), 6.99 (dd, 1H , J = 9.1, 2.5 Hz, ArCH), 6.79 (td, 1H, J = 8.6, 2.5 Hz, ArCH), 3.89 (d, 2H, J = 1.3 Hz, CH_2); ^{13}C-NMR (100 MHz, $CDCl_3$) δ 197.4 (CO), 163.0 (d, J = 249.3 Hz, ArCF), 140.8 (d, J = 8.0 Hz, ArCH), 138.3 (d, J = 8.0 Hz, ArC), 118.2 (d, J = 22.3 Hz, ArCH), 116.7 (d, J = 22.3 Hz, ArCH), 93.8 (d, J = 3.4 Hz, ArCI), 54.5 (CH_2); $^{19}F\{^1H\}$-NMR (377 MHz, $CDCl_3$) δ -113.4; $υ_{max}/cm^{-1}$ 1715.63, 1692.61, 1575.42, 1465.56, 1404.98, 1274.71, 1232.56, 1155.37, 1018.80, 811.63.

***(±)-tert*-Butyl (3a*S*,7a*R*)-1-(5-fluoro-2-iodophenethyl)-1,2,3,3a,4,5-hexahydro-7a*H*-indole-7a-carboxylate S23**

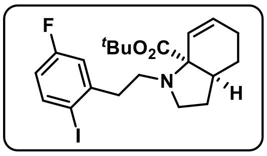

According to general procedure A, crude (±)-*tert*-butyl (3a*S*,7a*R*)-3-3a,4,5-tetrahydro-7a*H*-indole-7a-carboxylate **S7** (0.10 g, 0.45 mmol) and 2-(5-fluoro-2-iodophenyl)acetaldehyde **S22** (0.12 g, 0.45 mmol) were stirred with sodium triacetoxyborohydride (0.290 g, 1.35 mmol) in CH_2Cl_2 (2 mL). The crude product was purified by SiO_2 flash chromatography, 0-10% EtOAc/Petrol, to give a yellow oil (0.14 g, 67%). ^1H-NMR (400 MHz, $CDCl_3$) δ 7.72 (dd, 1H, J = 8.6, 5.8 Hz, ArCH), 7.00 (dd, 1H, J = 9.8, 2.9 Hz, ArCH), 6.65 (td, 1H, J = 8.6, 2.9 Hz, ArCH), 5.92 (dt, 1H, J = 10.3, 3.8 Hz, 6-CH), 5.72 (br d, 1H, J = 10.3 Hz, 7-CH), 3.04-2.99 (m, 1H, 1-CH), 2.95-2.83 (m, 4H, 1-CH, NCHH, NCH_2), 2.77-2.71 (m, 1H, NCHH), 2.56-2.49 (m, 1H, 3-CH), 2.13-1.92 (m, 3H, 2-CH and 5-CH_2), 1.74-1.66 (m, 1H, 4-CH), 1.60-1.54 (m, 2H, 2-CH and 4-CH), 1.45 (s, 9H, CH_3); ^{13}C-NMR (125 MHz, $CDCl_3$) δ 173.7 (CO), 163.0 (d, J = 247.7 Hz, ArCF), 145.7 (d, J = 7.1 Hz, ArC) 140.2 (d, J = 7.9 Hz, ArCH), 130.5 (6-CH), 124.4 (7-CH), 117.1 (d, J = 22.2 Hz, ArCH), 115.1 (d, J = 21.6 Hz, ArCH), 93.3 (d, J = 2.8 Hz, ArCI), 80.8 (C), 69.1 (CCH_3), 50.2 (1-CH), 50.0 (NCH_2), 40.6 (NCH_2CH_2), 40.4 (3-CH), 28.2 (CH_3), 27.4 (5-CH_2), 25.3 (4-CH_2), 22.3 (2-CH_2); $^{19}F\{^1H\}$-NMR (377 MHz, $CDCl_3$) δ -114.82; $υ_{max}/cm^{-1}$ 2928.70, 1716.73, 1461.25, 1366.94, 1250.93, 1230.81, 1155.59, 1048.07, 1014.84, 806.63; *m/z* HRMS (ESI^+) found $[M + H]^+$ 472.1130, $[C_{21}H_{28}FINO_2]^+$ requires 472.1149.

Synthesis of Aryl Triflate and Bromide

***(±)-tert*-Butyl (3a*S*,7a*R*)-1-(2-(((trifluoromethyl)sulfonyl)oxy)benzyl)-1,2,3,3a,4,5-hexahydro-7aH-indole-7a-carboxylate S24**

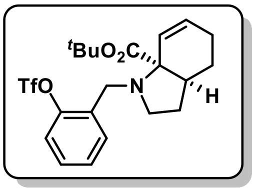

To a solution of imine **S7** (crude from rearrangement, 90 mg, ~0.41 mmol) and 2-formylphenyl triflate (126 mg, 0.50 mmol) in dry DCM (4 mL) at 0 °C under nitrogen was added $NaBH(OAc)_3$ (270 mg, 1.27 mmol) in one portion. Allowed to warm to rt, stirred for 20 h, quenched with sat. aq. NaHCO3 (10 mL), stirred for 15 min and extracted with DCM (2 × 10 mL). Drying ($MgSO_4$) and evaporation gave a yellow oil. Purification by silica gel chromatography (EtOAc/petrol, 5:95 to 3:7 as eluent) afforded the title compound (115 mg, 61% over two steps) as a clear oil. ν_{max} /cm^{-1} (film) 2931, 1718, 1453, 1420, 1368 and 1247; δ_H (500 MHz, $CDCl_3$) 1.51 (9H, s, *Me*$_3$C), 1.52 – 1.70 (2H, m, NCH_2CH*H* and =CH-CH_2-CH*H*), 1.77 (1H, ddt, J 13.0, 6.6, 5.0 Hz, NCH_2C*H*H), 2.01 – 2.16 (3H, m, allyic CH_2 and =CH-CH_2-C*H*H), 2.60 (1H, tt, J 9.0, 4.6 Hz, C*H*), 2.68 – 2.75 (2H, m, NC*H*$_2$), 4.02 (1H, d, J 15.1 Hz, NCH*H*Ph), 3.90 (1H, d, J 15.1 Hz, NC*H*HPh), 5.84 (1H, dt, J 10.2, 2.1 Hz, C*H*=CH-CH_2), 6.04 (1H, dt, J = 10.2, 3.9 Hz, =C*H*-CH_2), 7.23 (1H, dd, J 8.2, 1.2 Hz, CHAr), 7.32 – 7.28 (1H, td, J 8.1, 1.8, CHAr), 7.37 (1H, td, J 7.5, 1.3 Hz, CHAr) and 7.75 (1H, dd, J 7.7, 1.8 HzCHAr); δ_C (126 MHz, $CDCl_3$) 22.9 (allylic CH_2), 25.9 (NCH_2CH_2), 27.8 (homoalllyic) 28.1 (C*Me*$_3$), 40.3 (CH), 47.4 (*C*H$_2$Ph), 50.0 (NCH_2), 69.1 (Cq-CO2tBu), 81.2 Me$_3$*C*O), 118.6 (q, J = 320), 120.8 (CHAr), 124.5 (*C*H=CH-CH_2), 128.1 (CHAr), 128.4 (CHAr), 131.0 (CHAr), 131.2 (=*C*H-CH_2), 133.8 (Cq), 147.7 (Cq) and 173.6 (*C*O$_2$tBu); HRMS (ESI$^+$) 462.1540 ($C_{21}H_{27}F_3NO_5S$, $[M+H]^+$, requires 462.1557).

***(±)-tert*-Butyl (3a*S*,7a*R*)-1-(5-methoxy-2-(((trifluoromethyl)sulfonyl)oxy)benzyl)-1,2,3,3a,4,5-hexahydro-7a*H*-indole-7a-carboxylate S25**

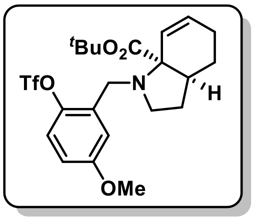

To a solution of imine **S7** (crude from rearrangement, 90 mg, ~0.41 mmol) and 2-formyl-4-methoxphenyl triflate (141 mg, 0.50 mmol) in dry DCM (4 mL) at 0 °C under nitrogen was added $NaBH(OAc)_3$ (270 mg, 1.27 mmol) in one portion. Allowed to warm to rt, stirred for 20 h, quenched with sat. aq. $NaHCO_3$ (10 mL), stirred for 15 min and extracted with DCM (2 × 10 mL). Drying ($MgSO_4$) and evaporation gave a yellow oil. Purification by silica gel chromatography (EtOAc/petrol, 5:95 to 1:9 as eluent) afforded the title compound (105 mg, 52% over two steps) as a clear oil. ν_{max} /cm^{-1} (film) 2973, 1718, 1488, 1418, 1368 and 1248; δ_H (500 MHz, $CDCl_3$) 1.51 (9H, s, *Me*$_3$C), 1.52 – 1.70 (2H, m, NCH_2CH*H* and =CH-CH_2-CH*H*), 1.77 (1H, dq, J 16.2, 5.3 Hz, NCH_2C*H*H), 2.01 – 2.16 (3H, m, allyic CH_2 and =CH-CH_2-C*H*H), 2.60 (1H, tt, J 9.0, 4.6 Hz, C*H*), 2.70 – 2.78 (2H, m, NC*H*$_2$), 3.82 (3H, s OMe), 3.87 (1H, d, J 15.4 Hz, NC*H*HPh), 3.98 (1H, d, J 15.4 Hz, NCH*H*Ph), 5.83 (1H, dt, J 10.2, 2.1 Hz, C*H*=CH-CH_2), 6.04 (1H, dt, J = 10.2, 3.9 Hz, =C*H*-CH_2), 6.78 (1H, dd, J 9.0, 3.3 Hz, CHAr), 7.14 (1H, d, J 8.9, CHAr) and 7.28 (1H, d, J 3.3 Hz, CHAr); δ_C (126 MHz, $CDCl_3$) 23.0 (allylic CH_2), 26.1 (NCH_2*C*H$_2$), 27.9 (homoalllyic), 28.1 (C*Me*$_3$), 40.3 (CH), 47.5 (*C*H$_2$Ar), 50.0 (NCH_2), 55.6 (OMe), 69.0 (C_q-$CO_2{}^tBu$), 81.2 (Me$_3$*C*O), 112.9 (CH_{Ar}), 115.5 (CH_{Ar}), 118.5 (q, J = 321 Hz), 121.8 (CH_{Ar}), 124.6 (*C*H=CH-CH_2), 131.2 (=*C*H-CH_2), 135.4 (C_{qAr}), 141.2 (C_{qAr}), 159.1 (C_{qAr}) and 173.6 ($CO_2{}^tBu$); HRMS (ESI$^+$) 492.1644 ($C_{22}H_{29}F_3NO_6S$, $[M+H]^+$, requires 492.1662).

(2-Bromo-4,5-dimethoxyphenyl)ethanol S26

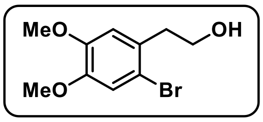

N-Bromo-succinimide (2.15 g, 12.1 mmol) was added in one portion to a stirred solution of of 2-(3,4-dimethoxyphenyl)ethanol (2.00 g, 11.0 mmol) in $CHCl_3$ (70 mL) and heated to reflux. After 5.5 h the reaction mixture was cooled to RT, quenched with $Na_2S_2O_3$ (1M aqueous solution, 80 mL), dried over $MgSO_4$, filtered and concentrated *in vacuo*. The crude product was purified by SiO_2 flash chromatography, eluting with 10-50% EtOAc/Petrol, to give the product as an orange oil (2.18 g, 76%). ^{1}H-NMR (400 MHz, $CDCl_3$) δ 7.02 (s, 1H, ArCH), 6.78 (s, 1H, ArCH), 3.87-3.84 (m, 8H, C<u>H</u>$_2$OH and 2 x OCH_3), 2.95 (t, 2H, *J* = 6.7 Hz, CH_2); ^{13}C-NMR (100 MHz, $CDCl_3$) δ 148.4 (ArC), 148.3 (ArC), 129.7 (ArC), 115.7 (ArCH), 114.4 (ArC), 113.8 (ArCH), 62.3 (OCH_2), 56.1 (OCH_3), 56.1 (OCH_3), 39.0 (CH_2). ^{1}H-NMR and ^{13}C-NMR data is consistent with literature.[9]

2-(2-Bromo-4,5-dimethoxyphenyl)acetaldehyde S27

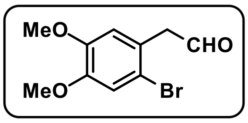

Dess Martin periodinane (3.84 g, 9.05 mmol) was added in one portion to a stirred solution of (2-bromo-4,5-dimethoxphenyl)ethanol **S26** (2.15 g, 8.23 mmol) in CH_2Cl_2 (35 mL). After 2 h the reaction was quenched with $Na_2S_2O_3$ (1M aqueous solution, 20 mL) and extracted with CH_2Cl_2 (3 x 15 mL). The combined organic layers were washed with $NaHCO_3$ (saturated aqueous solution, 20 mL) dried over $MgSO_4$, filtered and concentrated *in vacuo*. The crude was purified by SiO_2 flash chromatography, eluting with 0-20% EtOAc/Petrol, to yield the product as a yellow oil (1.26 g, 80%). ^{1}H-NMR (400 MHz, $CDCl_3$) δ 9.73 (br s, 1H, CHO), 7.07 (s, 1H, ArCH), 6.70 (s, 1H, ArCH), 3.87 (s, 3H, OCH_3), 3.85 (s, 3H, OCH_3), 3.78 (d, 2H, *J* = 1.6 Hz, CH_2); ^{13}C-NMR (100 MHz, $CDCl_3$) δ 198.5 (CO), 149.1 (ArC), 148.7 (ArC), 124.2 (ArC), 115.7 (ArCH), 115.0 (ArC), 114.0 (ArCH), 56.2 (OCH_3), 56.1 (OCH_3), 50.1 (CH_2); m.p. 59-62 ˚C (EtOAc/Petrol), lit. 64-65 ˚C.[10] ^{1}H-NMR and ^{13}C-NMR data is consistent with literature.[11]

(±)-*tert*-Butyl (3aS,7a*R*)-1-(2-bromo-4,5-dimethoxyphenethyl)-1,2,3,3a,4,5-hexahydro-7a*H*-indole-7a-carboxylate S28

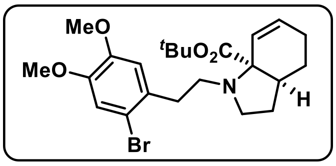

According to general procedure A, crude (±)-*tert*-butyl (3a*S*,7a*R*)-3-3a,4,5-tetrahydro-7a*H*-indole-7a-carboxylate **S5** (0.25 g, 0.95 mmol) and 2-(2-bromo-4,5-dimethoxyphenl)acetaldehyde **S27** (0.25 g, 0.95 mmol) were stirred with sodium triacetoxyborohydride (0.600 g, 2.85 mmol) in CH_2Cl_2 (4 mL). The crude was purified by SiO_2 flash chromatography, 0-30% EtOAc/Petrol, to give a yellow oil (0.39 g, 89%). ^{1}H-NMR (400 MHz, $CDCl_3$) δ 6.98 (s, 1H, ArCH), 6.78 (s, 1H, ArCH), 5.93 (tt, 1H, *J* = 10.3, 3.7 Hz, 6-CH), 5.75 (br d, 1H, *J* = 10.3 Hz, 7-CH), 3.84 (s, 3H, OCH_3), 3.06-2.99 (m, 1H, 1-CH), 2.94- 2.78 (m, 4H, 1-CH, NCH$\underline{H}$ and $NCH_2C\underline{H}_2$), 2.78-2.67 (m, 1H, NC$\underline{H}$H), 2.58-2.50 (m, 1H, 3-CH), 2.13-1.92 (m, 3H, 4-CH_2 and 5-CH), 1.75-1.68 (m, 1H, 2-CH), 1.61-1.53 (m, 2H, 2-CH and 5-CH), 1.45 (s, 9H, CH_3); ^{13}C-NMR (125 MHz, $CDCl_3$) δ 173.6 (CO), 148.2 (ArC), 147.8 (ArC), 131.9 (ArC), 130.4 (6-CH), 124.4 (7-CH) 115.3 (ArCH), 114.0 (ArC), 113.4 (ArCH), 80.8 (C), 69.0 ($\underline{C}CH_3$), 56.1 (OCH_3), 56.0 (NCH_2), 50.3 (1-CH), 40.3 (3-CH), 35.7 ($NCH_2\underline{C}H_2$), 28.1 (5-CH_2), 27.3 (CH_3), 25.2 (2-CH), 22.1 (4-CH_2); υ_{max}/cm^{-1} 1715.34, 1505.62, 1455.53, 1439.62, 1382.35, 1366.82, 1256.40, 1219.67, 1161.88, 1033.21; *m/z* HRMS (ESI$^+$) found [M + H]$^+$ 466.1574 and 468.1555, $[C_{23}H_{33}{}^{79}BrNO_4]^+$ requires 466.1593 and $[C_{23}H_{33}{}^{81}BrNO_4]^+$ requires 468.1572.

Synthesis of Additives

1-Iodo-4,5-dimethoxy-2-methybenzene S1

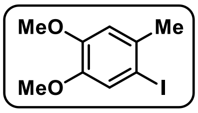

To a stirred solution of 1,2-dimethoxy-3-methylbenzene (1.50 mL, 10.4 mmol) and NIS (2.50 g, 11.1 mmol) in dry MeCN (40 mL) at rt under nitrogen was added TFA (0.080 mL, 1.05 mmol) dropwise. The reaction was stirred at rt for 7 h, quenched by the addition of 2M NaOH (10 mL) and 1M sodium thiosulphate (20 mL), stirredfor 10 min and extracted with EtOAc (30 mL). The organic phase was washed with brine (30 mL), dried ($MgSO_4$) and evaporated to give a brown oil. Purification by silica gel chromatography (EtOAc/petrol, 5:95 to 1:9 as eluent) afforded the title compound (2.03 g, 70%) as a light yellow oil.^{1}H-NMR and ^{13}C-NMR are conistant with literature.[12]

***N,N*-diisopropylethylamine hydroiodide**

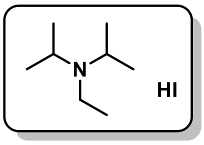

Hydriodic acid (56% aqueous solution, 0.77 mL, 5.7 mmol) was added dropwise to a stirred solution of *N*,*N*-diisopropylethylamine (1.0 mL, 5.7 mmol) in CH_2Cl_2 (10 mL) at 0 ˚C. The reaction mixture was warmed to RT and stirred for 10 min, extracted with CH_2Cl_2 (4 x 10 mL) and concentrated *in vacuo*. The crude material was recrystallised from Et_2O to give a pale-yellow solid (1.26 g, 85%). ^{1}H-NMR (400 MHz, $CDCl_3$) δ 9.35 (br s, 1H, HI), 3.82-3.76 (m, 2H, NCH), 3.20 (q, 2H, *J* = 6.7 Hz, ($C\underline{H}_2CH_3$), 1.66-1.61 (m, 9H, CH3), 1.51-1.50 (d, 6H, J = 6.7 Hz, CH_3); ^{13}C-NMR (100 MHz, $CDCl_3$) δ 54.6 (NCH), 42.7 (NCH_2), 18.6 (CH_3), 17.4 (CH_3), 12.1 (CH_3). ^{1}H-NMR and ^{13}C-NMR data is consistent with literature.[13]

***N,N*-diisopropylethylamine hydrochloride**

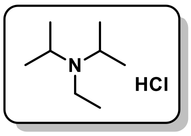

Hydrochloric acid (2M sol in Et_2O, 2.87 mL, 5.74 mmol) added dropwise to a stirred solution of of *N*,*N*-diisopropylethylamine (1 mL, 5.74 mmol) in CH_2Cl_2 (15 mL) and stirred for 15 min. The reaction mixture was concentrated *in vacuo* and the solid washed with Et_2O to give a colourless solid (0.91 g, 96%). ^{1}H-NMR 11.19 (br s, 1H, HCl), 3.64 (qn, 2H, *J* = 6.4 Hz, 2 x NCH), 3.07 (q, 2H, *J* = 7.4 Hz, NCH_2), 1.55-1.42 (m, 15H, 5 x CH_3); ^{13}C-NMR (100 MHz, $CDCl_3$) δ 53.6 (NCH), 41.9 (NCH_2), 15.6 (CH_3), 17.3 (CH_3), 12.0 (CH_3).

Synthesis of Substrates

2-(3,4-Dimethoxyphenyl)acetaldehyde S29

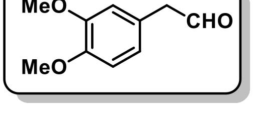

To a stirred solution of substrate (1.00 g, 5.50 mmol) in dry DCM (22 mL) at rt under nitrogen was added DMP (2.8 g, 6.6 mmol) in one portion – exotherm. The mixture was stirred for 4 h, quenched with 1 M sodium thiosulphate (15 mL), the mixture stirred for 10 minutes, diluted with water (10 mL) and extracted with DCM (2 × 25 mL). Drying ($MgSO_4$) and evaporation gave a white solid. Purification by silica gel chromatography (EtOAc/petrol, 1:9 to 1:4 as eluent) gave a white solid which was redissolved in Et_2O (30 mL) and washed with sat. aq. $NaHCO_3$ (2 × 15 mL). Drying ($MgSO_4$) and evaporation afforded the title compound as a clear oil (636 mg, 64%). ^{1}H-NMR and ^{13}C-NMR data is consistent with literature.[14]

***(±)-tert*-Butyl (3a*S*,7a*R*)-1-(3,4-dimethoxyphenethyl)-1,2,3,3a,4,5-hexahydro-7a*H*-indole-7a-carboxylate 16a**

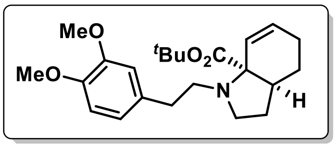

To a stirred solution of substrate **S7** (120 mg, 0.54 mmol) and 2-(3,4-dimethoxyphenyl)acetaldehyde (100 mg, 0.56 mmol) in anhydrous DCM (5 mL) at 0 °C under nitrogen was added $NaBH(OAc)_3$ (360 mg, 1.7 mmol) in one portion. The reaction was warmed to rt and stirred overnight. After 13 h the reaction was quenched by the addition of sat. aq. $NaHCO_3$ (10 mL), stirred for 15 min and extracted with DCM (2 × 10 mL). Drying ($MgSO_4$) and evaporation gave a yellow oil. Purification by silica gel chromatography (EtOAc/petrol, 5:95 to 1:4 as eluent) afforded the title compound (158 mg, 77% over two steps) as a clear oil. ν_{max}/cm^{-1} (film) 2972, 1716, 1515 and 1260; δ_H (500 MHz, $CDCl_3$) 1.48 (9H, s, Me_3C), 1.61 (2H, m, NCH_2CH*H* and homoallylic CH*H*), 1.70 – 1.81 (1H, m, homoallyllic C*H*H), 1.94 – 2.15 (3H, m, NCH_2C*H*H and allylic C*H*$_2$), 2.57 (1H, m, C*H*), 2.68 – 2.81 (4H, m, NCH*H*, NCH*H* and benzylic CH_2), 2.90 – 3.08 (2H, m, NC*H*H and NC*H*H), 3.88 (3H, s, OMe), 3.89 (3H, s, OMe), 5.73 – 5.80 (1H, d, J 10.3, C*H*=CH-CH_2), 5.97 (1H, d, J 10.2, =C*H*-CH_2), 6.72 – 6.82 (3H, m, 3 × CH_{Ar}); δ_C (126 MHz, $CDCl_3$) 22.1 (allylic *C*H_2), 25.1 (homoallylic CH_2), 27.3 (NCH_2CH_2), 28.2 (*Me*$_3$C), 35.8 (benzylic CH_2), 40.4 (CH), 50.5 (NCH_2), 52.4 (NCH_2), 55.8 (OMe), 55.9 (OMe), 69.1 (C_q-$CO_2{}^tBu$), 80.8 (Me_3*C*O), 111.2 (CH_{Ar}), 112.1 (CH_{Ar}), 120.5 (CH_{Ar}), 124.5 (*C*H=CH-CH), 130.4 (=*C*H-CH), 133.5 (C_{qAr}), 147.2 (C_{qAr}), 148.7 (C_{qAr}) and 173.7 (*C*$O_2{}^tBu$); HRMS (ESI^+) 388.2500 ($C_{23}H_{34}NO_4$, $[M+H]^+$, requires 388.2482).

(±)-*tert*-Butyl (3a*S*,7a*R*)-1-(4-methoxybenzyl)-1,2,3,3a,4,5-hexahydro-7a*H*-indole-7a-carboxylate 12 (**16e**)

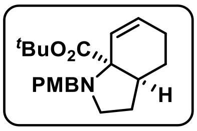

A stirred solution of aziridine **S6** (340 mg, 1.54 mmol) in toluene (15 mL) was heated to 100 °C under nitrogen. After 16 h the reaction was evaporated to give a brown oil which was redissolved in anhydrous DCM (12 mL) and cooled to 0 °C. Aldehyde (0.20 mL, 1.6 mmol) was added, followed by $NaBH(OAc)_3$ (1.08 g, 5.0 mmol) in one portion. The reaction was allowed to warm to rt, stirred for 13 h and quenched by the addition of sat. aq. $NaHCO_3$ (10 mL). The mixture was stirred for 15 min and extracted with DCM (2 × 8 mL). Drying ($MgSO_4$) and evaporation gave an orange oil which was purified by silica gel chromatography (EtOAc/petrol, 5:95 as eluent) to afford the title compound (380 mg, 72%) as a clear oil. ν_{max}/cm^{-1} (film) 2931, 1717, 1612, 1511, 1455, 1367 and 1244; δ_H (500 MHz, $CDCl_3$) 1.47 – 1.56 (10H, m, *Me*3C and NCH2CH*H*), 1.60 – 1.68 (1H, m, homoallylic CH*H*), 1.75 (1H, dddd, J 13.1, 7.7, 5.3, 4.3, homoallylic C*H*H), 1.96 – 2.07 (2H, m, NCH2C*H*H and allylic CH*H*), 2.11 (1H, dddd, J 15.2, 7.6, 3.8, 2.1, allylic C*H*H), 2.54 – 2.65 (2H, m, NCH*H*CH2 and C*H*), 2.71 (1H, td, J 9.0, 4.8, NC*H*HCH2), 3.61 (1H, d, J 13.1, NCH*H*Ar), 3.81 (3H, s, OMe), 3.83 (1H, d, J 13.1, NC*H*HAr), 5.87 (1H, dt, J 10.7, 1.9, C*H*=CH-CH2), 6.03 (1H, dt, J 10.3, 3.9, =C*H*-CH2), 6.83 – 6.88 (2H, m, 2 × CH_{Ar}) and 7.25 – 7.31 (2H, m, 2 × CH_{Ar}); δ_C (126 MHz, $CDCl_3$) 22.4 (allylic CH_2), 25.4 (homoallylic CH_2), 27.3 (NCH_2CH_2), 28.3 (*Me*$_3$C), 40.4 (CH), 49.8 (N*C*H_2CH_2), 53.4 (N*C*H_2Ar), 55.3 (OMe), 69.0 (*C*$_q$-$CO_2{}^tBu$), 80.8 (Me_3*C*O), 113.5 (2 × CH_{Ar}), 124.7 (*C*H=CH-CH_2), 129.4 (2 × CH_{Ar}), 130.7 (=*C*H-CH_2), 132.8 (C_{qAr}), 158.4 (C_{qAr}) and 173.8 (*C*$O_2{}^tBu$); HRMS (ESI^+) 344.2218 ($C_{21}H_{30}NO_3$, $[M+H]^+$, requires 344.2220).

(±)-*tert*-Butyl (3a*R*,7a*R*)-1-pentyl-)-1,2,3,3a,4,5-hexahydro-7a*H*-indole-7a-carboxylate 16b

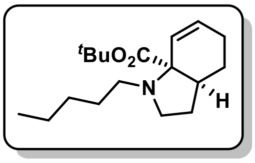

According to general procedure A, crude (±)-*tert*-butyl (3a*S*,7a*R*)-3-3a,4,5-tetrahydro-7a*H*-indole-7a-carboxylate **S7** (0.15 g, 0.68 mmol) and valeraldehyde (0.070 mL, 0.68 mmol) was stirred with sodium triacetoxyborohydride (0.43 g, 2.04 mmol) in CH_2Cl_2 (3 mL). The crude material was purified by SiO_2 flash chromatography, 0-10% EtOAc/petrol to give the product as a yellow oil (0.11 g, 55%). ^{1}H-NMR (400 MHz, $CDCl_3$) δ 5.95 (dt, 1H, *J* = 10.3, 3.8 Hz, 6-CH), 5.78 (br d, 1H, *J* = 10.3 Hz, 7-CH), 2.89 (td, 1H, *J* = 8.9, 4.8 Hz, 1-CH), 2.69-2.59 (m, 2H, 1-CH and NC$\underline{H}$H), 2.57-2.50 (m, 1H, 3-CH), 2.48-2.41 (m, 1H, NCH$\underline{H}$), 2.08-2.01 (m, 2H, 2-CH and 5-CH), 1.99-1.91 (m, 1H, 2-CH), 1.75-1.67 (m, 1H, 4-CH), 1.61-1.53 (m, 4H, 4-CH, 5-CH and CH_2), 1.46 (s, 9H, 3 x CH_3), 1.34-1.25 (m, 4H, CH_2 and CH_2), 0.88 (t, 3H, *J* = 7.0 Hz, CH_3); ^{13}C-NMR (100 MHz, $CDCl_3$) δ 173.8 (CO), 130.0 (6-CH), 124.8 (7-CH), 80.6 (C(CH_3)), 69.0 (C), 50.4 (1-CH_2), 50.3 (NCH_2), 40.5 (3-CH), 29.8 (CH_2), 29.1 (CH_2), 28.2 (3 x CH_3), 27.3 (5-CH_2), 25.2 (4-CH_2), 22.6 (CH_2), 22.1 (2-CH_2), 14.1 (CH_3); υ_{max}/cm^{-1} 2957.28, 2930.28, 2859.84, 1718.28, 1456.16, 1392.08, 1367.30, 1250.14, 1156.77, 1048.29; *m/z* HRMS (ESI^+) found $[M + H]^+$ 294.2428, $[C_{18}H_{32}NO_2]^+$ requires 294.2433.

(±)-*tert*-Butyl (3a*R*,7a*R*)-1-(3,3,3-trifluoropropyl)-1,2,3,3a,4,5-hexahydro-7a*H*-indole-7a-carboxylate 16c

According to general procedure B, crude (±)-*tert*-butyl (3a*S*,7a*R*)-3-3a,4,5-tetrahydro-7a*H*-indole-7a-carboxylate **S7** (0.15 g, 0.68 mmol) and 3,3,3-trifluoropropanal (0.060 mL, 0.68 mmol) was stirred with sodium triacetoxyborohydride (0.43 g, 2.04 mmol) in CH_2Cl_2 (3 mL). The crude material was purified by SiO_2 flash chromatography, 0-3% EtOAc/petrol to give the product as a yellow oil (0.17 g, 75%). ^{1}H-NMR (400 MHz, $CDCl_3$) δ 5.99 (dt, 1H, *J* = 10.7, 4.1 Hz, 6-CH), 5.72 (dt, 1H, *J* = 10.1, 2.0 Hz, 7-CH), 3.03-2.96 (m, 1H, NC$\underline{H}$HCH_2), 2.91 (td, 1H, *J* = 8.6, 4.6 Hz, 1-CH), 2.78-2.66 (m, 2H, 1-CH and NCH$\underline{H}$), 2.57-2.50 (m, 1H, 3-CH), 2.31-2.24 (m, 2H, CH_2CF_3), 2.09-1.98 (m, 3H, 2-CH and 5-CH_2), 1.77-1.69 (m, 1H, 4-CH), 1.62-1.52 (m, 2H, 2-CH and 4-CH), 1.46 (s, 9H, CH_3); ^{13}C-NMR (100 MHz, $CDCl_3$) δ 73.3 (CO), 131.2 (6-CH), 126. (q, *J* = 278.1 Hz, CF_3), 123.8 (7-CH), 81.1 (C(CH_3)), 69.0 (C), 50.1 (1-CH_2), 43.0 (N$\underline{C}H_2CH_2$), 34.2 (q, *J* = 27.2 Hz, $\underline{C}H_2CF_3$), 28.1 (CH_3), 27.1 (2-CH_2), 25.0 (4-CH_2), 22.2 (5-CH_2); ^{19}F{^{1}H}-NMR (377 MHz, $CDCl_3$) δ -65.43; υ_{max}/cm^{-1} 1718.14, 1393.20, 1368.37, 1342.08, 1250.14, 1155.53, 1128.14, 1046.63, 1007.18, 847.24; *m/z* HRMS (ESI^+) found $[M + H]^+$ 320.1820, $[C_{16}H_{25}F_3NO_2]^+$ requires 320.1837.

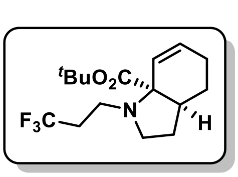

(±)-*tert*-Butyl (3a*S*,7a*R*)-1-(3,4-dimethoxybenzy)-1,2,3,3a,4,5-hexahydro-7a*H*-indole-7a-carboxylate 16d

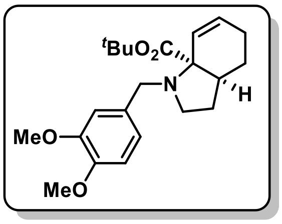

A stirred solution of aziridine **S6** (320 mg, 1.45 mmol) in toluene (15 mL) was heated to 100 °C under nitrogen. After 16 h the reaction was evaporated to give a brown oil which was redissolved in anhydrous DCM (12 mL) and cooled to 0 °C. 3,4-dimethoxybenzaldehyde (262 mg, 1.58 mmol) was added, followed by $NaBH(OAc)_3$ (1.08 g, 5.0 mmol) in one portion. The reaction was allowed to warm to rt, stirred for 14 h and quenched by the addition of sat. aq. $NaHCO_3$ (10 mL). The mixture was stirred for 15 min and extracted with DCM (2 × 8 mL). Drying ($MgSO_4$) and evaporation gave an orange oil which was purified by silica gel chromatography (EtOAc/petrol, 1:9 to 1:4 as eluent) to afford the title compound (442 mg, 82%) as a clear oil. ν_{max} /cm^{-1} (film) 2931, 1716, 1592, 1512, 1367 and 1254; δ_H (500 MHz, $CDCl_3$) 1.52 (10H, s, *Me*3C and NCH_2CH*H*), 1.60 – 1.68 (1H, m, homooallylic CH*H*), 1.71 – 1.79 (1H, m, homoallylic C*H*H), 1.97 – 2.08 (1H, m, allylic C*H*H and NCH_2C*H*H), 2.11 (1H, dddd, J 15.0, 7.5, 3.8, 2.0, allylic C*H*H), 2.57 – 2.66 (2H, m, NCH*H*CH_2 and C*H*), 2.73 (1H, td, J 8.9, 4.8, NC*H*HCH_2), 3.64 (1H, d, J 13.2, NCH*H*Ar), 3.83 (H, d, J 13.3, NC*H*HAr), 3.88 (3H, s, OMe), 3.89 (3H, s, OMe), 5.87 (1H, dt, J 10.3, 2.1, C*H*=CH-CH_2), 6.04 (1H, dt, J 10.2, 3.8-, =C*H*-CH_2), 6.81 (1H, d, J 8.1, CH_{Ar}), 6.89 (1H, dd, J 8.1, 1.9, CH_{Ar}) and 6.95 (1H, d, J 1.9, CH_{Ar}); δ_C (126 MHz, $CDCl_3$) 22.5 (allylic CH_2), 25.5 (homoallylic CH_2), 27.4 (NCH_2CH_2), 28.3 (*Me*$_3$C), 40.4 (CH), 49.9 (N*C*H$_2$CH$_2$), 53.8 (N*C*H$_2$Ar), 55.8 (OMe), 55.9 (OMe), 69.0 (C_q-$CO_2{}^tBu$), 80.7 (Me_3CO), 110.8 (CH_{Ar}), 111.6 (CH_{Ar}), 120.2 (CH_{Ar}), 124.7 (*C*H=CH-CH_2), 130.7 (=*C*H-CH_2), 133.4 (C_{qAr}), 147.8 (C_{qAr}), 148.8 (C_{qAr}) and 173.8 (*C*O$_2{}^t$Bu); HRMS (ESI$^+$) 374.2320 ($C_{22}H_{31}NO_4$, $[M+H]^+$, requires 374.2326).

(±)-*tert*-Butyl (3a*S*,7a*R*)-1-ethyl-1,2,3,3a,4,5-hexahydro-7a*H*-indole-7a-carboxylate 16f

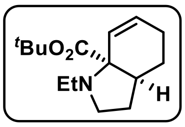

A stirred solution of aziridine **S6** (110 mg, 0.50 mmol) in toluene (5 mL) was heated to 100 °C under nitrogen. After 16 h the reaction was evaporated to give a brown oil which was redissolved in anhydrous DCM (5 mL) and cooled to 0 °C. Acetaldehyde (0.050 mL, 0.89 mmol) was added, followed by $NaBH(OAc)_3$ (360 mg, 1.7 mmol) in one portion. The reaction was allowed to warm to rt, stirred for 13 h and quenched by the addition of sat. aq. $NaHCO_3$ (8 mL). The mixture was stirred for 10 min and extracted with DCM (2 × 8 mL). Drying ($MgSO_4$) and evaporation gave an orange oil which was purified by silica gel chromatography (EtOAc/petrol, 1:9 to 1:4 as eluent) to afford the title compound (88 mg, 70%) as a clear oil. ν_{max} /cm^{-1} (film) 2970, 1716, 1453, 1367, 1248 and 1155; δ_H (500 MHz, $CDCl_3$) 1.11 (3H, t, J 7.2, Me), 1.48 (9H, s, *Me*$_3$C), 1.53 – 1.64 (2H, m, homoallylic CH*H* and NCH_2CH*H*), 1.73 – 1.81 (1H, m, homoallylic C*H*H), 1.93 – 2.02 (1H, m, allylic C*H*H), 2.02 – 2.13 (2H, m, NCH_2C*H*H and allylic C*H*H), 2.50 – 2.61 (2H, m, NC*H*HMe and C*H*), 2.66 (1H, td, J 8.8, 6.1, NCH*H*CH_2), 2.74 (1H, dq, J 11.7, 7.3, NCH*H*Me), 2.95 (1H, td, J 8.9, 4.7, NC*H*HCH_2), 5.79 (1H, dt, J 10.6, 1.9, C*H*=CH-CH_2) and 5.98 (1H, dt, J 10.3, 3.9, =C*H*-CH_2); δ_C (126 MHz, $CDCl_3$) 14.7 (Me), 21.8 (allylic CH_2), 25.1 (homoallylic CH_2), 27.0 (NCH_2CH_2), 28.2 (*Me*$_3$C), 40.5 (CH), 44.2 (N*C*H$_2$Me), 50.0 (N*C*H$_2$CH$_2$), 68.9 (C_q-$CO_2{}^tBu$), 80.7 (Me_3CO), 124.6 (*C*H=CH-CH_2), 130.2 (=*C*H-CH_2) and 173.7 (*C*O$_2{}^t$Bu); HRMS (ESI$^+$) 252.1954 ($C_{15}H_{26}NO_2$, $[M+H]^+$, requires 252.1958).

(±)-*tert*-Butyl (3a*S*,7a*R*)-1-methyl-1,2,3,3a,4,5-hexahydro-7a*H*-indole-7a-carboxylate 16h

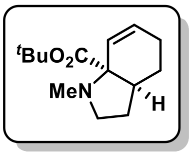

According to general procedure B, crude (±)-*tert*-butyl (3a*S*,7a*R*)-3-3a,4,5-tetrahydro-7a*H*-indole-7a-carboxylate **S7** (0.13 g, 0.59 mmol) and paraformaldehyde (53 mg, 1.77 mmol) was stirred with sodium triacetoxyborohydride (0.38 g, 1.77 mmol) in CH_2Cl_2 (3 mL). The crude material was purified by SiO_2 flash chromatography, 10-40% EtOAc/petrol to give the product as a yellow oil (49 mg, 35%). ^{1}H-NMR (400 MHz, $CDCl_3$) δ 6.01 (dt, 1H, *J* = 10.2, 4.0 Hz, 6-CH), 5.82 (dt, 1H, *J* = 10.2, 1.9 Hz, 7-CH), 2.90-2.84 (m, 1H, 1-CH), 2.77-2.71 (m, 1H, 1-CH), 2.61-2.54 (m, 1H, 3-CH), 2.41 (s, 3H, CH_3), 2.13-2.02 (m, 2H, 5-CH and 2-CH), 2.00-1.92 (m, 1H, 5-CH), 1.79-1.71 (m, 1H, 4-CH), 1.63-1.52 (m, 2H, 4-CH and 2-CH), 1.47 (s, 9H, CH_3); ^{13}C-NMR (100 MHz, $CDCl_3$) δ 173.1 (CO), 130.7 (6-CH), 124.0 (7-CH), 80.1 (C(CH_3)), 63.3 (C), 53.1 (1-CH_2), 40.4 (3-CH), 35.7 (CH_3), 28.2 (3 x CH_3), 27.5 (2-CH_2), 25.6 (4-CH_2), 22.1 (5-CH_2); υ_{max}/cm^{-1} 2972.60, 2929.89, 1716.06, 1454.24, 1367.27, 1250.75, 1156.17, 1049.48, 1033.30, 846.17; *m/z* HRMS (ESI$^+$) found $[M + H]^+$ 238.1795, $[C_{14}H_{24}NO_2]^+$ requires 238.1807.

(±)-*tert*-Butyl (3a*S*,7a*R*)-1-phenethyl)-1,2,3,3a,4,5-hexahydro-7a*H*-indole-7a-carboxylate 16g

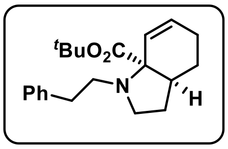

According to general procedure A, crude (±)-*tert*-butyl (3a*S*,7a*R*)-3-3a,4,5-tetrahydro-7a*H*-indole-7a-carboxylate **S7** (0.20 g, 0.90 mmol) and phenyl acetaldehyde (0.11 mL, 0.90 mmol) were stirred with sodium triacetoxyborohydride (0.57 g, 2.70 mmol) in CH_2Cl_2 (3 mL). The crude was purified by SiO_2 flash chromatography, 0-10% EtOAc/Petrol, to give a yellow oil (0.26 g, 90%). ^{1}H-NMR (400 MHz, $CDCl_3$) δ 7.30-7.16 (m, 5H, ArCH), 5.97-5.92 (m, 1H, 6-CH), 5.74 (br d, 1H, 7-CH), 3.03-2.94 (m, 2H, 1-CH and NC$\underline{H}$H), 2.82-2.69 (m, 4H, 1-CH, NCH$\underline{H}$ and $NCH_2C\underline{H_2}$), 2.56 (br s, 1H, 3-CH), 2.10-2.03 (m, 2H, 2-CH and 5-CH), 1.99-1.92 (m, 1H, 5-CH), 1.78-1.70 (m, 1H, 4-CH), 1.62-1.54 (m, 2H, 2-CH and 4-CH), 1.45 (s, 9H CH_3); ^{13}C-NMR (100 MHz, $CDCl_3$) δ 173.6 (CO), 140.8 (ArC), 130.4 (6-CH), 128.7 (ArCH), 128.2 (ArCH), 125.8 (ArCH), 124.4 (7-CH), 80.7 (C), 69.1 ($\underline{C}CH_3$), 52.3 (NCH_2), 50.5 (1-CH_2), 40.4 (3-CH), 36.3 ($NCH_2\underline{C}H_2$), 28.2 (CH_3), 27.2 (2-CH_2), 25.0 (4-CH_2), 22.0 (5-CH_2); υ_{max}/cm^{-1} 2928.95, 1715.98, 1453.88, 1366.60, 1248.87, 1154.74, 1047.32, 1029.85, 749.01, 698.51; *m/z* HRMS (ESI$^+$) found $[M + H]^+$ 328.2308, $[C_{21}H_{30}NO_2]^+$ requires 328.2277.

(±)-*tert*-Butyl (3a*S*,7a*R*)-1-(naphthalen-1-ylmethyl)-1,2,3,3a,4,5-hexahydro-7a*H*-indole-7a-carboxylate 16i

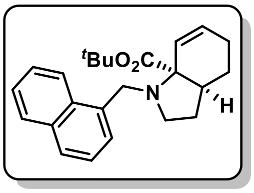

According to general procedure A, crude (±)-*tert*-butyl (3a*S*,7a*R*)-3-3a,4,5-tetrahydro-7a*H*-indole-7a-carboxylate **S7** (0.25 g, 1.13 mmol) and 1-naphthaldehyde (0.15 mL, 1.13 mmol) was stirred with sodium triacetoxyborohydride (0.72 g, 3.39 mmol) in CH_2Cl_2 (5 mL). The crude product was purified by SiO_2 flash chromatography, 0-5% EtOAc/petrol, which was contaminated with 1-naphthaldehyde. The crude material was left open to air for 3 weeks, dissolved in MeOH (10 mL) and $NaHSO_3$ (saturated aqueous solution, 10 mL) and extracted with CH_2Cl_2 (3 x 15 mL). The combined organic layers were dried over $MgSO_4$, filtered, concentrated *in vacuo* to give the pure product as a yellow oil (0.12 g, 29%). ^{1}H-NMR (400 MHz, $CDCl_3$) δ 8.38-8.36 (m, 1H, ArCH), 7.82-7.80 (m, 1H, ArCH), 7.72 (d, 1H, *J* = 8.2 Hz, ArCH), 7.47-7.43 (m, 3H, ArCH), 7.36 (t, 1H, *J* = 8.2 Hz, ArCH), 6.08 (br s, 2H, 6-CH and 7-CH), 4.22 (app q, 2H, NCH_2), 2.65-2.54 (m, 3H, 1-CH_2 and 3-CH), 2.12-1.95 (m, 3H, 2-CH and 5-CH_2), 1.69-1.58 (m, 2H, 4-CH_2), 1.52 (s, 9H, 3 x CH_3), 1.45-1.37 (m, 1H, 2-CH); ^{13}C-NMR (100 MHz, $CDCl_3$) δ; 173.6 (CO), 136.7 (ArC), 135.9 (ArC), 133.7 (ArCH), 132.4 (ArC), 131.3 (6-CH), 128.3 (ArCH), 127.3 (ArCH), 126.4 (ArCH), 125.4 (ArCH), 125.2 (ArCH), 124.8 (7-CH), 124.6 (ArCH), 81.0 (C(CH_3)), 69.2 (C), 52.0 (NCH_2), 49.7 (1-CH_2), 40.4 (3-CH), 28.3 (3 x CH_3), 27.6 (2-CH_2), 26.1 (4-CH_2), 23.2 (5-CH_2); υ_{max}/cm^{-1} 1716.38, 1692.87, 1367.01, 1249.00, 1154.57, 1118.22, 1048.93, 801.03, 792.19, 778.71; *m/z* HRMS (ESI$^+$) found [M + H]$^+$ 364.2269, $[C_{24}H_{30}NO_2]^+$ requires 364.227.

(±)-*tert*-Butyl (3a*S*,7a*R*)-1-(pyridine-4-ylmethyl)-1,2,3,3a,4,5-hexahydro-7a*H*-indole-7a-carboxylate 16j

According to general procedure A, crude (±)-*tert*-butyl (3a*S*,7a*R*)-3-3a,4,5-tetrahydro-7a*H*-indole-7a-carboxylate **S7** (0.12 g, 0.52 mmol) and 4-pyridinecarboxaldehyde (0.050 mL, 0.52 mmol) was stirred with sodium triacetoxyborohydride (0.330 g, 1.56 mmol) in CH_2Cl_2 (2 mL). The crude product was purified by SiO_2 flash chromatography, 0-10% EtOAc/petrol to give the product as a yellow oil (89 mg, 56%). ^{1}H-NMR (400 MHz, $CDCl_3$) δ 8.51 (d, 2H, *J* = 5.8 Hz, ArCH), 7.31 (d, 2H, *J* = 5.8 Hz, ArCH), 6.03 (dt, 1H, *J* = 10.4, 3.8 Hz, 6-CH), 5.79 (dt, 1H, *J* = 10.4, 1.8 Hz, 7-CH), 3.80 (d, 1H, *J* = 15.2 Hz, NCHH), 3.70 (d, 1H, *J* = 15.2 Hz, NCHH), 2.73-2.67 (m, 1H, 1-CH), 2.64-2.57 (m, 2H, 1-CH and 3-CH), 2.11-1.99 (m, 3H, 2-CH and 5-CH_2), 1.80-1.72 (m, 1H, 4-CH), 1.67-1.61 (m, 1H, 4-CH), 1.58-1.51 (m, 1H, 2-CH), 1.48 (s, 9H, 3 x CH_3); ^{13}C-NMR (100 MHz, $CDCl_3$) δ 173.6 (CO), 150.3 (ArC), 149.5 (ArCH), 131.3 (6-CH), 124.3 (7-CH), 123.3 (ArCH), 81.0 (C(CH_3)), 69.0 (C), 53.0 (NCH_2), 50.0 (1-CH_2), 40.2 (3-CH), 28.2 (3 x CH_3), 27.5 (5-CH_2), 25.5 (4-CH_2), 22.6 (2-CH_2); υ_{max}/cm^{-1} 2972.83, 2929.11, 1717.75, 1601.61, 1413.64, 1367.14, 1250.06, 1155.80, 1047.59, 846.27; *m/z* HRMS (ESI$^+$) found [M + H]$^+$ 315.2065, $[C_{19}H_{27}N_2O_2]^+$ requires 315.2073.

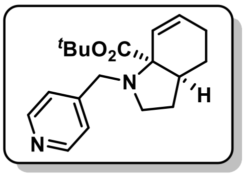

(±)-*tert*-Butyl (3a*S*,7a*R*)-1-(4-nitrobenzyl)-1,2,3,3a,4,5-hexahydro-7a*H*-indole-7a-carboxylate 16k

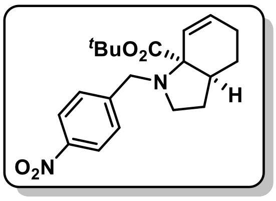

According to general procedure A, crude (±)-*tert*-butyl (3a*S*,7a*R*)-3-3a,4,5-tetrahydro-7a*H*-indole-7a-carboxylate **S7** (0.12 g, 0.52 mmol) and 4-nitrobenzaldehyde (79 mg, 0.52 mmol) was stirred with sodium triacetoxyborohydride (0.330 g, 1.56 mmol) in CH_2Cl_2 (2 mL). The crude material was purified by SiO_2 flash chromatography, 0-2.5% EtOAc/petrol, to give a yellow oil (0.13 g, 68%). ^{1}H-NMR (400 MHz, $CDCl_3$) δ 8.15 (d, 2H, *J* = 8.6 Hz, ArCH_2), 7.53 (d, 2H, *J* = 8.6 Hz, ArCH), 6.05 (dt, 1H, *J* = 10.1, 4.0 Hz, 6-CH), 5.79 (br dt, 1H, *J* = 10.1, 1.8 Hz, 7-CH), 4.00 (d, 1H, *J* = 14.3 Hz, NCHH), 3.77 (d, 1H, *J* = 14.3 Hz, NCHH), 2.72-2.66 (m, 1H, 1-CH), 2.61-2.55 (m, 2H, 1-CH and 3-CH), 2.12-1.98 (m, 3H, 2-CH and 5-CH_2), 1.81-1.73 (m, 1H, 4-CH), 1.67-1.60 (m, 1H, 2-CH), 1.55-1.53 (m, 1H, 4-CH), 1.49 (s, 9H, CH_3); ^{13}C-NMR (100 MHz, $CDCl_3$) δ 173.6 (CO), 148.9 (ArC), 146.9 (ArC), 131.5 (6-CH), 128.8 (ArCH), 124.1 (7-CH), 123.4 (ArCH), 81.1 (C(CH_3)), 69.0 (C), 53.5 (NCH_2), 49.9 (1-CH_2), 40.2 (3-CH), 28.2 (CH_3), 27.4 (2-CH_2), 25.4 (4-CH_2), 22.5 (5-CH_2); υ_{max}/cm^{-1} 2929.97, 1717.70, 1519.69, 1367.19, 1344.02, 1250.05, 1155.02, 1108.89, 1047.63, 847.31; *m/z* HRMS (ESI$^+$) found [M + H]$^+$ 359.1960, $[C_{20}H_{27}N_2O_4]^+$ requires 359.1971.

(±)-*tert*-Butyl (3a*S*,7a*R*)-1-(furan-2-ylmethyl)-1,2,3,3a,4,5-hexahydro-7a*H*-indole-7a-carboxylate 16l

According to general procedure A, crude (±)-*tert*-butyl (3a*S*,7a*R*)-3-3a,4,5-tetrahydro-7a*H*-indole-7a-carboxylate **S7** (0.10 g, 0.45 mmol) and furfural (0.04 mL, 0.45 mmol) were stirred with $NaBH(OAc)_3$ (0.290 g, 1.35 mmol) in CH_2Cl_2 (2 mL). The crude was purified by SiO_2 flash chromatography, 0-5% EtOAc/Isohexane, to give a colourless oil (94.0 mg, 67%). ^{1}H-NMR (400 MHz, $CDCl_3$) δ 7.35-7.34 (m, 1H, ArCH), 6.28-6.27 (m, 1H, ArCH), 6.15-6.14 (m, 1H, ArCH), 6.01 (dt, 1H, *J* = 10.3, 4.0 Hz, 6-CH), 5.75 (dt, 1H, *J* = 10.3, 1.9 Hz, 7-CH), 3.96 (d, 1H, *J* = 13.6 Hz, ArCHH), 3.63 (d, 1H, *J* = 13.6 Hz, ArCHH), 2.88 (td, 1H, *J* = 8.6, 4.6 Hz, 1-CH), 2.68-2.60 (m, 2H, 1-CH and 3-CH), 2.12- 2.04 (m, 1H, 5-CH), 2.03-1.94 (m, 2H, 5-CH and 2-CH), 1.80-1.73 (m, 1H, 4-CH), 1.63-1.58 (m, 1H, 4-CH), 1.56-1.51 (m, 1H, 2-CH), 1.49 (s, 9H, 3 x CH_3); ^{13}C-NMR (125 MHz, $CDCl_3$) δ 173.3 (CO), 153.8 (ArC), 141.7 (ArCH), 131.2 (6-CH), 124.0 (7-CH), 110.0 (ArCH), 109.2 (ArCH), 80.9 (C), 68.5 (C), 50.7 (1-CH_2), 46.8 (ArCH_2), 40.3 (3-CH), 28.2 (CH_3), 27.0 (2-CH_2), 25.1 (4-CH_2), 21.9 (5-CH_2); υ_{max}/cm^{-1} 2926.8, 2852.3, 1717.2, 1456.6, 1366.9, 1248.4, 1156.2, 1012.7, 847.2, 729.9; *m/z* HRMS (ESI$^+$) found [M + H]$^+$ 304.1907, $[C_{18}H_{26}NO_3]^+$ requires 304.1913.

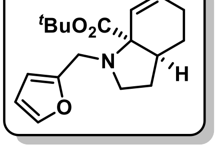

(±)-*tert*-Butyl (3a*S*,7a*R*)-1-benzyl-1,2,3,3a,4,5-hexahydro-7a*H*-indole-7a-carboxylate 16m

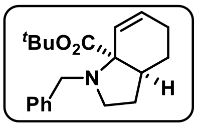

A stirred solution of aziridine **S6** (320 mg, 1.45 mmol) in toluene (15 mL) was heated to 100 °C under nitrogen. After 16 h the reaction was evaporated to give a brown oil which was redissolved in anhydrous DCM (12 mL) and cooled to 0 °C. Benzaldehyde (0.16 mL, 1.6 mmol) was added, followed by $NaBH(OAc)_3$ (1.08 g, 5.0 mmol) in one portion. The reaction was allowed to warm to rt, stirred for 14.5 h and quenched by the addition of sat. aq. $NaHCO_3$ (10 mL). The mixture was stirred for 15 min and extracted with DCM (2 × 8 mL). Drying ($MgSO_4$) and evaporation gave an orange oil which was purified by silica gel chromatography (EtOAc/petrol, 2:98 to 3:97 as eluent) to afford the title compound (345 mg, 76%) as a clear oil. ν_{max}/cm^{-1} (film) 2973, 1717, 1603, 1454, 1367 and 1248; δ_H (500 MHz, $CDCl_3$) 1.49 – 1.57 (10H, m, *Me*3C and NCH2CH*H*), 1.61 – 1.69 (1H, m, homoallylic CH*H*), 1.76 (1H, dddd, J 13.0, 7.5, 5.2, 4.4, homoallylic C*H*H), 1.98 – 2.07 (2H, m, allylic CH*H* and NCH2C*H*H), 2.12 (1 H, dddd, J 15.1, 7.5, 3.8, 2.1, allylic C*H*H), 2.57 – 2.66 (2H, m, NCH*H*CH2 and C*H*), 2.73 (1H, td, J 9.0, 4.8, NC*H*HCH2), 3.69 (1H, d, J 13.3, NCH*H*Ph), 3.89 (1H, d, J 13.3, NC*H*HPh), 5.88 (1H, dt, J 10.2, 2.1, C*H*=CH-CH2), 6.04 (1H, dt, J 10.2, 3.9, =C*H*-CH2), 7.21 – 7.26 (1H, m, CH_{Ar}), 7.31 (2H, d, J 8.9, 2 × CH_{Ar}) and 7.38 (2H, dd, J 8.9, 5.3, 2 × CH_{Ar}); δ_C (126 MHz, $CDCl_3$) 22.5 (allylic *C*H2), 25.5 (homoallylic *C*H_2), 27.4 (NCH_2*C*H_2), 28.3 (*Me*$_3$C), 40.4 (CH), 49.8 (N*C*H_2CH_2), 54.1 (N*C*H_2Ph), 69.1 (*C*$_q$-CO_2^{t}Bu), 80.8 (Me_3*C*O), 124.7 (*C*H=CH-CH_2), 126.6 (CH_{Ar}), 128.1 (2 × CH_{Ar}), 128.4 (2 × CH_{Ar}), 130.8 (=C*H*-CH_2), 140.7 (2 × C_{qAr}) and 173.8 (*C*O_2^{t}Bu); HRMS (ESI$^+$) 314.2110 ($C_{20}H_{28}NO_2$, $[M+H]^+$, requires 314.2115).

(±)-*tert*-Butyl (3a*S*,7a*R*)-1-(pyridine-2-ylmethyl)-1,2,3,3a,4,5-hexahydro-7a*H*-indole-7a-carboxylate 16n

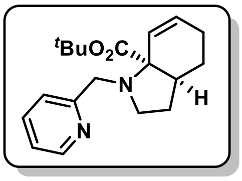

According to general procedure A, crude (±)-*tert*-butyl (3a*S*,7a*R*)-3-3a,4,5-tetrahydro-7a*H*-indole-7a-carboxylate **S7** (0.15 g, 0.68 mmol) and 2-pyridinecarboxyladehyde (0.06 mL, 0.68 mmol) were stirred with $NaBH(OAc)_3$ (0.430 g, 2.04 mmol) in CH_2Cl_2 (3 mL). The crude was purified by SiO_2 flash chromatography, 0-5% EtOAc/Isohexane, to give a yellow oil (0.13 g, 62%). ^{1}H-NMR (400 MHz, $CDCl_3$) δ 8.49-8.48 (m, 1H, ArCH), 7.63 (td, 1H, *J* = 7.7, 2.0 Hz, ArCH), 7.55 (br d, 1H, *J* = 7.7 Hz, ArCH), 7.12-7.10 (m, 1H, ArCH), 6.01 (dt, 1H, *J* = 10.3, 3.8 Hz, 6-CH), 5.84 (dt, 1H, *J* = 10.3, 2.1 Hz, 7-CH), 4.04 (d, 1H, *J* = 15.3 Hz, ArC<u>H</u>H), 3.87 (d, 1H, *J* = 15.3 Hz, ArCH<u>H</u>), 2.80 (td, 1H, *J* = 8.9, 5.0 Hz, 1-CH), 2.73-2.68 (m, 1H, 1-CH), 2.63-2.57 (m, 1H, 3-CH), 2.13-1.98 (m, 3H, 5-CH_2 and 2-CH), 1.80-1.74 (m, 1H, 4-CH), 1.67-1.60 (m, 1H, 4-CH), 1.52-1.48 (m, 1H, 2-CH), 1.48 (s, 9H, 3 x CH_3); ^{13}C-NMR (100 MHz, $CDCl_3$) δ 173.7 (CO), 161.2 (ArC), 148.6 (ArCH), 136.4 (ArCH), 130.9 (6-CH), 124.6 (7-CH), 122.4 (ArCH), 121.6 (ArCH), 80.9 (C), 69.1 (C), 56.1 ($ArCH_2$), 50.3 (1-CH_2), 40.3 (3-CH), 28.2 (3 x CH_3), 27.7 (2-CH_2), 25.6 (4-CH_2), 22.5 (5-CH_2); υ_{max}/cm^{-1} 2972.8, 2929.0, 1716.7, 1432.2, 1366.8, 1248.6, 1156.0, 1046.7, 846.6, 757.0;*m/z* HRMS (ESI$^+$) found $[M + H]^+$ 315.2061, $[C_{19}H_{27}N_2O_2]^+$ requires 315.2073.

(±)-*tert*-Butyl (3a*S*,7a*R*)-1-(thiophen-2-ylmethyl)-1,2,3,3a,4,5-hexahydro-7a*H*-indole-7a-carboxylate 16o

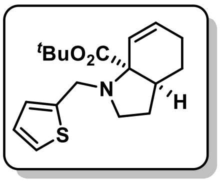

According to general procedure A, crude (±)-*tert*-butyl (3a*S*,7a*R*)-3-3a,4,5-tetrahydro-7a*H*-indole-7a-carboxylate **S7** (0.10 g, 0.45 mmol) and 2-thiphenecarboxaldehyde (0.04 mL, 0.45 mmol) were stirred with $NaBH(OAc)_3$ (0.290 g, 1.35 mmol) in CH_2Cl_2 (2 mL). The crude was purified by SiO_2 flash chromatography, 0-5% EtOAc/Isohexane, to give a colourless oil (75.0 mg, 54%). ^{1}H-NMR (400 MHz, $CDCl_3$) δ 7.18 (dd, 1H, *J* = 4.9, 1.3 Hz, ArCH), 6.91-6.88 (m, 2H, ArCH), 6.02 (dt, 1H, *J* = 10.2, 3.8 Hz, CH), 5.81 (dt, 1H, *J* = 10.2, 2.1 Hz, CH), 4.08 (d, 1H, *J* = 14.2 Hz, ArC<u>H</u>H), 3.90 (d, 1H, *J* = 14.2 Hz, ArCH<u>H</u>), 2.81 (td, 1H, *J* = 8.8, 5.0 Hz, 1-CH), 2.73-2.69 (m, 1H, 1-CH), 2.62-2.57 (m, 1H, 3-CH), 2.13-2.06 (m, 1H, 5-CH), 2.05-1.97 (m, 2H, 2-CH and 5-CH), 1.75-1.69 (m, 1H, 4-CH), 1.66-1.59 (m, 1H, 4-CH), 1.52-1.51 (m, 1H, 2-CH), 1.49 (s, 9H, 3 x CH_3); ^{13}C-NMR (125 MHz, $CDCl_3$) δ 173.6 (CO), 145.7 (ArC), 131.3 (6-CH), 126.2 (ArCH), 124.3 (7-CH), 124.2 (ArCH), 124.0 (ArCH), 80.9 (C), 68.9 (C), 50.0 (1-CH_2) 48.9 ($ArCH_2$), 40.2 (3-CH), 28.2 (3 x CH_3), 27.3 (2-CH_2), 25.4 (4-CH_2), 22.6 (5-CH_2); υ_{max}/cm^{-1} 2971.8, 2904.4, 1703.7, 1393.7, 1384.3, 1251.0, 1166.0, 1066.3, 1056.8, 1020.5; *m/z* HRMS (ESI$^+$) found $[M + H]^+$ 320.1678, $[C_{18}H_{26}NO_2S]^+$ requires 320.1684.

(±)-*tert*-Butyl (3a*S*,7a*R*)-1-(2-hyroxybenzyl)-1,2,3,3a,4,5-hexahydro-7a*H*-indole-7a-carboxylate 16p

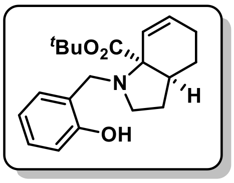

According to general procedure A, crude (±)-*tert*-butyl (3a*S*,7a*R*)-3-3a,4,5-tetrahydro-7a*H*-indole-7a-carboxylate **S7** (0.10 g, 0.45 mmol) and salicyaldehyde (0.05 mL, 0.45 mmol) were stirred with $NaBH(OAc)_3$ (0.290 g, 1.35 mmol) in CH_2Cl_2 (2 mL). The crude was purified by SiO_2 flash chromatography, 0-5% EtOAc/Isohexane, to give a colourless oil (78.0 mg, 52%). ^{1}H-NMR (400 MHz, $CDCl_3$) δ 7.15 (td, 1H, *J* = 7.4, 1.5 Hz, ArCH), 6.96 (br d, 1H, *J* = 7.5 Hz, ArCH), 6.81 (d, 1H, *J* = 8.4 Hz, ArCH), 6.75 (td, 1H, *J* = 7.4, 1.5 Hz, ArCH), 6.15 (dt, 1H, *J* = 10.4, 3.7 Hz, 6-CH), 5.81 (br t, 1H, *J* = 10.4 Hz, 7-CH), 4.13 (d, 1H, *J* = 13.4 Hz, ArC<u>H</u>H), 3.64 (d, 1H, *J* = 13.4 Hz, ArCH<u>H</u>), 2.85 (dt, 1H, *J* = 9.4, 3.9 Hz, 1-CH), 2.66-2.62 (m, 1H, 3-CH), 2.51 (m, 1H, 1-CH), 2.17-2.10 (m, 1H, 5-CH), 2.07-1.99 (m, 2H, 2-CH and 5-CH), 1.85-1.78 (m, 1H, 4-CH), 1.63-1.57 (m, 2H, 2-CH and 4-CH), 1.51 (s, 9H, 3 x CH_3); 4.08 (d, 1H, *J* = 14.3 Hz, ArC<u>H</u>H), 3.89 (d, 1H, *J* = 14.3 Hz, ArCH<u>H</u>), 2.81 (dt, 1H, *J* = 8.6, 4.9 Hz, CH), 2.73-2.69 (m, 1H, CH), 2.62-2.57 (m, 1H, CH), 2.13-2.06 (m, 1H, CH), 2.05-1.97 (m, 2H, CH), 1.75-1.69 (m, 1H, CH), 1.66-1.59 (m, 1H, CH), 1.52-1.51 (m, 1H, CH), 1.49 (s, 9H, 3 x CH_3); ^{13}C-NMR (125 MHz, $CDCl_3$) δ 173.2 (CO), 157.8 (ArC), 132.8 (6-CH), 128.6 (ArCH), 128.5 (ArCH), 122.3 (7-CH), 122.1 (ArC), 118.8 (ArCH), 115.9 (ArCH), 82.0 (C), 69.1 (C), 53.3 ($ArCH_2$), 49.8 (1-CH_2), 40.8 (3-CH), 28.0 (3 x CH_3), 26.5 (2-CH_2), 24.2 (4-CH_2), 21.5 (5-CH_2); υ_{max}/cm^{-1} 2972.6, 2928.5, 1721.5, 1588.3, 1489.4, 1367.8, 1252.7, 1160.2, 1036.7, 753.3; *m/z* HRMS (ESI$^+$) found $[M + H]^+$ 330.2061, $[C_{20}H_{27}NO_3]^+$ requires 320.2069.

***(±)-tert*-Butyl (3a*S*,7a*R*)-1-(4-fluorobenzyl)-1,2,3,3a,4,5-hexahydro-7a*H*-indole-7a-carboxylate 16q**

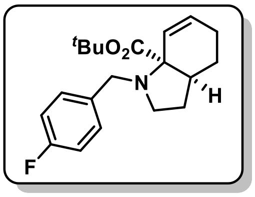

According to general procedure A, crude (±)-*tert*-butyl (3a*S*,7a*R*)-3-3a,4,5-tetrahydro-7a*H*-indole-7a-carboxylate **S7** (0.10 g, 0.45 mmol) and 4-fluorobenzaldehyde (0.05 mL, 0.45 mmol) were stirred with NaBH(OAc)$_3$ (0.290 g, 1.35 mmol) in CH_2Cl_2 (2 mL). The crude was purified by SiO_2 flash chromatography, 0-5% EtOAc/Isohexane, to give a colourless oil (71.0 mg, 47%). ^{1}H-NMR (400 MHz, $CDCl_3$) δ 7.32-7.29 (m, 2H, ArCH), 6.98-6.94 (m, 2H, ArCH), 6.02 (dt, 1H, *J* = 10.2, 3.9 Hz, 6-CH), 5.82 (dt, *J* = 10.2, 2.0 Hz, 7-CH), 3.84 (d, 1H, *J* = 13.2 Hz, ArCH̲H), 3.62 (d, 1H, *J* = 13.2 Hz, ArCHH̲), 2.67 (td, 1H, *J* = 8.8, 4.6 Hz, 1-CH), 2.61-2.53 (m, 2H, 1-CH and 3-CH), 2.13-2.06 (m, 1H, 2-CH), 2.04-1.96 (m, 2H, 2-CH and 5-CH), 1.77-1.71 (m, 1H, 4-CH), 1.65-1.58 (m, 1H, 4-CH), 1.54-1.50 (m, 1H, 5-CH), 1.49 (s, 9H, 3 x CH_3); ^{13}C-NMR (125 MHz, $CDCl_3$) δ 173.7 (CO), 161.8 (d, *J* = 246.6 Hz, ArCF), 136.3 (ArC), 130.9 (6-CH), 129.1 (d, *J* = 8.2 Hz, ArCH), 124.5 (7-CH), 114.7 (d, *J* = 21.2 Hz, ArCH), 80.8 (C), 67.0 (C), 53.3 ($ArCH_2$), 49.7 (1-CH_2), 40.3 (3-CH), 28.2 (3 x CH_3), 27.3 (5-CH_2), 25.4 (4-CH_2), 22.4 (2-CH_2); ^{19}F{^{1}H}-NMR (377 MHz, $CDCl_3$) δ -116.69; $υ_{max}$/cm^{-1} 2930.3, 1717.7, 1603.0, 1508.1, 1367.0, 1247.2, 1220.6, 1151.6, 848.4, 825.7; *m/z* HRMS (ESI$^+$) found [M + H]$^+$ 332.2014, $[C_{20}H_{27}FNO_2]^+$ requires 332.2026.

***(±)-tert*-Butyl (3a*S*,7a*R*)-1-((1-methyl-1H-pyrol-2-yl)methyl)-1,2,3,3a,4,5-hexahydro-7a*H*-indole-7a-carboxylate 16r**

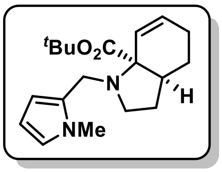

According to general procedure A, crude (±)-*tert*-butyl (3a*S*,7a*R*)-3-3a,4,5-tetrahydro-7a*H*-indole-7a-carboxylate **S7** (0.15 g, 0.68 mmol) and *N*-methyl-2-pyrrolecarboxylaldehyde 75.0 mg, 0.68 mmol) were stirred with NaBH(OAc)$_3$ (0.430 g, 2.04 mmol) in CH_2Cl_2 (3 mL). The crude was purified by SiO_2 flash chromatography, 0-5% EtOAc/Isohexane, to give a yellow oil (91.0 mg, 41%). ^{1}H-NMR (400 MHz, $CDCl_3$) δ 6.55 (t, 1H, *J* = 2.2 Hz, ArCH), 5.99-5.98 (m, 1H, ArCH) superimposed on 5.98-5.96 (m, 1H, 6-CH), superimposed on 5.95-5.94 (m, 1H, ArCH), 5.95-5.94 (m, 1H, 6-CH), 5.87 (dt, 1H, *J* = 10.2, 2.1 Hz, 7-CH), 3.73 (d, 2H, *J* = 2.5 Hz, $ArCH_2$), 3.62 (s, 3H, CH_3), 2.64-2.61 (m, 2H, 1-CH_2), 2.55-2.54 (m, 1H, 3-CH), 2.05-1.97 (m, 3H, 2-CH and 5-CH_2), 1.69-1.61 (m, 1H, 4-CH), 1.59-1.56 (m, 2H, 2-CH and 4-CH), 1.49 (s, 9H, 3 x CH_3); ^{13}C-NMR (100 MHz, $CDCl_3$) δ 179.6 (CO), 173.4 (ArC), 130.7 (6-CH), 124.6 (7-CH), 122.2 (ArCH), 108.3 (ArCH), 105.9 (ArCH), 80.9 (C), 68.4 (C), 49.7 (1-CH), 45.4 ($ArCH_2$), 40.6 (3-CH), 34.0 (CH3), 28.3 (3 x CH_3), 27.5 (5-CH_2), 26.2 (2-CH_2), 23.1 (5-CH_2); 2979.9, 2928.7, 1715.8, 1392.4, 1368.1, 1250.9, 1158.5, 1073.3, 1048.8, 1028.5; *m/z* HRMS (ESI$^+$) found [M + H]$^+$ 317.2220, $[C_{19}H_{29}N_2O_2]^+$ requires 317.2229.

***(±)-tert*-Butyl (3a*S*,7a*R*)-1-(benzofuran-2-ylmethyl)-1,2,3,3a,4,5-hexahydro-7a*H*-indole-7a-carboxylate 16s**

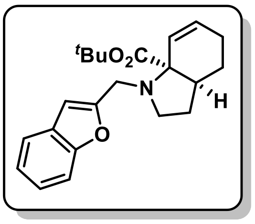

According to general procedure A, crude (±)-*tert*-butyl (3a*S*,7a*R*)-3-3a,4,5-tetrahydro-7a*H*-indole-7a-carboxylate **S7** (0.12 g, 0.52 mmol) and 2-benzofurancarboxaldehyde (0.06 mL, 0.52 mmol) was stirred with sodium triacetoxyborohydride (0.33 g, 1.56 mmol) in CH_2Cl_2 (2 mL). The crude product was purified by SiO_2 flash chromatography, 0-10% EtOAc/petrol to give the product as a yellow oil (0.12 g, 67%). ^{1}H-NMR (400 MHz, $CDCl_3$) δ 7.48-7.42 (m, 2H, ArCH), 7.21-7.13 (m, 3H, ArCH), 6.54 (br s, 1H, ArCH), 6.03 (dt, 1H, *J* = 10.2, 3.7 Hz, 6-CH), 5.78 (dt, 1H, *J* = 10.2, 2.1 Hz, CH), 4.10 (d, 1H, *J* = 15.0 Hz, NCH̲H), 3.78 (d, 1H, *J* = 15.0 Hz, NCHH̲), 2.93 (td, 1H, *J* = 8.7, 4.5 Hz, 1-CH), 2.73 (td, 1H, *J* = 8.7, 9.1 Hz, 1-CH), 2.67-2.60 (m, 1H, 3-CH), 2.12-1.97 (m, 3H, 2-CH and 5-CH_2), 1.80-1.73 (m, 1H, 4-CH), 1.65-1.60 (m, 1H, 4-CH), 1.58-1.53 (m, 1H, 2-CH), 1.47 (s, 9H, CH_3); ^{13}C-NMR (100 MHz, $CDCl_3$) δ 173.3 (CO), 157.1 (ArC), 155.0 (ArC), 131.5 (6-CH), 128.6 (ArC), 123.9 (7-CH), 123.4 (ArCH), 122.4 (ArCH), 120.5 (ArCH), 111.2 (ArCH), 103.9 (ArCH), 81.1 ($C(CH_3)$), 68.7 (C), 50.8 (1-CH_2), 47.4 (NCH_2), 40.2 (3-CH), 28.2 (3 x CH_3), 27.1 (2-CH_2), 25.2 (4-CH_2), 22.1 (5-CH_2); $υ_{max}$/cm^{-1} 2928.74, 1717.57, 1454.52, 1367.35, 1253.88, 1155.80, 1049.53, 1032.99, 751.26, 742.10; *m/z* HRMS (ESI$^+$) found [M + H]$^+$ 354.2061, $[C_{22}H_{28}NO_3]^+$ requires 354.2069.

***(±)-tert*-Butyl (3a*S*,7a*R*)-1-tosyl-1,2,3,3a,4,5-hexahydro-7a*H*-indole-7a-carboxylate 16t**

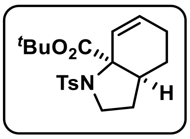

To a stirred solution of substrate **S7** (158 mg, 0.709 mmol) in anhydrous DCM (4 mL) at rt under nitrogen was added $^iPr_2NEt$ (0.14 mL, 0.80 mmol) and the mixture cooled to 0 °C. TsCl (136 mg, 0.71 mmol) was added and the reaction allowed to warm to rt over h. After 3 h the reaction was quenched by the addition of sat. aq. $NaHCO_3$ (10 mL) and the mixture extracted with DCM (2 × 10 mL). Drying ($MgSO_4$) and evaporation gave a light brown oil. Purification by silica gel chromatography (EtOAc/petrol, 1:9 to 15:85 as eluent) afforded the title compound (230 mg, 86%) as a clear oil. v_{max}/cm^{-1} (film) 2927, 1727, 1362 and 1154; $δ_H$ (500 MHz, $CDCl_3$) 1.54 (10H, m, Me_3C and homoallylic CH*H*), 1.65 – 1.79 (2 H, m, homoallylic C*H*H and NCH_2CHH), 1.96 – 2.09 (3 H, m, allylic C*H*$_2$ and NCH_2CHH), 2.42 (3 H, s, *Me*), 2.51 (1 H, qd, J 7.1, 4.1, CH), 3.31 (1 H, td, J 8.3, 4.9, NCH*H*), 3.50 (1 H, td, J 8.8, 7.7, NC*H*H), 5.92 (1 H, dt, J 10.3, 3.8, =C*H*-CH_2), 6.21 (1 H, dt, J 10.2, 2.1, C*H*=CH-CH_2), 7.22 – 7.33 (2 H, m, 2 × CH_{Ar}) and 7.71 – 7.81 (2 H, m, 2 × CH_{Ar}); $δ_C$ (126 MHz, $CDCl_3$) 21.4 (allylic CH_2), 21.5 (Me), 22.9 (homoallylic CH_2), 27.8 (NCH_2CH_2), 27.9 (Me_3C), 43.2 (CH), 46.4 (NCH_2), 69.6 (C_q-$CO_2{}^tBu$), 81.9 (Me_3CO), 125.8 (*C*H=CH-CH_2), 127.3 (2 × CH_{Ar}), 128.7 (=*C*H-CH_2), 129.3 (2 × CH_{Ar}), 138.0 (C_{qAr}), 142.7 (C_{qAr}) and 172.6 ($CO_2{}^tBu$); HRMS (ESI$^+$) 400.1567 ($C_{20}H_{27}NO_4S$, [M+Na]$^+$, requires 400.1553).

Synthesis of Morphan Products

(±)-*tert*-Butyl (1*R*)-2-(3,4-dimethoxyphenethyl)-2-azabicyclo[3.3.1]non-6-ene-6-carboxylate 11 (17a)

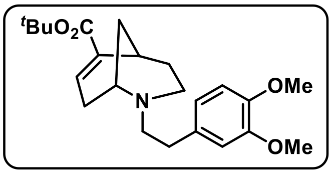

Pd(OAc)$_2$ (2 mg, 0.01 mmol) and DPEPhos (8 mg, 0.02 mmol) were stirred in dioxane (0.50 mL) for 10 min and the reaction mixture turned bright yellow. (±)-*tert*-Butyl (3*R*,7a*S*)-1-iodo-4, 5-dimethoxyphenethyl)-1,2,3,3a,4,5-hexhydro-7a*H*-indole-7a-carboxylate **10a** (50 mg, 0.10 mmol) in dioxane (0.50 mL) and *N*,*N*-diisopropylethylamine (0.030 mL, 0.19 mmol) were added to the stirred reaction solution and heated to reflux. After 17 h, the reaction mixture was cooled to RT, quenched with NaHCO$_3$ (saturated aqueous solution, 15 mL) and extracted with CH$_2$Cl$_2$ (3 x 5 mL). The combined organic layers were dried over MgSO$_4$, filtered and concentrated *in vacuo*. The crude was purified by SiO$_2$ flash chromatography, 30-50% EtOAc/petrol, to give a yellow oil (29 mg, 76%). ^{1}H-NMR (400 MHz, CDCl$_3$) δ 7.04 (t, 1H, *J* = 3.9 Hz, 7-CH), 6.79-6.73 (m, 3H, ArCH), 3.86 (s, 3H, OCH$_3$), 3.84 (s, 3H, OCH$_3$), 3.13 (br s, 1H, 1-CH), 2.89 (br s, 1H, 5-CH), 2.75-2.56 (m, 5H, 3-CH, NCH$_2$ and NCH$_2$C$\underline{H}_2$), 2.41 (dd, 1H, *J* = 20.9, 3.9 Hz, 8-CH), 2.25 (td, 1H, *J* = 12.2, 3.8 Hz, 3-CH), 2.09-2.02 (m, 1H, 8-CH), 1.96 (br d, 1H, *J* = 12.4 Hz, 9-CH), 1.88 (tt, 1H, *J* = 12.8, 3.8 Hz, 4-CH), 1.64-1.60 (m, 1H, 9-CH), 1.52 (br s, 1H, 4-CH), 1.47 (s, 9H, CH$_3$); υ_{max}/cm^{-1} 2975, 2935, 1699, 1515, 1367, 1259, 1157, 1139, 1026; ^{13}C-NMR (100 MHz, CDCl$_3$) δ 166.8 (CO), 148.7 (ArC), 147.3 (ArC), 139.9 (7-CH), 134.2 (6-C), 133.1 (ArC), 120.5 (ArCH), 79.9 (C), 57.6 (NCH$_2\underline{C}$H$_2$), 55.7 (OCH$_3$), 50.3 (1-CH), 44.4 (3-CH), 34.0 (NCH$_2$), 31.8 (9-CH), 28.8 (4-CH), 28.1 (CH$_3$), 26.7 (5-CH), 24.8 (8-CH); *m/z* HRMS (ESI$^+$) found [M + H]$^+$ 388.2496, [C$_{23}$H$_{34}$NO$_4$]$^+$ requires 388.2485.

(±)-*tert*-Butyl (1*R*)-2-phenethyl-2-azabicyclo[3.3.1]non-6-ene-6-carboxylate 17g

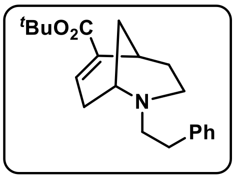

Pd(OAc)$_2$ (3.0 mg, 0.01 mmol), DPEPhos (10.0 mg, 0.02 mmol) and *N*,*N*-diisopropylethylamine (0.040 mL, 0.24 mmol) were stirred in dioxane (0.5 mL) for 10 min. The reaction mixture turned bright yellow then turned orange. (±)-*tert*-Butyl (3a*R*,7a*S*)-1-(2-iodophenethyl)- 1,2,3,3a,4,5-hexhydro-7a*H*-indole-7a-carboxylate **S14** (53.0 mg, 0.12 mmol) in dioxane (0.5 mL) was added to the stirred reaction solution and heated to reflux. After 17 h, the reaction mixture was cooled to RT, quenched with NaHCO$_3$ (saturated aqueous solution, 10 mL) and extracted with CH$_2$Cl$_2$ (3 x 5 mL). The combined organic layers were dried over MgSO$_4$, filtered and concentrated *in vacuo*. The crude was purified by SiO$_2$ flash chromatography, 10-50% EtOAc/Petrol, to give a yellow oil (29.0 mg, 74%). ^{1}H-NMR (400 MHz, CDCl$_3$) δ 7.34-7.31 (m, 2H, ArCH), 7.26-7.22 (m, 3H, ArCH), 7.09 (t, 1H, *J* = 3.6 Hz, 7-CH), 3.19 (br s, 1H, 1-CH), 2.94 (br s, 1H, 5-CH), 2.85-2.81 (m, 2H, NCH$_2$), 2.76-2.73 (m, 1H, 3-CH), 2.70-2.63 (m, 2H, NCH$_2$C$\underline{H}_2$), 2.46 (dd, 1H, *J* = 20.7, 4.3 Hz, 8-CH), 2.30 (td, 1H, *J* = 12.4, 3.3 Hz, 3-CH), 2.13-2.07 (m, 1H, 8-CH), 2.01 (br dt, *J* = 12.3, 2.8 Hz, 1H, 9-CH), 1.93 (tt, 1H, *J* = 12.9, 4.3 Hz, 4-CH), 1.69-1.65 (m, 1H, 9-CH), 1.58-1.57 (m, 1H, 4-CH), 1.53 (s, 9H, CH$_3$); ^{13}C-NMR (125 MHz, CDCl$_3$) δ 165.8 (CO), 140.5 (6-CH), 140.0 (7-CH), 134.3 (ArC), 128.7 (ArCH), 128.3 (ArCH), 126.0 (ArCH), 79.9 (C), 57.5 (NCH$_2\underline{C}$H$_2$), 50.3 (1-CH), 44.5 (3-CH), 34.5 (NCH$_2$), 31.8 (9-CH), 28.9 (4-CH), 28.2 (CH$_3$), 26.8 (5-CH), 24.8 (8-CH); υ_{max}/cm^{-1} 2930, 1701, 1366 and 1251; *m/z* HRMS (ESI$^+$) found [M + H]$^+$ 328.2285, [C$_{21}$H$_{30}$NO$_2$]$^+$ requires 328.2277.

(±)-*tert*-Butyl (1*R*)-2-(4-methoxyphenethyl)-2-azabicyclo[3.3.1]non-6-ene-6-carboxylate S30

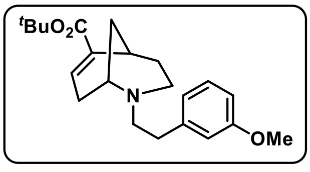

Pd(OAc)$_2$ (2.0 mg, 0.01 mmol), DPEPhos (9.0 mg, 0.02 mmol) and *N*,*N*-diisopropylethylamine (0.040 mL, 0.22 mmol) were stirred in dioxane (0.5 mL) for 10 min. The reaction mixture turned bright yellow. (±)-*tert*-Butyl (3*R*,7a*S*)-1-(2-iodo-5-methoxyphenethyl)-1,2,3,3a,4,5-hexhydro-7a*H*-indole-7a-carboxylate **S18** (52.0 mg, 0.11 mmol) in dioxane (0.5 mL) was added to the stirred reaction and heated to reflux. After 17 h, the reaction mixture was cooled to RT, quenched with NaHCO$_3$ (saturated aqueous solution, 15 mL) and extracted with CH$_2$Cl$_2$ (3 x 5 mL). The combined organic layers were dried over MgSO$_4$, filtered and concentrated *in vacuo*. The crude was purified by SiO$_2$ flash chromatography, 30-50% EtOAc/Petrol, to give a yellow oil (39.0 mg, 21%). ^{1}H-NMR (400 MHz, CDCl$_3$) δ 7.20 (t, 1H, *J* = 7.8 Hz, ArCH), 7.04 (t, 1H, *J* = 3.7 Hz, 7-CH), 6.81-6.73 (m, 3H, ArCH), 3.80 (s, 3H, OCH$_3$), 3.14 (br s, 1H, 1-CH), 2.89 (br s, 1H, 5-CH), 2.79-2.74 (m, 2H, NCH$_2$), 2.71-2.68 (m, 1H, 3-CH), 2.65-2.60 (m, 2H, NCH$_2$C$\underline{H}_2$), 2.43-2.39 (m, 1H, 8-CH), 2.25 (br t, 1H, *J* = 12.3 Hz, 3-CH), 2.09-2.03 (m, 1H, 8-CH), 1.98-1.85 (m, 1H, 9-CH), 1.92-1.85 (m, 1H, 4-CH), 1.65-1.61 (m, 1H, 9-CH), 1.53 (br s, 1H, 4-CH), 1.48 (s, 9H, CH$_3$); ^{13}C-NMR (125 MHz, CDCl$_3$) δ 165.8 (CO), 159.6 (ArC), 142.2 (6-C), 140.0 (7-CH), 134.3 (ArC), 129.3 (ArCH), 121.1 (ArCH), 114.5 (ArCH), 111.3 (ArCH), 80.0 (C), 57.3 (NCH$_2\underline{C}$H$_2$), 55.1 (OCH$_3$), 50.4 (1-CH), 44.5 (3-CH), 34.5 (NCH$_2$), 31.8 (9-CH), 28.9 (4-CH), 28.0 (CH$_3$), 26.8 (5-CH), 24.8 (8-CH); υ_{max}/cm^{-1} 2931, 1701, 1601, 1585, 1454, 1251; *m/z* HRMS (ESI$^+$) found [M + H]$^+$ 358.2393, [C$_{22}$H$_{32}$NO$_3$]$^+$ requires 358.2377.

(±)-*tert*-Butyl (1*R*)-2-(3-fluorophenethyl)2-azabicylo[3.3.1]non-6-ene-6-carboxylate S31

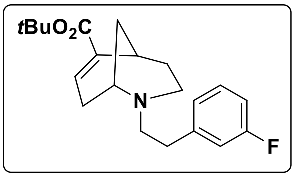

Pd(OAc)$_2$ (3.0 mg, 0.01 mmol), DPEPhos (11.0 mg, 0.02 mmol) and *N*,*N*-diisopropylethylamine (0.050 mL, 0.28 mmol) were stirred in dioxane (0.6 mL) for 10 min. The reaction mixture turned orange. (±)-*tert*-Butyl (3a*S*,7a*R*)-1-(5-fluoro-2-iodophenethyl)- 1,2,3,3a,4,5-hexhydro-7a*H*-indole-7a-carboxylate **S23** (65.0 mg, 0.14 mmol) in dioxane (0.7 mL) was added to the stirred reaction and heated to reflux. After 17 h, the reaction mixture was cooled to RT, quenched with NaHCO$_3$

(saturated aqueous solution, 10 mL) and extracted with CH_2Cl_2 (3 x 5 mL). The combined organic layers were dried over $MgSO_4$, filtered and concentrated *in vacuo*. The crude was purified by SiO_2 flash chromatography, 30-40% EtOAc/Petrol, to give a brown oil (16.0 mg, 33%). ^{1}H-NMR (400 MHz, $CDCl_3$) δ 7.23 (q, 1H, *J* = 6.7 Hz, ArCH), 7.04 (br s, 1H, 7-CH), 6.98 (d, 1H, *J* = 7.5 Hz, ArCH), 6.93-6.87 (m, 2H, ArCH), 3.11 (s, 1H, 1-CH), 2.89 (s, 1H, 5-CH), 2.81-2.76 (m, 2H, NCH_2), 2.69-2.60 (m, 3H, 3-CH and $NCH_2C\underline{H}_2$), 2.40 (br d, 1H, *J* = 20.4 Hz, 8-CH), 2.26 (t, 1H, *J* = 11.6 Hz, 3-CH), 2.07 (br d, *J* = 20.4 Hz, 8-CH), 1.95 (br d, 1H, *J* = 12.1 Hz, 9-CH), 1.87 (br t, 1H, *J* = 12.6 Hz, 4-CH), 1.63 (br d, 1H, *J* = 12.6 Hz, 9-CH), 1.52 (s, 1H, 4-CH), 1.48 (s, 9H, CH_3); ^{13}C-NMR (100 MHz, $CDCl_3$) δ 165.8 (CO), 162.8 (d, *J* = 245.0 Hz, ArCF), 143.2 (ArC), 139.9 (7-CH), 134.3 (6-CH), 129.7 (d, *J* = 8.3 Hz, ArCH), 124.4 (d, *J* = 2.8 Hz, ArCH), 115.5 (d, *J* = 20.9, ArCH), 112.8 (d, *J* = 21.1 Hz, ArCH), 80.0 (C), 57.0 ($NCH_2\underline{C}H_2$), 50.5 (1-CH), 44.4 (3-CH_2), 34.2 (NCH_2), 31.8 (9-CH_2), 28.8 (4-CH_2), 28.2 (CH_3), 26.8 (5-CH), 24.9(8-CH_2); υ_{max}/cm^{-1} 2930.23, 1701.68, 1588.91, 1448.24, 1367.03, 1284.53, 1251.16, 1167.65, 1137.72, 1077.14; *m/z* HRMS (ESI$^+$) found [M + H]$^+$ 346.2177, $[C_{21}H_{29}FNO_2]^+$ requires 346.2182.

(±)-tert-*Butyl (1*R*)*-2-(4-methoxybenzyl)-2-azabicyclo[3.3.1]non-6-ene-6-carboxylate 13 (17e)

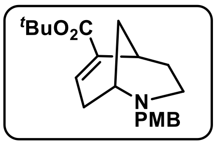

According to general procedure C, (±)-*tert*-butyl (3a*S*,7a*R*)- 1- (4-methoxybenzyl) - 1, 2, 3, 3a, 4, 5-hexahydro-7a*H*-indole-7a-carboxylate **16e** (62.0 mg, 0.18 mmol) in dioxane (0.8 mL) was added to a stirred solution of $Pd(OAc)_2$, DPEPhos and *N*,*N*-diisopropylethylamine in dioxane (0.8 mL). After 10 min methanesulfonic acid was added and the reaction was heated under reflux. The crude was purified by SiO_2 flash chromatography, eluting with 10-20% EtOAc/isohexane to give a yellow oil (43 mg, 70%). ^{1}H-NMR (400 MHz, $CDCl_3$) δ 7.29-7.28 (m, 2H, ArCH), 7.10 (t, 1H, *J* = 3.6 Hz, 7-CH), 6.88-6.86 (m, 2H, ArCH) 3.82 (s, 3H, OCH_3), 3.52 (d, 2H, *J* = 6.5 Hz, $ArCH_2$), 3.01 (br s, 1H, 1-CH), 2.90 (br s, 1H, 5-CH), 2.56-2.42 (m, 2H, 3-CH and 8-CH), 2.26 (td, 1H, *J* = 12.5, 3.4 Hz, 3-CH), 2.07-2.00 (m, 1H, 8-CH), 1.94 (br d, 1H, *J* = 12.2 Hz, 9-CH), 1.90-1.81 (tt, 1H, *J* = 12.7, 4.3 Hz, 4-CH), 1.59 (br dq, 1H, *J* = 12.2, 2.7 Hz, 9-CH), 1.51 (s, 9H, CH_3) superimposed on 1.49 (m, 1H, 4-CH); ^{13}C-NMR (100 MHz, $CDCl_3$) δ 165.9 (CO), 158.5 (ArC), 140.2 (7-CH), 134.4 (6-C), 131.3 (ArC), 129.9 (ArCH), 113.6 (ArCH), 79.9 (C), 58.9 ($ArCH_2$), 55.2 (OCH_3), 49.5 (1-CH), 44.2 (3-CH), 31.9 (9-CH), 28.9 (4-CH), 28.2 (CH_3), 26.9 (5-CH), 24.8 (8-CH); υ_{max}/cm^{-1} 1700.74, 1511.28, 1366.23, 1284.47, 1245.48, 1166.89, 1071.83, 1131.65, 1036.54, 1025.76; *m/z* HRMS (ESI$^+$) found [M + H]$^+$ 344.2232, $[C_{21}H_{30}NO_3]^+$ requires 344.2226.

(±)-*tert*-Butyl (1*R*)-2-(3,4-dimethoxyphenethyl)-2-azabicyclo[3.3.1]non-6-ene-6-carboxylate (17a)

According to general procedure C, (±)-*tert*-butyl (3a*S*, 7a*R*)-1-(3,4-dimethoxyphenethyl)-1, 2, 3, 3a, 4, 5-hexahydro-7a*H*-indole-7a-carboxylate **16a** (58 mg, 0.15 mmol) in dioxane (0.6 mL) was added to a stirred solution of $Pd(OAc)_2$ (3 mg, 0.02 mmol), DPEPhos (12 mg, 0.02 mmol) and *N*,*N*-diisopropylethylamine (0.03 mL, 0.15 mmol) in dioxane (0.6 mL). After 10 min methanesulfonic acid (0.010 mL, 0.15 mmol) was added and the reaction was heated under reflux. The crude was purified by SiO_2 flash chromatography, eluting with 10-60% EtOAc/petrol to give a yellow oil (45 mg, 78%).

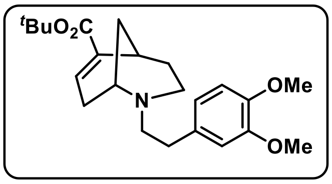

(±)-*tert*-Butyl (1*R*)-2-pentyl-2-azabicyclo[3.3.1]non-6-ene-6-carboxylate 17b

According to general procedure C, (±)-*tert*-butyl (3a*R*,7a*R*)-1-pentyl-)-1,2,3,3a,4,5-hexahydro-7a*H*-indole-7a-carboxylate **16b** (44 mg, 0.15 mmol) in dioxane (0.6 mL) was added to a stirred solution of $Pd(OAc)_2$ (3 mg, 0.02 mmol), DPEPhos (12 mg, 0.02 mmol) and *N*,*N*-diisopropylethylamine (0.03 mL, 0.15 mmol) in dioxane (0.6 mL). After 10 min methanesulfonic acid (0.01 mL, 0.15 mmol) was added and the reaction was heated 100 °C. The crude was purified by SiO_2 flash chromatography, eluting with 10-40% EtOAc/petrol to give a yellow oil (33 mg, 75%). ^{1}H-NMR (400 MHz, $CDCl_3$) δ 7.04 (t, 1H, *J* = 3.5 Hz, 7-CH), 3.07 (br s, 1H, 1-CH), 2.87 (t, 1H, *J* = 2.9 Hz, 5-CH), 2.59 (dd, 1H, *J* = 11.8, 4.9 Hz, 3-CH), 2.42-2.33 (m, 3H, 8-CH and NCH_2), 2.17 (td, 1H, *J* = 12.3, 3.4 Hz, 3-CH), 2.05-1.98 (m, 1H, 8-CH), 1.92 (br d, 1H, *J* = 12.5 Hz, 4-CH), 1.85 (tt, 1H, *J* = 12.6, 4.5 Hz, 9-CH), 1.63-1.57 (m, 1H, 9-CH), 1.48 (s, 9H, 3 x CH_3) superimposed on 1.47-1.44 (m, 3H, 4-CH and CH_2), 1.35-1.25 (m, 4H, CH_2 and CH_2), 0.89 (t, 3H, *J* = 7.8 Hz, CH_3); ^{13}C-NMR (100 MHz, $CDCl_3$) δ 165.9 (CO), 140.2 (7-CH), 134.3 (6-C), 79.9 (C), 55.3 (NCH_2), 49.8 (1-CH), 44.6 (3-CH_2), 31.9 (9-CH_2), 29.9 (CH_2), 28.9 (4-CH_2), 28.2 (3 x CH_3), 27.3 (CH_2), 26.9 (5-CH), 24.5 (8-CH_2), 22.7 (CH_2), 14.1 (CH_3); υ_{max}/cm^{-1} 2931.11, 2860.00, 1703.96, 1645.68, 1367.05, 1334.91, 1284.47, 1251.39, 1169.94, 1080.23; *m/z* HRMS (ESI$^+$) found [M + H]$^+$ 294.2435, $[C_{18}H_{32}NO_4]^+$ requires 294.2433.

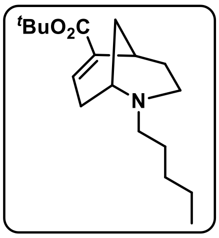

(±)-*tert*-Butyl (1*R*)-2-(3,3,3-trifluoropropyl)-2-azabicyclo[3.3.1]non-6-ene-6-carboxylate 17c

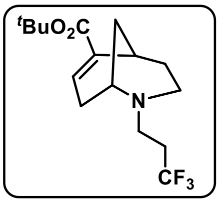

According to general procedure C, (±)-*tert*-butyl (3a*R*,7a*R*)-1-(3,3,3-trifluoropropyl)-1,2,3,3a,4,5-hexahydro-7a*H*-indole-7a-carboxylate **16c** (48 mg, 0.15 mmol) in dioxane (0.6 mL) was added to a stirred solution of $Pd(OAc)_2$ (3 mg, 0.02 mmol), DPEPhos (12 mg, 0.02 mmol) and *N*,*N*-diisopropylethylamine (0.030 mL, 0.15 mmol) in dioxane (0.6 mL). After 10 min methanesulfonic acid (0.010 mL, 0.15 mmol) was added and the reaction was heated under 100 °C. The crude was purified by SiO_2 flash chromatography, eluting with 5-50% EtOAc/petrol to give a yellow oil (34 mg, 71%). ^{1}H-NMR (400 MHz, $CDCl_3$) δ 7.05 (t, 1H, *J* = 3.5 Hz, 7-CH), 3.02 (br s, 1H, 1-CH), 2.87 (br s, 1H, 5-CH), 2.69-2.53 (m, 3H, NCH_2 and 3-CH), 2.38-2.21 (m, 4H, 3-CH, 8-CH and CH_2CF_3), 2.13-2.05 (m, 1H, 8-CH), 1.90 (br d, 1H, *J* = 12.2 Hz, 9-CH), 1.82 (tt, 1H, *J* = Hz, 12.9, 4.3

Hz, 4-CH), 1.64-1.60 (m, 2H, 4-CH and 9-CH), 1.48 (s, 9H, CH_3); ^{13}C-NMR (100 MHz, $CDCl_3$) δ 165.7 (CO), 139.7 (7-CH), 134.3 (6-C), 126.7 (q, *J* = 280.5 Hz, CF_3), 80.0 (C), 50.9 (1-CH), 48.0 (NCH_2), 44.2 (3-CH_2), 32.9 (q, *J* = 27.3 Hz, $\underline{C}H_2CF_3$), 31.8 (9-CH_2), 28.8 (4-CH_2), 28.2 (CH_3), 26.6 (5-CH), 25.2 (8-CH_2); υ_{max}/cm^{-1} 1703.10, 1367.70, 1336.32, 1285.72, 1251.64, 1222.46, 1145.09, 1122.38, 1080.89, 996.00; *m/z* HRMS (ESI$^+$) found [M + H]$^+$ 320.1831, $[C_{16}H_{25}F_3NO_2]^+$ requires 320.1837.

(±)-*tert*-Butyl (1*R*)--2-(3,4-dimethoxybenzyl)-2-azabicyclo[3.3.1]non-6-ene-6-carboxylate 17d

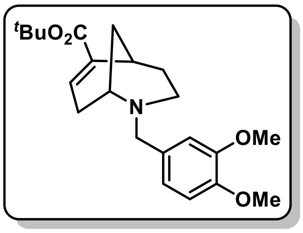

According to general procedure C, *tert*-butyl (±)-(3a*S*,7a*R*)-1-(3,4-dimethoxybenzyl) - 1,2,3,3a,4,5 - hexahydro - 7a*H* - indole - 7a -carboxylate **16d** (56.0 mg, 0.15 mmol) in dioxane (0.6 mL) was added to a stirred solution of $Pd(OAc)_2$, DPEPhos and *N*,*N*-diisopropylethylamine in dioxane (0.6 mL). After 10 min methanesulfonic acid was added and the reaction was heated under 100 °C. The crude was purified by SiO_2 flash chromatography, eluting with 10-30% EtOAc/Isohexane to give a yellow oil (39.0 mg, 70%). ^{1}H-NMR (400 MHz, $CDCl_3$) δ 7.06 (t, 1H, *J* = 3.6 Hz, 7-CH), 6.93 (br s, 1H, ArCH), 6.84-6.78 (m, 2H, ArCH), 3.88 (s, 3H, OCH_3), 3.86 (s, 3H, OCH_3), 3.49 (d, 2H, $ArCH_2$), 2.98 (br s, 1H, 1-CH), 2.87 (br s, 1H, 5-CH), 2.54-2.51 (m, 1H, 3-CH), 2.48-2.44 (m, 1H, 8-CH), 2.24 (td, *J* = 12.3, 3.2 Hz, 1H, 3-CH), 2.05-1.99 (m, 1H, 8-CH), 1.95-1.92 (m, 1H, 9-CH), 1.87-1.80 (m, 1H, 4-CH), 1.59-1.55 (m, 1H, 9-CH), 1.48 (s, 9H, 3 x CH_3) superimposed on 1.48-1.46 (m, 1H, 4-CH); ^{13}C-NMR (100 MHz, $CDCl_3$) δ 177.9 (CO), 148.9 (ArC), 147.9 (ArC), 140.1 (7-CH), 134.4 (6-CH), 131.8 (ArC), 120.7 (ArCH), 111.9 (ArCH), 110.7 (ArCH), 79.9 (C), 59.2 ($ArCH_2$), 55.9 (OCH_3), 55.8 (OCH_3), 49.5 (1-CH), 44.2 (3-CH_2), 31.8 (9-CH_2), 28.9 (4-CH_2), 28.2 (3 x CH_3), 26.9 (5-CH), 24.9 (8-CH_2); υ_{max}/cm^{-1} 1700.5, 1512.9, 1463.8, 1366.0, 1252.9, 1233.4, 1156.4, 1138.5, 1072.8, 1027.2; *m/z* HRMS (ESI$^+$) found [M + H]$^+$ 374.2315, $[C_{22}H_{32}NO_4]^+$ requires 374.2331.

***(±)*-tert-*Butyl (1*R*)*-2-ethyl-2-azabicyclo[3.3.1]non-6-ene-6-carboxylate 17f**

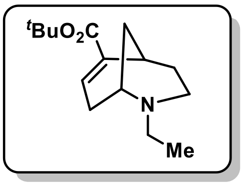

According to general procedure C, (±)-*tert*-butyl (3a*S*,7a*R*)-1-ethyl- 1,2,3,3a,4,5-hexahydro-7a*H*-indole-7a-carboxylate **16f** (38.0 mg, 0.15 mmol) in dioxane (0.6 mL) was added to a stirred solution of $Pd(OAc)_2$, DPEPhos and *N*,*N*-diisopropylethylamine in dioxane (0.6 mL). After 10 min methanesulfonic acid was added and the reaction was heated under 100 °C. The crude was purified by SiO_2 flash chromatography, eluting with 10-50% EtOAc/isohexane then 10% MeOH/ CH_2Cl_2 to give a brown oil (26.0 mg, 68%). ^{1}H-NMR (400 MHz, $CDCl_3$) δ 7.00 (t, 1H, *J* = 3.7 Hz, 7-CH), 3.53 (br s, 1H, 1-CH), 3.03 (br d, 1H, *J* = 12.4 Hz, 3-CH), 2.98 (br t, 1H, *J* = 3.1 Hz, 5-CH), 2.91-2.84 (m, 1H, NC$\underline{H}$H), 2.80-2.74 (m, 1H, NCH$\underline{H}$), 2.55-2.47 (m, 2H, 3-CH and 8-CH), 2.38-2.29 (m, 2H, 8-CH and 9-CH), 2.17-2.14 (br t, 1H, *J* = 13.2 Hz, 4-CH), 1.67 (dq, 1H, *J* = 13.2, 2.9 Hz, 9-CH), 1.59 (br d, 1H, *J* = 13.0 Hz, 4-CH), 1.48 (s, 3 x CH_3), 1.27 (t, 3H, *J* = 7.5 Hz, CH_3); ^{13}C-NMR (100 MHz, $CDCl_3$) δ 176.6 (CO), 138.6 (7-CH), 134.2 (6-C), 80.7 (C), 49.8 (1-CH), 48.2 (NCH_2), 43.9 (3-CH_2), 29.0 (9-CH_2), 28.1 (3 x CH_3), 26.7 (4-CH_2), 25.6 (5-CH), 25.0 (8-CH_2), 10.5 (CH_3); υ_{max}/cm^{-1} 2974.1, 1702.1, 1646.3, 1436.9, 1392.5, 1367.4, 1253.6, 1166.9, 1082.6, 758.0; *m/z* HRMS (ESI$^+$) found [M + H]$^+$ 252.1955, $[C_{15}H_{25}NO_2]^+$ requires 252.1964.

(±)-*tert*-Butyl (1*R*)-2-methyl-2-azabicyclo[3.3.1]non-6-ene-6-carboxylate 17h

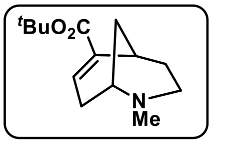

According to general procedure C, (±)-*tert*-butyl (3a*S*,7a*R*)-1-methyl-1,2,3,3a,4,5-hexahydro-7a*H*-indole-7a-carboxylate **16h** (36 mg, 0.15 mmol) in dioxane (0.6 mL) was added to a stirred solution of $Pd(OAc)_2$ (3 mg, 0.02 mmol), DPEPhos (12 mg, 0.02 mmol) and *N*,*N*-diisopropylethylamine (0.03 mL, 0.15 mmol) in dioxane (0.6 mL). After 10 min methanesulfonic acid (0.01 mL, 0.15 mmol) was added and the reaction was heated 100 °C. The crude was purified by SiO_2 flash chromatography, eluting with 0-10% MeOH/CH_2Cl_2 to give a yellow oil (24 mg, 67%). ^{1}H-NMR (400 MHz, $CDCl_3$) δ 7.02 (t, 1H, *J* = 3.7 Hz, 7-CH), 3.12 (br s, 1H, 1-CH), 2.90 (br t, 1H, *J* = 2.9 Hz, 5-CH), 2.65 (dd, 1H, *J* = 12.0, 3.6 Hz, 3-CH), 2.49 (dd, 1H, *J* = 20.9, 3.5 Hz, 8-CH), 2.41 (s, 3H, CH_3) superimposed on 2.34 (br d, 1H, *J* = 12.2 Hz, 3-CH), (2.15 (br d, *J* = 20.6 Hz, 1H, 8-CH), 2.08 (br d, *J* = 12.9 Hz, 1H, 9-CH), 1.97 (tt, 1H, *J* = 13.1, 4.0 Hz, 4-CH), 1.65 (br d, *J* = 12.4 Hz, 1H, 9-CH), 1.54 (br d, *J* = 13.5 Hz, 1H, 4-CH) 1.48 (s, 9H, CH_3); ^{13}C-NMR (100 MHz, $CDCl_3$) δ 165.5, (CO) 139.1 (7-CH), 134.2 (6-C), 80.3 (C), 52.5 (1-CH), 46.1 (3-CH_2), 42.4 (CH_3), 30.9 (9-CH_2), 29.7 (4-CH_2), 28.1 (CH_3), 25.8 (5-CH), 24.3 (8-CH_2); υ_{max}/cm^{-1} 2928.70, 1703.49, 1367.87, 1286.14, 1253.07, 1168.99, 1077.03, 1042.08, 1020.14, 732.46; *m/z* HRMS (ESI$^+$) found [M + H]$^+$ 238.1792, $[C_{14}H_{24}NO_2]^+$ requires 238.1807.

(±)-*tert*-Butyl (1*R*)-2-(naphthalen-1-ylmethyl)-2-azabicyclo[3.3.1]non-6-ene-6-carboxylate 17i

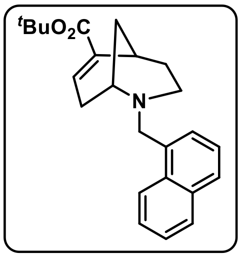

According to general procedure C, (±)-*tert*-butyl (3a*S*,7a*R*)-1-(naphthalen-1-ylmethyl)-1,2,3,3a,4,5-hexahydro-7a*H*-indole-7a-carboxylate **16i** (55 mg, 0.15 mmol) in dioxane (0.6 mL) was added to a stirred solution of $Pd(OAc)_2$ (3 mg, 0.02 mmol), DPEPhos (12 mg, 0.02 mmol) and *N*,*N*-diisopropylethylamine (0.030 mL, 0.15 mmol) in dioxane (0.6 mL). After 10 min methanesulfonic acid (0.01 mL, 0.15 mmol) was added and the reaction was heated under reflux. The crude was purified by SiO_2 flash chromatography, eluting with 0-10% EtOAc/petrol to give a yellow oil (30 mg, 55%). ^{1}H-NMR (400 MHz, $CDCl_3$) δ 8.32 (d, 1H, *J* = 7.9 Hz, ArCH), 7.84 (d, 1H, *J* = 6.8 Hz, ArCH), 7.76 (d, 1H, *J* = 7.4 Hz, ArCH), 7.51-7.47 (m, 2H, ArCH), 7.42-7.37 (m, 2H, ArCH), 7.11 (t, 1H, *J* = 3.5 Hz, 7-CH), 3.98 (d, 2H, *J* = 6.0 Hz, $ArCH_2$), 3.05 (br s, 1H, 1-CH), 2.88 (br s, 1H, 5-CH), 2.60 (br dd, 2H, *J* = 21.0, 3.9 Hz, 3-CH and 8-CH), 2.36 (br t, 1H, *J* =

12.5 Hz, 3-CH), 2.10 (br s, 1H, 8-CH), 1.92 (d, 1H, *J* = 12.9 Hz, 9-CH), 1.86-1.76 (m, 2H, 4-CH_2), 1.57-15.4 (m, 1H, 9-CH), 1.49 (s, 9H, CH_3); ^{13}C-NMR (100 MHz, $CDCl_3$) δ 165.3 (CO), 140.2 (7-CH), 133.9 (6-C), 133.6 (ArC), 133.4 (ArC), 132.7 (ArC), 128.3 (ArCH), 127.7 (ArCH), 126.8 (ArCH), 126.8 (ArCH), 125.6 (ArCH), 125.5 (ArCH), 125.1 (ArCH), 79.9 (C), 57.9 (ArCH_2), 49.8 (1-CH), 44.4 (3-CH_2), 30.9 (9-CH_2), 28.2 (CH_3), 28.0 (4-CH_2), 26.9 (5-CH), 25.0 (8-CH_2); *m/z* HRMS (ESI$^+$) found [M + H]$^+$ 364.2263, [$C_{24}H_{29}NO_2$]$^+$ requires 364.2277; $υ_{max}$/cm^{-1} 2929.73, 1723.36, 1701.78, 1366.64, 1284.91, 1252.04, 1163.89, 1073.52, 791.67, 779.71.

(±)-*tert*-Butyl (1*R*)-2-(pyridine-4-ylmethyl)-2-azabicyclo[3.3.1]non-6-ene-6-carboxylate 17j

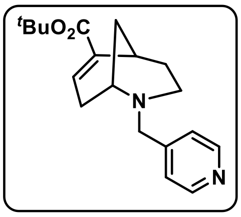

According to general procedure C, (±)-*tert*-butyl (3a*S*,7a*R*)-1-(pyridine-4-ylmethyl)-1,2,3,3a,4,5-hexahydro-7a*H*-indole-7a-carboxylate **16j** (47 mg, 0.15 mmol) in dioxane (0.6 mL) was added to a stirred solution of $Pd(OAc)_2$ (3 mg, 0.02 mmol), DPEPhos (12 mg, 0.02 mmol) and *N*,*N*-diisopropylethylamine (0.030 mL, 0.15 mmol) in dioxane (0.6 mL). After 10 min methanesulfonic acid (0.01 mL, 0.15 mmol) was added and the reaction was heated under reflux. The crude was purified by SiO_2 flash chromatography, eluting with 10-100% EtOAc/petrol to give a yellow oil (23 mg, 49%). ^{1}H-NMR (400 MHz, $CDCl_3$) δ 8.53-8.51 (m, 2H, ArCH), 7.29-7.28 (m, 2H, ArCH), 7.07 (t, 1H, *J* = 3.3 Hz, 7-CH), 3.56 (app q, 2H, ArCH_2), 2.99 (br s, 1H, 1-CH), 2.89 (br t, 1H, *J* = 3.5 Hz, 5-CH), 2.48-2.40 (m, 2H, 3-CH and 8-CH), 2.30 (td, 1H, *J* = 12.8, 3.3 Hz, 3-CH), 2.12-2.05 (m, 1H, 8-CH), 1.98 (dt, 1H, *J* = 12.1, 3.3 Hz, 9-CH), 1.80-1.89 (m, 1H, 4-CH), 1.63-1.54 (m, 2H, 4-CH and 9-CH), 1.48 (s, 9H, CH_3); ^{13}C-NMR (100 MHz, $CDCl_3$) δ 165.8 (CO), 149.8 (ArCH), 139.7 (7-CH), 134.4 (6-CH), 132.0 (ArC), 123.6 (ArCH), 80.0 (C), 58.5 (ArCH_2), 50.5 (1-CH), 44.3 (3-CH_2), 31.8 (9-CH_2), 28.2 (CH_3), 27.9 (4-CH_2), 26.6 (5-CH), 25.3 (8-CH_2); $υ_{max}$/cm^{-1} 2930.06, 1701.92, 1601.36, 1419.50, 1367.10, 1252.60, 1164.74, 1071.44, 1033.00, 800.49; *m/z* HRMS (ESI$^+$) found [M + H]$^+$ 315.2053, [$C_{19}H_{27}N_2O_2$]$^+$ requires 315.2073.

(±)-*tert*-Butyl (1*R*)-2-(4-nitrobenzyl)-2-azabicyclo[3.3.1]non-6-ene-6-carboxylate 17k

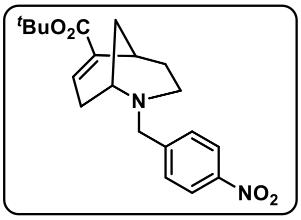

According to general procedure C, (±)-*tert*-butyl (3a*S*,7a*R*)-1-(4-nitrobenzyl)-1,2,3,3a,4,5-hexahydro-7a*H*-indole-7a-carboxylate **16k** (54 mg, 0.15 mmol) in dioxane (0.6 mL) was added to a stirred solution of $Pd(OAc)_2$ (3 mg, 0.02 mmol), DPEPhos (12 mg, 0.02 mmol) and *N*,*N*-diisopropylethylamine (0.030 mL, 0.15 mmol) in dioxane (0.6 mL). After 10 min methanesulfonic acid (0.01 mL, 0.15 mmol) was added and the reaction was heated under reflux fo 26 h. The crude was purified by SiO_2 flash chromatography, eluting with 0-25% EtOAc/petrol to give a yellow oil (23 mg, 43%). ^{1}H-NMR (400 MHz, $CDCl_3$) δ 8.16 (d, 2H, *J* = 8.7 Hz, ArCH), 7.53 (d, 2H, *J* = 8.7 Hz, ArCH), 7.07 (t, 1H, *J* = 3.7 Hz, 7-CH), 3.72-3.58 (m, 2H, ArCH_2), 3.00 (br s, 1H, 1-CH), 2.90 (br s, 1H. 5-CH), 2.48-2.42 (m, 2H, 3-CH and 8-CH), 2.31 (br t, 1H, *J* = 12.3 Hz, 3-CH), 2.14-2.06 (m, 1H , 8-CH), 1.98 (d, 1H, *J* = 11.6 Hz, 9-CH), 1.84 (br t, 1H, *J* = 12.5 Hz, 4-CH), 1.61 (dq, 1H, *J* = 12.2, 2.6 Hz, 9-CH), 1.48 (s, 9H, CH_3) superimposed on (m, 1H, 4-CH); ^{13}C-NMR (100 MHz, $CDCl_3$) δ 165.7 (CO), 147.6 (6-C), 147.1 (7-CH), 139.7 (ArC), 134.4 (ArC), 129.1 (ArCH), 123.5 (ArCH), 80.1 (C), 59.0 (ArCH_2), 50.6 (1-CH), 44.3 (3-CH_2), 31.8 (9-CH_2), 28.8 (4-CH_2), 28.2 (3 x CH_3), 26.6 (5-CH), 25.4 (8-CH_2); $υ_{max}$/cm^{-1} 1699.36, 1518.54, 1343.06, 1366.54, 1284.33, 1251.84, 1155.71, 1084.94, 1073.14, 852.83; *m/z* HRMS (ESI$^+$) found [M + H]$^+$ 359.1958, [$C_{20}H_{27}N_2O_4$]$^+$ requires 224.1651.

(±)-*tert*-Butyl (1*R*)-2-(furan-2-ylmethyl)-2-azabicyclo[3.3.1]non-6-ene-6- carboxylate 17l

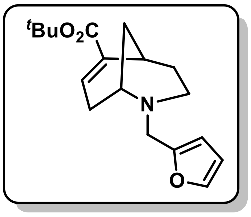

According to general procedure C, (±)-*tert*-butyl (3a*S*,7a*R*)-1-(furan-2-ylmethyl)-1,2,3,3a,4,5-hexahydro-7a*H*-indole-7a-carboxylate **16l** (46.0 mg, 0.15 mmol) in dioxane (0.6 mL) was added to a stirred solution of $Pd(OAc)_2$, DPEPhos and *N*,*N*-diisopropylethylamine in dioxane (0.6 mL). After 10 min methanesulfonic acid was added and the reaction was heated under reflux. The crude was purified by SiO_2 flash chromatography, eluting with 10-20% EtOAc/isohexane to give a yellow oil (19.0 mg, 41%). ^{1}H-NMR (400 MHz, $CDCl_3$) δ ^{13}C-NMR (100 MHz, $CDCl_3$) δ 7.37-7.37 (m, 1H, ArCH), 7.05 (t, 1H, *J* = 3.8 Hz, 7-CH), 6.30-6.29 (m, 1H, ArCH), 6.19-6.18 (m, 1H, ArCH), 3.58 (s, 2H, ArCH_2), 2.99-2.98 (m, 1H, 1-CH), 2.87 (br s, 1H, 5-CH), 2.60-2.56 (m, 1H, 3-CH), 2.43 (dd, 1H, *J* = 20.7, 4.3 Hz, 8-CH), 2.27 (td, 1H, *J* = 12.8, 3.5 Hz, 3-CH), 2.07-2.03 (m, 1H, 8-CH), 1.97 (br d, 1H, *J* = 13.0 Hz, 9-CH), 1.89 (tt, 1H, *J* = 12.6, 4.5 Hz, 4-CH), 1.58 (dq, 1H, *J* = 12.1, 3.0 Hz, 9-CH), 1.48 (s, 9 H, 3 x CH_3), superimposed on 1.46-1.45 (m, 1H, 4-CH); ^{13}C-NMR (100 MHz, $CDCl_3$) δ 165.8 (CO), 152.4 (ArC), 142.1 (ArCH), 139.9 (7-CH), 134.4 (6-C), 110.0 (ArCH), 108.3 (ArCH), 79.9 (C), 51.9 (ArCH_2), 49.6 (1-CH), 44.5 (3-CH_2), 31.6 (9-CH_2), 28.6 (4-CH_2), 28.2 (3 x CH_3), 26.7 (5-CH), 24.7 (8-CH_2); $υ_{max}$/cm^{-1} 2927.7, 1702.3, 1366.6, 1285.2, 1251.4, 1167.6, 1149.6, 1124.2, 1072.1, 731.6; *m/z* HRMS (ESI$^+$) found [M + H]$^+$ 304.1906, [$C_{18}H_{26}NO_3$]$^+$ requires 304.1913.

(±)-*tert*-Butyl (1*R*)-2-benzyl-2-azabicyclo[3.3.1]non-6-ene-6-carboxylate 17m

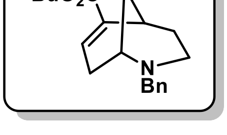

According to general procedure C, (±)-*tert*-butyl (3a*S*,7a*R*)-1-benzyl-2-1,2,3,3a,4,5-hexahydro-7a*H*-indole-7a-carboxylate **16m** (47.0 mg, 0.15 mmol) in dioxane (0.6 mL) was added to a stirred solution of $Pd(OAc)_2$, DPEPhos and *N*,*N*-diisopropylethylamine in dioxane (0.6 mL). After 10 min methanesulfonic acid was added and the reaction was heated under reflux. The crude was purified by SiO_2 flash chromatography, eluting with 10-20% EtOAc/Isohexane to give a colourless oil (18.0 mg, 38%). ^{1}H-NMR (400 MHz, $CDCl_3$)

7.35-7.29 (m, 4H, ArCH), 7.24-7.22 (m, 1H, ArCH), 7.08 (t, 1H, *J* = 3.6 Hz, 7-CH), 3.56 (q, 2H, *J* = 15.5 Hz, $ArCH_2$), 3.00 (br s, 1H, 1-CH), 2.88 (br s, 1H, 5-CH), 2.54-2.46 (m, 2H, 3-CH and 8-CH), 2.27 (td, 1H, *J* = 12.0, 3.6 Hz, 3-CH), 2.06-2.00 (m, 1H, 8-CH), 1.95 (br d, 1H, *J* = 12.0 Hz, 9-CH), 1.84 (tt, 1H, *J* = 12.8, 4.5 Hz, 4-CH), 1.59-1.57 (m, 1H, 9-CH), 1.48 (s, 9H, 3 x CH_3) 1.47-1.46 (m, 1H, 4-CH); ^{13}C-NMR (100 MHz, $CDCl_3$) δ 165.9 (CO), 140.2 (7-CH), 139.4 (ArC), 134.4 (6-C), 128.8 (ArCH), 128.2 (ArCH), 126.8 (ArCH), 79.9 (C), 59.6 ($ArCH_2$), 49.8 (1-CH), 44.3 (3-CH_2), 31.9 (9-CH_2), 28.9 (4-CH_2), 28.2 (3 x CH_3), 26.9 (5-CH), 24.9 (8-CH_2); υ_{max}/cm^{-1} 2930.4, 1701.9, 1366.5, 1284.2, 1251.3, 1166.9, 1135.8, 1070.2, 1024.8, 697.9; *m/z* HRMS (ESI$^+$) found [M + H]$^+$ 314.2109, $[C_{20}H_{28}NO_2]^+$ requires 314.2120.

(±)-*tert*-Butyl (1*R*)-2-(pyridine-2-ylmethyl)-2-azabicyclo[3.3.1]non-6-ene-6-carboxylate 17n

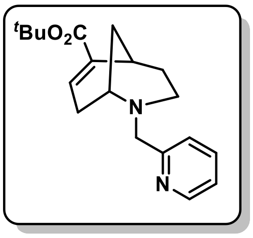

According to general procedure C, (±)-*tert*-butyl (3a*S*,7a*R*)-1-(pyridine-2-ylmethyl)-1,2,3,3a,4,5-hexahydro-7a*H*-indole-7a-carboxylate **16n** (47.0 mg, 0.15 mmol) in dioxane (0.6 mL) was added to a stirred solution of $Pd(OAc)_2$, DPEPhos and *N*,*N*-diisopropylethylamine in dioxane (0.6 mL). After 10 min methanesulfonic acid was added and the reaction was heated under reflux. The crude was purified by SiO_2 flash chromatography, eluting with 10-30% EtOAc/Isohexane to give a yellow oil (16.0 mg, 34%). ^{1}H-NMR (400 MHz, $CDCl_3$) δ 8.52 (d, 1H, *J* = 5.3 Hz, ArCH), 7.64 (t, 1H, *J* = 7.8 Hz, ArCH), 7.48 (d, 1H, *J* = 7.8 Hz, ArCH), 7.13 (t, 1H, *J* = 5.3 Hz, ArCH), 7.08 (t, 1H, *J* = 3.6 Hz, 7-CH), 3.72 (dd, 1H, *J* = 45.1, 14.2 Hz, $ArCH_2$), 3.02-3.01 (m, 1H, 1-CH), 2.89 (br t, 1H, *J* = 2.9 Hz, 5-CH), 2.54-2.50 (m, 2H, 3-CH and 8-CH), 2.36 (td, 1H, *J* = 12.2, 3.5 Hz, 3-CH), 2.08 (br d, 1H, *J* = 20.3 Hz, 8-CH), 2.00 (t, 1H, *J* = 12.2 Hz, 9-CH), 1.87 (tt, 1H, *J* = 12.5, 4.1 Hz, 4-CH), 1.60-1.51 (m, 2H, 4-CH and 9-CH), 1.48 (s, 9H, 3 x CH_3); ^{13}C-NMR (100 MHz, $CDCl_3$) δ 165.9 (CO), 159.8 (C), 149.0 (ArCH), 140.2 (7-CH), 136.4 (ArCH), 134.3 (6-C), 122.7 (ArCH), 121.8 (ArCH), 79.9 (C), 61.5 ($ArCH_2$), 50.5 (1-CH), 44.5 (3-CH_2), 31.9 (9-CH_2), 28.9 (4-CH_2), 28.2 (3 x CH_3), 26.7 (5-CH), 25.5 (8-CH_2); υ_{max}/cm^{-1} 2928.6, 1702.0, 1473.6, 1432.5, 1366.9, 1284.9, 1251.8, 1166.9, 1072.7, 755.7; *m/z* HRMS (ESI$^+$) found [M + H]$^+$ 315.2060, $[C_{19}H_{27}N_2O_2]^+$ requires 315.2073.

(±)-*tert*-Butyl (1*R*)-2-(thiophen-2-ylmethyl)-2-azabicyclo[3.3.1]non-6-ene-6- carboxylate 17o

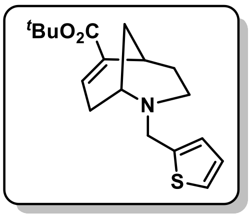

According to general procedure C, (±)-*tert*-butyl (3a*S*,7a*R*)-1-(thiophen-2-ylmethyl)-1,2,3,3a,4,5-hexahydro-7a*H*-indole-7a-carboxylate **16o** (48.0 mg, 0.15 mmol) in dioxane (0.6 mL) was added to a stirred solution of $Pd(OAc)_2$, DPEPhos and *N*,*N*-diisopropylethylamine in dioxane (0.6 mL). After 10 min methanesulfonic acid was added and the reaction was heated under reflux. The crude was purified by SiO_2 flash chromatography, eluting with 0-20% EtOAc/Isohexane to give a colourless oil (12.0 mg, 12%). ^{1}H-NMR (400 MHz, $CDCl_3$) δ ^{13}C-NMR (100 MHz, $CDCl_3$) δ 7.21-7.20 (m, 1H, ArCH), 7.06 (t, 1H, *J* = 3.6 Hz, 7-CH), 6.93-6.91 (m, 2H, ArCH), 3.76 (d, 2H, *J* = 6.5 Hz, $ArCH_2$), 3.08 (br s, 1H, 1-CH), 2.88 (br s, 1H, 5-CH), 2.74-2.60 (m, 2H, 3-CH and 8-CH), 2.41 (br dd, 1H, *J* = 20.8, 4.2 Hz, 8-CH), 2.28 (br t, 1H, *J* = 12.1 Hz, 3-CH), 2.09-2.02 (m, 1H, 8-CH), 1.94 (br d, 1H, *J* = 12.3 Hz, 9-CH), 1.90-1.81 (m, 1H, 4-CH), 1.59-1.55 (m, 2H, 4-CH and 9-CH), 1.48 (s, 9 H, 3 x CH_3); ^{13}C-NMR (100 MHz, $CDCl_3$) δ 165.9 (CO), 143.9 (ArC), 140.0 (7-CH), 134.4 (6-C), 126.3 (ArCH), 125.0 (ArCH), 124.7 (ArCH), 79.9 (C), 54.1 ($ArCH_2$), 49.6 (1-CH), 44.3 (3-CH_2), 31.7 (9-CH_2), 28.2 (3 x CH_3), 27.9 (4-CH_2), 26.8 (5-CH), 25.0 (8-CH_2); υ_{max}/cm^{-1} 2927.2, 1724.7, 1702.8, 1366.7, 1285.1, 1251.7, 1164.0, 1073.3, 731.7, 696.3; *m/z* HRMS (ESI$^+$) found [M + H]$^+$ 320.1672, $[C_{18}H_{26}NO_2S]^+$ requires 320.1684.

(±)-*tert*-Butyl (1*R*)-2-(2-hydroxybenzyl)-2-azabicyclo[3.3.1]non-6-ene-6-carboxylate 17p

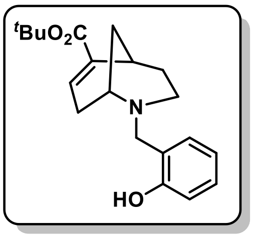

According to general procedure C, (±)-*tert*-butyl (3a*S*,7a*R*)-1-(2-hyroxybenzyl)-1,2,3,3a,4,5-hexahydro-7a*H*-indole-7a-carboxylate **16p** (49.0 mg, 0.15 mmol) in dioxane (0.6 mL) was added to a stirred solution of $Pd(OAc)_2$, DPEPhos and *N*,*N*-diisopropylethylamine in dioxane (0.6 mL). After 10 min methanesulfonic acid was added and the reaction was heated under reflux. The crude was purified by SiO_2 flash chromatography, eluting with 10-20% EtOAc/Isohexane to give a yellow oil (13.0 mg, 27%). ^{1}H-NMR (400 MHz, $CDCl_3$) δ 7.16 (t, 1H, *J* = 7.6 Hz, ArCH), 7.05 (t, 1H, *J* = 3.5 Hz, 7-CH), 6.95 (d, 1H, *J* = 7.6 Hz, CH), 6.80 (d, 1H, *J* = 7.6 Hz, ArCH), 6.75 (t, 1H, *J* = 7.6 Hz, ArCH), 3.77 (dd, 2H, *J* = 37.7, 14.3 Hz, $ArCH_2$), 3.17 (br s, 1H, 1-CH), 2.94 (br t, 1H, *J* = 3.0 Hz, 5-CH), 2.68 (dd, 1H, *J* = 12.5, 4.3 Hz, 3-CH), 2.46 (dd, 1H, *J* = 20.9, 4.4 Hz, 8-CH), 2.36 (br t, 1H, *J* = 12.8 Hz, 3-CH), 2.15 (br d, 1H, *J* = 20.6 Hz, 8-CH), 1.97 (br d, 1H, *J* = 12.6 Hz, 9-CH), 1.89 (tt, 1H, *J* = 12.8, 4.5 Hz, 4-CH) 1.67 (br d, 1H, *J* = 12.6 Hz, 9-CH),1.58-1.51 (m, 1H, 4-CH), 1.48 (s, 9H, 3 x CH_3); ^{13}C-NMR (100 MHz, $CDCl_3$) δ 165.5 (CO), 158.4 (ArC), 139.1 (7-CH), 134.4 (6-C), 128.7 (ArCH), 128.5 (ArCH), 121.2 (ArC), 118.9 (ArCH), 116.0 (ArCH), 80.3 (C), 58.4 ($ArCH_2$), 49.6 (1-CH_2), 43.7 (3-CH_2), 31.7 (9-CH_2), 28.6 (4-CH_2), 28.2 (3 x CH_3), 26.5 (5-CH), 24.6 (8-CH_2); υ_{max}/cm^{-1} 2930.2, 1703.6, 1477.8, 1367.2, 1255.7, 1167.5, 1103.5, 1073.0, 102.4, 753.7; *m/z* HRMS (ESI$^+$) found [M + H]$^+$ 330.2056 $[C_{20}H_{28}NO_3]^+$ requires 330.2069.

(±)-*tert*-Butyl (1*R*)-2-(4-fluorobenzyl)-2-azabicyclo[3.3.1]non-6-ene-6-carboxylate 17q

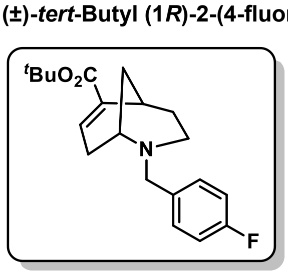

According to general procedure C, (±)-*tert*-butyl (3a*S*,7a*R*)-1-(4- fluorobenzyl) - 1, 2, 3, 3a, 4, 5 – hexahydro - 7a*H* – indole - 7a -carboxylate **16q** (52.0 mg, 0.15 mmol) in dioxane (0.6 mL) was added to a stirred solution of $Pd(OAc)_2$, DPEPhos and *N*,*N*-diisopropylethylamine in dioxane (0.6 mL). After

10 min methanesulfonic acid was added and the reaction was heated under reflux. The crude was purified by SiO_2 flash chromatography, eluting with 5-20% EtOAc/Isohexane to give a colourless oil (11.0 mg, 21%). ^{1}H-NMR (400 MHz, $CDCl_3$) δ 7.31-7.28 (m, 2H, ArCH), 7.07 (t, 1H, *J* = 3.7 Hz, 7-CH), 7.00-6.96 (m, 2H, ArCH), 3.51 (q, 2H, *J* = 20.6 Hz, $ArCH_2$), 2.98 (m, 1H, 1-CH), 2.88 (br t, *J* = 3.2 Hz, 5-CH), 2.49 (m, 2H, 3-CH and 8-CH), 2.25 (td, 1H, *J* = 12.3, 3.4 Hz, 3-CH), 2.07-2.01 (m, 1H, 8-CH), 1.93 (br d, 1H, *J* = 11.6 Hz, 9-CH), 1.82 (tt, 1H, *J* = 12.9, 4.2 Hz, 4-CH), 1.60-1.50 (m, 2H, 4-CH and 9-CH), 1.48 (s, 9H, 3 x CH_3); ^{13}C-NMR (100 MHz, $CDCl_3$) δ 165.9 (CO), 161.9 (d, *J* = 2.44.5 Hz, ArCF), 140.0 (7-CH), 134.9 (ArC), 134.4 (6-C), 130.1 (d, *J* = 7.7 Hz, ArCH), 115.0 (d, *J* = 22.2 Hz, ArCH), 79.9 (C), 58.8 ($ArCH_2$), 49.8 (1-CH), 44.2 (3-CH_2), 31.9 (9-CH_2), 28.9 (3 x CH_3), 28.2 (4-CH_2), 26.8 (5-CH), 25.0 (8-CH_2); ^{19}F{^{1}H}-NMR (377 MHz, $CDCl_3$) δ -116.19; υ_{max}/cm^{-1} 1702.3, 1508.7, 1366.8, 1285.1, 1252.2, 1220.6, 1166.5, 1072.6, 832.0, 760.1; *m/z* HRMS (ESI$^+$) found [M + H]$^+$ 332.2014, $[C_{20}H_{27}FNO_2]^+$ requires 332.2026.

(±)-*tert*-Butyl (1*R*)-2-((1-methyl-1H-pyrrol-2-yl)methyl)-2-azabicyclo[3.3.1]non-6-ene-carboxylate 17r

According to general procedure C, (±)-*tert*-butyl (3a*S*,7a*R*)-1-((1-methyl-1*H*-pyrol-2-yl)methyl)-1,2,3,3a,4,5-hexahydro-7a*H*-indole-7a-carboxylate **16r** (47.0 mg, 0.15 mmol) in dioxane (0.6 mL) was added to a stirred solution of $Pd(OAc)_2$, DPEPhos and *N*,*N*-diisopropylethylamine in dioxane (0.6 mL). After 10 min methanesulfonic acid was added and the reaction was heated under reflux. The crude was purified by SiO_2 flash chromatography, eluting with 10-30% EtOAc/Isohexane to give a yellow oil (9.0 mg, 19%). ^{1}H-NMR (400 MHz, $CDCl_3$) δ 7.06 (br t, 1H, *J* = 3.6 Hz, 7-CH), 6.58 (br s, 1H, ArCH), 6.01-5.95 (m, 2H, ArCH), 3.65 (s, 3H, CH_3), 3.49 (s, 2H, $ArCH_2$), 2.90-2.86 (m, 2H, 1-CH and 5-CH), 2.53-2.40 (m, 2H, 3-CH and 8-CH), 2.25-2.18 (m, 1H, 3-CH), 2.05-1.97 (m, 1H, 8-CH), 1.85-1.72 (m, 2H, 4-CH and 9-CH), 1.56-1.54 (m, 2H, 4-CH and 9-CH), 1.48 (s, 9H, 3 x CH_3); ^{13}C-NMR (100 MHz, $CDCl_3$) δ 165.9 (CO), 140.3 (7-CH), 134.3 (6-C), 129.4 (ArC), 122.4 (ArCH), 108.9 (ArCH), 106.0 (ArCH), 79.9 (C), 51.2 ($ArCH_2$), 48.9 (1-CH), 44.0 (3-CH_2), 33.8 (CH_3), 32.0 (9-CH_2), 29.0 (4-CH_2), 28.2 (3 x CH_3), 27.0 (5-CH), 24.5 (8-CH_2); υ_{max}/cm^{-1} 2927.0, 1702.8, 1454.9, 1366.8, 1251.7, 1168.2, 1072.4, 1024.7, 912.0, 708.0; *m/z* HRMS (ESI$^+$) found [M + H]$^+$ 317.2223 $[C_{19}H_{29}N_2O_2]^+$ requires 317.2229.

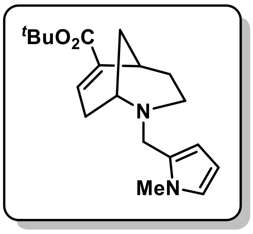

(±)-*tert*-Butyl (1*R*)-2-(benzofuran-2-ylmethyl)-2-azabicyclo[3.3.1]non-6-ene-6-carboxylate 17s

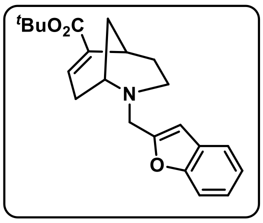

According to general procedure C, (±)-*tert*-butyl (3a*S*,7a*R*)-1-(benzofuran-2-ylmethyl)-1,2,3,3a,4,5-hexahydro-7a*H*-indole-7a-carboxylate **16s** (53 mg, 0.15 mmol) in dioxane (0.6 mL) was added to a stirred solution of $Pd(OAc)_2$ (3 mg, 0.02 mmol), DPEPhos (12 mg, 0.02 mmol) and *N*,*N*-diisopropylethylamine (0.030 mL, 0.15 mmol) in dioxane (0.6 mL). After 10 min methanesulfonic acid (0.01 mL, 0.15 mmol) was added and the reaction was heated under reflux. The crude was purified by SiO_2 flash chromatography, eluting with 0-30% EtOAc/petrol to give a yellow oil (10 mg, 19%). ^{1}H-NMR (400 MHz, $CDCl_3$) δ 7.52-7.46 (m, 2H, ArCH), 7.24-7.17 (m, 2H, ArCH), 7.07 (t, 1H, *J* = 3.5 Hz, 7-CH), 6.59 (s, 1H, ArCH), 3.74 (s, 2H, $ArCH_2$), 3.08 (br s, 1H, 1-CH), 2.90 (br t, 1H, *J* = 3.0 Hz, 5-CH), 2.69-2.63 (m, 1H, 3-CH), 2.48 (dd, 1H, *J* = 20.7, 4.0 Hz, 8-CH), 2.36 (td, 1H, *J* = 12.0, 3.5 Hz, 3-CH), 2.12-2.04 (m, 1H, 8-CH), 2.01 (td, 1H, *J* = 12.3, 3.0 Hz, 9-CH), 1.93 (tt, 1H, *J* = 12.6, 4.1 Hz, 4-CH), 1.62-1.57 (m, 2H, 4-CH and 9-CH), 1.48 (s, 9H, CH_3); ^{13}C-NMR (100 MHz, $CDCl_3$) δ 165.8 (CO), 155.5 (ArC), 155.1 (ArC), 139.8 (7-CH), 134.4 (6-CH), 128.4 (ArC), 123.8 (ArCH), 122.6 (ArCH), 120.6 (ArCH), 111.3 (ArCH), 105.1 (ArCH), 80.0 (C), 52.5 ($ArCH_2$), 49.8 (1-CH), 44.7 (3-CH), 31.6 (9-CH_2), 28.6 (4-CH_2), 28.2 (CH_3), 26.7 (5-CH), 24.8 (8-CH_2); υ_{max}/cm^{-1} 2928.52, 1701.39, 1454.33, 1366.78, 1284.15, 1252.74, 1165.75, 1072.62, 1025.26, 750.88; *m/z* HRMS (ESI$^+$) found [M + H]$^+$ 354.2058, $[C_{22}H_{28}NO_3]^+$ requires 354.2069.

Intermediate Trapping

***tert*-Butyl 6-(2-(*N*-(4-methoxybenzyl)acetamido)ethyl)cyclohexa-1,3-diene-1-caboxylate 20**

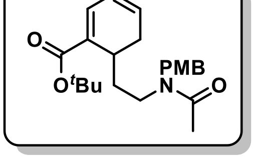

N,*N*-diisopropylethylamine (0.020 mL, 0.13 mmol) in dioxane (0.6 mL) was added to $Pd(OAc)_2$ (3.0 mg, 0.01 mmol) and DPEPhos (9.0 mg, 0.02 mmol) and stirred for 10 min. The reaction mixture turned bright yellow. (±)-*tert*-Butyl (3a*S*,7a*R*)- 1- (4-methoxybenzyl) - 1, 2, 3, 3a, 4, 5-hexahydro-7a*H*-indole-7a-carboxylate **12** (44.0 mg, 0.13 mmol) in dioxane (0.6 mL), camphorsulfonic acid (30.0 mg, 0.13 mmol) and acetic anhydride (0.01 mL, 0.13mmol) were added sequentially and the reaction mixture was heated to reflux. After 17 h, the reaction was cooled to RT, quenched with $NaHCO_3$ (saturated aqueous solution, 5 mL) and extracted with CH_2Cl_2 (3 x 5 mL). The combined organic layers were dried over $MgSO_4$, filtered and concentrated *in vacuo*. The crude was purified by SiO_2 flash chromatography, 10-30% EtOAc/Petrol, to give a yellow oil (41.0 mg, 82%). ^{1}H-NMR (400 MHz, $CDCl_3$) δ 7.16-7.06 (m, 2H, ArCH), 6.93-6.89 (m, 1H, CH), 6.88-6.81 (m, 2H, ArCH), 6.05-5.95 (m, 2H, 2 x CH), 4.57-4.42 (m, 2H, $ArCH_2$), 3.78 (s, 3H, OCH_3), 3.49-3.37 (m, 1H, 3-CH), 3.31-3.05 (m, 2H, 1-CH_2), 2.67-2.58 (m, 1H, 2-CH), 2.49-2.38 (m, 1 H, 2-CH), 2.19-2.13 (m, 1H, 4-CH), 2.11 (s, 3H, CH_3), 1.65-1.60 (m, 1H, 4-CH), 1.46 (s, 9H, CH_3); ^{13}C-NMR (100 MHz, $CDCl_3$) δ 170.2 (CO), 166.5 (CO), 158.9 (C), 132.1 (7-CH), 132.0 (ArC), 130.9 (CH), 129.5 (ArCH), 128.7 (ArC), 123.8 (CH), 113.9 (ArCH), 80.3 (C), 55.2 (OCH_3), 50.8 ($ArCH_2$), 45.4 (1-CH_2), 42.6 (3-CH), 28.2 (4-CH_2), 28.1 (3 x CH_3), 27.4 (2-CH_2), 21.4 ($COCH_3$); υ_{max}/cm^{-1} 2931.88, 1696.54, 1645.23, 1512.89, 141.96, 1366.83, 1279.59, 1247.00, 1161.79, 1033.74; *m/z* HRMS (ESI$^+$) found [M + H]$^+$ 386.2329 and [M + Na]$^+$ 408.2146, $[C_{23}H_{32}NO_4]^+$ requires 386.2331 and $[C_{23}H_{31}NO_4Na]^+$ requires 408.2151.

(±)-*tert*-Butyl (1*S*,6*S*)-2-(4-methoxybenzyl)-2-azabicyclo[3.3.1]non-7-ene-6-carboxylate 21

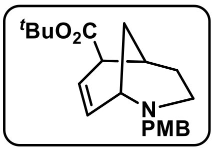

(±)-*tert*-Butyl (3a*S*,7a*R*)- 1- (4-methoxybenzyl) - 1, 2, 3, 3a, 4, 5-hexahydro-7a*H*-indole-7a-carboxylate **12** (104 mg, 0.30 mmol) in dioxane (1.2 mL) was added to a stirred solution of *N*,*N*-diisopropylethylamine (0.060 mL, 0.30 mmol), $Pd(OAc)_2$ (6 mg, 0.03 mmol) and DPEPhos (24 mg, 0.05 mmol) in dioxane (1.2 mL). The reaction mixture was stirred for 10 min, methanesulfonic acid (0.02 mL, 0.30 mmol) was added and the reaction was heated under reflux. After 3 h, the reaction mixture was cooled to RT, quenched with $NaHCO_3$ (saturated aqueous solution, 5 mL) and extracted with CH_2Cl_2 (3 x 5 mL). The combined organic layers were dried over $MgSO_4$, filtered and concentrated *in vacuo*. The crude material was purified by SiO_2 flash chromatography, 5-50% EtOAc/petrol, to give the conjugated morphan product (29 mg, 28%) and the unconjugated morphan intermediate (16 mg, 15%) as a 5:4 inseparable mixture of diastereoisomers. ^{1}H-NMR (400 MHz, $CDCl_3$) δ 7.28-7.25 (m, 2H, ArCH), 6.85 (d, 2H, *J* = 8.9 Hz, ArCH), 6.22-6.13 (m, 1H, 7-CH), 5.87-5.72 (m, 1H, 8-CH), 3.79 (s, 3H, OCH_3), 3.51-3.47 (m, 1H, $ArCH_2$), 3.36 (app t, 1H, $ArCH_2$), 3.24-3.17 (m, 1H, 1-CH), 2.74 (app t, 1H, 6-CH), 2.58-2.52 (m, 1H, 3-CH), 2.45-2.25 (m, 2H, 3-CH and 4-CH), 2.02-1.71 (m, 3H, 5-CH and 9-CH_2), 1.59-1.53 (m, 1H, 4-CH), 1.44 (s, 9H, CH_3); ^{13}C-NMR (106 MHz, $CDCl_3$) δ 172.5 (CO), 158.7 (ArC), 132.0 (ArC), 130.2 (7-CH), 130.1 (ArCH), 125.2 (8-CH), 113.6 (ArCH), 80.5 (C), 59.5 ($ArCH_2$), 55.2 (OCH_3), 49.5 (1-CH), 48.1 (6-CH), 44.9 (3-CH_2), 32.3 (9-CH_2), 29.3 (5-CH), 28.0 (CH_3), 27.8 (4-CH_2); υ_{max}/cm^{-1} 2929.43, 1724.69, 1512.21, 1367.28, 1301.73, 1244.31, 1151.36, 1105.27, 1037.31, 835.80; *m/z* HRMS (ESI$^+$) found [M + H]$^+$ 344.2234, $[C_{21}H_{30}NO_3]^+$ requires 344.2226.

Subjecting (±)-*tert*-butyl (1*S*,6*S*)-2-(4-methoxybenzyl)-2-azabicyclo[3.3.1]non-7-ene-6-carboxylate 21 to Standard Conditions

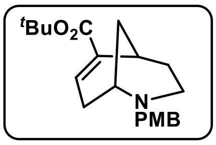

(±)-*tert*-Butyl (1*S*,6*S*)-2-(4-methoxybenzyl)-2-azabicyclo[3.3.1]non-7-ene-6-carboxylate **21** (29 mg, 0.08 mmol) in dioxane (0.35 mL) was added to a stirred solution of *N*,*N*-diisopropylethylamine (0.015 mL, 0.08 mmol), $Pd(OAc)_2$ (1 mg, 0.008 mmol) and DPEPhos (7 mg, 0.01 mmol) in dioxane (0.35 mL). The reaction mixture was stirred for 10 min, methanesulfonic acid (0.005 mL, 0.08 mmol) was added and the reaction was heated under reflux. After 20 min, the reaction mixture was cooled to RT, quenched with $NaHCO_3$ (saturated aqueous solution, 5 mL) and extracted with CH_2Cl_2 (3 x 5 mL). The combined organic layers were dried over $MgSO_4$, filtered and concentrated *in vacuo*, to give crude (±)-*tert*-butyl-(1*R*)-2-(4-methoxybenzyl)-2-azabicyclo[3.3.1]non-6-ene-6-carboxylate (24% based on ^{1}H-NMR) and unreacted (±)-*tert*-butyl-(1*S*,6*S*)-2-(4-methoxybenzyl)-2-azabicyclo[3.3.1]non-7-ene-6-carboxylate (76% based on ^{1}H-NMR).

Subjecting (±)-*tert*-butyl (1*S*,6*S*)-2-(4-methoxybenzyl)-2-azabicyclo[3.3.1]non-7-ene-6-carboxylate 21 to Acid and Base

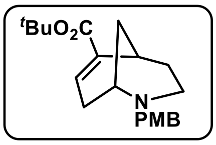

N,*N*-diisopropylethylamine (0.01 mL, 0.05 mmol) was added to a stirred solution of (±)-*tert*-butyl (1*S*,6*S*)-2-(4-methoxybenzyl)-2-azabicyclo[3.3.1]non-7-ene-6-carboxylate **21** (16 mg, 0.05 mmol) in dioxane (0.4 mL). The reaction mixture was stirred for 10 min, methanesulfonic acid (0.003 mL, 0.05 mmol) was added and the reaction was heated under reflux. After 20 h, the reaction mixture was cooled to RT, quenched with $NaHCO_3$ (saturated aqueous solution, 5 mL) and extracted with CH_2Cl_2 (3 x 5 mL). The combined organic layers were dried over $MgSO_4$, filtered and concentrated *in vacuo*, to give crude (±)-tert-butyl-(1*R*)-2-(4-methoxybenzyl)-2-azabicyclo[3.3.1]non-6-ene-6-carboxylate (quant. based on ^{1}H-NMR).

Synthesis of Deuterated Materials

1-(Phenylsulfonyl)-1*H*-pyrrole S32

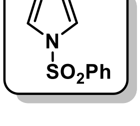

Pyrrole (8.0 mL, 0.12 mol) was added dropwise over 20 min to a stirred solution of NaH (60% in mineral oil, 5.60 g, 0.14 mol) in DMF (100 mL) at 0 ºC. The reaction was warmed to RT, stirred for 5 min then cooled back to 0 ºC, at which point benzenesulfonyl chloride (15.50 mL, 0.12 mol) was added dropwise over 8 min. The reaction was warmed to RT and stirred for 17 h, quenched with H_2O (200 mL) and extracted with EtOAc (3 x 200 mL). The combined organic layers were washed with brine, dried over $MgSO_4$, filtered and concentrated i*n vacuo*. The crude product was purified by SiO_2, eluting with 0-20% EtOAc/petrol, to give a yellow solid. This was further purified by trituration (5% Et_2O/hexane, 100 mL) to give the product as a yellow solid (15.28 g, 61%). ^{1}H-NMR (400 MHz, $CDCl_3$) δ 7.87-7.84 (m, 2H, ArCH), 7.60 (app tt, 1H, ArCH), 7.52-7.48 (m, 2H, ArCH), 7.17 (t, 2H, *J* = 2.3 Hz, NCH), 6.30 (t, 2H, *J* = 2.3 Hz, NCHC<u>H</u>); ^{13}C-NMR (100 MHz, $CDCl_3$) δ 139.1 (ArC), 133.8 (ArCH), 129.4 (ArCH), 126.8 (ArCH), 120.8 (NCH), 113.7 (NCH<u>C</u>H); m.p. ˚C (EtOAc/petrol) 88-89 ˚C, lit. 88.5-89.3 ˚C.[15] ^{1}H-NMR and ^{13}C-NMR data are consistent with the literature.[15]

3-Bromo-1-(Phenylsulfonyl)-1*H*-pyrrole S33

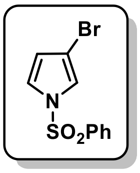

A solution of bromine (3.00 mL, 57.9 mmol) in acetic acid (20 mL) was added to a stirred solution of 1-(phenylsulfonyl)-1*H*-pyrrole **S32** (10.0 g, 48.3 mmol) in acetic acid (120 mL)and the reaction mixture was heated

under reflux. After 2 h the reaction was cooled to RT, concentrated *in vacuo* and dissolved in EtOAc (100 mL) and $Na_2S_2O_3$ (1M aqueous solution, 50 mL). The organic layer was extracted with EtOAc (2 x 50 mL), and the combined organic layers were dried over $MgSO_4$, filtered and concentrated i*n vacuo*. The crude product was purified by SiO_2, eluting with 0-5% EtOAc/petrol, to give a pink solid (9.71 g, 70%). ^{1}H-NMR (400 MHz, $CDCl_3$) δ 7.87-7.86 (d, 2H, *J* = 7.6 Hz, ArCH), 7.63 (t, 1H, *J* = 7.5 Hz, ArCH), 7.57-7.50 (m, 2H, ArCH), 7.20-7.17 (m, 1H, NCHCBr), 7.09 (t, 2H, *J* = 2.7 Hz, NCH), 6.30 (br s, 1H, NCHCH); ^{13}C-NMR (100 MHz, $CDCl_3$) δ 138.4 (ArC), 134.2 (ArCH), 129.6 (ArCH), 127.0 (ArCH), 121.3 (NCH), 119.7 (NCHCBr), 116.4 (NCHCH) 102.2 (CBr); m.p. 73-75 ˚C (EtOAc/petrol), lit. 66.5-67 ˚C.[16] ^{1}H-NMR and ^{13}C-NMR data are consistent with the literature.[16]

Methyl 3-bromo-1-(phenylsulfonyl)-1*H*-pyrrole-2-carboxylate S34

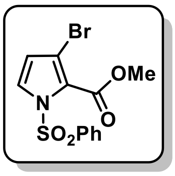

n-BuLi (2.5 M solution in hexanes, 16.40 mL, 41.0 mmol) was added dropwise over 10 min to a stirred solution of diisopropylamine (7.20 mL, 51.26 mmol) in THF (120 mL) at -78 ºC. The reaction mixture was stirred for 15 min, warmed to 0 ºC for 5 min then cooled to -78 ºC, at which point 3-bromo-1-(phenylsulfonyl)-1H-pyrrole **S32** (9.71 g, 34.17 mmol) in THF (33 mL) was added dropwise over 10 min. The reaction mixture was stirred for 1 h at -78 ºC and methyl chloroformate (5.80 mL, 75.17 mmol) in THF (33 mL) was added dropwise over 10 min. After stirring for a further hour, the reaction mixture was warmed to RT, quenched with NH_4Cl (saturated aqueous solution, 300 mL) and extracted with EtOAc (3 x 200 mL). The combined organic layers were dried over $MgSO_4$, filtered and concentrated i*n vacuo*. The crude product was purified by SiO_2, eluting with 0-20% EtOAc/petrol, to give an orange liquid (9.34 g, 79%). ^{1}H-NMR (400 MHz, $CDCl_3$) δ 7.96-7.94 (m, 2H, ArCH), 7.68-7.63 (m, 1H, ArCH), 7.58-7.54 (m, 3H, ArCH and NCH), 6.41 (d, 1H, *J* = 3.4 Hz, NCHCH), 3.80 (s, 3H, CH_3); ^{13}C-NMR (100 MHz, $CDCl_3$) δ 159.3 (CO), 138.7 (ArC), 134.1 (ArCH), 129.0 (ArCH), 127.9 (ArCH), 126.8 (NCH), 115.3 (NCHCH), 109.7 (CBr), 52.1 (CH_3). ^{1}H-NMR and ^{13}C-NMR data are consistent with the literature.[15]

Methyl 3-bromo-1*H*-pyrrole-2-carboxylate S35

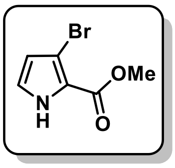

Tetrabutylammonium fluoride solution (1 M, 52.0 mL) was added to a stirred solution of methyl 3-bromo-1-(phenylsulfonyl)-1*H*-pyrrole-2-carboxylate **S34** (9.30 g, 27.02 mmol) in THF (27 mL) and the reaction was heated to 65 ºC for 5 h. The reaction mixture was cooled to RT, concentrated *in vacuo* and loaded directly onto a prepacked SiO_2 column, eluting with 0-100% EtOAc/petrol to afford a pink solid (4.79 g, 87%). ^{1}H-NMR (400 MHz, $CDCl_3$) δ 9.20 (br s, 1H, NH), 6.87 (t, 1H, *J* = 3.1 Hz, NCH), 6.34 (t, 1H, *J* = 3.1 Hz, NCHCH), 3.89 (s, 3H, CH_3); ^{13}C-NMR (100 MHz, $CDCl_3$) δ 160.5 (CO), 122.4 (NCH), 116.9 (C), 114.9 (NCHCH), 103.6 (CBr), 51.7 (CH_3); m.p. 197-198 ˚C (EtOAc/petrol), lit. 202-204 ˚C.[17] ^{1}H-NMR and ^{13}C-NMR data are consistent with the literature.[17]

Methyl 1*H*-pyrrole-2-carboxylate-3-*d* S36

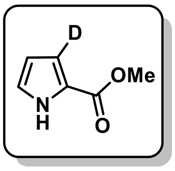

Pd/C (5% wt., 4.71 g) and K_2CO_3 (4.71 g, 34.09 mmol) were added to a stirred solution of methyl 3-bromo-1*H*-pyrrole-2-carboxylate **S35** (5.74 g, 28.41 mmol) in MeOH-d_4 (145 mL) and the reaction mixture was hydrogenated with deuterium for 5.5 h. The reaction mixture was cooled to RT, filtered through celite and concentrated *in vacuo*. The crude material was dissolved in Et_2O (20 mL), washed with H_2O (20 mL) and extracted with Et_2O (2 x 10 mL). The combined organic layers were dried over $MgSO_4$, filtered and concentrated *in vacuo*. The crude product was used directly in the next step without any further purification (37 mg, 59%). ^{1}H-NMR (400 MHz, $CDCl_3$) δ 9.30 (br s, 1H, NH), 6.94 (t, 1H, *J* = 2.7 Hz, ArCH), 6.24 (t, 1H, *J* = 2.7 Hz, ArCH) 3.84 (s, 3H, OCH_3); ^{13}C-NMR (125 MHz, $CDCl_3$) δ 161.7 (CO), 122.9 (ArCH), 122.5 (ArC), 115.0 (t, *J* = 26.7 Hz, ArCD), 110.3 (ArCH), 51.4 (OCH_3).

Methyl 1(but-3-en-1yl)-1*H*-pyrrole-2-carboxylate-3-*d* S37

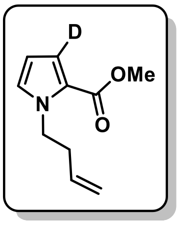

K_2CO_3 (0.15 g, 1.11 mmol), tetrabutylammonium iodide (29 mg, 0.08 mmol) and 4-bromo-1-butene (0.16 mL, 1.58 mmol) was added to a stirred solution of methyl 1*H*-pyrrole-2-carboxylate-3-*d* **S36** (0.1 g, 0.79 mmol) in 2-butanone (1.5 mL) and the reaction mixture was heated under reflux. After 24 h, the reaction mixture was cooled to RT, filtered, concentrated *in vacuo* and purified by SiO_2 flash chromatography, eluting with 0-5% EtOAc/petrol to give the product as a yellow solid (0.14 g, 100%). ^{1}H-NMR (400 MHz, $CDCl_3$) δ 6.83 (d, 1H, *J* = 2.7 Hz, ArCH), 6.11 (d, 1H, *J* = 2.7 Hz, ArCH), 5.81-5.71 (m, 1H, CH), 5.07-5.02 (m, 2H, CHCH), 4.36 (d, 2H, *J* = 7.4 Hz, NCH_2), 3.81 (s, 3H, CH_3), 2.51 (app q, 2H, NCH_2CH_2); ^{13}C-NMR (125 MHz, $CDCl_3$) δ 161.5 (CO), 134.6 ($CHCH_2$), 128.8 (ArCH), 121.3 (ArC), 118.0 (t, *J* = 25.9 Hz, ArCD), 117.21 ($CHCH_2$), 107.7 (ArCH), 51.0 (OCH_3), 48.7 (NCH_2), 35.9 (NCH_2CH_2); υ_{max}/cm^{-1} 1703.44, 1512.87, 1436.63, 1409.55, 1332.22, 1247.27, 1108.12, 1089.80, 786.55, 678.21; *m/z* HRMS ($APCI^+$) found $[M + H]^+$ 181.1078, $[C_{10}H_{13}DNO_2]^+$ requires 181.1087.

1-(But-3-en-1-yl)-1*H*-pyrrole-2-carboxylic-3-*d* acid S38

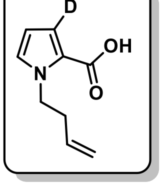

NaOH (2 M aqueous solution, 21 mL) was added to a stirred solution of methyl 1(but-3-en-1yl)-1*H*-pyrrole-2-carboxylate-3-*d* **S37** (2.36 g, 13.09 mmol) in THF (11 mL) and the reaction mixture was heated under reflux for 18

h. The reaction mixture was neutralised with HCl (3 M aqueous solution), extracted with EtOAc (3 x 10 mL) and the combined organic layers were dried over $MgSO_4$, filtered and concentrated *in vacuo*. The crude material was used directly in the next step without further purification (1.72 g, 79%). ^{1}H-NMR (400 MHz, $CDCl_3$) δ 6.88 (d, 1H, *J* = 2.5 Hz, ArCH), 6.15 (d, 1H, *J* = 2.5 Hz, ArCH), 5.81-5.71 (m, 1H, C<u>H</u>CH₂), 5.07-5.02 (m, 2H, CHC<u>H</u>₂), 4.37 (t, 2H, *J* = 7.2 Hz, NCH_2), 2.56-2.51 (m, 2H, NCH₂C<u>H</u>₂); ^{13}C-NMR (125 MHz, $CDCl_3$) δ 165.6 (CO), 134.5 (<u>C</u>HCH₂), 130.0 (ArCH), 120.6 (C), 120.19 (t, *J* = 25.0 Hz, ArCD), 117.4 (CH<u>C</u>H₂), 108.2 (ArCH), 48.9 (NCH_2), 35.9 (NCH₂C<u>H</u>₂); υ_{max}/cm^{-1} 1664.93, 1517.09, 1429.61, 1324.42, 1254.43, 1107.65, 903.98, 727.57, 680.72, 649.94; *m/z* HRMS (ES$^-$) found [M - H]$^-$ 165.0770, $[C_9H_9DNO_2]^+$ requires 165.0774.

tert-Butyl 1-(but-3-en-1-yl)-1*H*-pyrrole-2-carboxylate-3-*d* S39

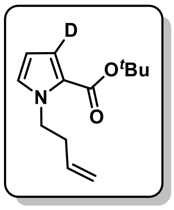

Oxalyl chloride (0.90 mL, 10.66 mmol) was added dropwise over 5 min to a stirred solution of 1-(but-3-en-1-yl)-1*H*-pyrrole-2-carboxylic-3-*d* acid **S38** (1.72 g, 10.35 mmol) and DMF (1 drop) in CH_2Cl_2 (24 mL) at -10 °C. The reaction mixture was warmed to RT for 45 min then cooled to 0 °C and potassium *tert*-butoxide (3.48 g, 31.05 mmol) was added portion wise over 5 min. The reaction was warmed to RT, stirred for 2.5 h and quenched with H_2O (20 mL). The mixture was separated, extracted with CH_2Cl_2 (2 x 30 mL), washed with brine (40 mL), dried over $MgSO_4$, filtered and concentrated *in vacuo*. The crude was purified by SiO_2 flash chromatography, eluting with 0-4% EtOAc/Petrol, to give the product as a yellow oil (1.81 g, 79%). ^{1}H-NMR (400 MHz, $CDCl_3$) δ 6.77 (d, 1H, *J* = 2.6 Hz, ArCH), 6.07 (d, 1H, *J* = 2.5 Hz, ArCH), 5.81-5.71 (m, 1H, C<u>H</u>CH₂), 5.07-5.01 (m, 2H, CHC<u>H</u>₂), 4.34 (t, 2H, *J* = 7.1 Hz, NCH_2), 2.54-2.48 (m, 2H, NCH₂C<u>H</u>₂), 1.55 (s, 9H, CH_3); ^{13}C-NMR (125 MHz, $CDCl_3$) δ 160.6 (CO), 134.7 (<u>C</u>HCH₂), 128.1 (ArCH), 123.1 (ArC), 117.7 (t, *J* = 26.6 Hz, ArCD), 117.1 (CH<u>C</u>H₂), 107.3 (ArCH), 80.2 ($C(CH_3)$), 48.6 (NCH_2), 36.0 (NCH_2CH_2), 28.4 (CH_3); υ_{max}/cm^{-1} 1695.95, 1409.23, 1247.44, 1167.20, 1126.17, 1103.20, 904.14, 726.78, 678.26, 649.63; *m/z* HRMS (ESI$^+$) found [M + Na]$^+$ 245.1369, $[C_{13}H_{18}DNO_2Na]^+$ requires 245.1376.

(±)-*tert*-Butyl (3^1*R*,3a*S*,6a*S*)-1,3a,6,6a-tetrahydroazirino[2,3,1-hi]indole-3^1(2*H*)-carboxylate-5-*d* S40

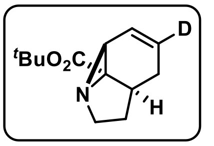

tert-Butyl 1-(but-3-en-1-yl)-1*H*-pyrrole-2-carboxylate-3-*d* **S39** (1.00 g, 4.50 mmol) in degassed cyclohexane:EtOAc (5.7:1, 470 mL) was irradiated using a water cooled 36 W low pressure Hg lamp. After 14 h the irradiation was stopped, and the reaction mixture was concentrated *in vacuo*. The crude material was purified by SiO_2 flash chromatography, eluting with 0-30% EtOAc/petrol, to give a yellow oil (0.40 g, 40%). ^{1}H-NMR (400 MHz, $CDCl_3$) δ 5.76 (br t, 1H, *J* = 3.1 Hz, 6-CH), 3.22-3.14 (m, 2H, 1-CH and 3-CH), 2.81 (d, 1H, *J* = 3.8 Hz, 7-CH), 2.57-2.45 (m, 2H, 1-CH and 2-CH), 2.27 (dt, 1H, *J* = 17.8, 3.6 Hz, 4-CH), 1.89 (d, 1H, *J* = 17.8 Hz, 4-CH), 1.52-1.49 (m, 1H, 2-CH), 1.45 (s, 9H, CH_3); ^{13}C-NMR (125 MHz, $CDCl_3$) δ 171.6 (CO), 134.6 (t, *J* = 24.4 Hz, CD), 120.5 (6-CH), 81.2 ($C(CH_3)$), 52.4 (C), 49.8 (1-CH_2), 43.5 (7-CH), 41.2 (2-CH_2), 33.4 (3-CH), 29.4 (4-CH_2), 28.0 (CH_3); υ_{max}/cm^{-1} 2973.10, 2894.91, 1713.36, 1392.59, 1367.07, 1302.22, 1250.60, 1150.82, 1072.94, 1051.14; *m/z* HRMS (ESI$^+$) found [M + H]$^+$ 223.1553, $[C_{13}H_{19}DNO_2]^+$ requires 223.1557.

(±)-*tert*-Butyl (3a*S*,5*R*,7a*S*)-3,3a,4,5-tetrahydro-7a*H*-indole-7a-carboxylate-5-*d* S41

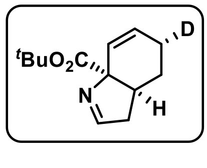

(±)-*tert*-Butyl (3^1*S*,3a*R*,6a*R*)-1,3a,6,6a-tetrahydoazirino[2,3,1-*hi*]indole-3^1(2*H*) carboxylate-5-*d* **S40** (0.38 g, 1.71 mmol) was stirred in PhMe (16 mL) at 100 °C for 16 h. The reaction mixture was concentrated *in vacuo* to give the crude as a brown oil which was used directly in the next step without further purification (0.36 g, 95%). ^{1}H-NMR (400 MHz, $CDCl_3$) δ 7.62 (s, 1H, 1-CH), 6.01 (dd, 1H, *J* = 10.1, 1.5 Hz, 6-CH), 5.94 (dd, 1H, *J* = 10.1, 4.3 Hz, 7-CH), 2.83-2.76 (m, 1H, 2-CH), 2.74-2.67 (m, 1H, 3-CH), 2.40-2.33 (m, 1H, 2-CH), 1.98 (br s, 1H, 5-CH), 1.85-1.78 (m, 1H, 4-CH), 1.44 (s, 9H, CH_3), 1.41-1.34 (m, 1H, 4-CH); ^{13}C-NMR (125 MHz, $CDCl_3$) δ 172.1 (CO), 167.8 (1-CH), 129.9 (6-CH), 126.8 (7-CH), 81.3 (C), 80.9 (C), 42.7 (2-CH_2), 37.2 (3-CH), 27.9 (CH_3), 24.6 (4-CH_2), 21.1 (t, *J* = 19.6 Hz, 5-CDH); υ_{max}/cm^{-1} 2975.43, 2927.64, 1722.01, 1367.41, 1248.61, 1159.49, 1126.90, 1079.18, 1055.61, 846.64; *m/z* HRMS (ESI$^+$) found [M + H]$^+$ 223.1554, $[C_{13}H_{19}DNO_2]^+$ requires 223.1557.

(±)-*tert*-Butyl (3a*S*,5*R*,7a*R*)-1-(4-methoxybenzyl)-1,2,3,3a,4,5-hexahydro-7a*H*-indole-7a-carboxylate-5-*d* 22

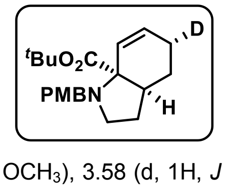

According to general procedure A, crude (±)-*tert*-butyl (3a*S*,7a*R*)-3-3a,4,5-tetrahydro-7a*H*-indole-7a-carboxylate-5-*d* **S40** (0.34 g, 1.53 mmol) and *p*-anisaldehyde (0.19 mL, 1.53 mmol) were stirred with sodium triacetoxyborohydride (0.97 g, 4.86 mmol) in CH_2Cl_2 (7 mL). The crude was purified by SiO_2 flash chromatography, 0-5% EtOAc/petrol, to give the product as a yellow oil (0.39 g, 74%). ^{1}H-NMR (400 MHz, $CDCl_3$) δ 7.26 (d, 2H, *J* = 8.7 Hz, ArCH), 6.83 (d, 2H, *J* = 8.7 Hz, ArCH), 6.01 (dd, 1H, *J* = 10.5, 3.4 Hz, 6-CH), 5.84 (dd, 1H, *J* = 10.5, 2.04 Hz, 7-CH), 3.81 (d, 1H, *J* = 13.6 Hz, ArC<u>H</u>H), superimposed on 3.79 (s, 3H, OCH_3), 3.58 (d, 1H, *J* = 13.6 Hz, ArCH<u>H</u>), 2.69 (td, 1H, *J* = 9.2, 4.8 Hz, 1-CH), 2.63-2.51 (m, 2H, 1-CH and 3-CH), 2.07 (br s, 1H, 5-CH), 2.03-1.93 (m, 1H, 2-CH), 1.76-1.69 (m, 1H, 4-CH), 1.65-1.59 (m, 1H, 4-CH), 1.54-1.50 (m, 1H, 2-CH) superimposed on 1.50 (s, 9H, CH_3); ^{13}C-NMR (100 MHz, $CDCl_3$) δ 173.8 (CO), 158.4 (ArC), 132.8 (ArC), 130.6 (6-CH), 129.4 (ArCH), 124.7 (7-CH), 113.5 (ArCH), 80.7 ($C(CH_3)$), 69.0 (C), 55.2 (OCH_3), 53.4 ($ArCH_2$), 49.8 (1-CH_2), 40.4 (3-CH), 28.2 (CH_3), 27.3 (2-CH_2), 25.3 (4-CH_2), 22.0 (t, *J* = 17.9 Hz, CDH); υ_{max}/cm^{-1} 2971.95, 2929.50, 1716.42, 1510.82, 1366.56, 1243.43, 1161.35, 1074.76, 1065.62, 1042.43; *m/z* HRMS (ESI$^+$) found [M + H]$^+$ 345.2278, $[C_{21}H_{29}DNO_3]^+$ requires 345.2288.

(±)-*tert*-Butyl 2-(4-methoxybenzyl)-2-azabicyclo[3.3.1]non-6-ene-6-carboxylate-1-*d* 24

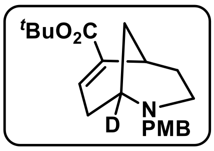

According to general procedure C, (±)-*tert*-butyl (3a*S*,7a*R*)-1-(4-methoxybenzyl)-1,2,3,3a,4,5-hexahydro-7a*H*-indole-7a-carboxylate-5-*d* **22** (52 mg, 0.15 mmol) in dioxane (0.6 mL) was added to a stirred solution of $Pd(OAc)_2$ (3 mg, 0.02 mmol), DPEPhos (12 mg, 0.02 mmol) and *N*,*N*-diisopropylethylamine (0.03 mL, 0.15 mmol) in dioxane (0.6 mL). After 10 min methanesulfonic acid (0.01 mL, 0.15 mmol) was added and the reaction was heated under reflux. The crude was purified by SiO_2 flash chromatography, eluting with 5-50% EtOAc/petrol to give a yellow oil (25 mg, 48%). ^{1}H-NMR (400 MHz, $CDCl_3$) δ 7.25 (d, 2H, *J* = 8.7 Hz, ArCH), 7.07 (t, 1H, *J* = 3.6 Hz, 7-CH), 6.85 (d, 2H, *J* = 8.7 Hz, ArCH), 3.80 (s, 3H, OCH_3), 3.50 (d, 2H, *J* = 5.1 Hz, $ArCH_2$), 2.98 (br s, 0.4H, 1-CH), 2.87 (br s, 1H, 5-CH), 2.54-2.43 (m, 2H, 3-CH and 8-CH), 2.24 (td, 1H, *J* = 12.2, 3.1 Hz, 3-CH), 2.05-1.90 (m, 2H, 8-CH and 9-CH), 1.87-1.79 (m, 1H, 4-CH), 1.58-1.53 (m, 1H, 9-CH), 1.48 (s, 9H, CH_3) superimposed on 1.48-1.46 (m, 1H, 4-CH); ^{13}C-NMR (125 MHz, $CDCl_3$) δ 165.9 (CO), 158.6 (ArC), 140.2 (7-CH), 134.4 (6-C), 131.3 (ArC), 129.9 (ArCH), 113.6 (ArCH), 79.9 (C), 58.8 ($ArCH_2$), 55.2 (OCH_3), 49.5 (1-CH), 49.0 (t, *J* = 20.8 Hz, 1-CD), 44.2 (3-CH_2), 31.7 (9-CH_2), 28.9 (4-CH_2), 28.2 (CH_3), 26.9 (5-CH), 24.7 (8-CH_2); υ_{max}/cm^{-1} 2931.58, 1702.20, 1512.10, 1366.97, 1247.19, 1168.49, 1072.35, 903.22, 724.29, 649.94; *m/z* HRMS (ESI$^+$) found [M + H]$^+$ 345.2280, $[C_{21}H_{29}DNO_3]^+$ requires 345.2288.

But-3-en-1,1-*d*$_2$-1-ol S42

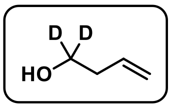

3-Butenoic acid (3.39 mL, 39.9 mmol) was added dropwise over 10 min to a stirred solution of lithium aluminium deuteride (1.91 g, 45.50 mmol) in Et_2O (68 mL) at 0 °C. Significant effervescence was observed during this addition. The reaction mixture was stirred at 0 °C for 20 min, heated to reflux for 2 h then cooled to RT and stirred for a further 4 h. The reaction mixture was quenched with H_2O (1 mL), NaOH (15% aqueous solution, 3.3 mL) and H_2O (1 mL). After 15 min the solution was filtered through celite, the filtrate dried over $MgSO_4$, filtered and concentrated *in vacuo* to give the crude material as a yellow oil which was used directly in the next step without purification (2.61 g, 88% accounting for 0.22 equiv. Et_2O). ^{1}H-NMR (400 MHz, $CDCl_3$) δ 5.86-5.75 (m, 1H, CH), 5.18-5.11 (m, 2H, CH$\underline{H_2}$), 2.32 (br d, 2H, CH_2); ^{13}C-NMR (125 MHz, $CDCl_3$) δ 134.8 (CH), 117.7 (CH$\underline{C}H_2$), 60.9 (qn, *J* = 21.9 Hz, CD_2), 36.9 (CH_2).

1-(1-(But-3-en-1-yl-1,1-*d*$_2$)-1*H*-pyrrol-2-yl)-2,2,2,-trichloroethan-1-one S43

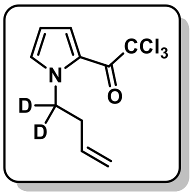

Diisopropyl azodicarboxylate (0.12 mL, 0.61 mmol) was added dropwise to a stirred solution of triphenylphosphine (0.16 g, 0.60 mmol) in THF (1.5 mL) at -78 °C. The mixture was stirred for 50 min and but-3-en-1,1-*d*$_2$-1-ol **S40** (81% solution in Et_2O, 61 mg, 0.67 mmol) was added dropwise. After a further 1 h, 2-(trichloroacetyl)pyrrole (0.13 g, 0.59 mmol) was added in one portion and the reaction mixture was warmed to RT. After 21 h, the reaction mixture was concentrated *in vacuo* and the residue triturated in Et_2O/Petrol (2:3, 5 mL) at -10 °C and filtered. The residue was concentrated *in vacuo* and purified by SiO_2 flash chromatography, eluting with 0-2% Et_2O/petrol, to give the product as a light yellow oil (94 mg, 59%). ^{1}H-NMR (400 MHz, $CDCl_3$) δ 7.54 (dd, 1H, *J* = 4.4, 1.6 Hz, ArCH), 7.00-6.99 (m, 1H, ArCH), 6.23-6.21 (m, 1H, ArCH), 5.82-5.71 (m, 1H, CH), 5.08-5.02 (m, 2H, CH$\underline{H_2}$), 2.50 (d, 1H, *J* = 6.9 Hz, NCD_2CH_2); ^{13}C-NMR (125 MHz, $CDCl_3$) δ 172.6 (CO), 134.0 (CH), 133.1 (ArCH), 124.7 (ArCH), 121.0 (ArC), 117.8 (CH$\underline{C}H_2$), 108.9 (ArCH), 96.4 (C), 49.5 (qn, *J* = 21.8 Hz, CD_2), 35.2 ($NCD_2\underline{C}H_2$); υ_{max}/cm^{-1} 1666.74, 1458.75, 1408.55, 1354.48, 1319.26, 1057.48, 842.73, 802.15, 741.25, 686.08; *m/z* HRMS (ESI$^+$) found [M + H]$^+$ 267.9948, $[C_{10}H_9D_2ClNO]^+$ requires 268.0032.

1-(But-3-en-1-yl-1,1-*d*$_2$)-1*H*-pyrrole-2-carboxylic acid S44

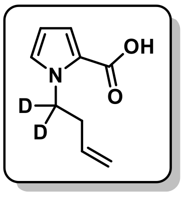

NaOH (2 M aqueous solution, 26 mL) was added to a stirred solution of 1-(1-(but-3-en-1-yl-1,1-*d*$_2$)-1*H*-pyrrol-2-yl)-2,2,2,-trichloroethan-1-one **S43** (3.93 g, mmol) in THF (14 mL) and the reaction mixture was heated to 60 °C for 16 h. The reaction was neutralised with HCl (3 M aqueous solution), extracted with EtOAc (3 x 30 mL) and the combined organic layers were dried over $MgSO_4$, filtered and concentrated *in vacuo*. The crude material was used directly in the next step without further purification (2.29 g, 93 %). ^{1}H-NMR (400 MHz, $CDCl_3$) δ 7.12 (dd, 1H, *J* = 4.2, 1.8 Hz, ArCH), 6.88 (app t, 1H, ArCH), 6.16-6.14 (m, 1H, ArCH), 5.81-5.71 (m, 1H, CH), 5.07-5.02 (m 2H, CH$\underline{H_2}$), 2.52 (d, 2H, *J* = 7.1 Hz, NCD_2CH_2); ^{13}C-NMR (125 MHz, $CDCl_3$) δ 165.9 (CO), 134.4 (CH), 130.0 (ArCH), 122.2 (ArC), 120.4 (ArCH), 117.4 (CH$\underline{C}H_2$), 108.3 (ArCH), 48.3 (qn, *J* = 21.2 Hz, CD_2), 35.7 ($NCD_2\underline{C}H_2$); υ_{max}/cm^{-1} 2973.06, 1664.21, 1462.90, 1428.70, 1312.93, 1256.18, 1098.46, 1079.62, 1054.71, 740.53; *m/z* HRMS (ES$^-$) found [M - H]$^-$ 166.0837, $[C_9H_9DNO_2]^+$ requires 166.0843.

***tert*-Butyl 1-(but-3-en-1-yl-1,1-*d*$_2$)-1*H*-pyrrole-2-carboxylate S45**

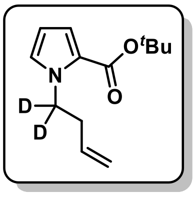

Oxalyl chloride (0.90 mL, 10.66 mmol) was added dropwise over 5 min to a stirred solution of 1-(but-3-en-1-yl)-1*H*-pyrrole-2-carboxylic-3-*d* acid **S44** (1.72 g, 10.35 mmol) and DMF (1 drop) in CH_2Cl_2 (24 mL) at -10 °C. The reaction mixture was warmed to RT for 45 min then cooled to 0 °C and potassium *tert*-butoxide (3.48 g, 31.05

mmol) was added portion wise over 5 min. The reaction was warmed to RT, stirred for 2.5 h and quenched with H_2O (20 mL). The mixture was separated, extracted with CH_2Cl_2 (2 x 30 mL), washed with brine (40 mL), dried over $MgSO_4$, filtered and concentrated *in vacuo*. The crude was purified by SiO_2 flash chromatography, eluting with 0-4% EtOAc/petrol, to give the product as a yellow oil (1.81 g, 79%). ^{1}H-NMR (400 MHz, $CDCl_3$) δ 6.89-6.87 (m, 1H, ArCH), 6.78-6.76 (m, 1H, CH), 6.07 (dd, 1H, *J* = 3.9, 2.6 Hz, ArCH), 5.81-5.71 (m, 1H, C<u>H</u>CH_2), 5.07-5.01 (m, 2H, CHC<u>H$_2$</u>), 2.49 (d, 2H, *J* = 6.8 Hz, NCD_2CH_2), 1.55 (s, 9H, CH_3); ^{13}C-NMR (125 MHz, $CDCl_3$) δ 160.6 (CO), 134.7 (<u>C</u>HCH_2), 128.1 (ArCH), 123.2 (ArC), 117.9 (ArCH), 117.1 (CH<u>C</u>H_2), 107.4 (ArCH), 80.2 (C), 48.1 (qn, *J* = 21.7 Hz, CD_2), 35.8 (NCD_2<u>C</u>H_2), 28.4 (CH_3); υ_{max}/cm^{-1} 1696.93, 1413.32, 1367.66, 1315.89, 1250.76, 1178.09, 1144.62, 1121.78, 1097.41, 735.62; *m/z* HRMS (ESI$^+$) found [M + Na]$^+$ 246.1444, $[C_{13}H_{17}D_2NNaO_2]^+$ requires 246.1439.

(±)-tert-*Butyl (3^{1}R,3aS,6aS)-1,3a,6,6a-tetrahydroazirino[2,3,1-*hi*]indole-3^1(2H)-carboxylate-2,2-*d$_2$ S46

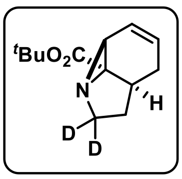

tert-Butyl 1-(but-3-en-1-yl-1,1-*d$_2$*)-1*H*-pyrrole-2-carboxylate **S45** (1.00 g, 4.48 mmol) in degassed cyclohexane:EtOAc (5.7:1, 470 mL) was irradiated using a water cooled 36 W low pressure Hg lamp. After 14 h the irradiation was stopped, and the reaction mixture was concentrated *in vacuo*. The crude material was purified by SiO_2 flash chromatography, eluting with 5-40% EtOAc/petrol, to give a yellow oil (0.27 g, 27%). ^{1}H-NMR (400 MHz, $CDCl_3$) δ 6.24-6.19 (m, 1H, 5-CH), 5.79 (dt, 1H, *J* = 10.1, 3.4 Hz, 6-CH), 3.22-3.19 (m, 1H, 3-CH), 2.85 (d, 1H, *J* = 3.8 Hz, 7-CH), 2.53 (t, 1H, *J* = 11.2 Hz, 2-CH), 2.30 (br d, *J* = 17.6 Hz, 4-CH), 1.92 (dd, 1H, *J* = 17.6, 6.3 Hz, 4-CH), 1.51 (br s, 1H, 2-CH) superimposed on 1.48 (s, 9H, CH_3); ^{13}C-NMR (125 MHz, $CDCl_3$) δ 171.6 (CO), 135.0 (5-CH), 120.7 (6-CH), 81.2 (<u>C</u>$(CH_3)_3$), 52.4 (C), 49.2 (qn, *J* = 21.2 Hz, CD_2), 43.4 (7-CH), 41.0 (2-CH_2), 33.5 (3-CH), 29.5 (4-CH_2), 28.0 (CH_3); υ_{max}/cm^{-1} 2975.86, 2932.89, 1714.23, 1367.68, 1324.01, 1303.69, 1276.19, 1252.87, 1149.56, 1057.61; *m/z* HRMS (ESI$^+$) found [M + H]$^+$ 224.1614, $[C_{13}H_{18}D_2NO_2]^+$ requires 224.1620.

(±)-*tert*-Butyl (3a*S*,5*S*,7a*S*)-3,3a,4,5-tetrahydro-7a*H*-indole-7a-carboxylate-2,5-*d$_2$* 23

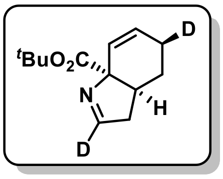

(±)-*tert*-Butyl (3^1*R*,3a*S*,6a*S*)-1,3a,6,6a-tetrahydroazirino[2,3,1-*hi*]indole-3^1(2*H*)-carboxylate-2,2-*d$_2$* **S46** (0.25 g, 1.12 mmol) was stirred in PhMe (10 mL) at 100 °C for 16 h. The reaction mixture was concentrated *in vacuo* to give the crude as a brown oil which was used directly in the next step without further purification (0.25 g, 100%). ^{1}H-NMR (400 MHz, $CDCl_3$) δ 6.04 (dd, 1H, *J* = 10.2, 2.0 Hz, 7-CH), 5.97 (dd, 1H, *J* = 10.2, 3.3 Hz, 6-CH), 2.86-2.79 (m, 1H, 2-CH), 2.76-2.70 (m, 1H, 3-CH), 2.39 (dd, 1H, *J* = 17.1, 5.2 Hz, 2-CH), 1.95 (br s, 1H, 5-CH), 1.84 (dt, 1H, *J* = 13.2, 4.9 Hz, 4-CH), 1.44 (s, 9H, CH_3), 1.41-1.34 (m, 1H, 4-CH), 1.46 (s, 9H, CH_3), 1.39-1.34 (m, 1H, 4-CH); ^{13}C-NMR (125 MHz, $CDCl_3$) δ 172.0 (CO), 167.4 (t, *J* = 28.1 Hz, NCD), 130.0 (6-CH), 126.8 (7-CH), 81.4 (<u>C</u>$(CH_3)_3$), 80.9 (C), 42.7 (2-CH_2), 37.2 (3-CH), 27.9 (CH_3), 24.8 (4-CH_2), 21.2 (t, *J* = 19.3 Hz, CD); υ_{max}/cm^{-1} 2976.74, 2930.18, 1722.76, 1392.52, 1367.77, 1254.57, 1162.82, 1092.51, 1064.74, 847.78; *m/z* HRMS (ESI$^+$) found [M + H]$^+$ 224.1615, $[C_{13}H_{18}D_2NO_2]^+$ requires 224.1620.

(±)-*tert*-Butyl (2*R*,3a*S*,5*S*,7a*R*)-1-(4-methoxybenzyl)-1,2,3,3a,4,5-hexahydro-7a*H*-indole-7a-carboxylate-2,5-*d$_2$* 23

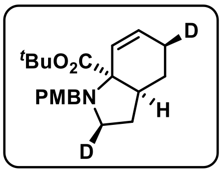

According to general procedure A, crude (±)-*tert*-butyl (3a*S*,5*S*,7a*S*)-3,3a,4,5-tetrahydro-7a*H*-indole-7a-carboxylate-2,5-*d$_2$* **23** (0.24 g, 1.07 mmol) and *p*-anisaldehyde (0.13 mL, 1.07 mmol) were stirred with sodium triacetoxyborohydride (0.68 g, 3.21 mmol) in CH_2Cl_2 (5 mL). The crude was purified by SiO_2 flash chromatography, 0-10% EtOAc/petrol, to give the product as a yellow oil (0.15 g, 41%). ^{1}H-NMR (400 MHz, $CDCl_3$) δ 7.26 (d, 2H, *J* = 8.8 Hz, ArCH), 6.83 (d, 2H, *J* = 8.8 Hz, ArCH), 6.01 (dd, 1H, *J* = 10.4, 4.0 Hz, 7-CH), 5.85 (dd, 1H, *J* = 10.4, 1.9 Hz, 6-CH), 3.79 (dd, 1H, *J* = 13.0 Hz, ArCH_2), superimposed on 3.79 (s, 3H, OCH_3), 3.60 (dd, 1H, *J* = 13.0 Hz, ArCH_2), 2.67 (dd, 1H, *J* = 9.1, 4.8 Hz, 1-CHD), 2.61-2.55 (m, 1H, 3-CH), 2.02-1.94 (m, 2H, 2-CH and 5-CHD), 1.72 (dt, 1H, *J* = 13.2, 4.7 Hz, 4-CH), 1.62 (app qn, 1H, 4-CH), 1.50 (s, 9H, CH_3), 1.47-1.46 (m, 1H, 2-CH);^{13}C-NMR (125 MHz, $CDCl_3$) δ 173.8 (CO), 158.4 (ArC), 132.8 (ArC), 130.6 (7-CH), 129.4 (ArCH), 124.8 (6-CH), 113.5 (ArCH), 80.8 (<u>C</u>$(CH_3)_3$), 69.0 (C), 55.2 (OCH_3), 53.4 (ArCH_2), 49.4 (t, *J* = 20.6 Hz, 2-CDH), 40.4 (3-CH), 28.2 (CH_3), 27.3 (2-CH_2), 25.4 (4-CH_2), 22.1 (t, *J* = 19.2 Hz, 5-CDH); υ_{max}/cm^{-1} 2932.60, 1716.79, 1612.26, 1511.21, 1366.91, 1244.67, 1163.85, 1038.84, 845.88, 821.14; *m/z* HRMS (ESI$^+$) found [M + H]$^+$ 346.2352, $[C_{21}H_{28}D_2NO_3]^+$ requires 346.2351.

(±)-*tert*-Butyl 2-(4-methoxybenzyl)-2-azabicyclo[3.3.1]non-6-ene-6-carboxylate-1,3-*d$_2$* 24

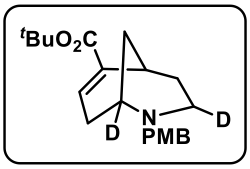

According to general procedure C, (±)-*tert*-butyl (2*R*,3a*S*,5*S*,7a*R*)-1-(4-methoxybenzyl)-1,2,3,3a,4,5-hexahydro-7a*H*-indole-7a-carboxylate-2,5-*d$_2$* **22** (52 mg, 0.15 mmol) in dioxane (0.6 mL) was added to a stirred solution of $Pd(OAc)_2$ (3 mg, 0.02 mmol), DPEPhos (12 mg, 0.02 mmol) and *N*,*N*-diisopropylethylamine (0.03 mL, 0.15 mmol) in dioxane (0.6 mL). After 10 min methanesulfonic acid (0.01 mL, 0.15 mmol) was added and the reaction was heated under reflux. The crude was purified by SiO_2 flash chromatography, eluting with 10-25% EtOAc/petrol to give a yellow oil (20 mg, 38%). ^{1}H-NMR (400 MHz, $CDCl_3$) δ 7.25 (d, 2H, *J* = 9.0 Hz, ArCH), 7.07 (t, 1H, *J* = 3.8 Hz, 7-CH), 6.85 (d, 2H, *J* = 9.0 Hz, ArCH), 3.79 (s, 3H, OCH_3), 3.50 (d, 2H, *J* = 3.4 Hz, ArCH_2), 3.00 (br s, 0.7H, 1-CH), 2.88 (t, 1H, *J* = 3.2 Hz, 5-CH), 2.48 (br dd, 1H, *J* = 20.0, 4.5 Hz, 8-CH), 2.23 (dd, 1H, *J* = 12.8, 3.0 Hz, 3-CHD), 2.06-1.99 (m, 1H, 8-CH), 1.95-1.92 (m, 1H, 9-CH), 1.83 (td, 1H, *J* = 12.9, 4.4 Hz, 4-CH) 1.59-1.53 (m, 1H, 9-CH), 1.48 (s, 9H, CH_3), 1.43-1.42 (m, 1H, 4-CH); ^{13}C-NMR (125 MHz, $CDCl_3$) δ 165.9 (CO), 158.6 (ArC), 140.1 (7-CH), 134.4 (6-C), 131.0 (ArC), 129.9 (ArCH), 113.6 (ArCH), 79.9 (C), 58.8 (ArCH_2), 55.2 (OCH_3), 49.5 (1-CH), 49.0 (t,

J = 21.1 Hz, 1-CD), 43.8 (t, *J* = 20.5 Hz, 3-CDH), 31.8 (9-CH_2), 28.7 (4-CH_2), 28.2 (CH_3), 26.9 (5-CH), 24.8 (8-CH_2); υ_{max}/cm^{-1} 2929.43, 1724.69, 1512.21, 1367.28, 1301.73, 1244.31, 11551.36, 1105.27, 1037.31, 835.80; *m/z* HRMS (ESI^+) found $[M + H]^+$ 346.2330, $[C_{21}H_{28}D_2NO_3]^+$ requires 345.2288.

Derivatization of Morphan

(±)-2-Benzyl 6-(*tert*-butyl) (1*R*)-2-azabicyclo [3.3.1]non-6-ene-2,6-dicarboxylate 18

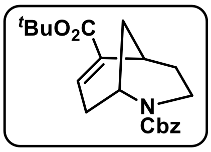

Benzyl chloroformate (0.03 mL, 0.23 mmol) was added to a stirred solution of (±)-*tert*-butyl (3a*S*,7a*R*)-1-(4-methoxybenzyl)-1,2,3,3a,4,5-hexhydro-7a*H*-indole-7a-carboxylate **17e** (50 mg, 0.15 mmol) in $CHCl_3$ (1 mL). The reaction mixture was stirred for 17.5 h, concentrated *in vacuo* and loaded directly onto a prepacked SiO_2 column, eluting with 0-20% EtOAc/petrol to afford the product as a yellow oil (43 mg, 80%). ^{1}H-NMR (400 MHz, $CDCl_3$) δ 7.35 (br s, 5H, ArCH), 7.06-7.04 (m, 1H, 7-CH), 5.14-5.12 (m, 2H, ArCH_2), 4.50 (d, 1H, *J* = 29.6 Hz, 1-CH), 3.97 (dd, 1H, *J* = 31.2, 4.0 Hz, 3-CH), 2.96-2.85 (m, 2H, 3-CH and 5-CH), 2.60-2.49 (m, 1H, 8-CH), 2.24-2.14 (m, 1H, 8-CH), 1.85-1.70 (m, 2H, 4-CH and 9-CH), 1.65-1.58 (m, 2H, 4-CH and 9-CH), 1.48 (s, 9H, CH_3); ^{13}C-NMR (100 MHz, $CDCl_3$) δ 165.5 (CO), 139.0 (7-CH), 136.9 (6-C), 128.5 (ArCH), 127.9 (ArCH) 127.8 (ArCH), 113.9 (ArC), 80.3 (C), 66.9 (ArCH_2), 44.3 (1-CH), 37.2 (3-CH_2), 32.6 (8-CH_2), 30.5 (9-CH_2), 28.1 (CH_3), 27.9 (4-CH_2), 26.5 (5-CH); υ_{max}/cm^{-1} 1694.38, 1366.42, 1410.74, 1275.42, 1250.92, 1215.15, 1163.60, 1088.17, 1067.31, 697.44; *m/z* HRMS (ESI^+) found $[M + H]^+$ 358.2001 and $[M = Na]^+$ 380.1808, $[C_{21}H_{28}NO_4]^+$ requires 358.2018 and $[C_{21}H_{27}NNaO_4]^+$ requires 380.1838.

(±)-*tert*-Butyl (1*R*)-2-azabicyclo [3.3.1]non-6-ene-6-carboxylate 19

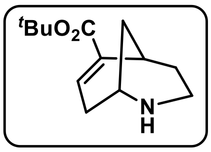

1-Chloroethyl chloroformate (0.02 mL, 0.23 mmol) was added to a stirred solution of (±)-*tert*-butyl (3a*S*,7a*R*)-1-(4-methoxybenzyl)-1,2,3,3a,4,5-hexhydro-7a*H*-indole-7a-carboxylate **17e** (50 mg, 0.15 mmol) in $CHCl_3$ (1 mL). After 5 h, the reaction mixture was concentrated *in vacuo*, dissolved in MeOH (1.5 mL) and stirred for 16 h. The reaction mixture was concentrated *in vacuo* and loaded directly onto a prepacked SiO_2 column, eluting with 10-20% EtOAc/petrol then 0-10% MeOH/CH_2Cl_2 to give the product as a brown solid (23 mg, 70%). ^{1}H-NMR (400 MHz, $CDCl_3$) δ 9.56 (br s, 1H, NH), 7.02 (t, 1H, *J* = 3.6 Hz, 7-CH), 3.88 (br s, 1H, 1-CH), 3.22 (dd, 1H, *J* = 13.2, 4.2 Hz, 3-CH), 3.05 (br s, 1H, 5-CH), 2.97 (td, 1H, *J* = 13.2, 3.4 Hz, 3-CH), 2.83 (dd, 1H, *J* = 21.4, 3.6 Hz, 8-CH), 2.69-2.61 (m, 1H, 8-CH), 2.35 (br d, 1H, *J* = 13.3 Hz, 9-CH), 2.16 (tt, 1H, *J* = 13.7, 4.2 Hz, 4-CH), 1.75-1.66 (m, 2H, 4-CH and 9-CH), 1.48 (s, 9H, CH_3); ^{13}C-NMR (100 MHz, $CDCl_3$) δ 164.6 (CO), 137.2 (7-CH), 133.7 (6-C), 81.0 (C), 45.8 (1-CH), 36.3 (3-CH_2), 28.9 (8-CH_2), 28.1 (CH_3), 27.9 (9-CH_2), 26.0 (4-CH_2), 25.0 (5-CH); υ_{max}/cm^{-1} 2933.50, 2791.76, 2706.77, 2758.29, 1699.83, 1369.75, 1288.95, 1253.13, 1165.84, 1081.04; *m/z* HRMS (ESI^+) found $[M + H]^+$ 224.1648, $[C_{13}H_{22}NO_2]^+$ requires 224.1651; m.p. 216-218 ˚C (MeOH/CH_2Cl_2).

Crystal Structure Data

(±)-*tert*-Butyl (1*R*)-2-(3,4-dimethoxyphenethyl)-2-azabicyclo[3.3.1]non-6-ene-6-carboxylate 11

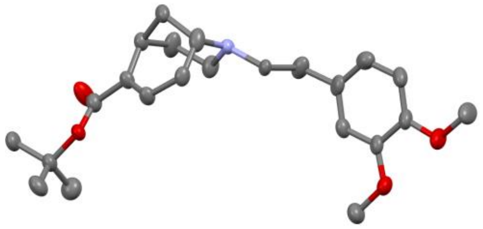

Table S4. Crystal data and structure refinement.

Identification code	WY09_083A
Empirical formula	$C_{23}H_{34}INO_4$
Formula weight	515.41
Temperature/K	100(2)
Crystal system	monoclinic
Space group	$P2_1/c$
a/Å	20.0118(3)

b/Å	8.18260(10)
c/Å	29.6117(5)
α/°	90
β/°	91.7360(10)
γ/°	90
Volume/Å^3	4846.65(12)
Z	8
ρ_{calc}g/cm^3	1.413
μ/mm^{-1}	1.348
F(000)	2112.0
Crystal size/mm^3	0.268 × 0.209 × 0.168
Radiation	MoKα (λ = 0.71073)
2Θ range for data collection/°	2.036 to 54.342
Index ranges	-25 ≤ h ≤ 22, -10 ≤ k ≤ 10, -37 ≤ l ≤ 35
Reflections collected	39473
Independent reflections	10726 [R_{int} = 0.0403, R_{sigma} = 0.0398]
Data/restraints/parameters	10726/601/695
Goodness-of-fit on F^2	1.235
Final R indexes [I>=2σ (I)]	R_1 = 0.0581, wR_2 = 0.1147
Final R indexes [all data]	R_1 = 0.0693, wR_2 = 0.1183
Largest diff. peak/hole / e Å^{-3}	1.32/-1.31

References

(1) Keinan, E.; Kumar, S.; Dangu, V.; Vaya, J. *J. Am. Chem. Soc.* **1994**, *116*, 11151–11152.

(2) Simmons, E. M.; Hartwig, J. F. *Angew. Chem. Int. Ed.* **2012**, *51*, 3066–3072.

(3) Cristau, H.-J.; Mouchet, P.; Cristau, H. *Phosphorus. Sulfur. Silicon Relat. Elem.* **1995**, *107*, 135–144.

(4) Blackham, E. E.; Booker-Milburn, K. I. *Angew. Chem. Int. Ed.* **2017**, *56*, 6613–6616.

(5) Yu, W. L.; Nunns, T.; Richardson, J.; Booker-Milburn, K. I. *Org. Lett.* **2018**, *20*, 1272–1274.

(6) Huang, Q.; Larock, R. C. *Org. Lett.* **2002**, *4*, 2505–2508.

(7) Hashmi, A. S. K.; Lothschütz, C.; Döpp, R.; Ackermann, M.; De Buck Becker, J.; Rudolph, M.; Scholz, C.; Rominger, F. *Adv. Synth. Catal.* **2012**, *354*, 133–147.

(8) Coya, E.; Sotomayor, N.; Lete, E. *Adv. Synth. Catal.* **2015**, *357*, 3206–3214.

(9) Gupton, J. T.; Giglio, B. C.; Eaton, J. E.; Rieck, E. A.; Smith, K. L.; Keough, M. J.; Barelli, P. J.; Firich, L. T.; Hempel, J. E.; Smith, T. M.; et al. *Tetrahedron* **2009**, *65*, 4283–4292.

(10) Jenkins, E. F.; Costello, E. J.; Kaufmann, S.; Rosenkranz1, G.; López, J. *J . Am. Chem. Soc.* **1946**, *68*, 2733–2734.

(11) Kapadia, N.; Harding, W. *Tetrahedron* **2013**, *69*, 8914–8920.

(12) Song, S.; Sun, X.; Li, X.; Yuan, Y.; Jiao, N. *Org. Lett.* **2015**, *17*, 2886–2889.

(13) Rodríguez, F.; Burton, K. I.; Franzoni, I.; Petrone, D. A.; Scheipers, I.; Lautens, M. *Org. Lett.* **2019**, *13*, 59.

(14) *J . Am. Chem. Soc.* **2014**, *136*, 7205–7208.

(15) Moir, M.; Boyd, R.; Gunosewoyo, H.; Montgomery, A. P.; Connor, M.; Kassiou, M. *Tetrahedron Lett.* **2019**, *60*, 151019.

(16) Ohta, T.; Fukuda, T.; Ishibashi, F.; Iwao, M. *J. Org. Chem* **2009**, *74*, 8143.

(17) Axford, L. C.; Holden, K. E.; Hasse, K.; Banwell, M. G.; Steglich, W.; Wagler, J.; Willis, A. C. *Aust. J. Chem* **2008**, *61*, 80–93.

Author Contributions

CDCl$_3$

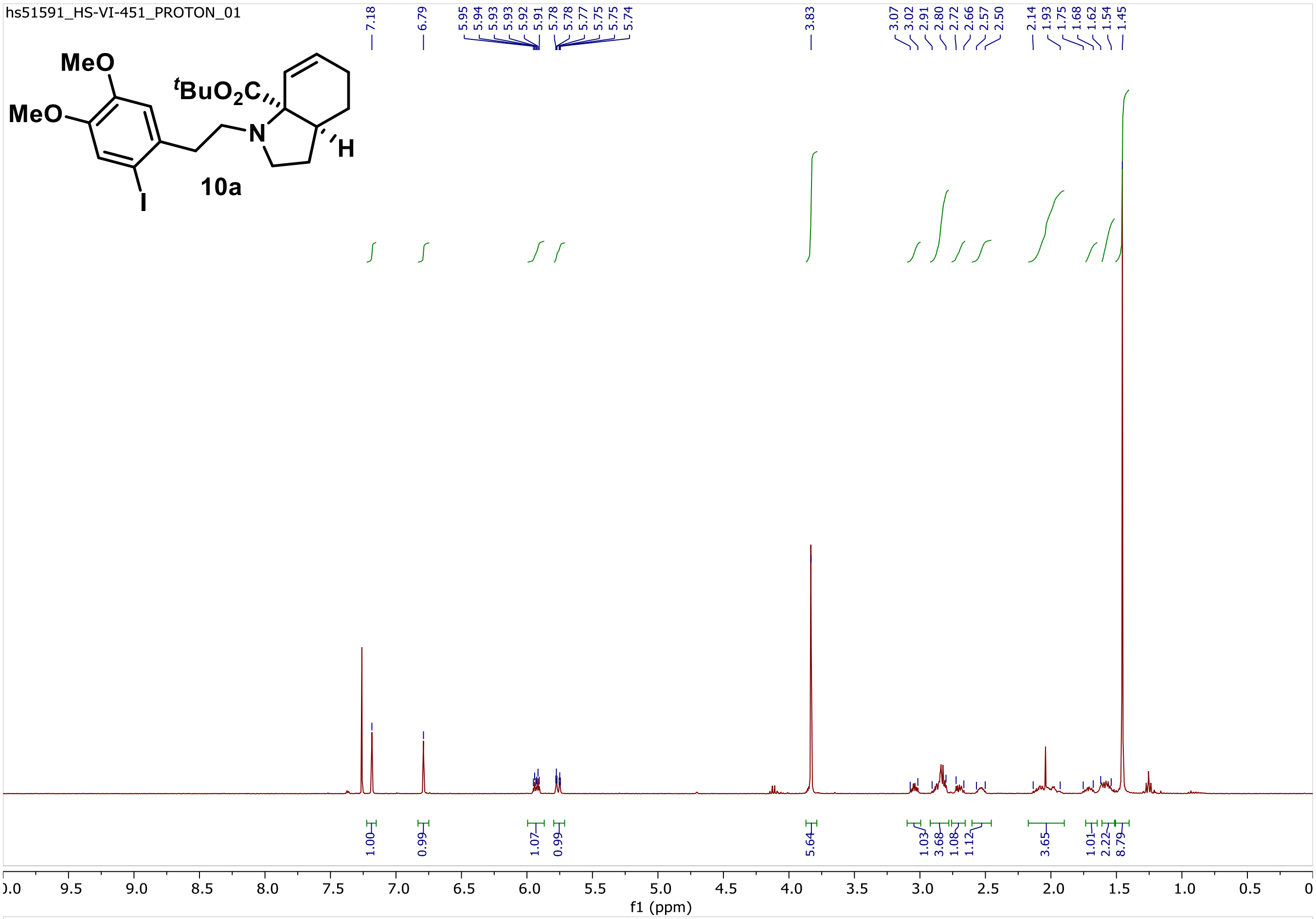

hs51591_HS-VI-451_PROTON_01
MeO
MeO
^{t}BuO$_2$C
N
H
I
10a
7.18
6.79
5.95 5.94 5.93 5.93 5.92 5.91 5.78 5.78 5.77 5.75 5.75 5.74
3.83
3.07 3.02 2.91 2.80 2.72 2.66 2.57 2.50
2.14 1.93 1.75 1.68 1.62 1.54 1.45
1.00 0.99 1.07 0.99 5.64 1.03 3.68 1.08 1.12 3.65 1.01 2.22 8.79
f1 (ppm)

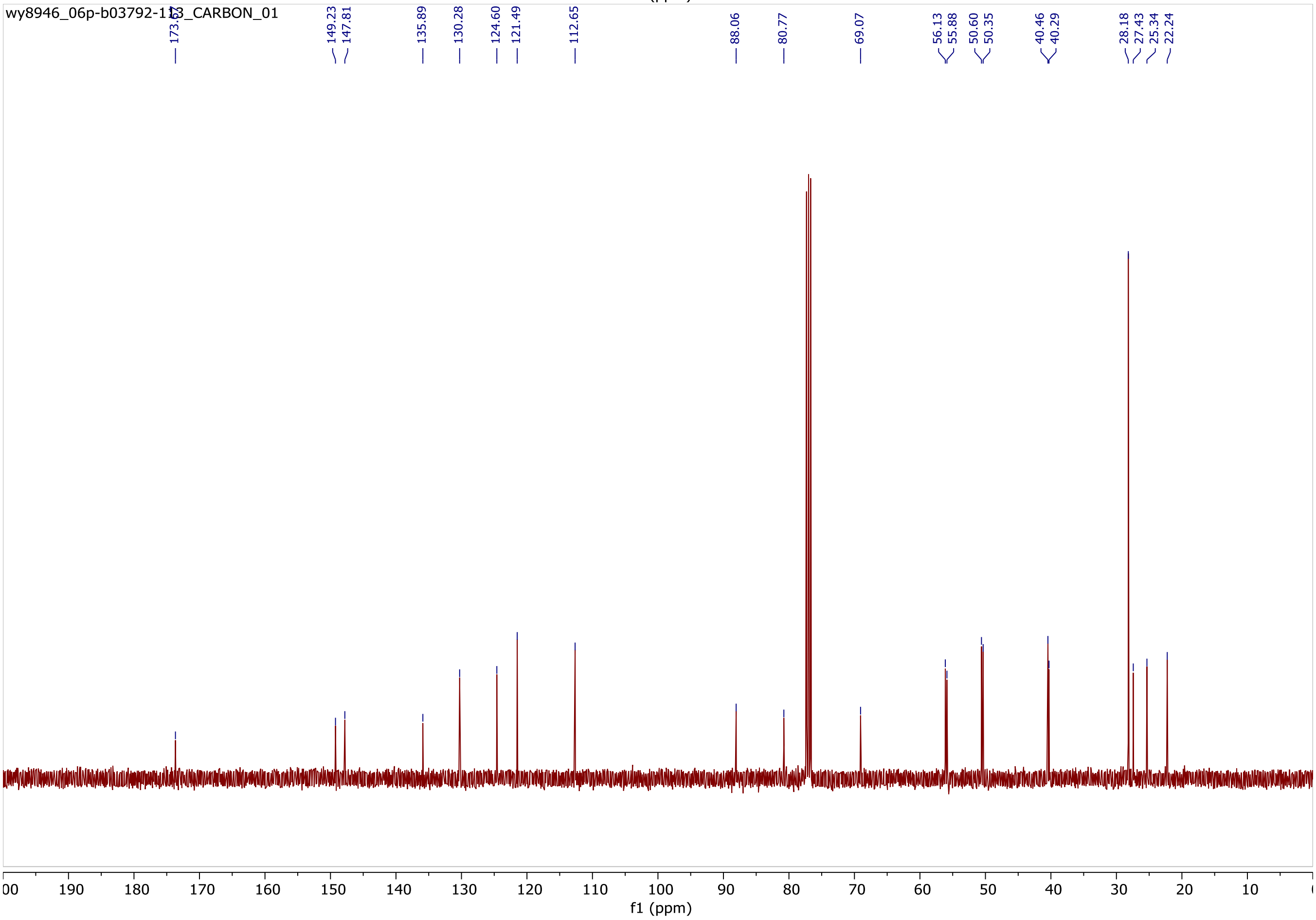

149.23 147.81
135.89
130.28
124.60
121.49
112.65
88.06
80.77
69.07
56.13 55.88
50.60 50.35
40.46 40.29
28.18 27.43 25.34 22.24
f1 (ppm)

CDCl$_3$

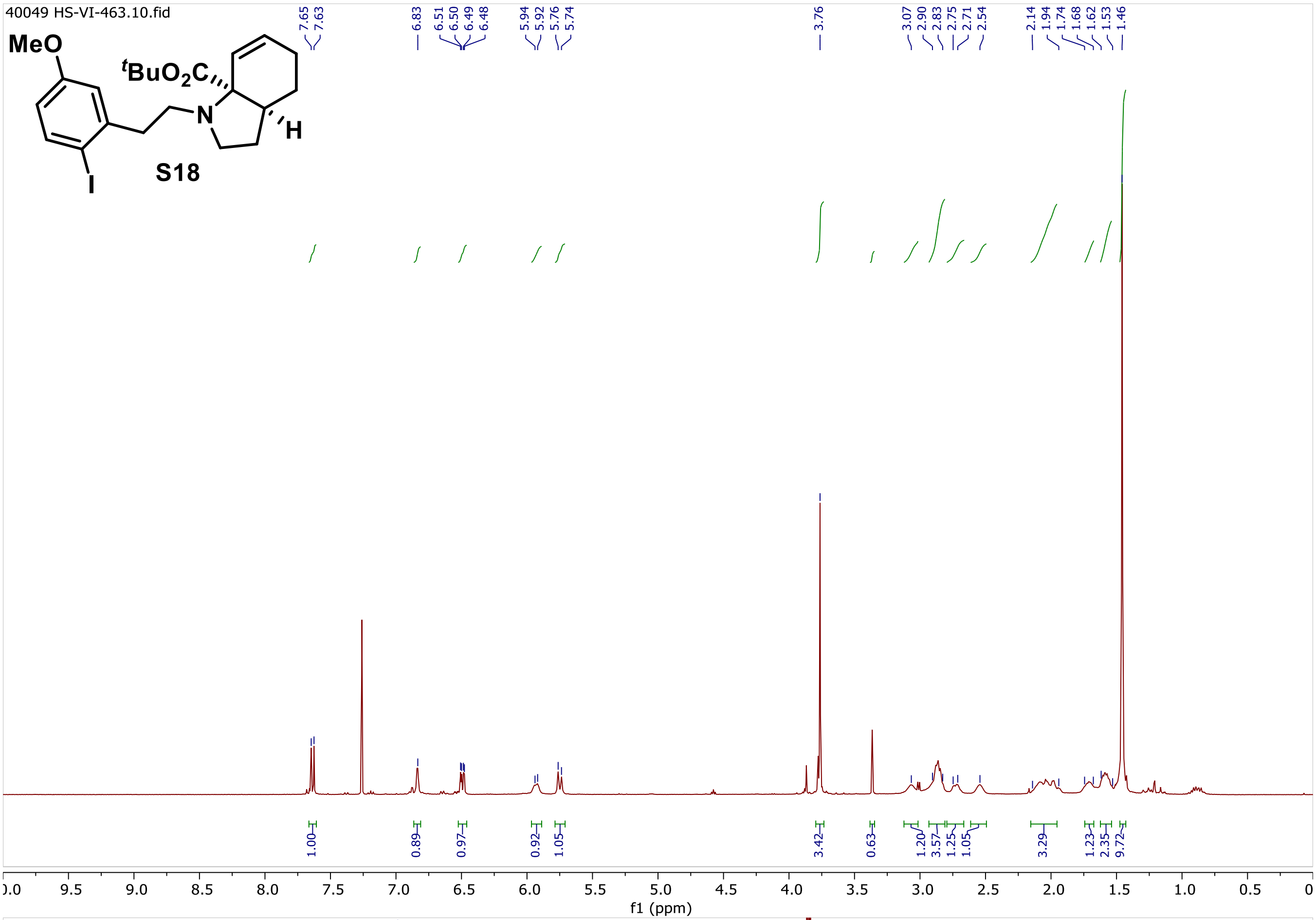

40049 HS-VI-463.10.fid
MeO
^{t}BuO$_2$C
N
H
I
S18
7.65 7.63 6.83 6.51 6.50 6.49 6.48 5.94 5.92 5.76 5.74 3.76 3.07 2.90 2.83 2.75 2.71 2.54 2.14 1.94 1.74 1.68 1.62 1.53 1.46
1.00 0.89 0.97 0.92 1.05 3.42 0.63 1.20 3.57 1.25 1.05 3.29 1.23 2.35 9.72
f1 (ppm)

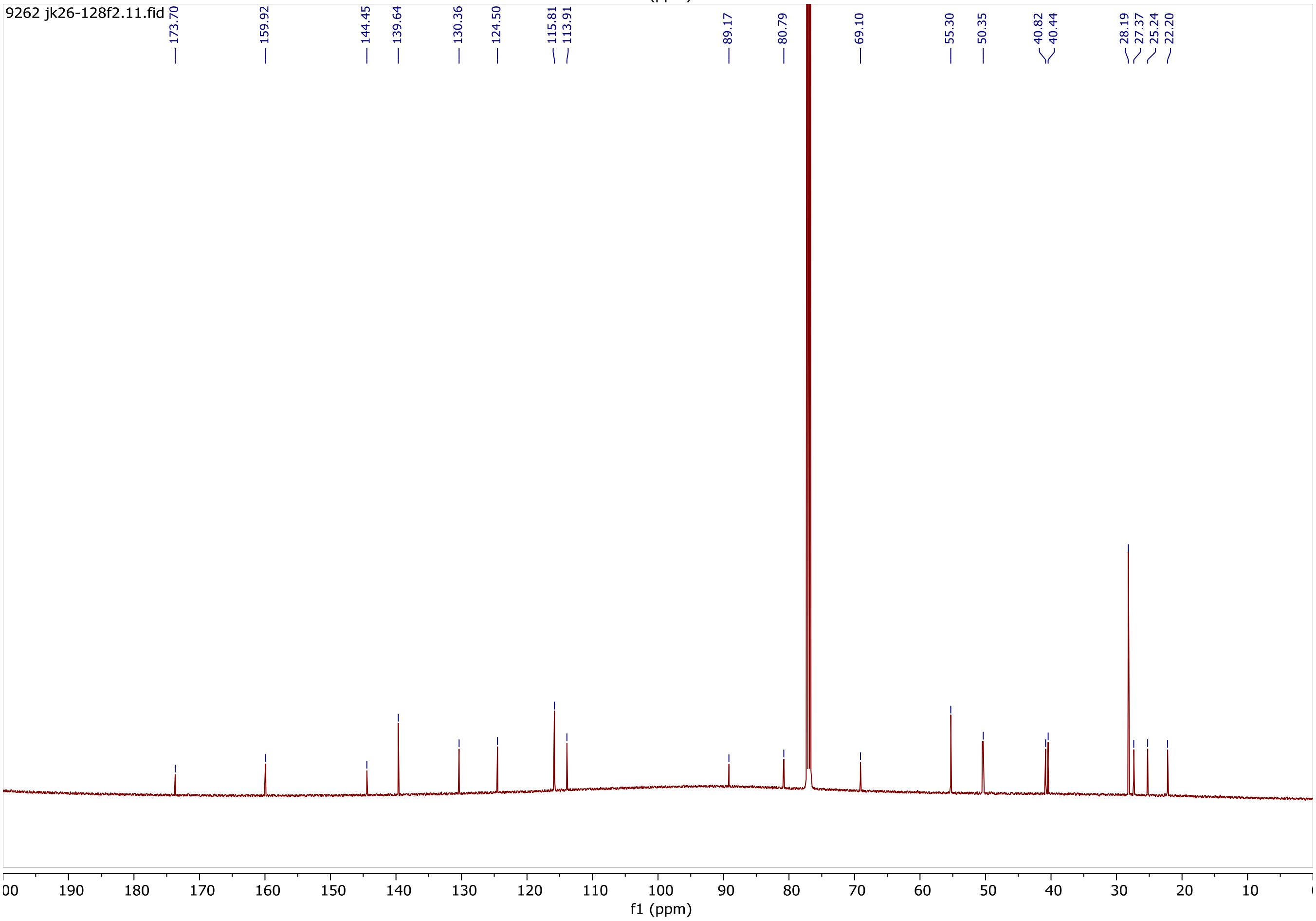

9262 jk26-128f2.11.fid
173.70 159.92 144.45 139.64 130.36 124.50 115.81 113.91 89.17 80.79 69.10 55.30 50.35 40.82 40.44 28.19 27.37 25.24 22.20
f1 (ppm)

CDCl$_3$

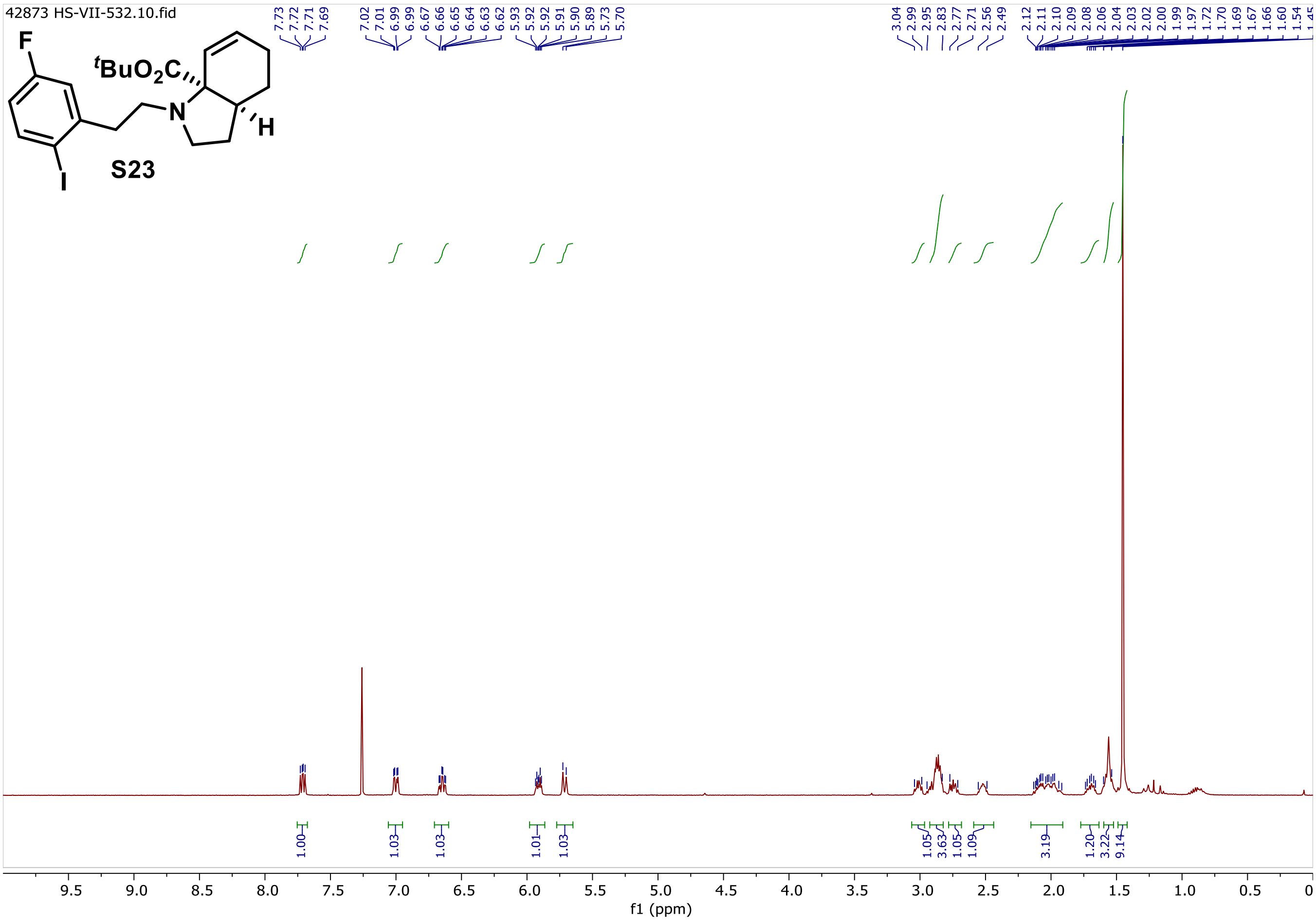
42873 HS-VII-532.10.fid
F
^{t}BuO$_2$C
N
H
I
S23
7.73 7.72 7.71 7.69 7.02 7.01 6.99 6.99 6.67 6.66 6.65 6.64 6.63 6.62 5.93 5.92 5.92 5.91 5.90 5.89 5.73 5.70 3.04 2.99 2.95 2.83 2.77 2.71 2.56 2.49 2.12 2.11 2.10 2.09 2.08 2.06 2.04 2.03 2.02 2.00 1.99 1.97 1.72 1.70 1.69 1.67 1.66 1.60 1.54
1.00 1.03 1.03 1.01 1.03 1.05 3.63 1.05 1.09 3.19 1.20 3.22 9.14
9.5 9.0 8.5 8.0 7.5 7.0 6.5 6.0 5.5 5.0 4.5 4.0 3.5 3.0 2.5 2.0 1.5 1.0 0.5
f1 (ppm)

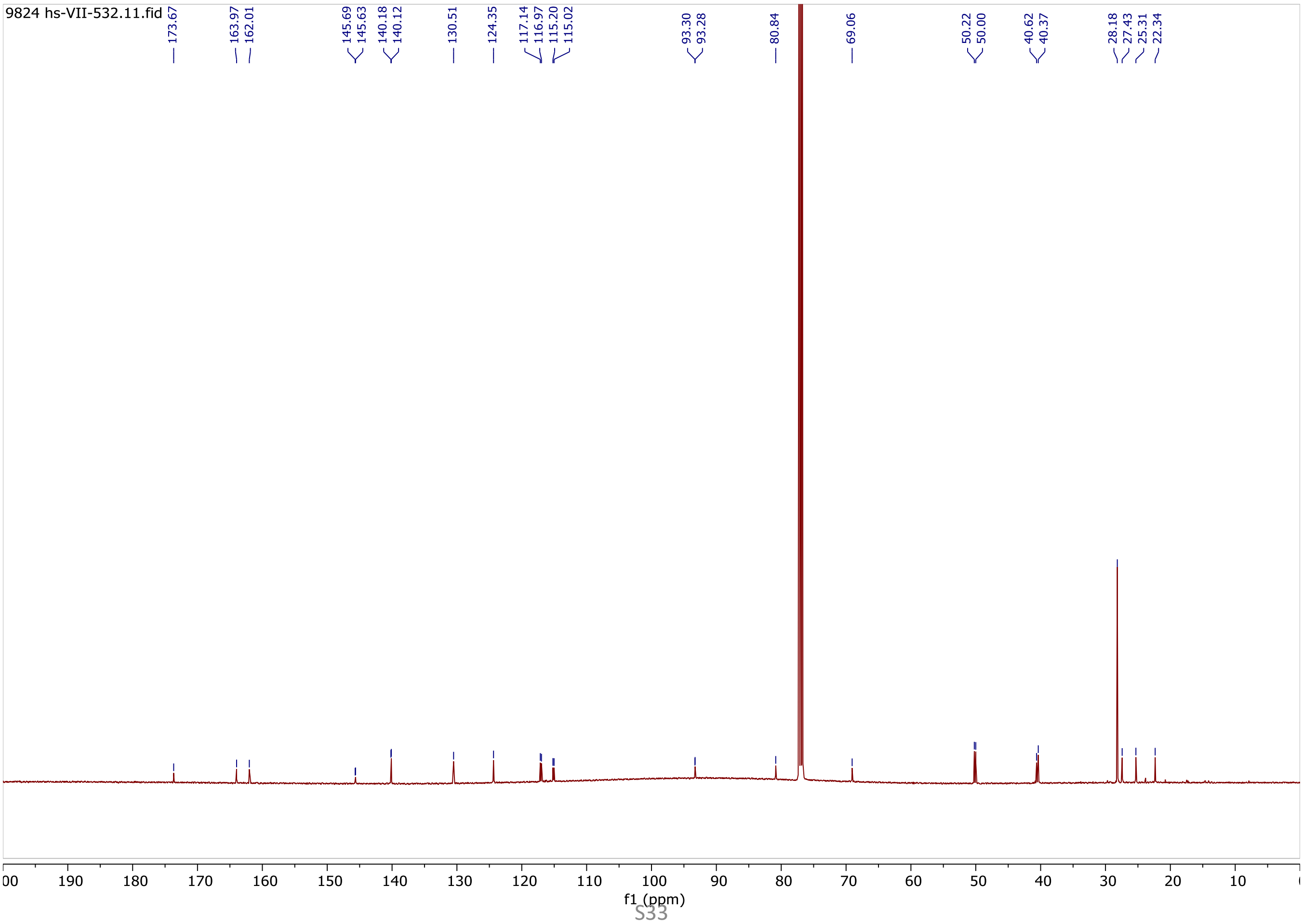
9824 hs-VII-532.11.fid
173.67 163.97 162.01 145.69 145.63 140.18 140.12 130.51 124.35 117.14 116.97 115.20 115.02 93.30 93.28 80.84 69.06 50.22 50.00 40.62 40.37 28.18 27.43 25.31 22.34
190 180 170 160 150 140 130 120 110 100 90 80 70 60 50 40 30 20 10
f1 (ppm)

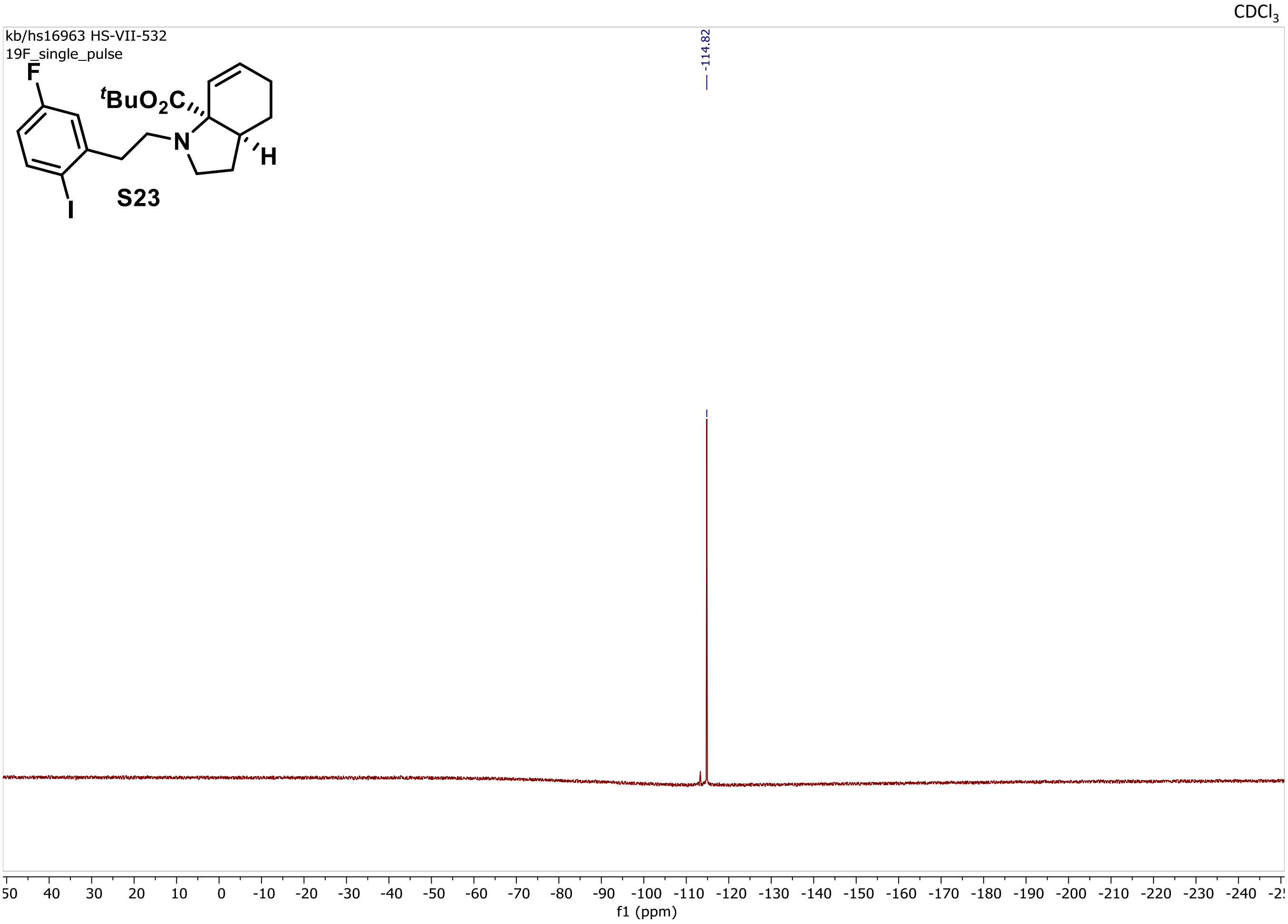
CDCl$_3$
kb/hs16963 HS-VII-532
19F_single_pulse
S23
-114.82
f1 (ppm)

CDCl$_3$

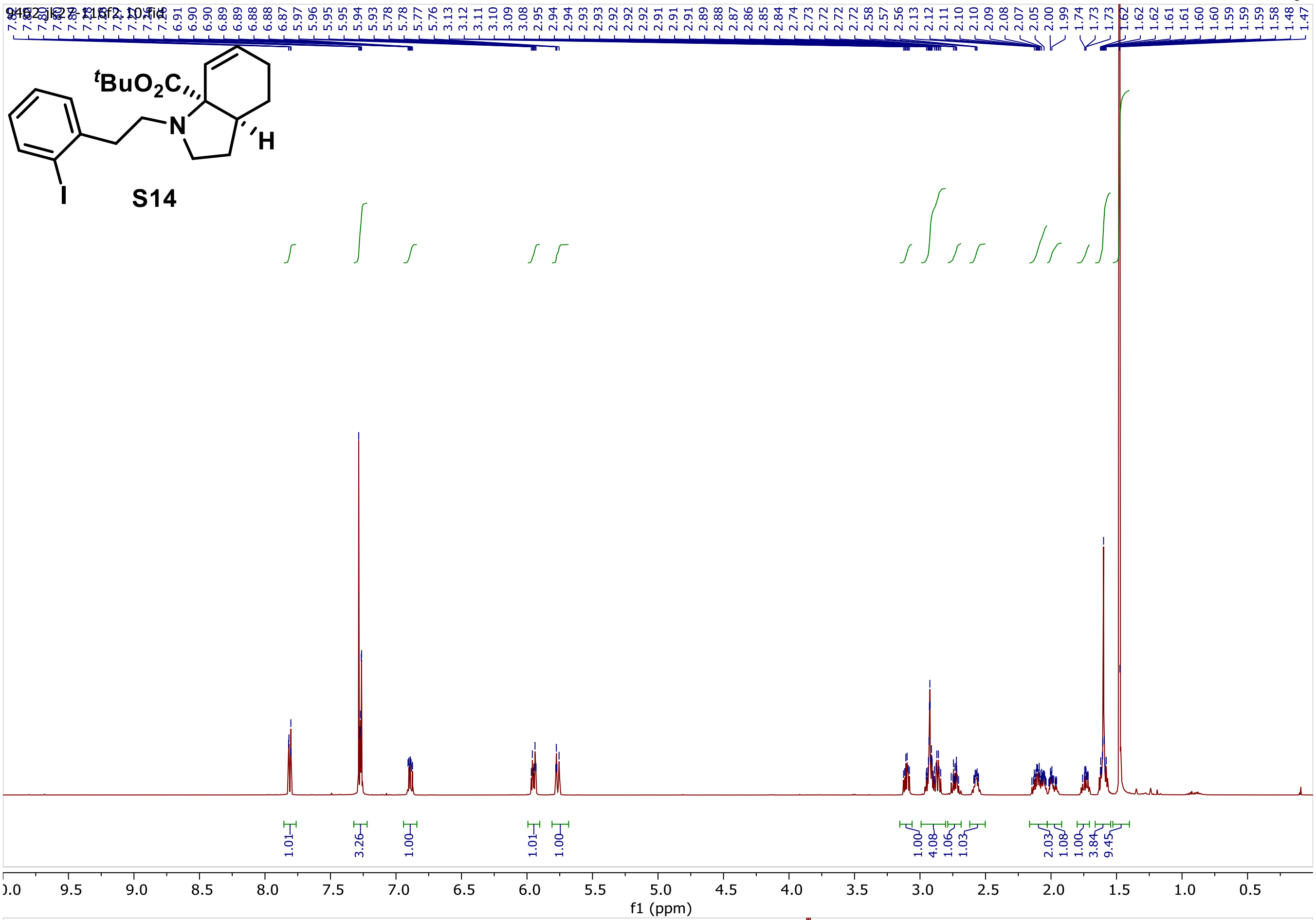

9462 jk27-116f2.10.fid
6.91
6.90
6.89
6.89
6.88
6.88
6.87
5.97
5.96
5.95
5.95
5.94
5.93
5.78
5.77
5.76
3.13
3.12
3.11
3.10
3.09
3.08
2.95
2.94
2.94
2.93
2.93
2.92
2.92
2.92
2.91
2.91
2.91
2.89
2.88
2.87
2.86
2.85
2.84
2.74
2.73
2.72
2.72
2.72
2.58
2.57
2.56
2.13
2.12
2.12
2.11
2.10
2.10
2.09
2.08
2.07
2.05
2.00
1.99
1.74
1.73
1.73
1.62
1.62
1.62
1.61
1.61
1.60
1.59
1.59
1.58
1.48
1.47
^{t}BuO$_2$C
N
H
I
S14
1.01
3.26
1.00
1.01
1.00
1.00
4.08
1.06
1.03
2.03
1.08
1.00
3.84
9.45
f1 (ppm)

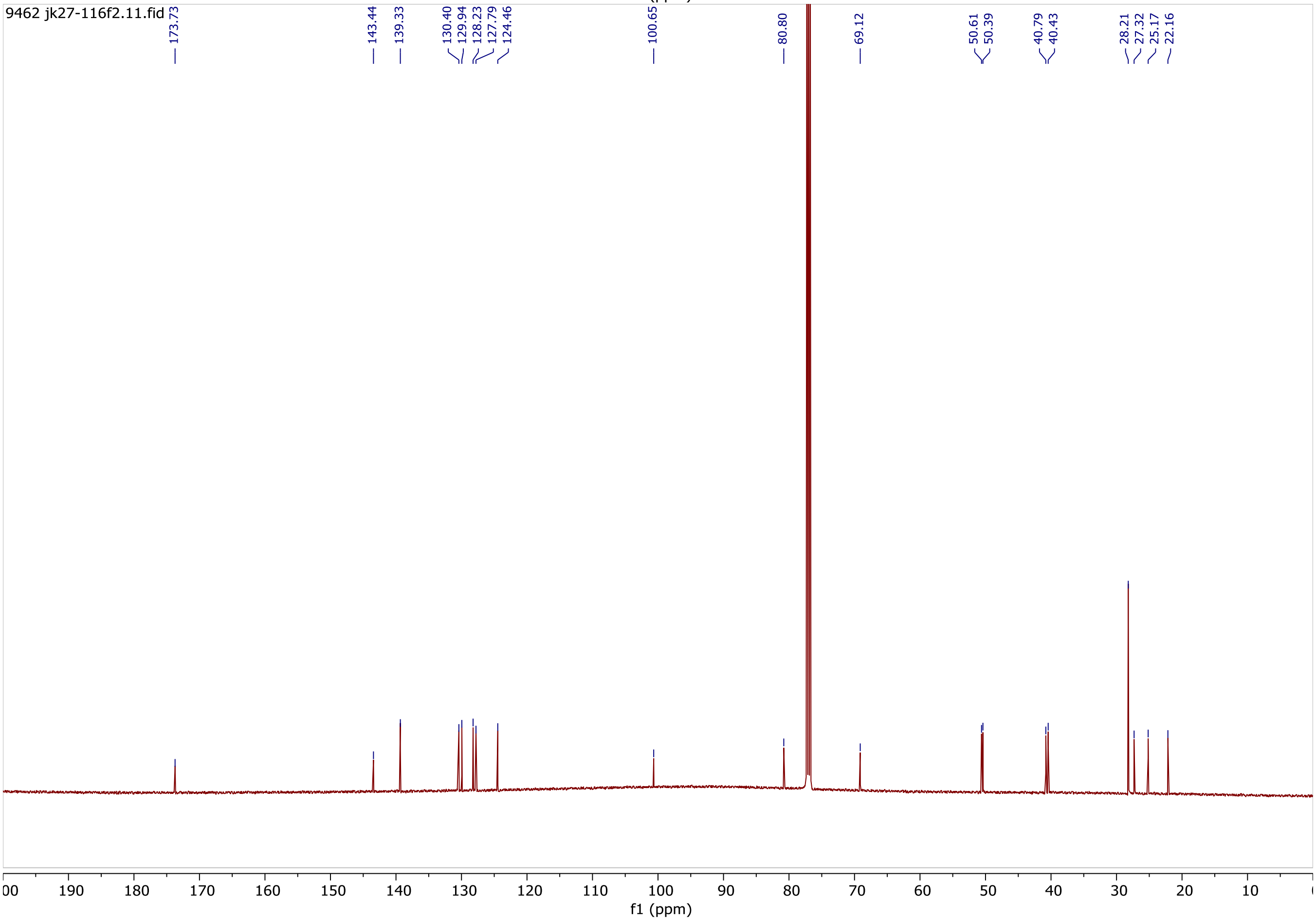

9462 jk27-116f2.11.fid
173.73
143.44
139.33
130.40
129.94
128.23
127.79
124.46
100.65
80.80
69.12
50.61
50.39
40.79
40.43
28.21
27.32
25.17
22.16
f1 (ppm)

CDCl$_3$

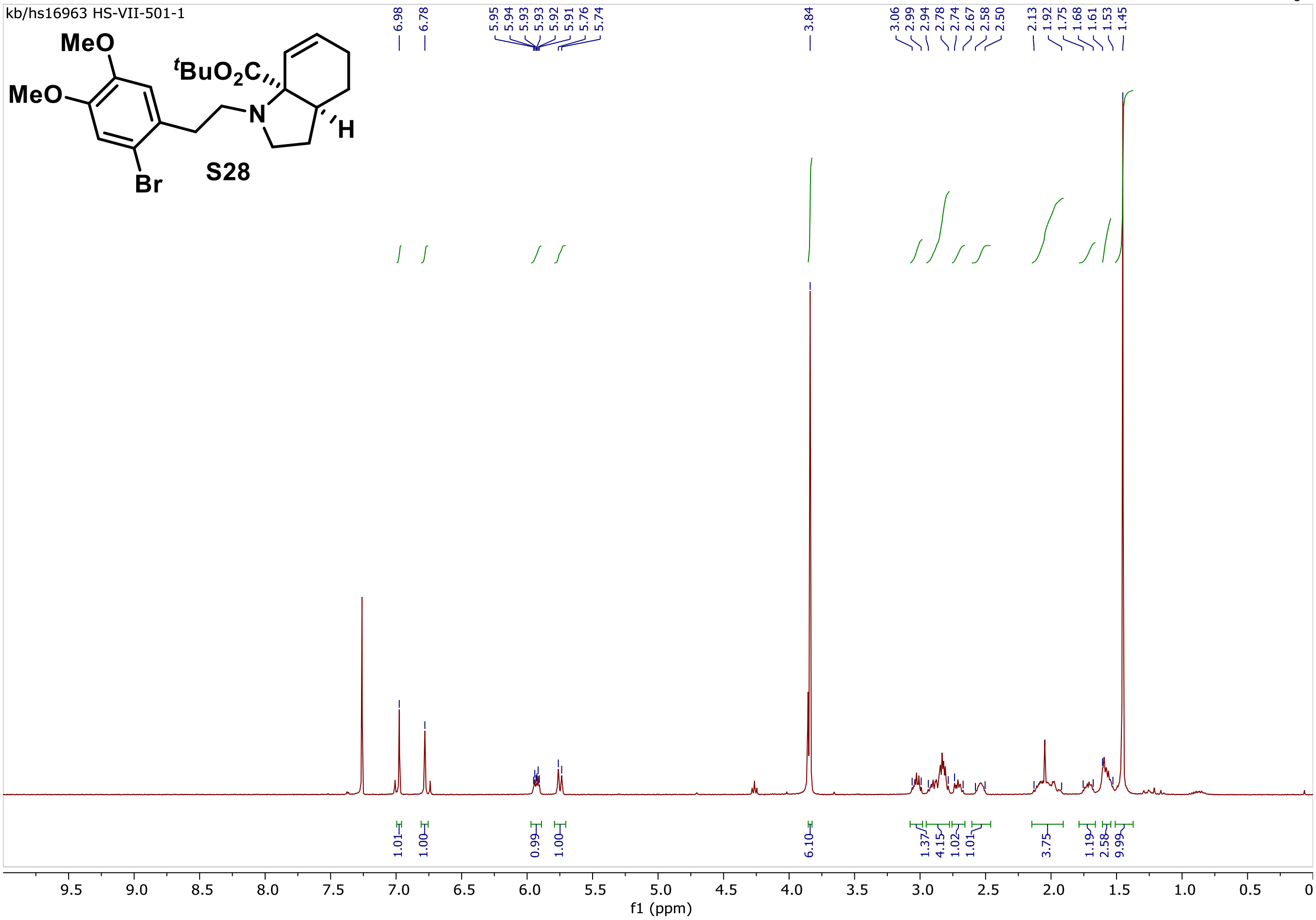

kb/hs16963 HS-VII-501-1
MeO
MeO
^{t}BuO$_2$C
N
H
Br
S28
6.98
6.78
5.95
5.94
5.93
5.93
5.92
5.91
5.76
5.74
3.84
3.06
2.99
2.94
2.78
2.74
2.67
2.58
2.50
2.13
1.92
1.75
1.68
1.61
1.53
1.45
1.01
1.00
0.99
1.00
6.10
1.37
4.15
1.02
1.01
3.75
1.19
2.58
9.99
f1 (ppm)

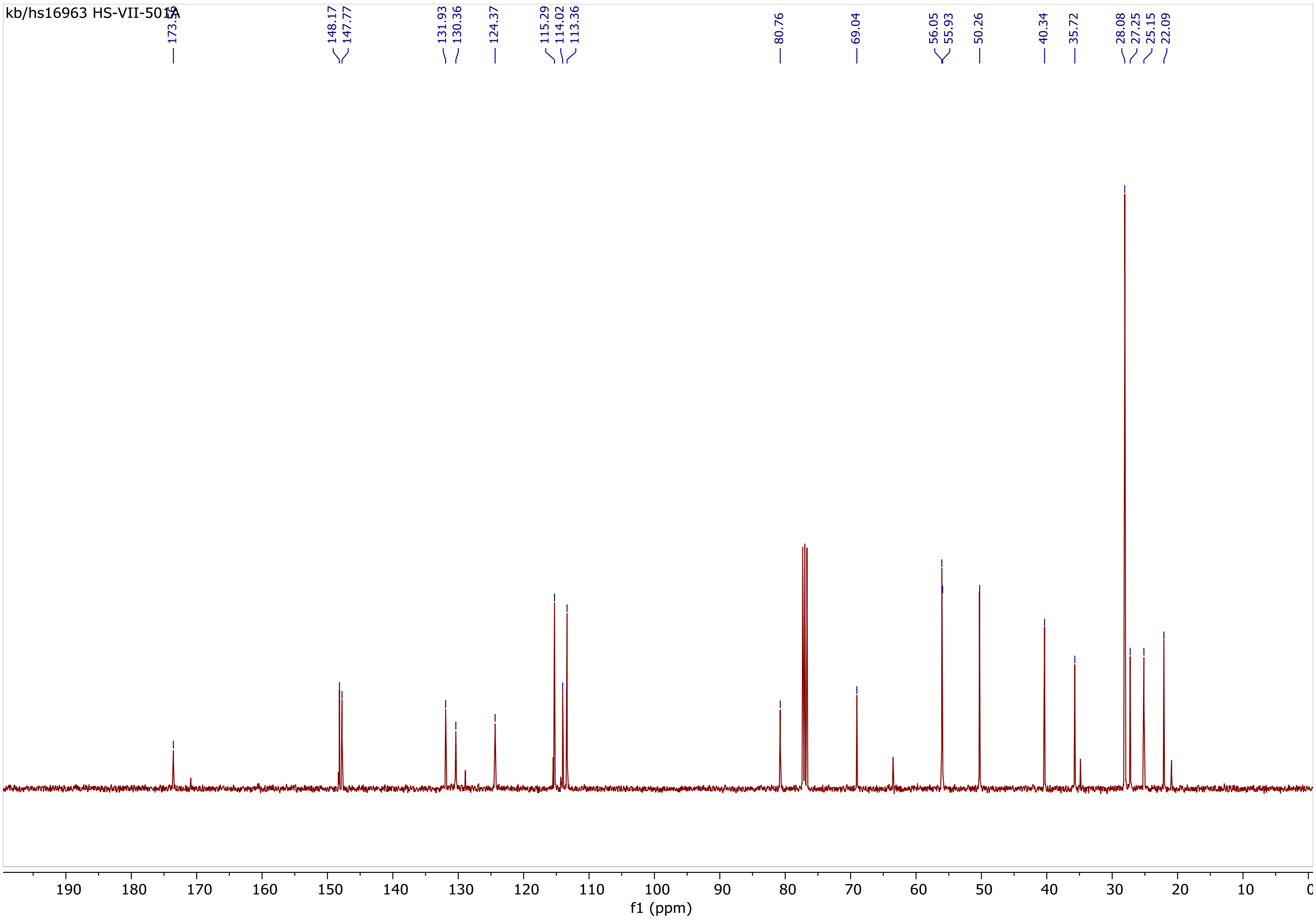

kb/hs16963 HS-VII-501A
173.39
148.17
147.77
131.93
130.36
124.37
115.29
114.02
113.36
80.76
69.04
56.05
55.93
50.26
40.34
35.72
28.08
27.25
25.15
22.09
f1 (ppm)

CDCl$_3$

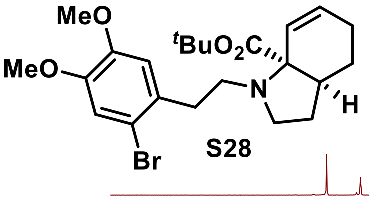
S28

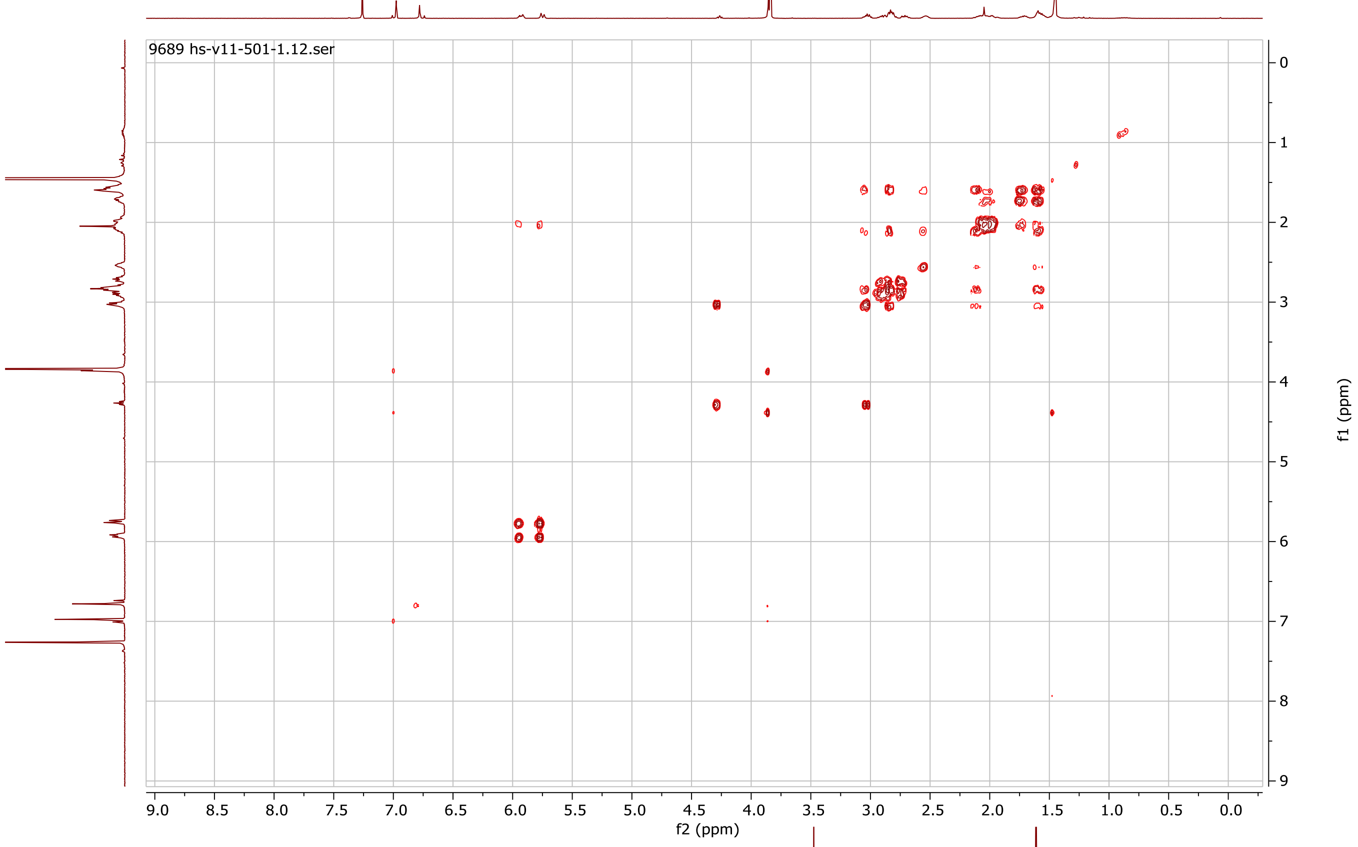
9689 hs-v11-501-1.12.ser
f2 (ppm)
f1 (ppm)

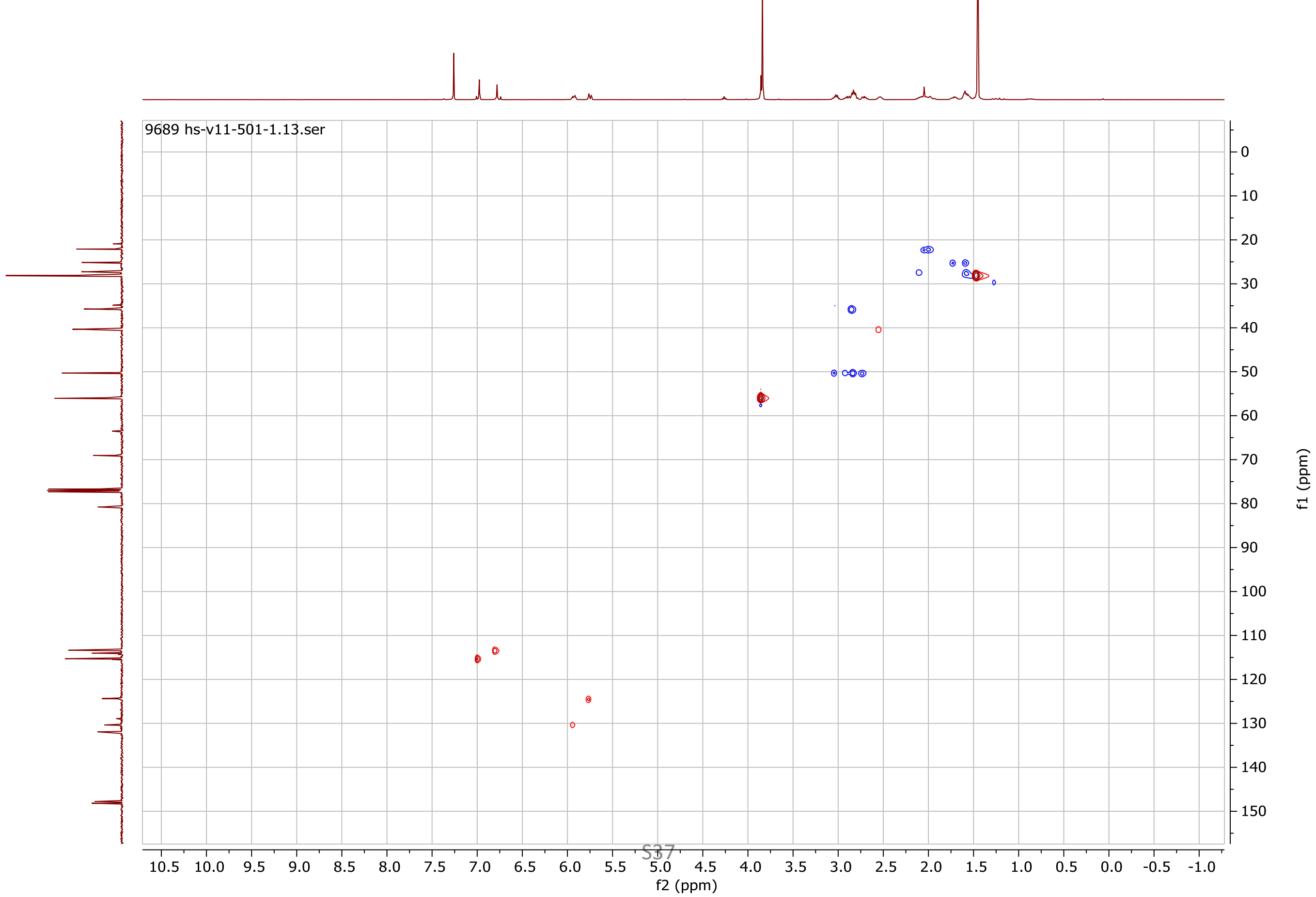
9689 hs-v11-501-1.13.ser
f2 (ppm)
f1 (ppm)

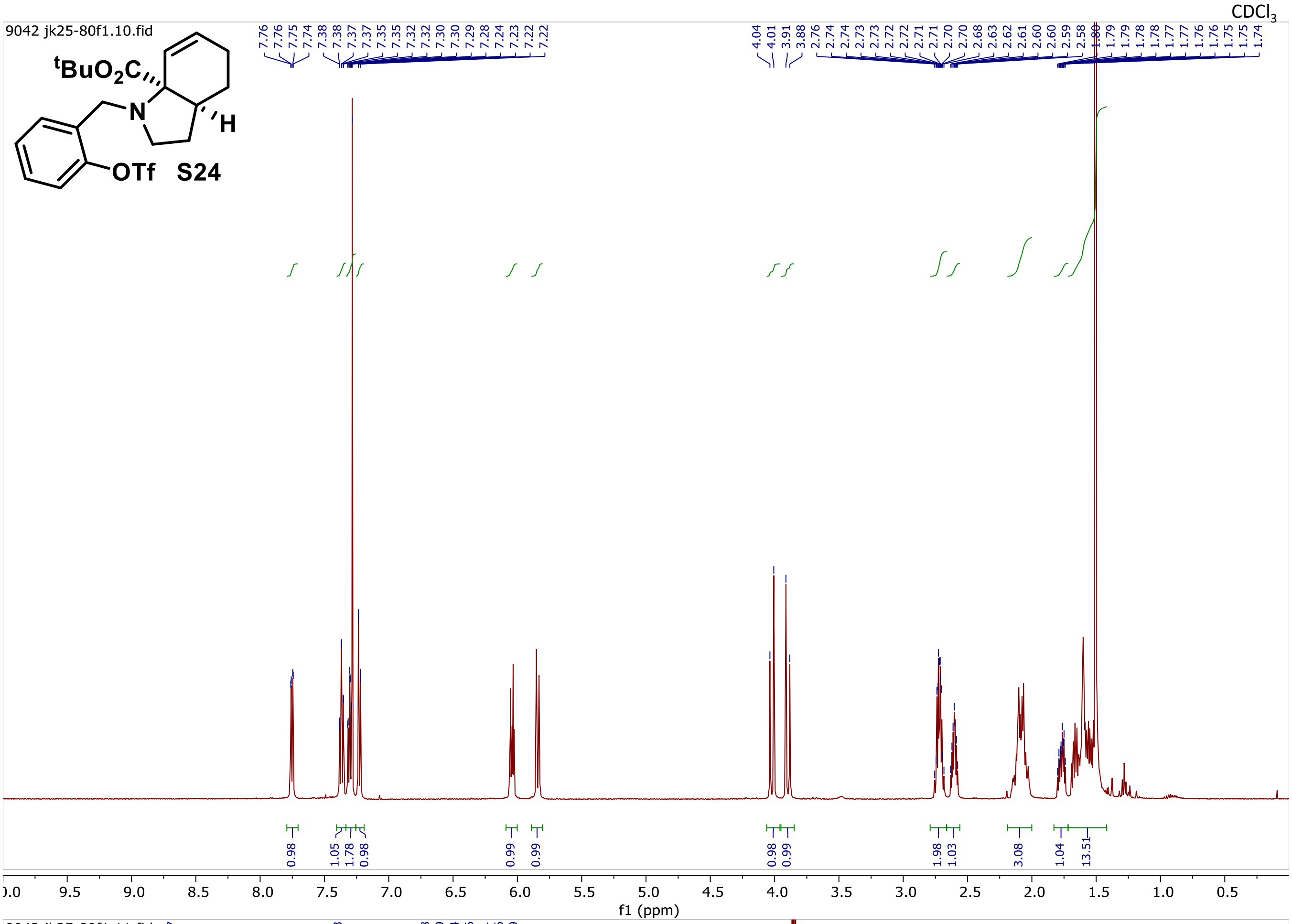
CDCl3
9042 jk25-80f1.10.fid
tBuO2C
N
H
OTf
S24
7.76 7.76 7.75 7.74 7.38 7.38 7.37 7.37 7.35 7.35 7.32 7.32 7.30 7.30 7.29 7.28 7.24 7.23 7.22 7.22
4.04 4.01 3.91 3.88 2.76 2.74 2.74 2.73 2.73 2.72 2.72 2.71 2.71 2.70 2.70 2.68 2.63 2.62 2.61 2.60 2.60 2.59 2.58 1.80 1.79 1.79 1.78 1.78 1.77 1.77 1.76 1.76 1.75 1.75 1.74
0.98 1.05 1.78 0.98 0.99 0.99 0.98 0.99 1.98 1.03 3.08 1.04 13.51
f1 (ppm)

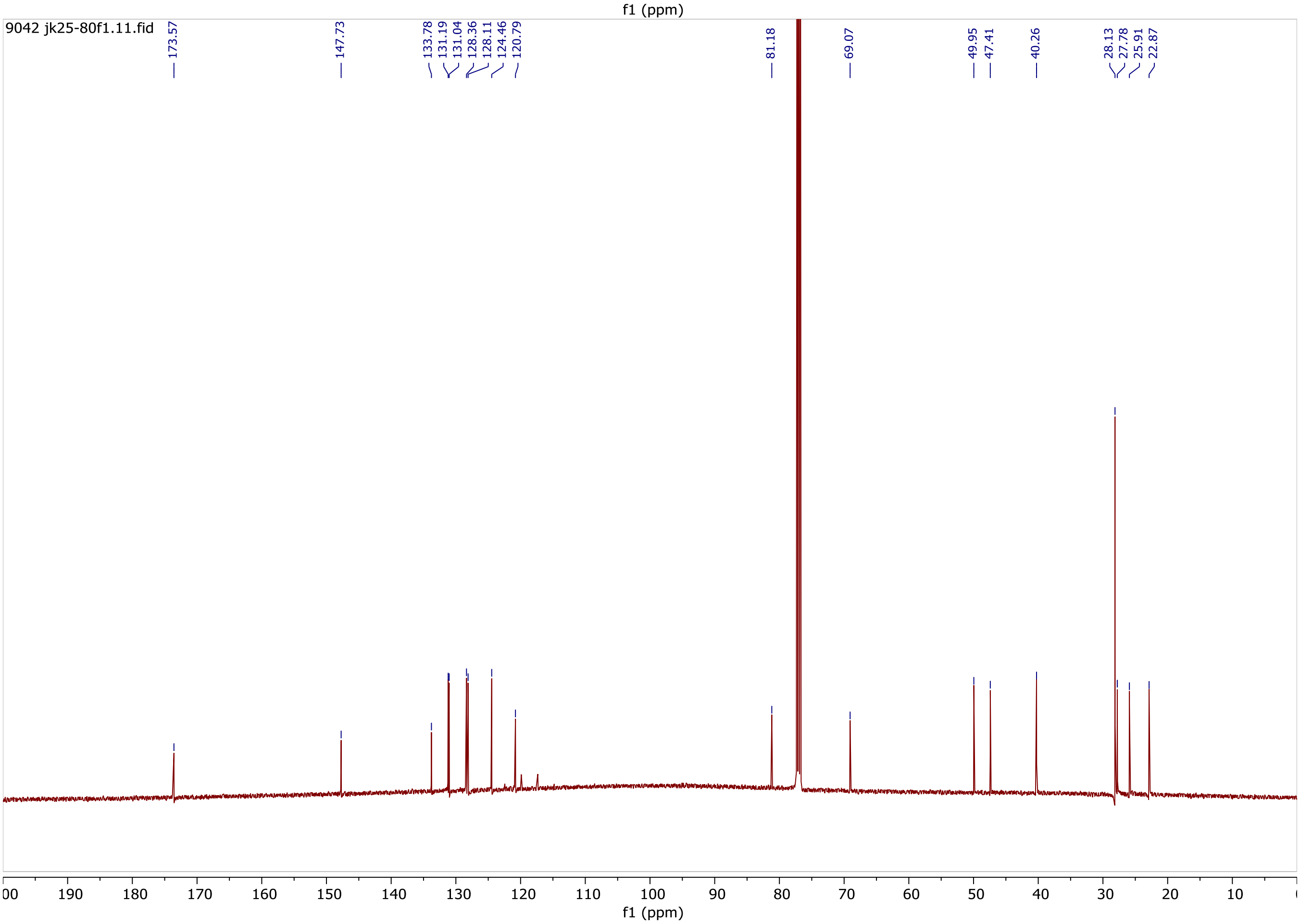
9042 jk25-80f1.11.fid
173.57
147.73
133.78 131.19 131.04 128.36 128.11 124.46 120.79
81.18
69.07
49.95 47.41
40.26
28.13 27.78 25.91 22.87
f1 (ppm)

$CDCl_3$

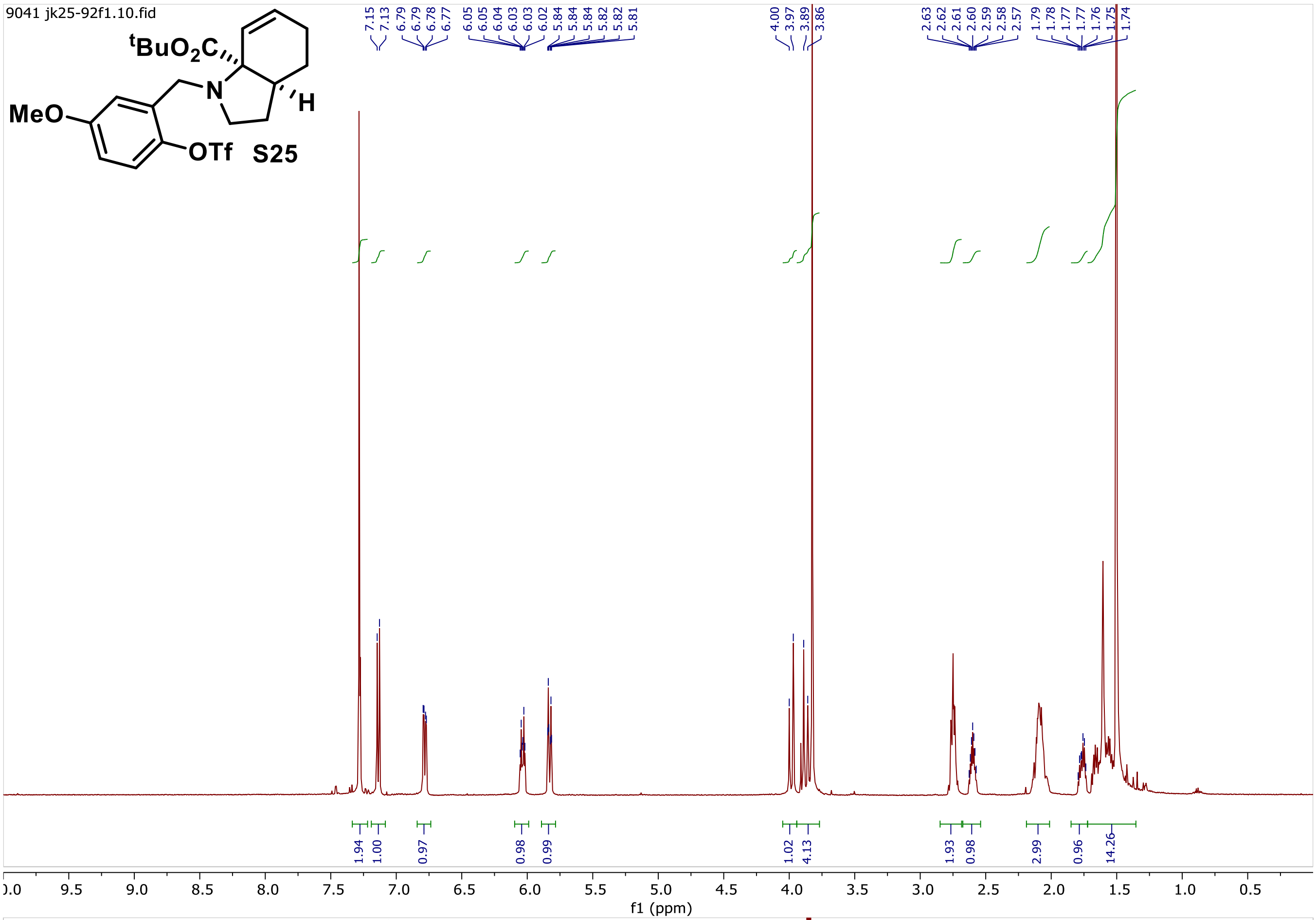
9041 jk25-92f1.10.fid
tBuO2C
MeO
N
H
OTf
S25
7.15 7.13 6.79 6.79 6.78 6.77 6.05 6.05 6.04 6.03 6.03 6.02 5.84 5.84 5.84 5.82 5.82 5.81
4.00 3.97 3.89 3.86
2.63 2.62 2.61 2.60 2.59 2.58 2.57 1.79 1.78 1.77 1.77 1.76 1.75 1.74
1.94 1.00 0.97 0.98 0.99 1.02 4.13 1.93 0.98 2.99 0.96 14.26
f1 (ppm)

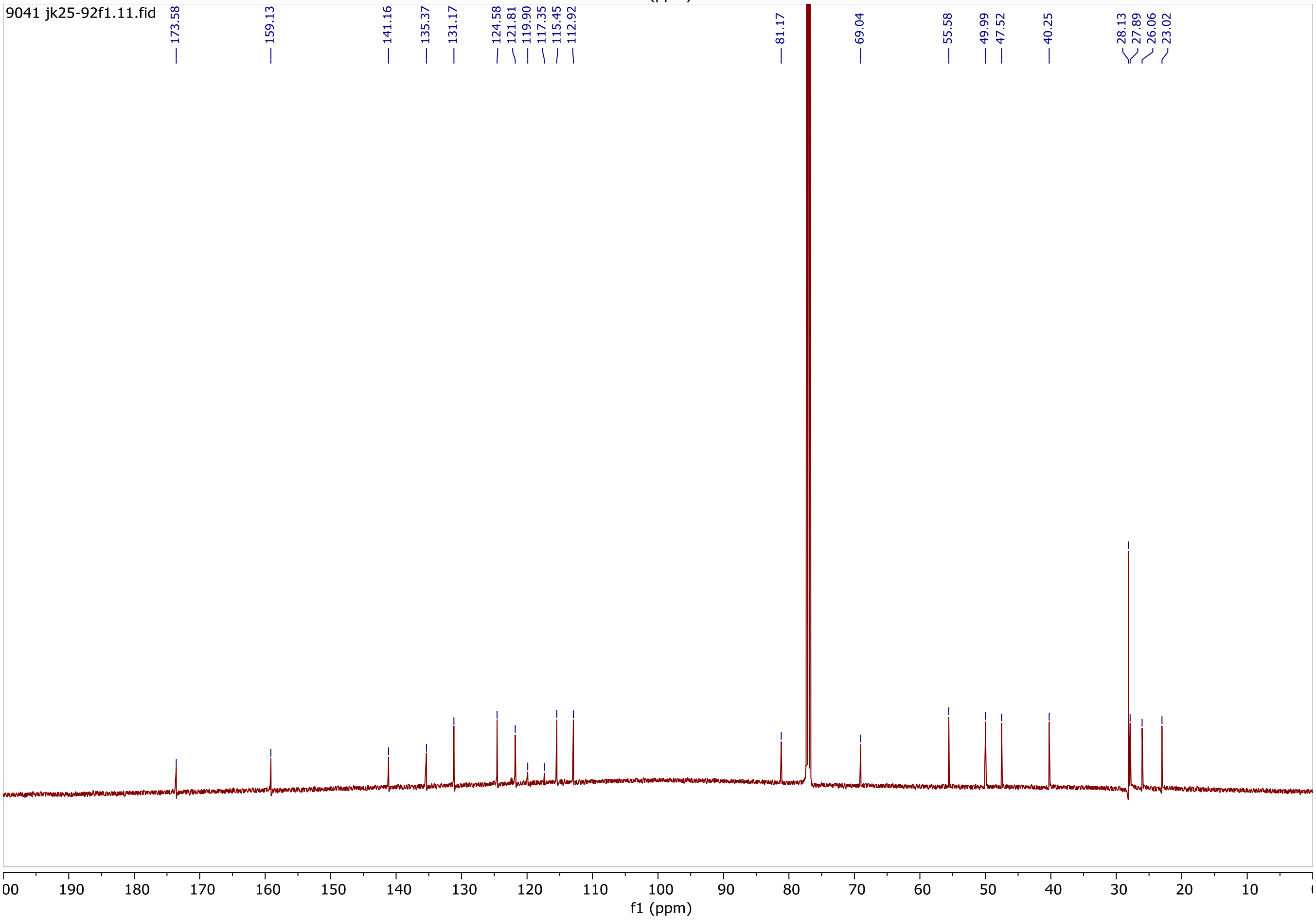
9041 jk25-92f1.11.fid
173.58 159.13 141.16 135.37 131.17 124.58 121.81 119.90 117.35 115.45 112.92 81.17 69.04 55.58 49.99 47.52 40.25 28.13 27.89 26.06 23.02
f1 (ppm)

$CDCl_3$

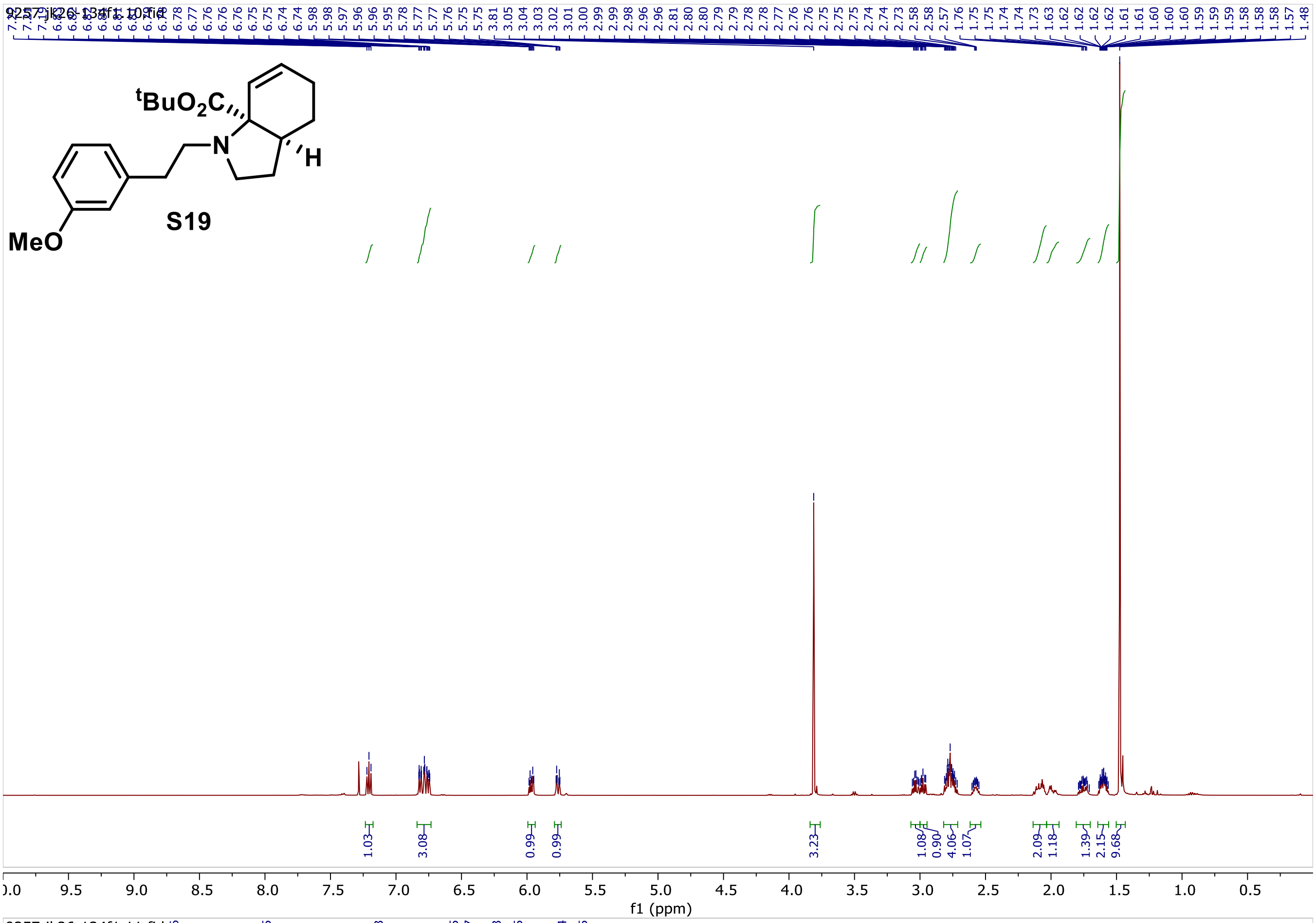
9257 jk26-134f1.10.fid
S19
1.03
3.08
0.99
0.99
3.23
1.08
0.90
4.06
1.07
2.09
1.18
1.39
2.15
9.68
f1 (ppm)

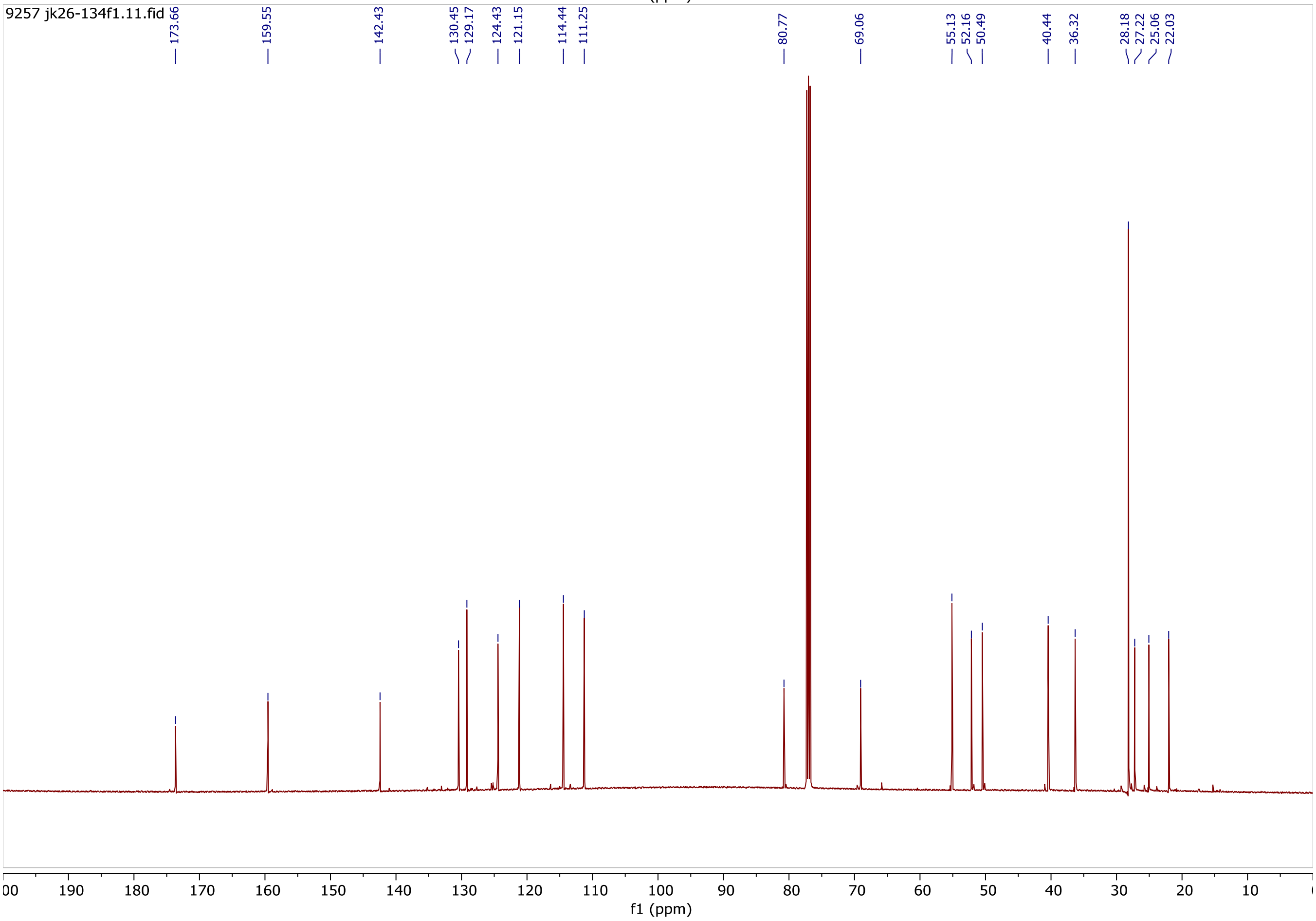
9257 jk26-134f1.11.fid
173.66
159.55
142.43
130.45
129.17
124.43
121.15
114.44
111.25
80.77
69.06
55.13
52.16
50.49
40.44
36.32
28.18
27.22
25.06
22.03
f1 (ppm)

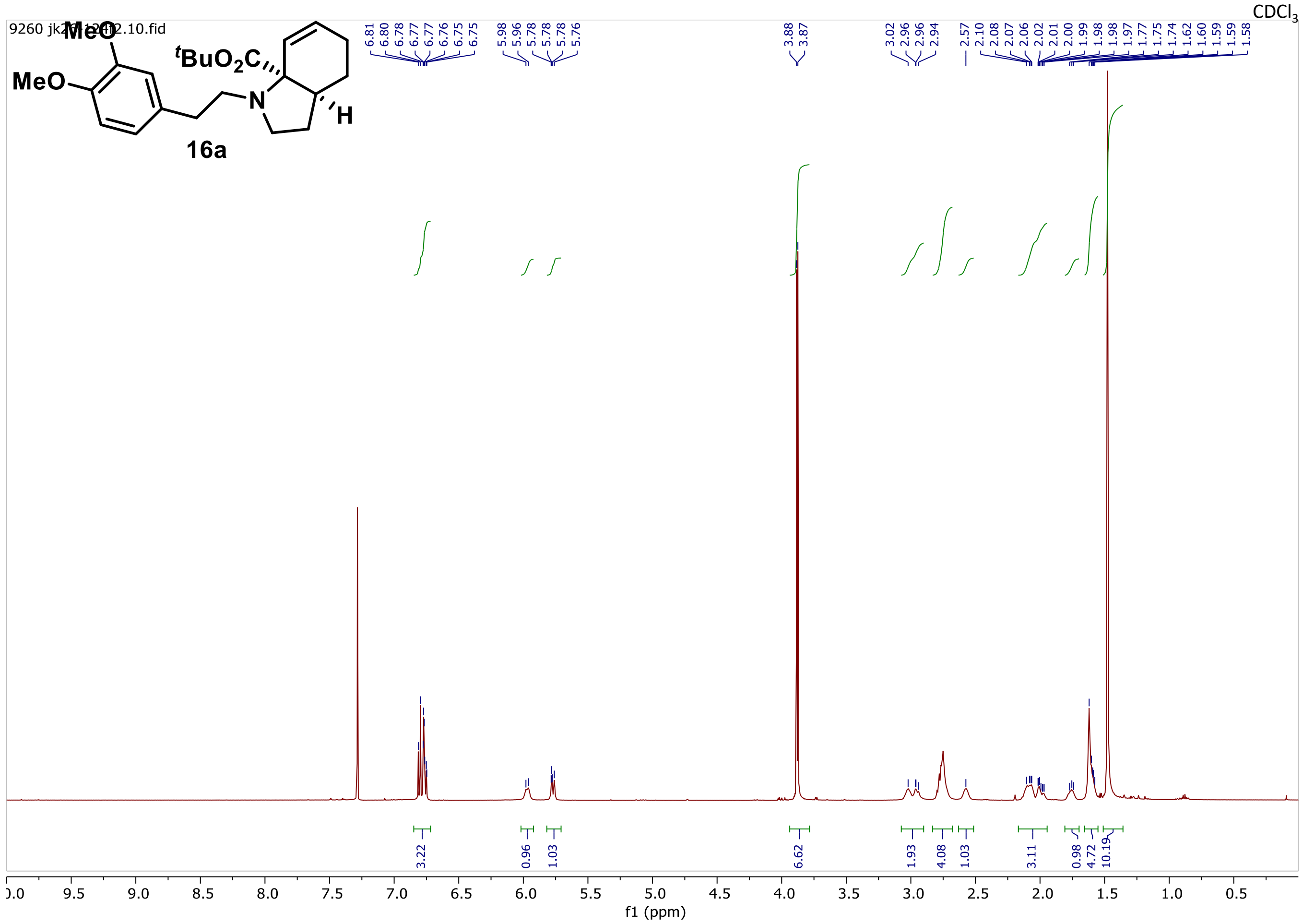
CDCl3
9260 jk26-124f2.10.fid
16a
f1 (ppm)

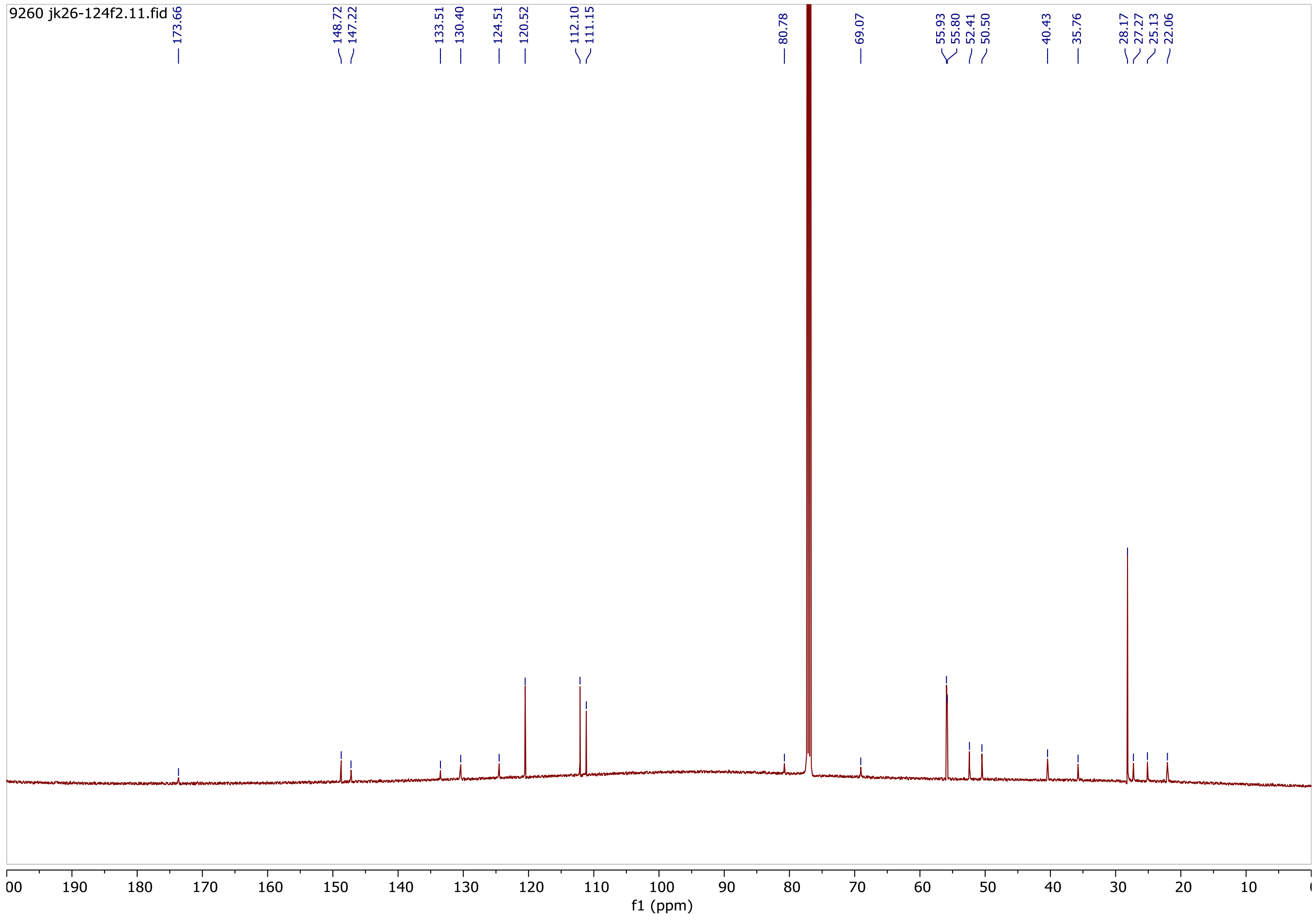
9260 jk26-124f2.11.fid
173.66
148.72
147.22
133.51
130.40
124.51
120.52
112.10
111.15
80.78
69.07
55.93
55.80
52.41
50.50
40.43
35.76
28.17
27.27
25.13
22.06
f1 (ppm)

CDCl$_3$

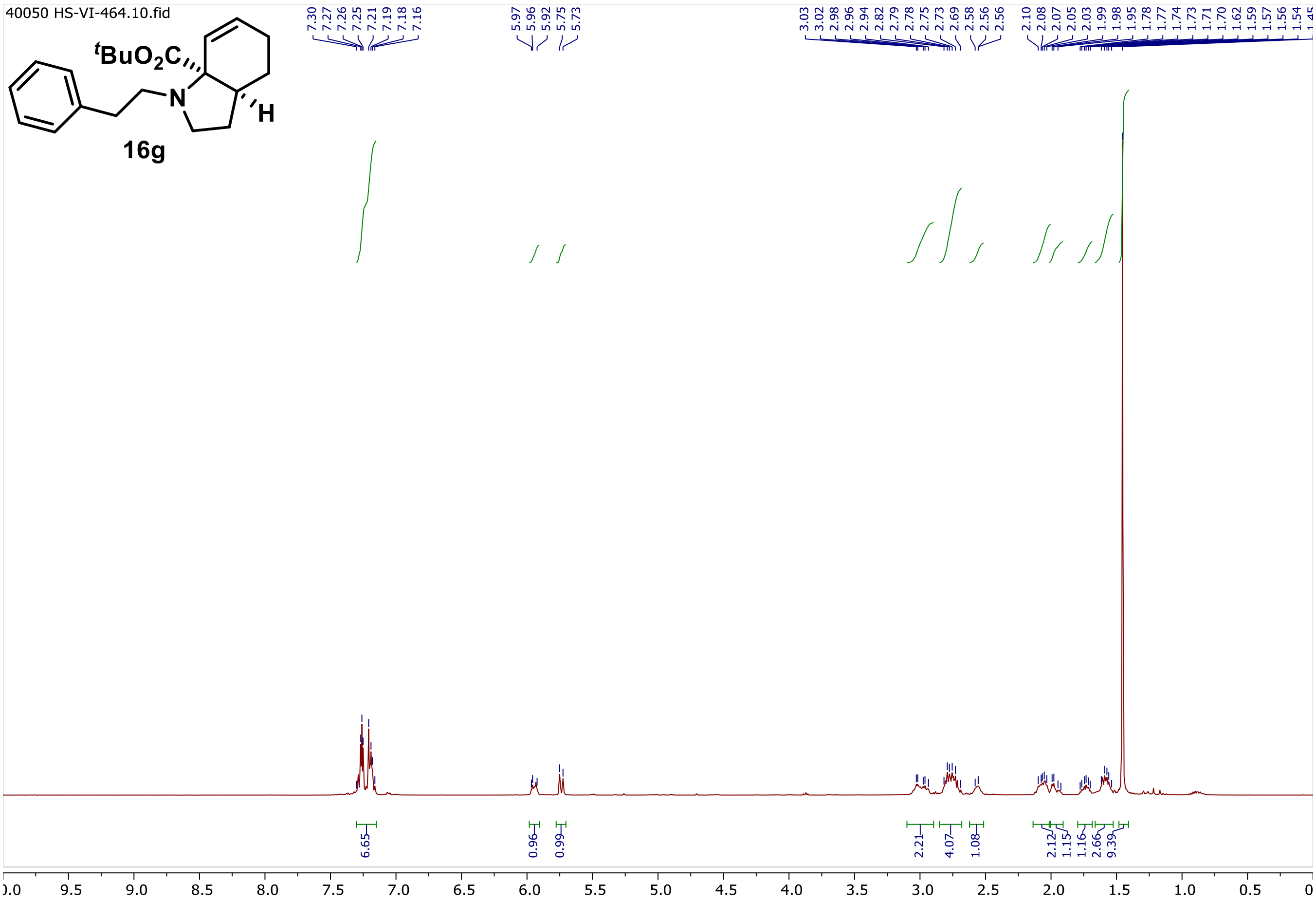
40050 HS-VI-464.10.fid
^{t}BuO$_2$C
N
H
16g
7.30
7.27
7.26
7.25
7.21
7.19
7.18
7.16
5.97
5.96
5.92
5.75
5.73
3.03
3.02
2.98
2.96
2.94
2.82
2.79
2.78
2.75
2.73
2.69
2.58
2.56
2.56
2.10
2.08
2.07
2.05
2.03
1.99
1.98
1.95
1.78
1.77
1.74
1.73
1.71
1.70
1.62
1.59
1.57
1.56
1.54
[illegible]
6.65
0.96
0.99
2.21
4.07
1.08
2.12
1.15
1.16
2.66
9.39
f1 (ppm)

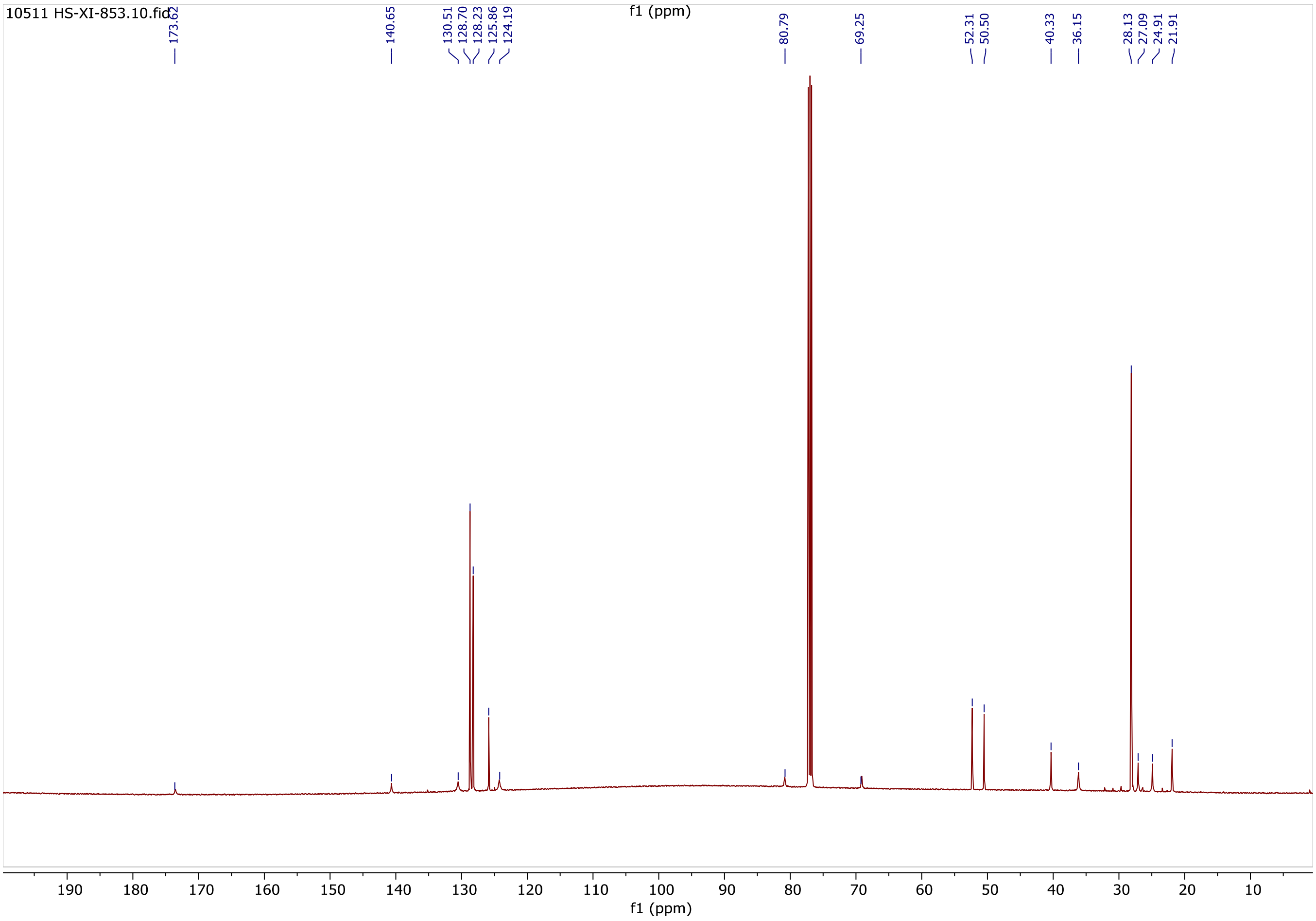
10511 HS-XI-853.10.fid
173.62
140.65
130.51
128.70
128.23
125.86
124.19
80.79
69.25
52.31
50.50
40.33
36.15
28.13
27.09
24.91
21.91
f1 (ppm)

CDCl$_3$

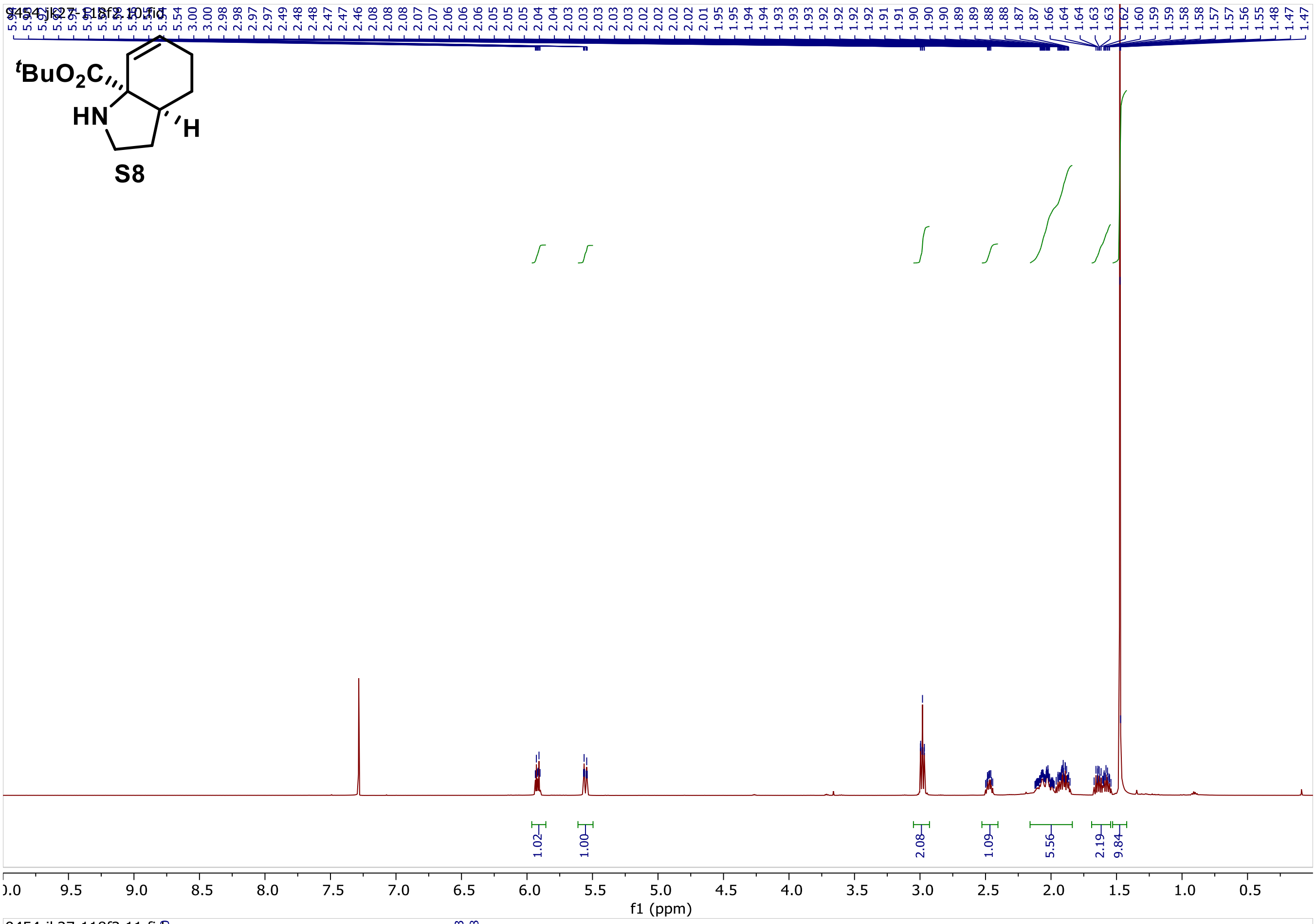

9454 jk27-118f2.10.fid
^{t}BuO$_2$C
HN
H
S8
1.02
1.00
2.08
1.09
5.56
2.19
9.84
f1 (ppm)

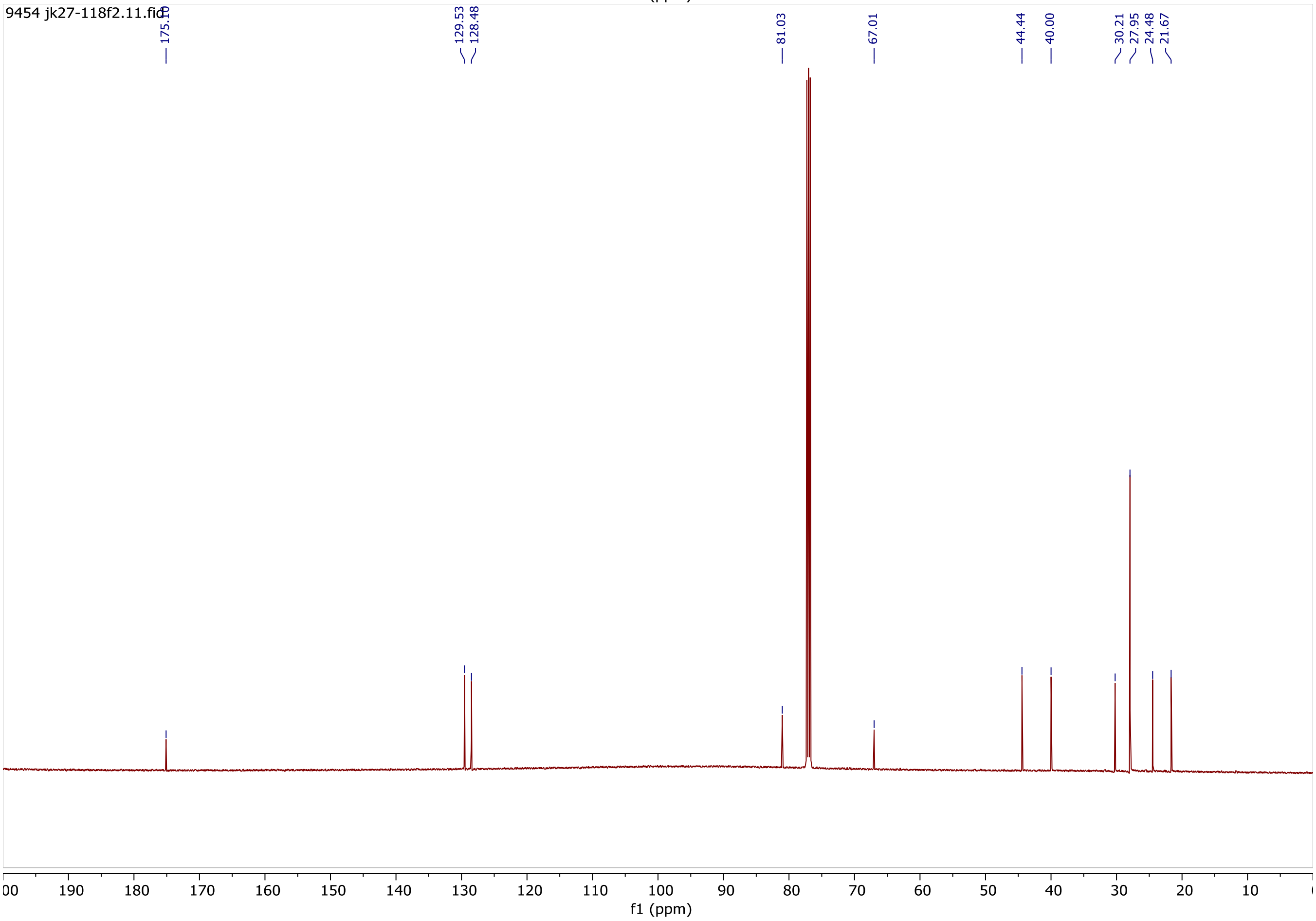

9454 jk27-118f2.11.fid
175.10
129.53
128.48
81.03
67.01
44.44
40.00
30.21
27.95
24.48
21.67
f1 (ppm)

CDCl$_3$

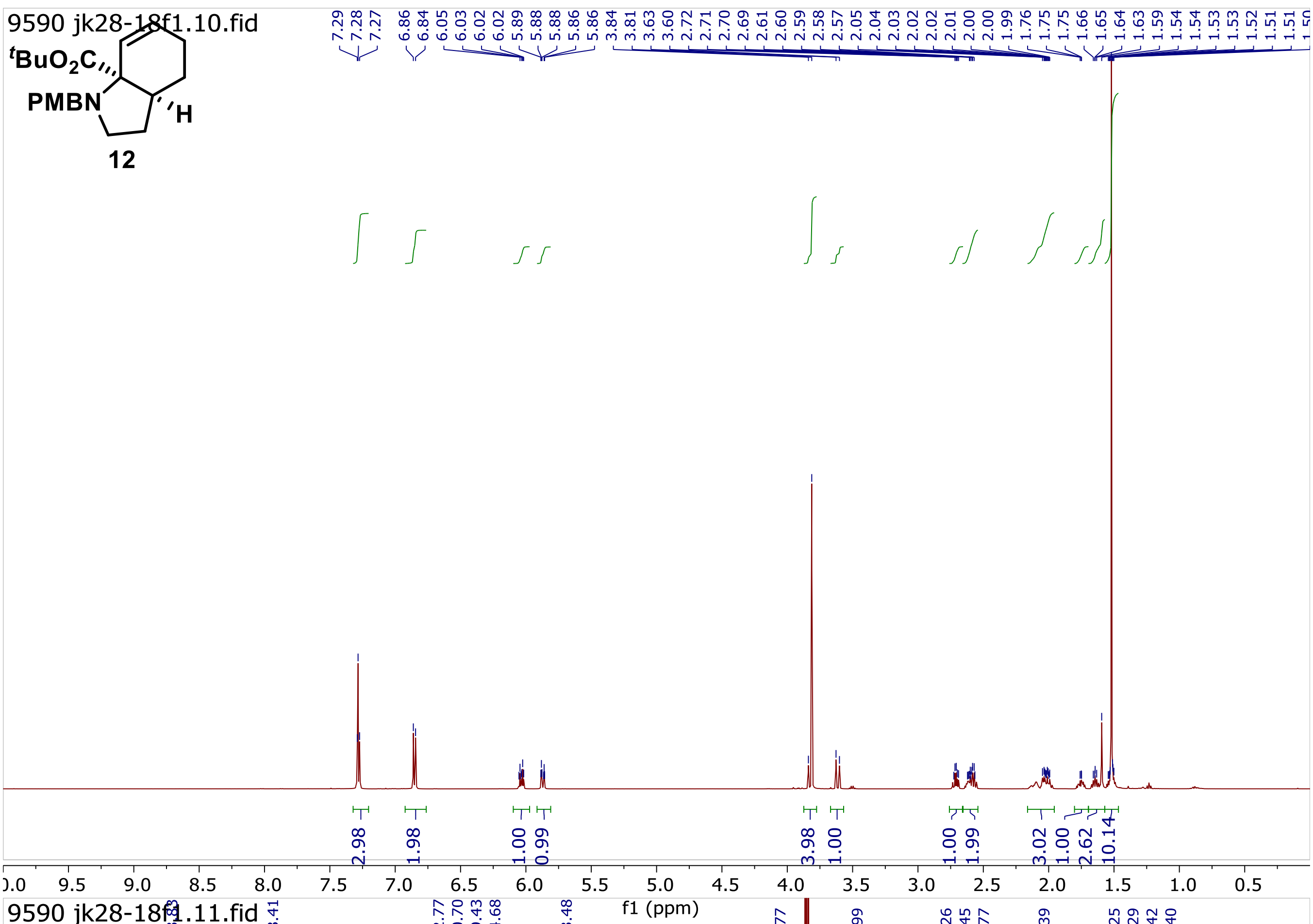

9590 jk28-18f1.10.fid
^{t}BuO$_2$C
PMBN
H
12
f1 (ppm)

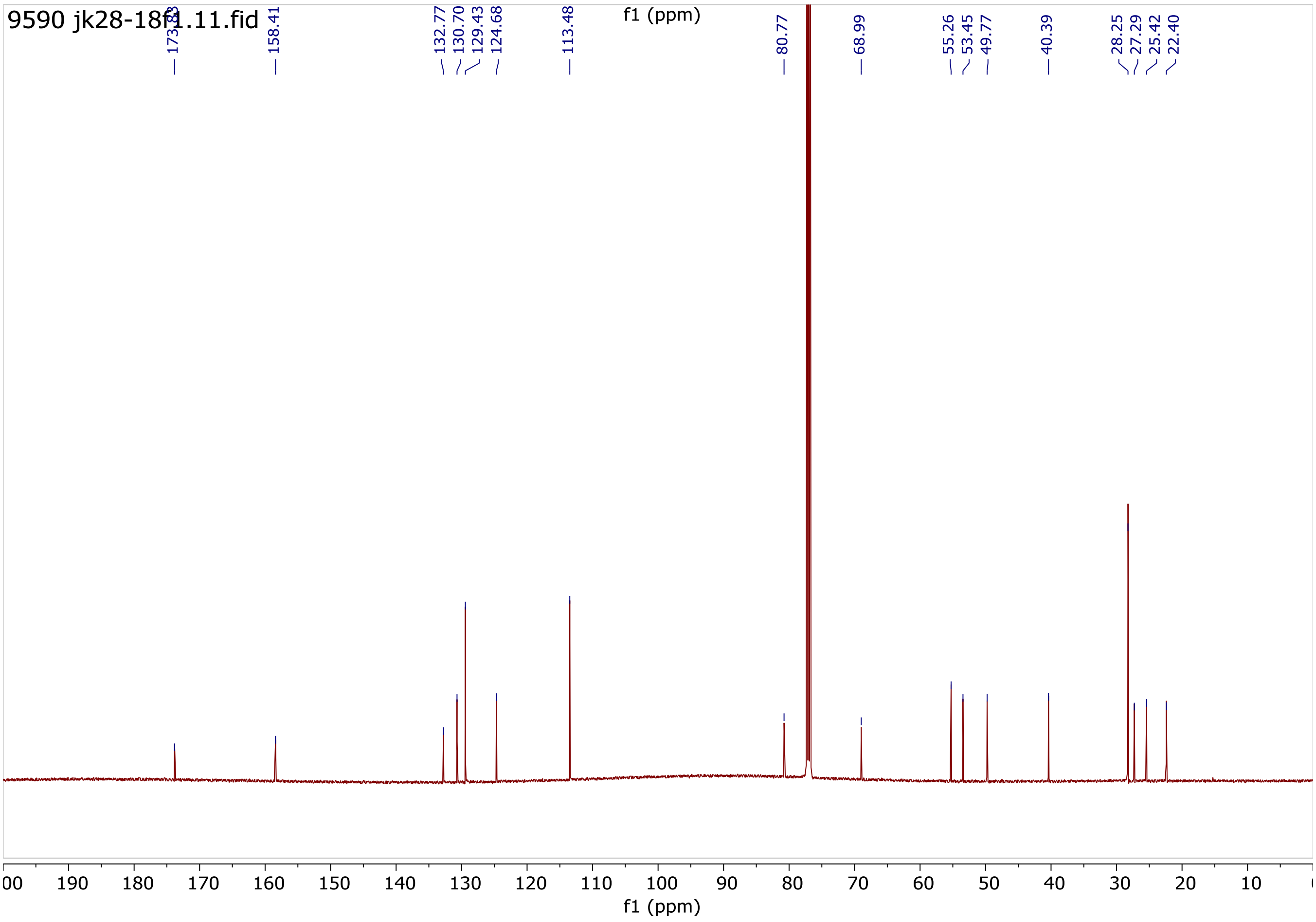

9590 jk28-18f1.11.fid
158.41
132.77
130.70
129.43
124.68
113.48
80.77
68.99
55.26
53.45
49.77
40.39
28.25
27.29
25.42
22.40
f1 (ppm)

CDCl$_3$

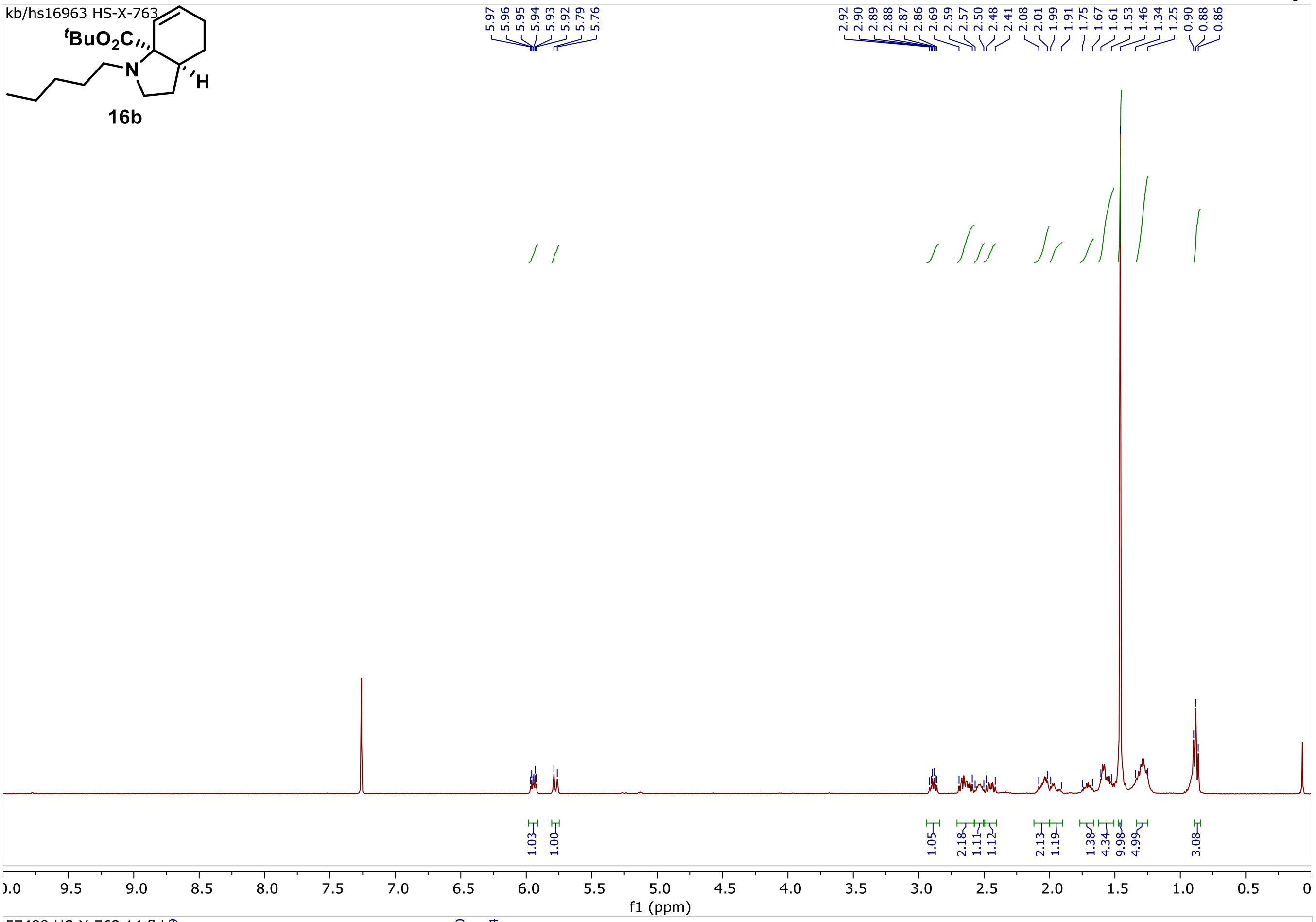

kb/hs16963 HS-X-763
^{t}BuO$_2$C
N
H
16b
5.97
5.96
5.95
5.94
5.93
5.92
5.79
5.76
2.92
2.90
2.89
2.88
2.87
2.86
2.69
2.59
2.57
2.50
2.48
2.41
2.08
2.01
1.99
1.91
1.75
1.67
1.61
1.53
1.46
1.34
1.25
0.90
0.88
0.86
1.03
1.00
1.05
2.18
1.11
1.12
2.13
1.19
1.38
4.34
9.98
4.99
3.08
f1 (ppm)

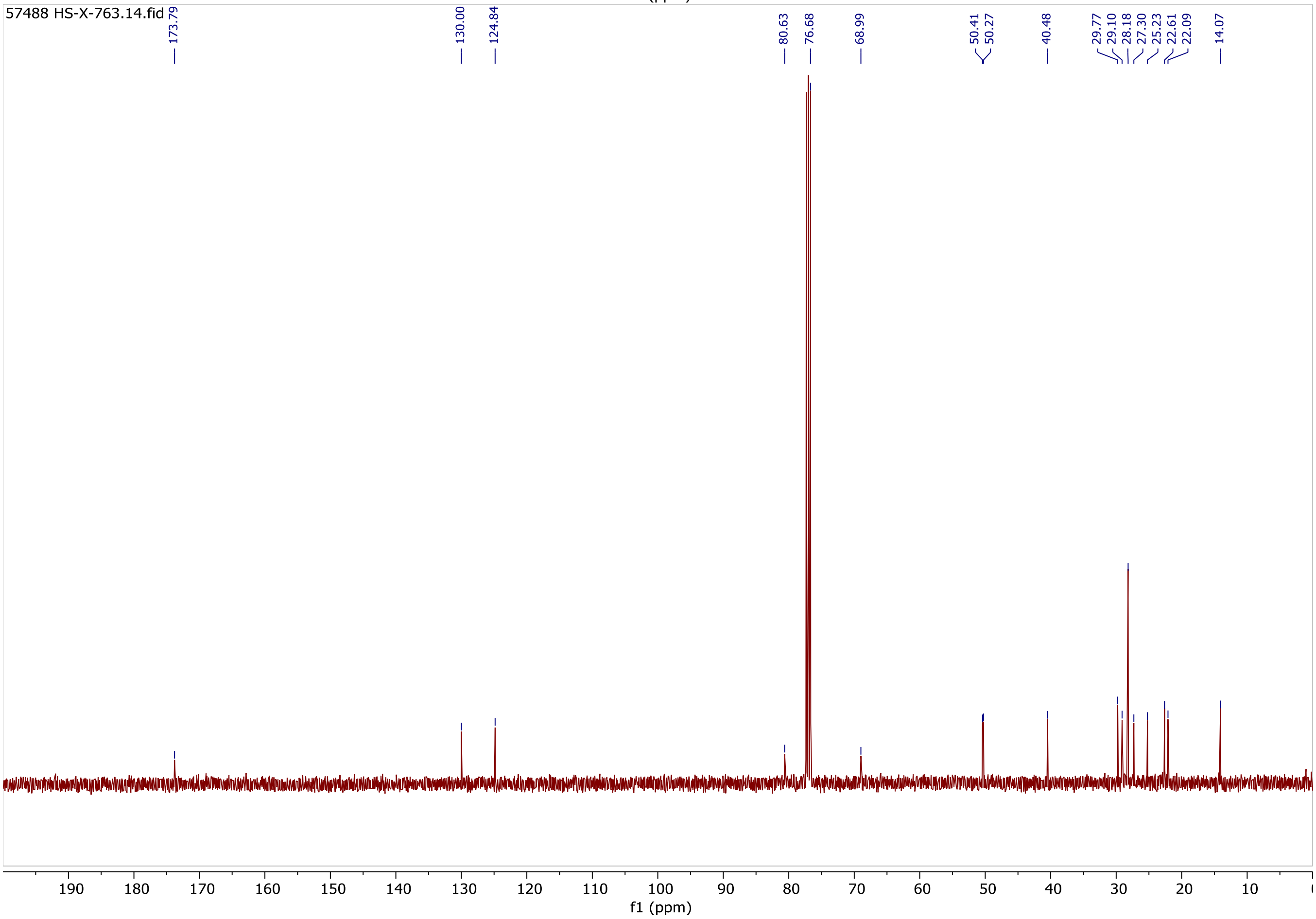

57488 HS-X-763.14.fid
173.79
130.00
124.84
80.63
76.68
68.99
50.41
50.27
40.48
29.77
29.10
28.18
27.30
25.23
22.61
22.09
14.07
f1 (ppm)

CDCl$_3$

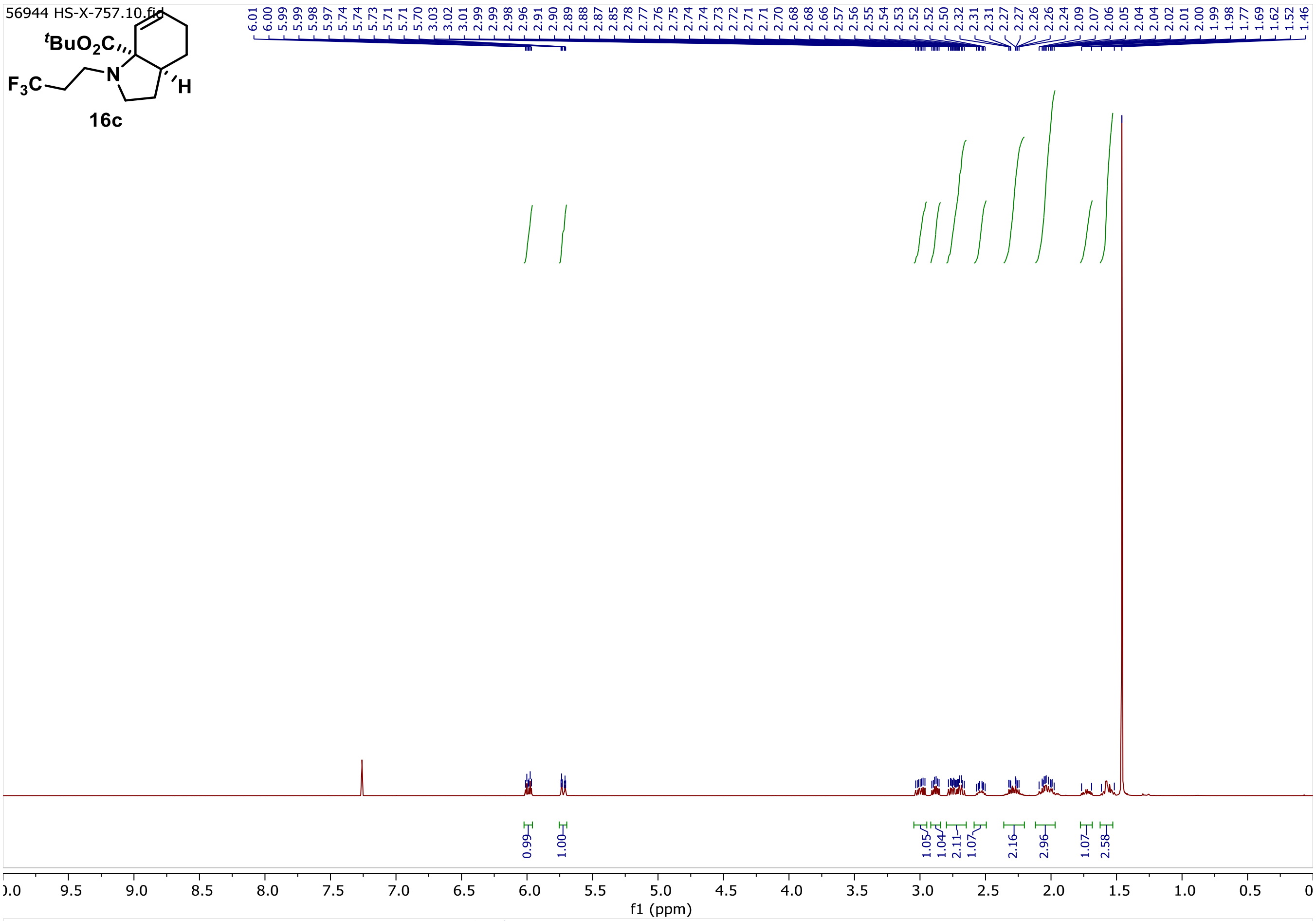
56944 HS-X-757.10.fid
^{t}BuO$_2$C
N
F$_3$C
H
16c
6.01 6.00 5.99 5.99 5.98 5.97 5.74 5.74 5.73 5.71 5.71 5.70 3.03 3.02 3.01 2.99 2.99 2.98 2.96 2.91 2.90 2.89 2.88 2.87 2.85 2.78 2.77 2.76 2.75 2.74 2.74 2.73 2.72 2.71 2.71 2.70 2.68 2.68 2.66 2.57 2.56 2.55 2.54 2.53 2.52 2.52 2.50 2.32 2.31 2.27 2.27 2.26 2.26 2.24 2.09 2.07 2.06 2.05 2.04 2.04 2.02 2.01 2.00 1.99 1.98 1.77 1.69 1.62 1.52 1.46
0.99 1.00 1.05 1.04 2.11 1.07 2.16 2.96 1.07 2.58
f1 (ppm)

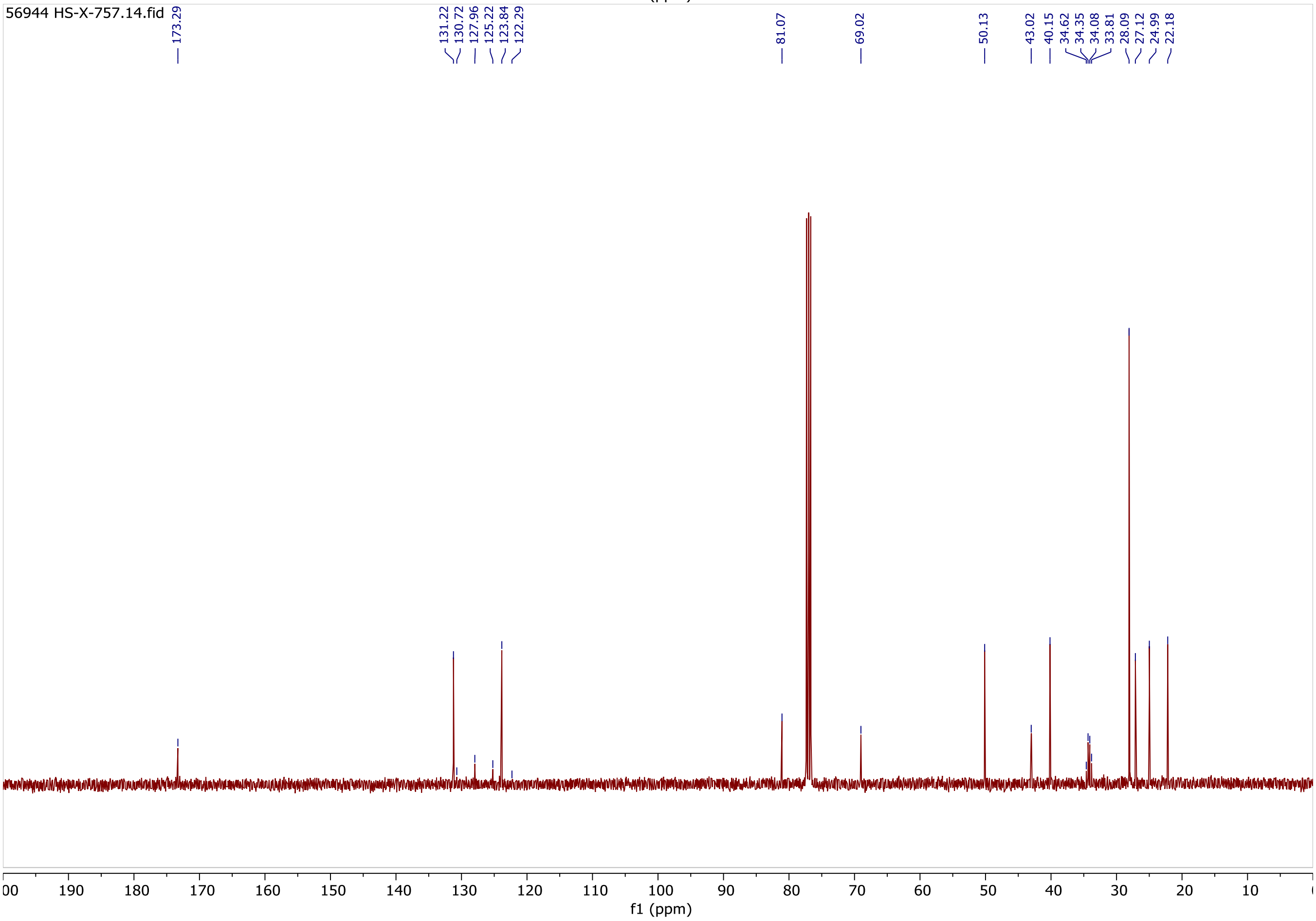
56944 HS-X-757.14.fid
173.29
131.22 130.72 127.96 125.22 123.84 122.29
81.07
69.02
50.13
43.02 40.15 34.62 34.35 34.08 33.81 28.09 27.12 24.99 22.18
f1 (ppm)

CDCl$_3$

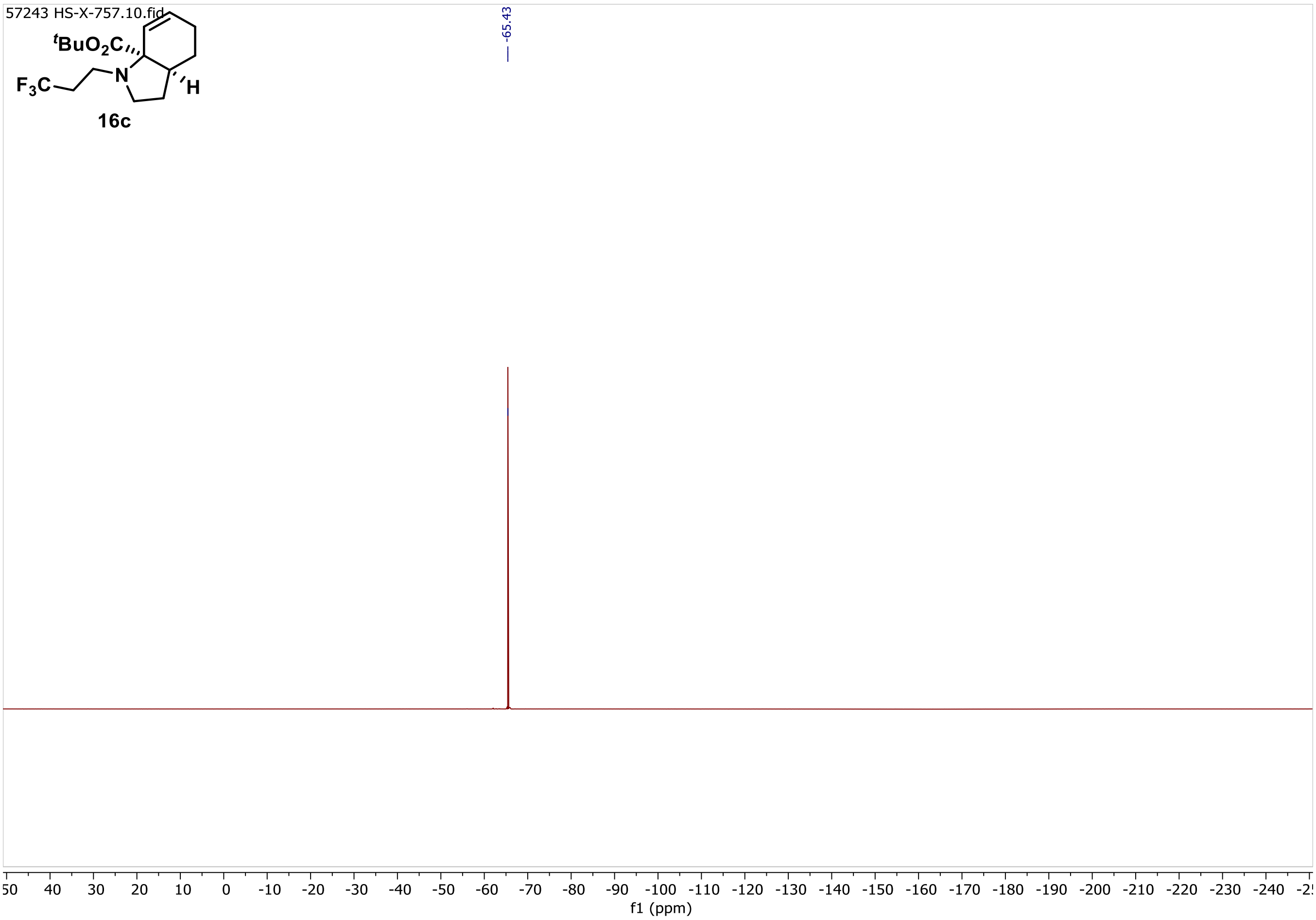

57243 HS-X-757.10.fid
-65.43
50 40 30 20 10 0 -10 -20 -30 -40 -50 -60 -70 -80 -90 -100 -110 -120 -130 -140 -150 -160 -170 -180 -190 -200 -210 -220 -230 -240
f1 (ppm)

16c

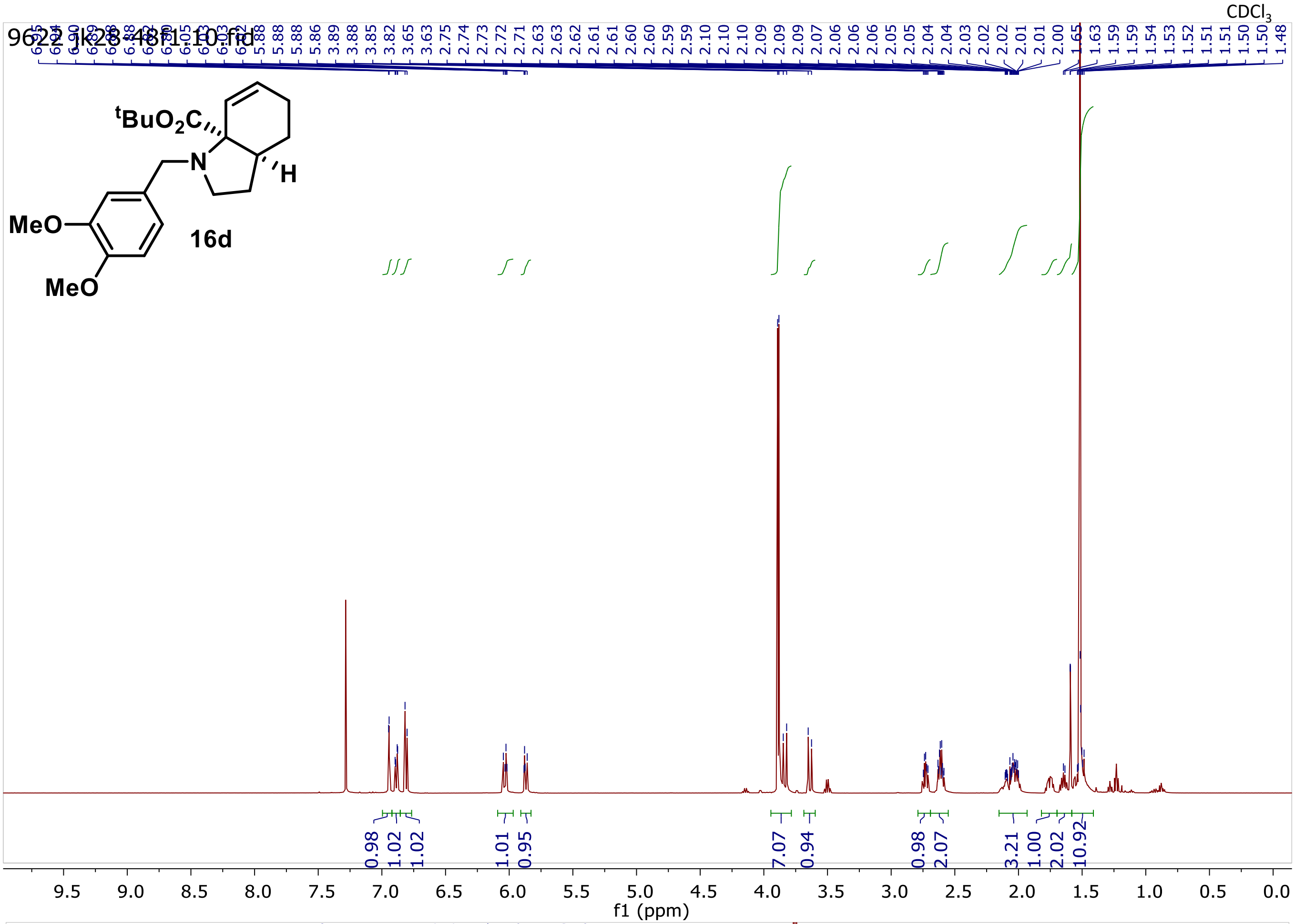

9622 jk28-48f1.10.fid
CDCl$_3$
5.88 5.88 5.86 3.89 3.88 3.85 3.82 3.65 3.63 2.75 2.74 2.73 2.72 2.71 2.63 2.62 2.61 2.61 2.60 2.60 2.59 2.59 2.10 2.10 2.10 2.09 2.09 2.09 2.07 2.06 2.06 2.05 2.05 2.04 2.04 2.03 2.02 2.02 2.01 2.01 2.00 1.65 1.63 1.59 1.59 1.54 1.53 1.52 1.51 1.51 1.50 1.50 1.48
^{t}BuO$_2$C
N
H
MeO
MeO
16d
0.98 1.02 1.02 1.01 0.95 7.07 0.94 0.98 2.07 3.21 1.00 2.02 10.92
9.5 9.0 8.5 8.0 7.5 7.0 6.5 6.0 5.5 5.0 4.5 4.0 3.5 3.0 2.5 2.0 1.5 1.0 0.5 0.0
f1 (ppm)

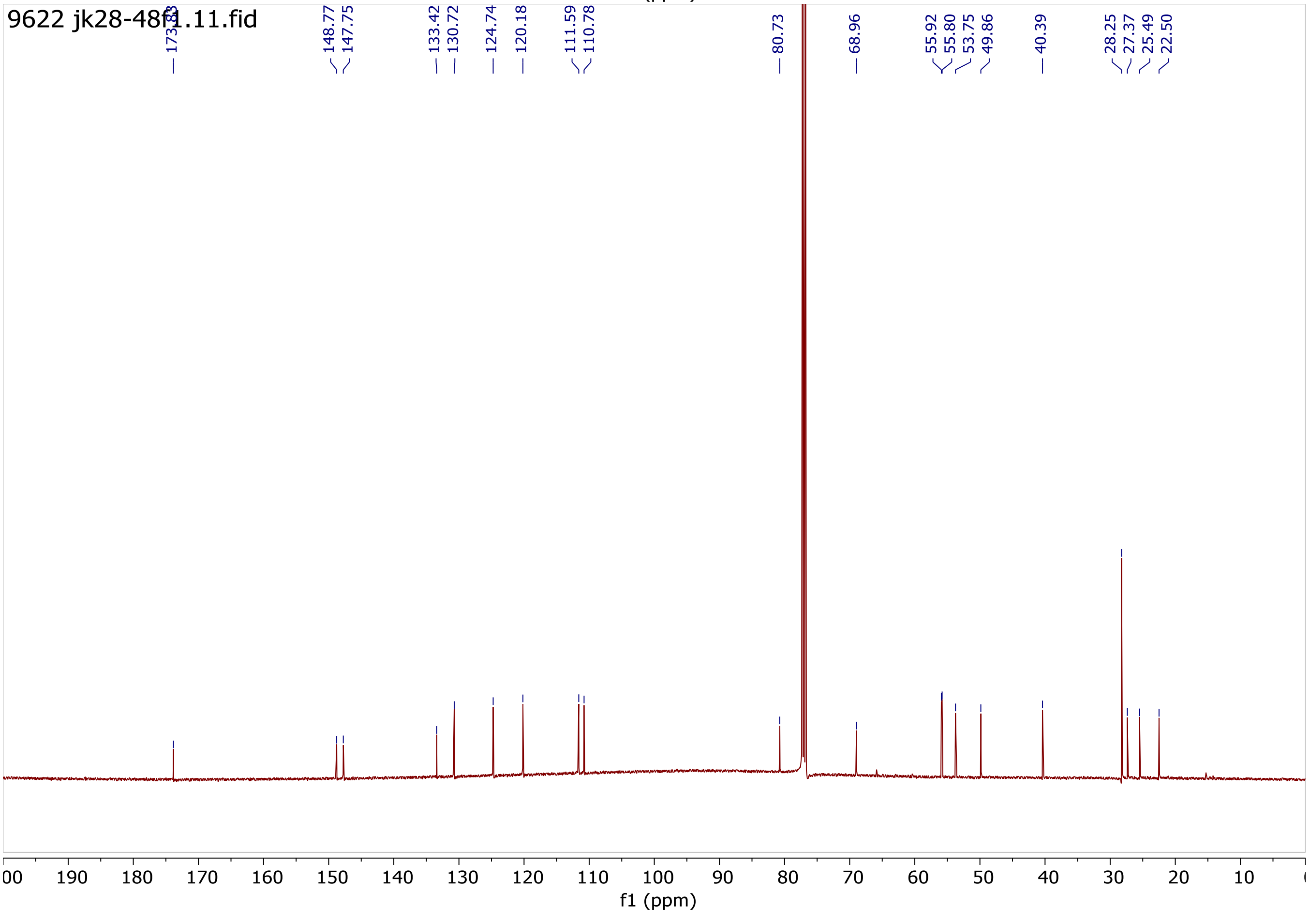

9622 jk28-48f1.11.fid
148.77 147.75 133.42 130.72 124.74 120.18 111.59 110.78 80.73 68.96 55.92 55.80 53.75 49.86 40.39 28.25 27.37 25.49 22.50
190 180 170 160 150 140 130 120 110 100 90 80 70 60 50 40 30 20 10
f1 (ppm)

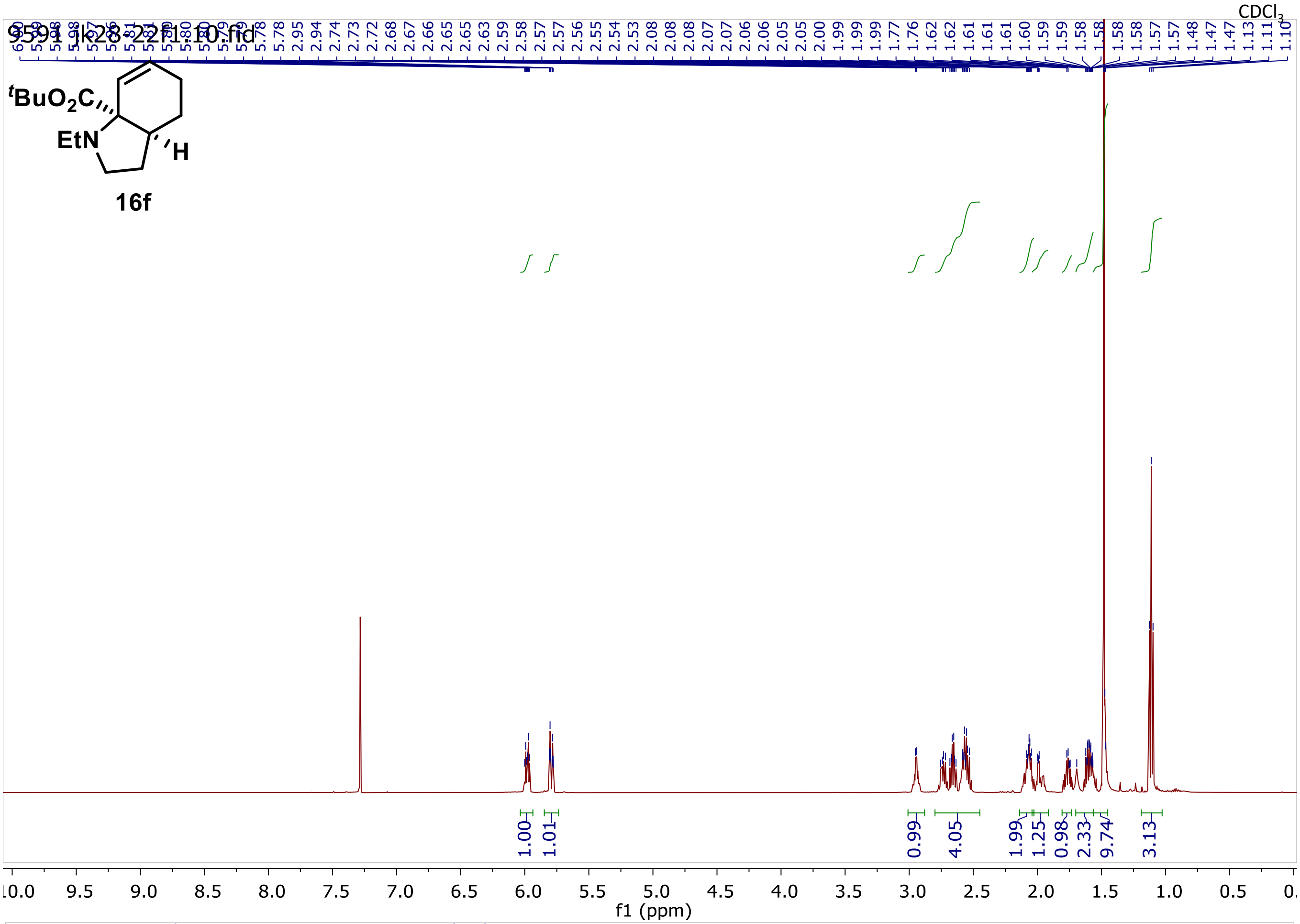
9591 jk28-22f1.10.fid
CDCl3
5.78
5.78
2.95
2.94
2.74
2.73
2.72
2.68
2.67
2.66
2.65
2.65
2.63
2.59
2.58
2.57
2.57
2.56
2.55
2.54
2.53
2.08
2.08
2.08
2.07
2.07
2.06
2.06
2.05
2.05
2.00
1.99
1.99
1.99
1.77
1.76
1.62
1.61
1.61
1.61
1.60
1.59
1.59
1.58
1.58
1.58
1.58
1.57
1.57
1.48
1.47
1.47
1.13
1.11
1.10
tBuO2C
EtN
H
16f
1.00
1.01
0.99
4.05
1.99
1.25
0.98
2.33
9.74
3.13
f1 (ppm)

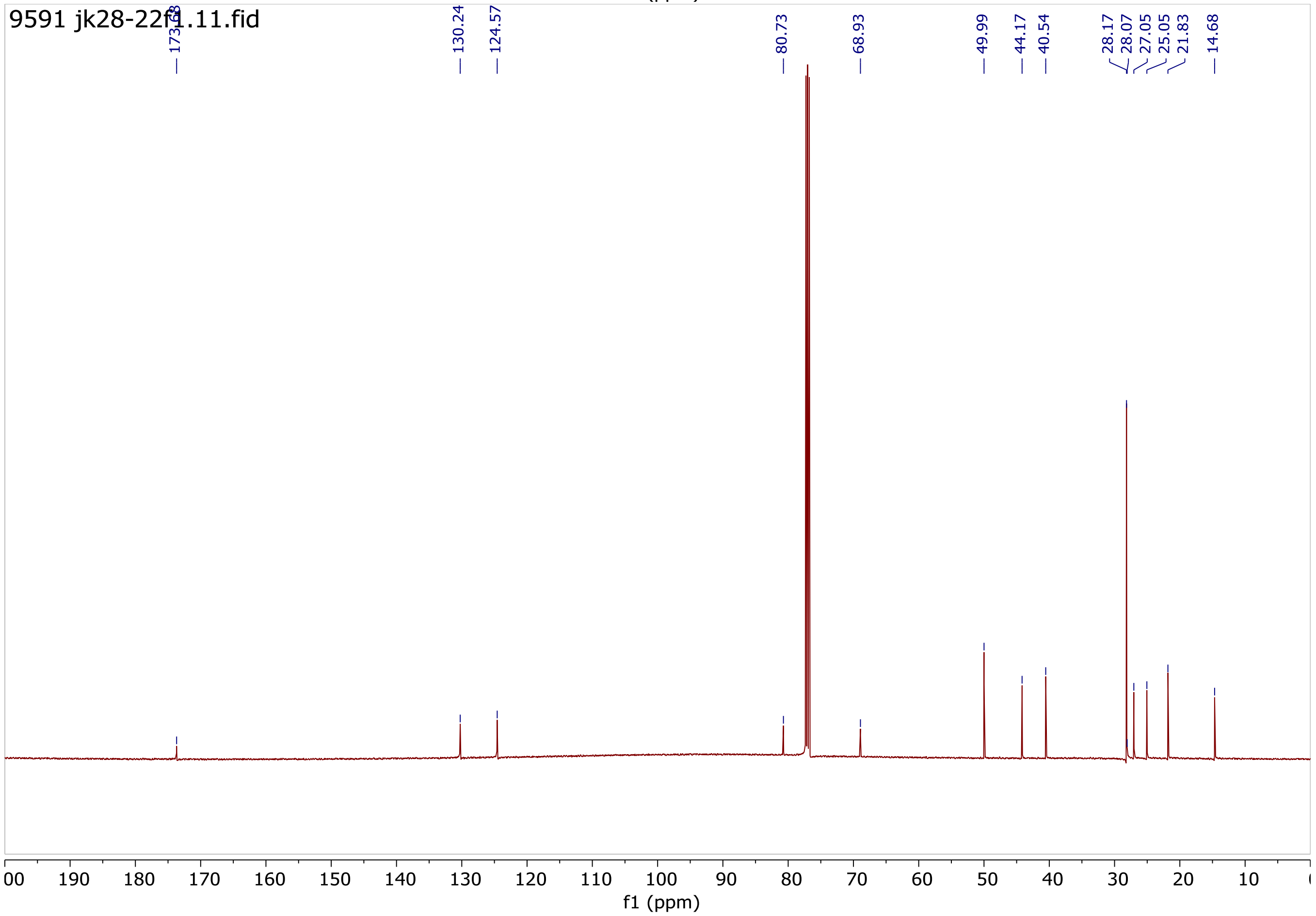
9591 jk28-22f1.11.fid
173.48
130.24
124.57
80.73
68.93
49.99
44.17
40.54
28.17
28.07
27.05
25.05
21.83
14.68
f1 (ppm)

CDCl$_3$

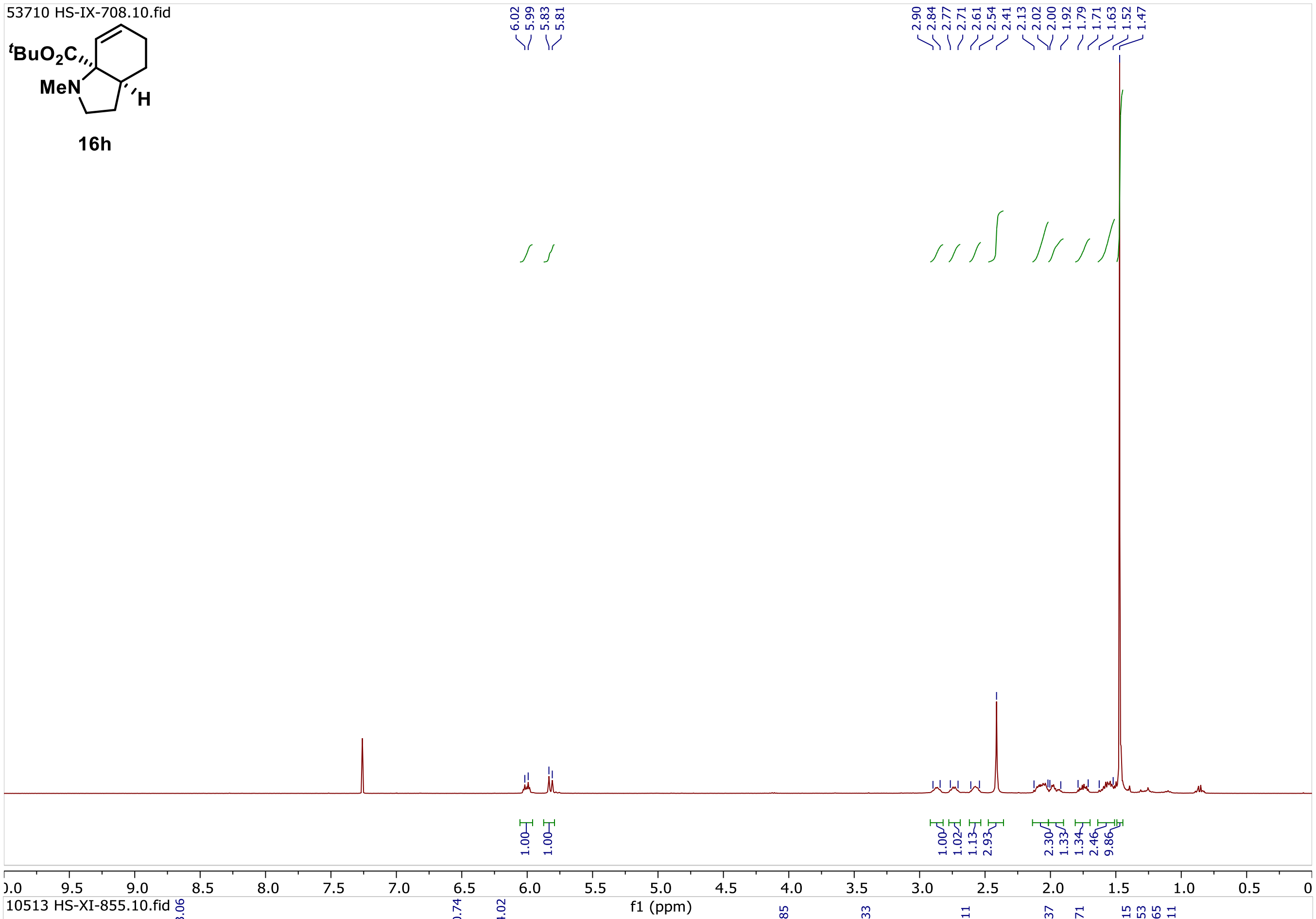

53710 HS-IX-708.10.fid
^{t}BuO$_2$C
MeN
H
16h
6.02 5.99 5.83 5.81
2.90 2.84 2.77 2.71 2.61 2.54 2.41 2.13 2.02 2.00 1.92 1.79 1.71 1.63 1.52 1.47
1.00 1.00
1.00 1.02 1.13 2.93
2.30 1.33 1.34 2.46 9.86
10.0 9.5 9.0 8.5 8.0 7.5 7.0 6.5 6.0 5.5 5.0 4.5 4.0 3.5 3.0 2.5 2.0 1.5 1.0 0.5 0
f1 (ppm)

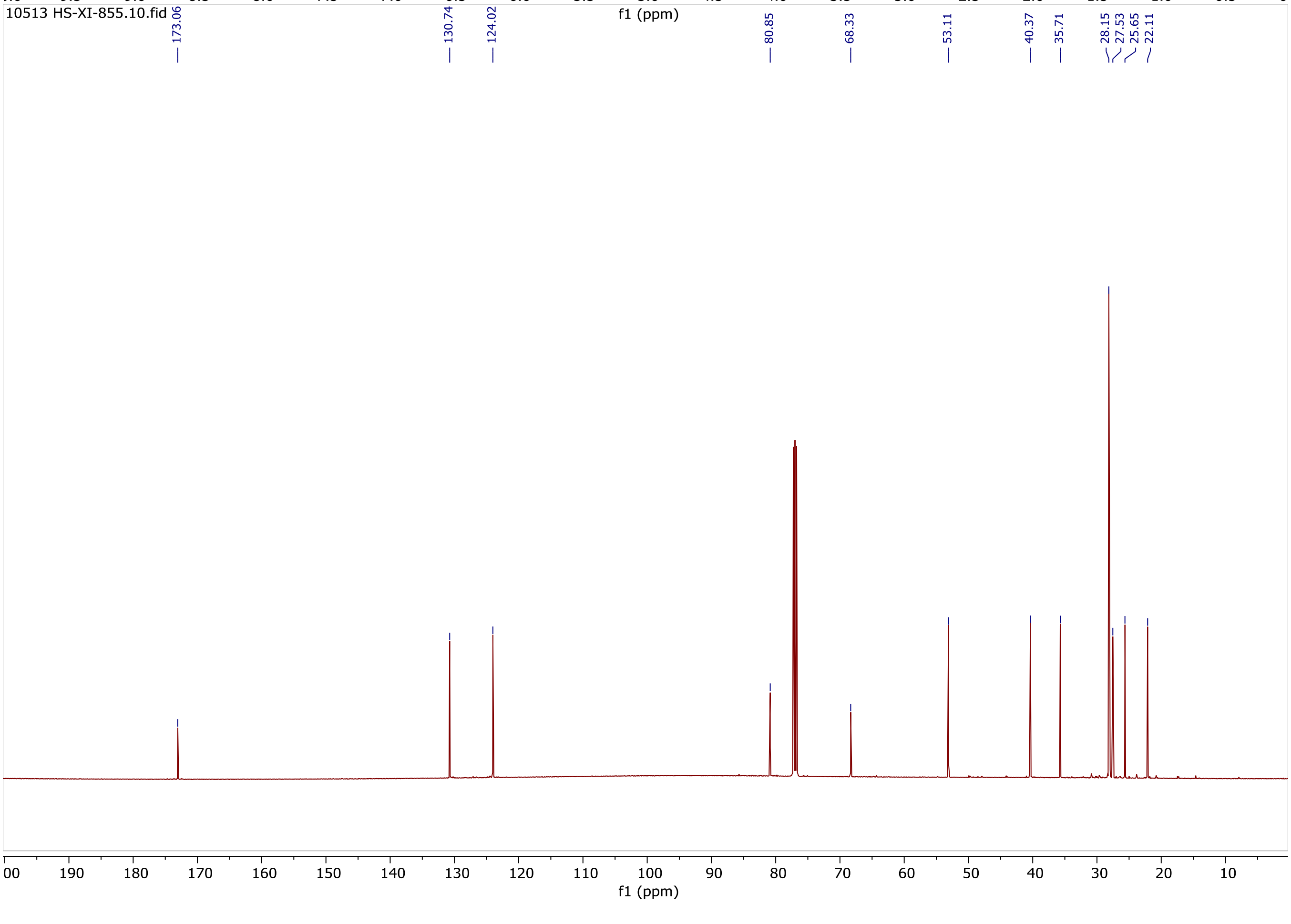

10513 HS-XI-855.10.fid
173.06
130.74
124.02
80.85
68.33
53.11
40.37
35.71
28.15 27.53 25.65 22.11
200 190 180 170 160 150 140 130 120 110 100 90 80 70 60 50 40 30 20 10
f1 (ppm)

CDCl$_3$

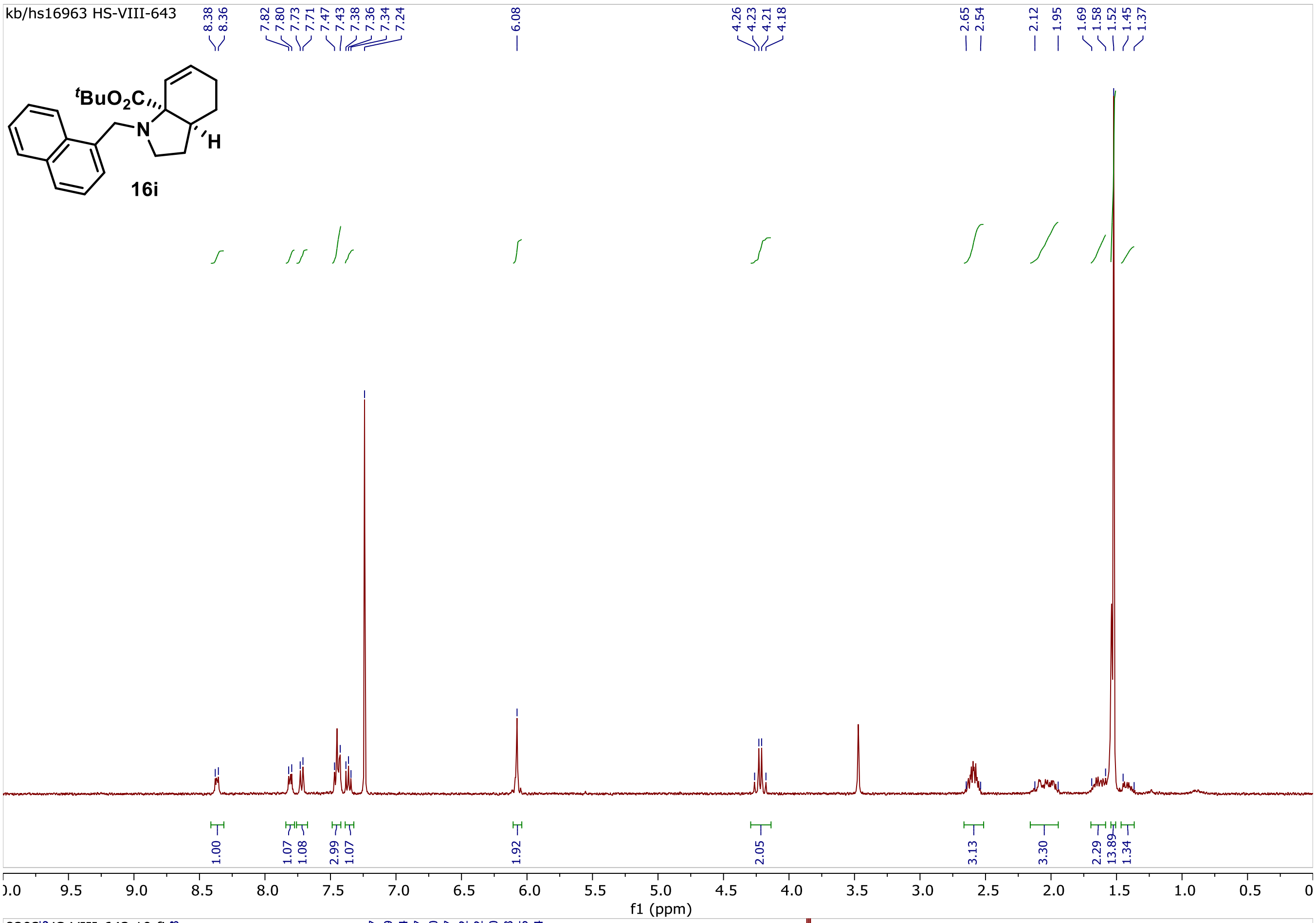

^{t}BuO$_2$C
N
H
16i
kb/hs16963 HS-VIII-643
8.38 8.36 7.82 7.80 7.73 7.71 7.47 7.43 7.38 7.36 7.34 7.24
6.08
4.26 4.23 4.21 4.18
2.65 2.54 2.12 1.95 1.69 1.58 1.52 1.45 1.37
1.00 1.07 1.08 2.99 1.07 1.92 2.05 3.13 3.30 2.29 13.89 1.34
f1 (ppm)

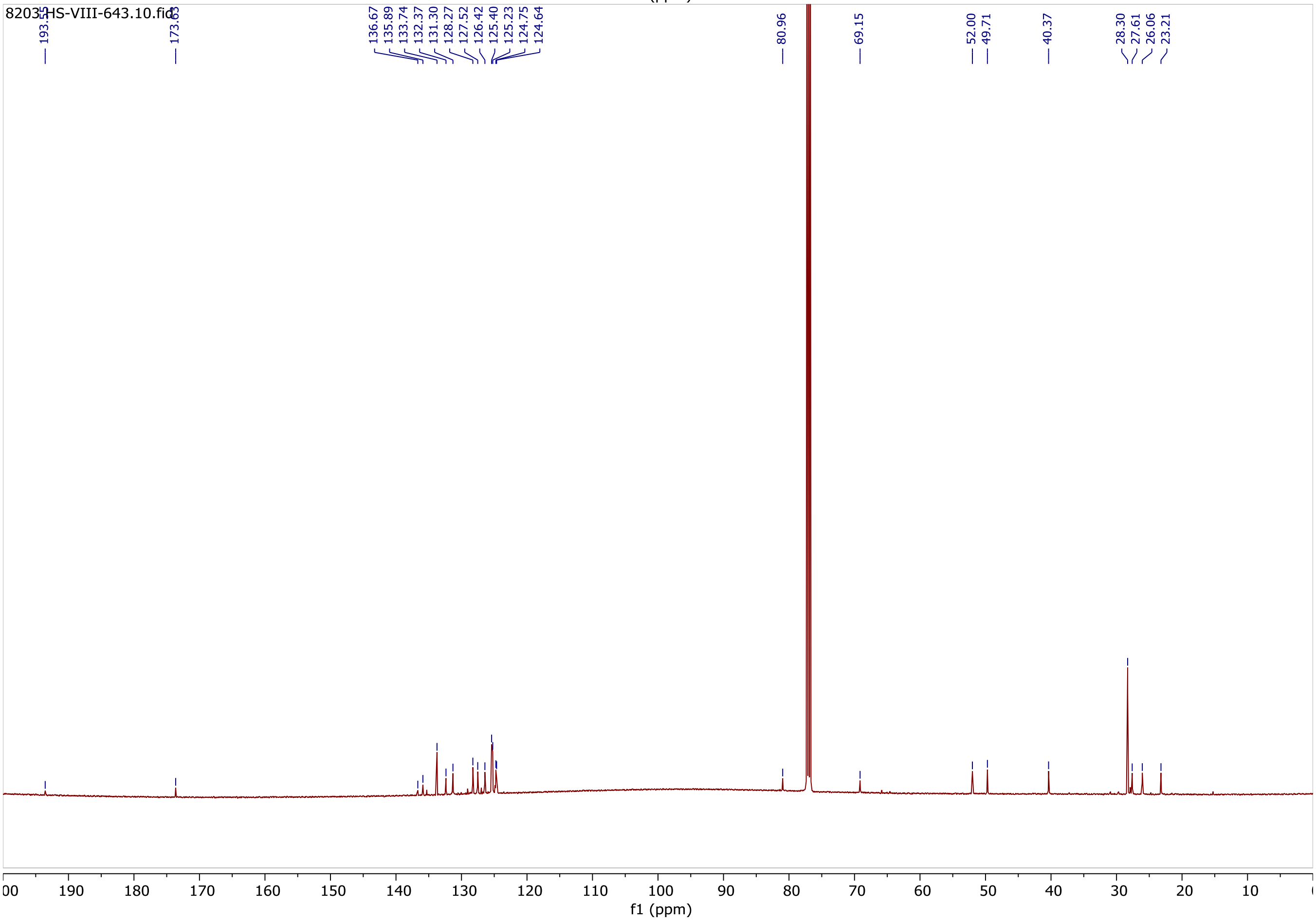

8203 HS-VIII-643.10.fid
136.67 135.89 133.74 132.37 131.30 128.27 127.52 126.42 125.40 125.23 124.75 124.64
80.96
69.15
52.00 49.71
40.37
28.30 27.61 26.06 23.21
f1 (ppm)

CDCl$_3$

kb/hs16963 HS-IX-675

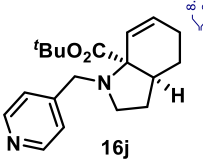

16j

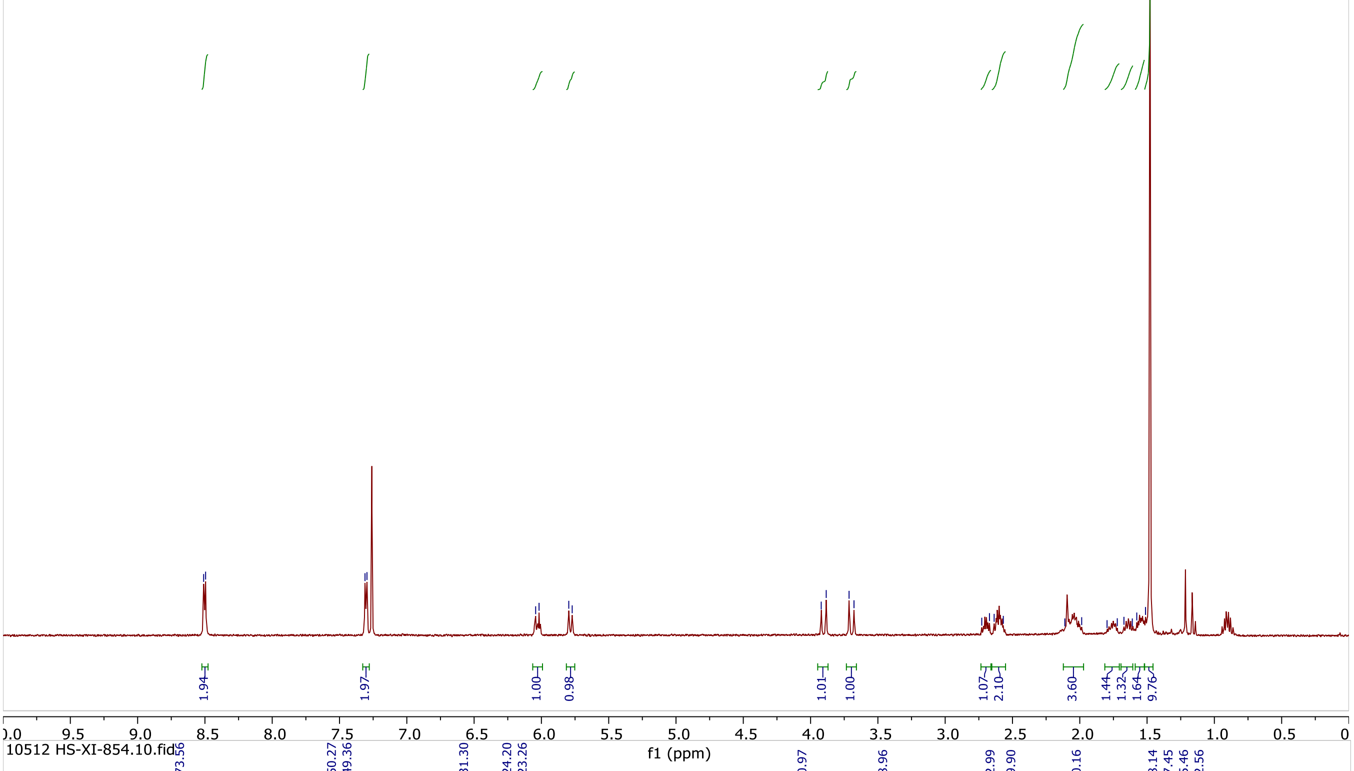

10512 HS-XI-854.10.fid

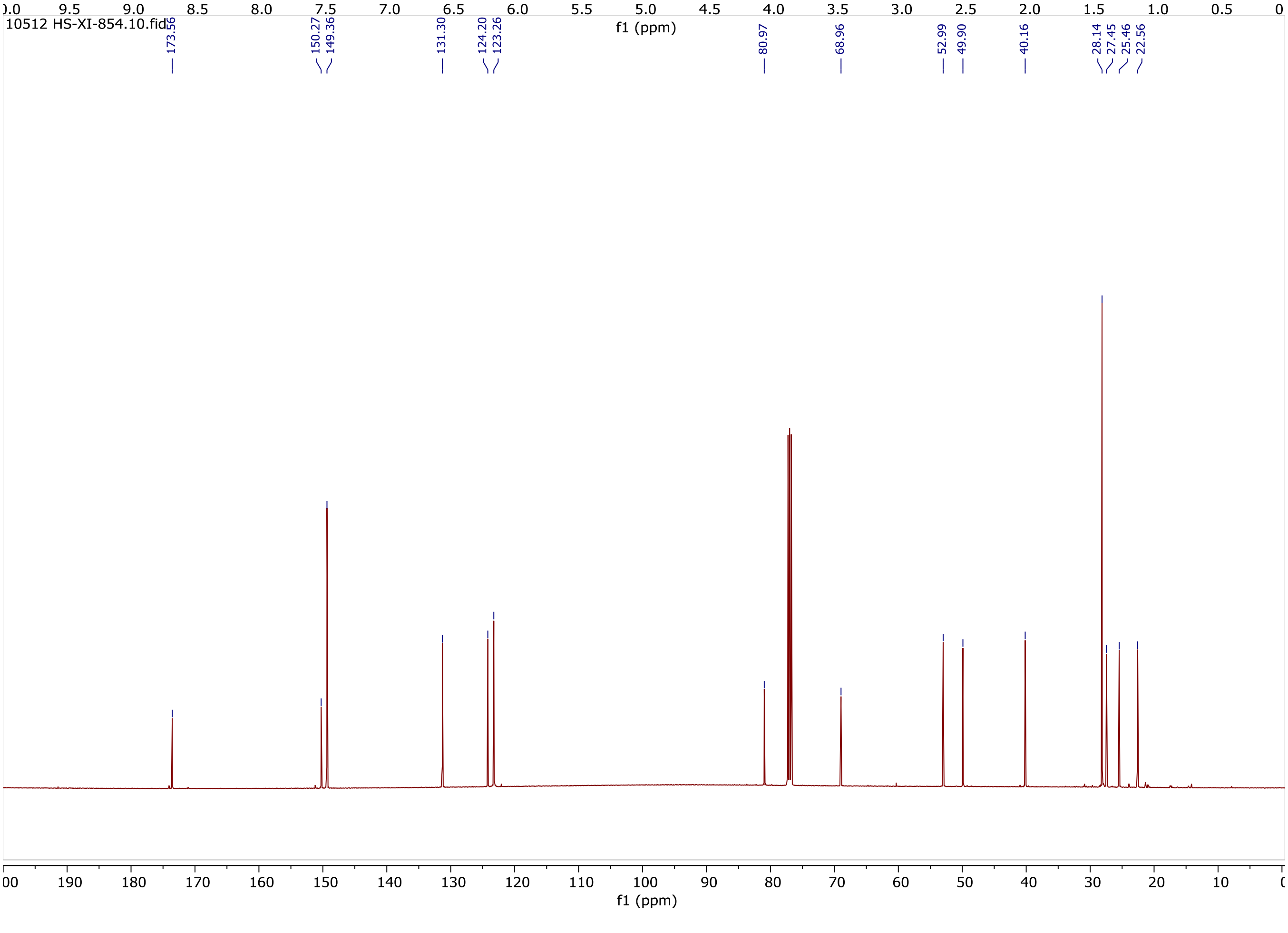

CDCl$_3$

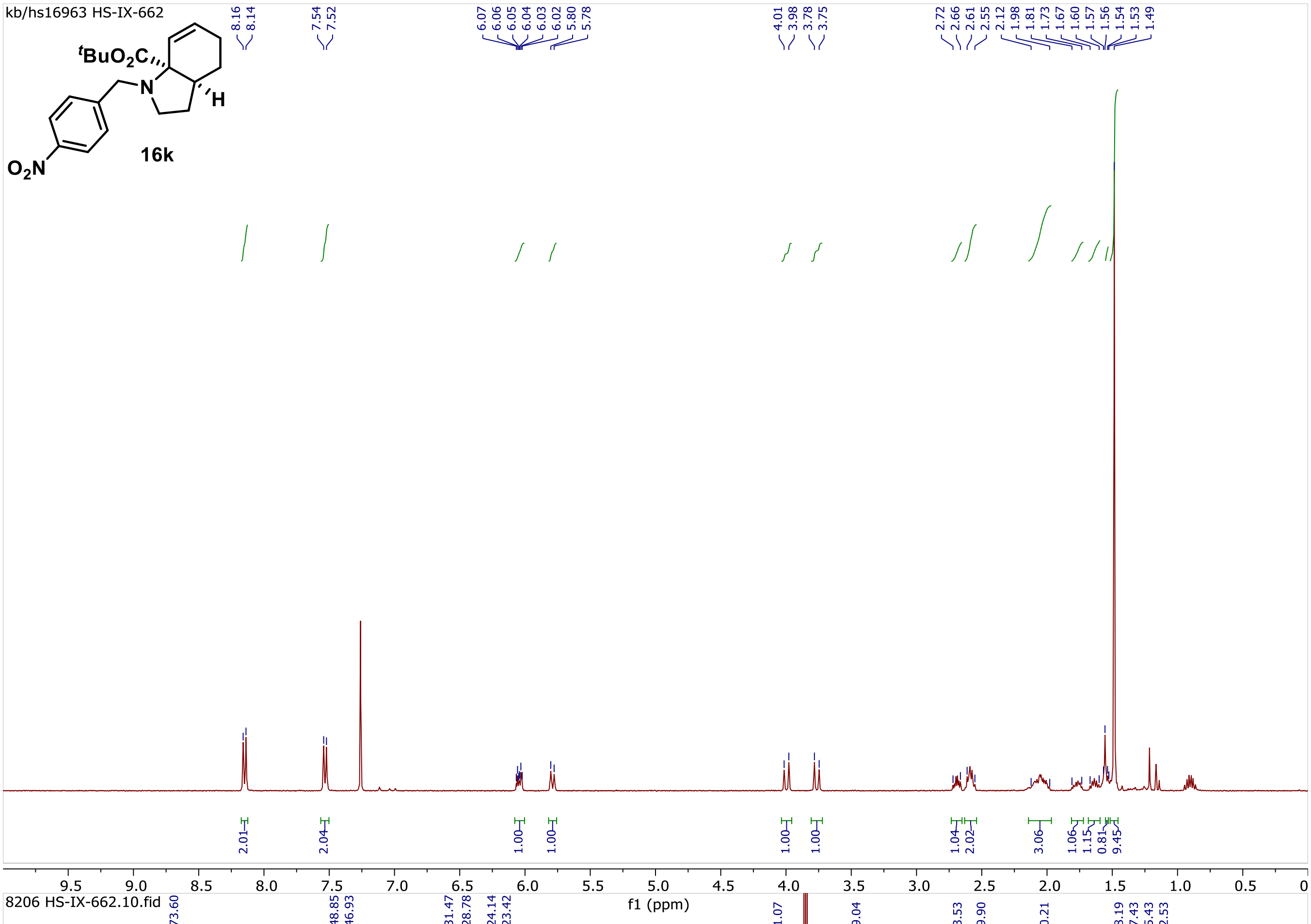
kb/hs16963 HS-IX-662
tBuO2C
N
H
O2N
16k
8.16
8.14
7.54
7.52
6.07
6.06
6.05
6.04
6.03
6.02
5.80
5.78
4.01
3.98
3.78
3.75
2.72
2.66
2.61
2.55
2.12
1.98
1.81
1.73
1.67
1.60
1.57
1.56
1.54
1.53
1.49
2.01
2.04
1.00
1.00
1.00
1.00
1.04
2.02
3.06
1.06
1.15
0.81
9.45
f1 (ppm)

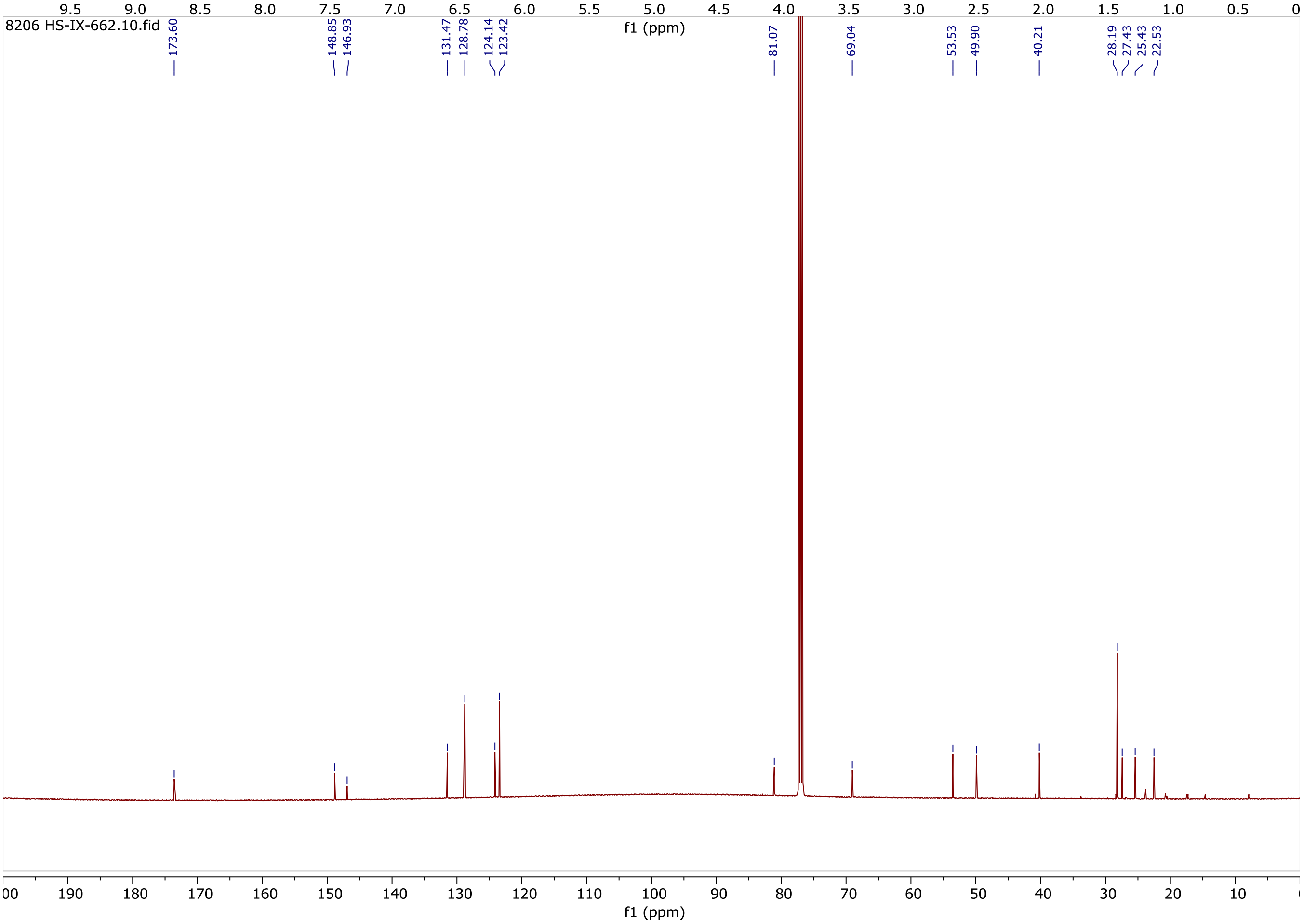
8206 HS-IX-662.10.fid
173.60
148.85
146.93
131.47
128.78
124.14
123.42
81.07
69.04
53.53
49.90
40.21
28.19
27.43
25.43
22.53
f1 (ppm)

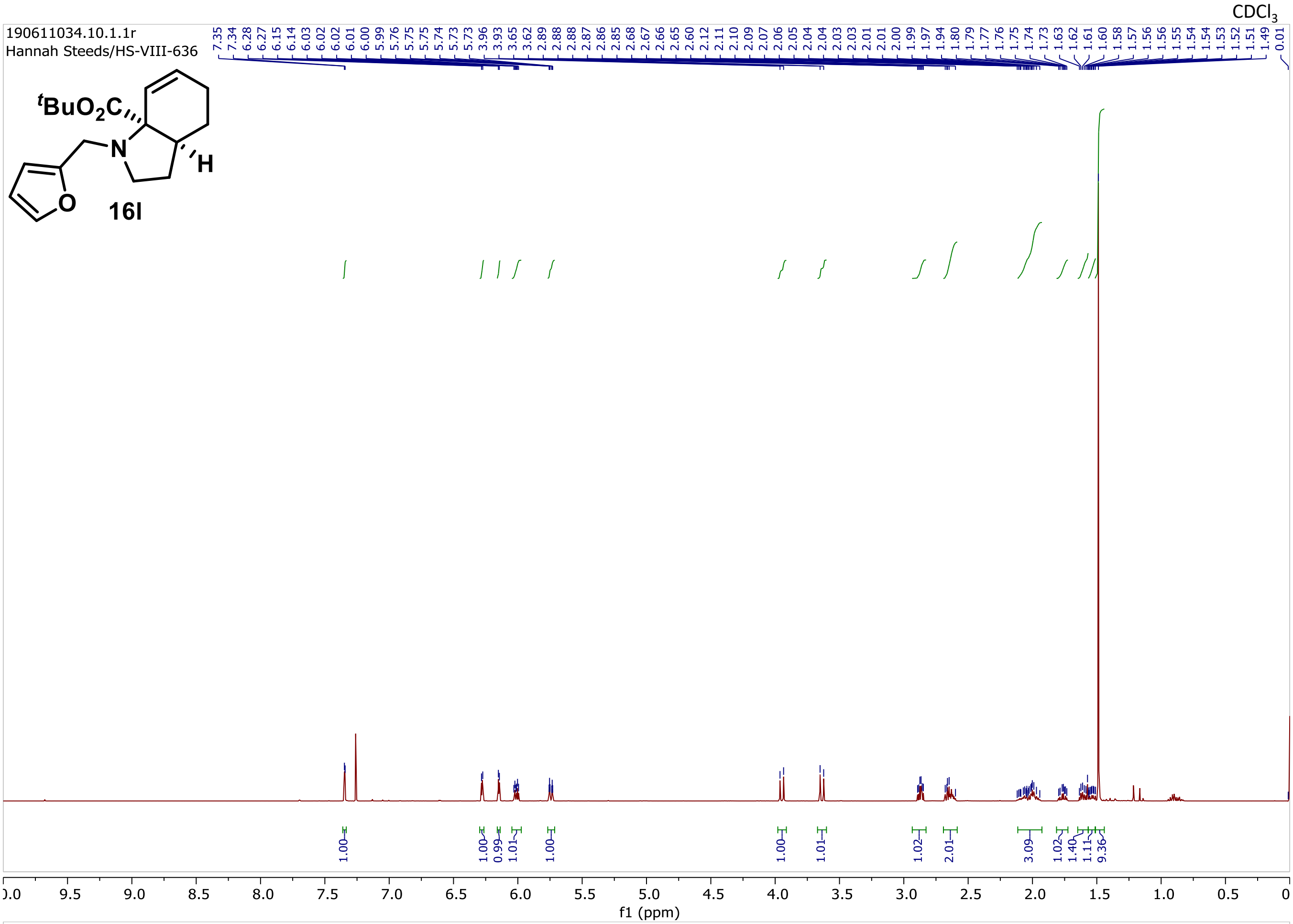
CDCl$_3$
190611034.10.1.1r
Hannah Steeds/HS-VIII-636
7.35 7.34 6.28 6.27 6.15 6.14 6.03 6.02 6.02 6.01 6.00 5.99 5.76 5.75 5.75 5.74 5.73 5.73 3.96 3.93 3.65 3.62 2.89 2.88 2.87 2.86 2.85 2.68 2.67 2.66 2.65 2.60 2.12 2.11 2.10 2.09 2.07 2.06 2.05 2.04 2.04 2.03 2.03 2.01 2.01 2.00 1.99 1.97 1.94 1.80 1.79 1.77 1.76 1.75 1.74 1.73 1.63 1.62 1.61 1.60 1.58 1.57 1.56 1.55 1.54 1.54 1.53 1.52 1.51 1.49 0.01
^{t}BuO$_2$C
N
H
O
16l
1.00 1.00 0.99 1.01 1.00 1.00 1.01 1.02 2.01 3.09 1.02 1.40 1.11 9.36
f1 (ppm)

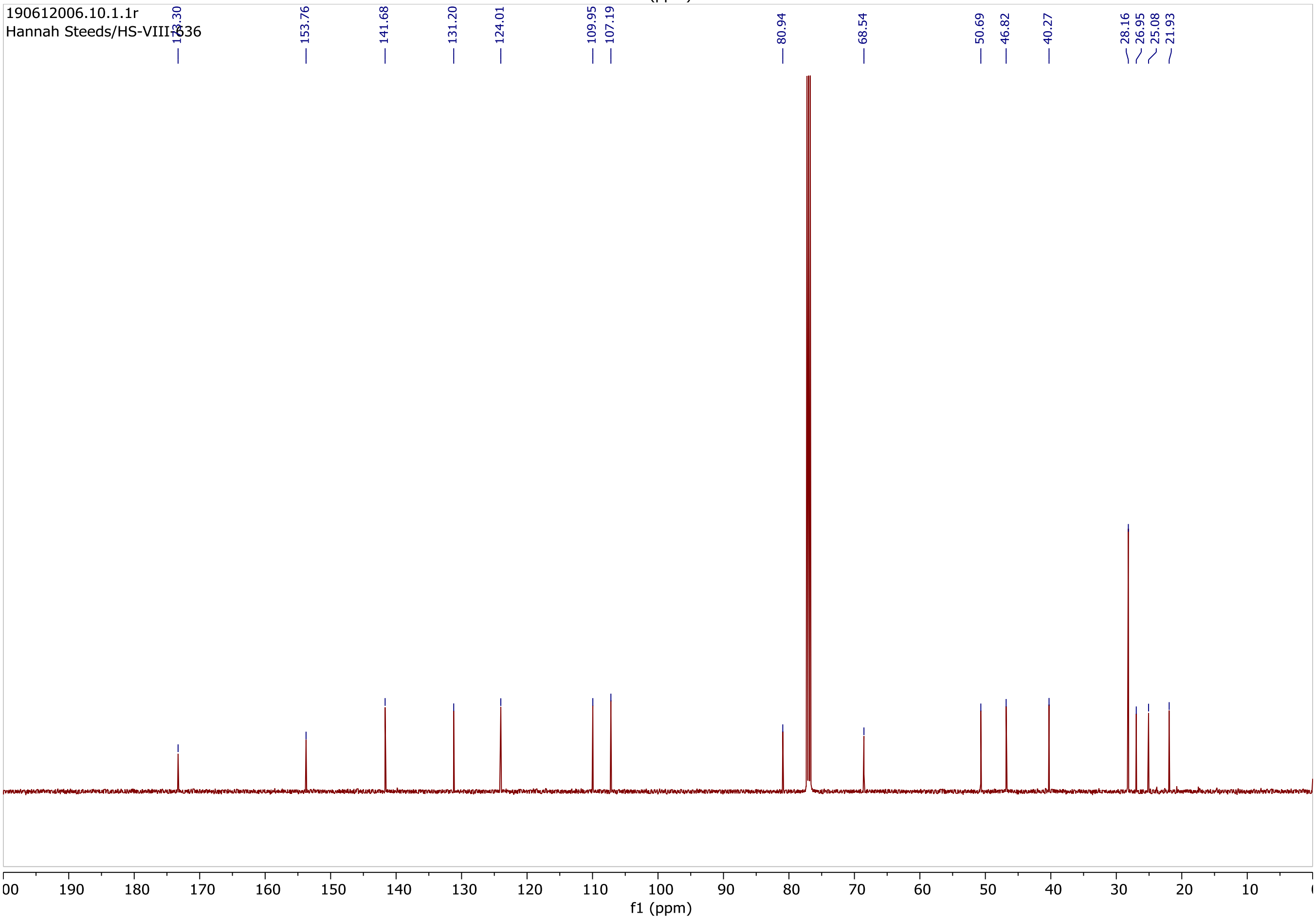
190612006.10.1.1r
Hannah Steeds/HS-VIII-636
173.30 153.76 141.68 131.20 124.01 109.95 107.19 80.94 68.54 50.69 46.82 40.27 28.16 26.95 25.08 21.93
f1 (ppm)

CDCl$_3$

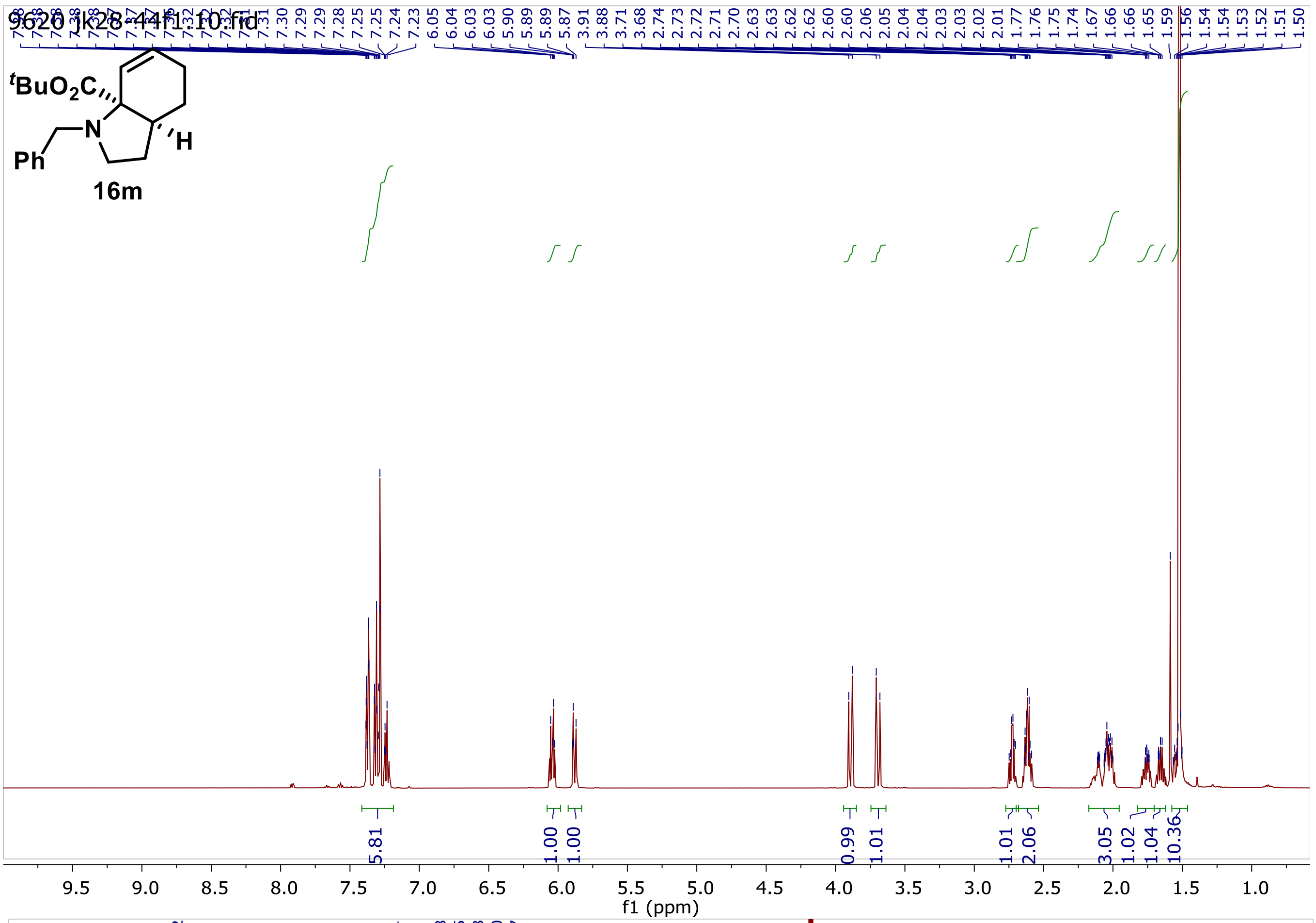
9620 jk28-44f1.10.fid
^{t}BuO$_2$C
N
Ph
H
16m
f1 (ppm)

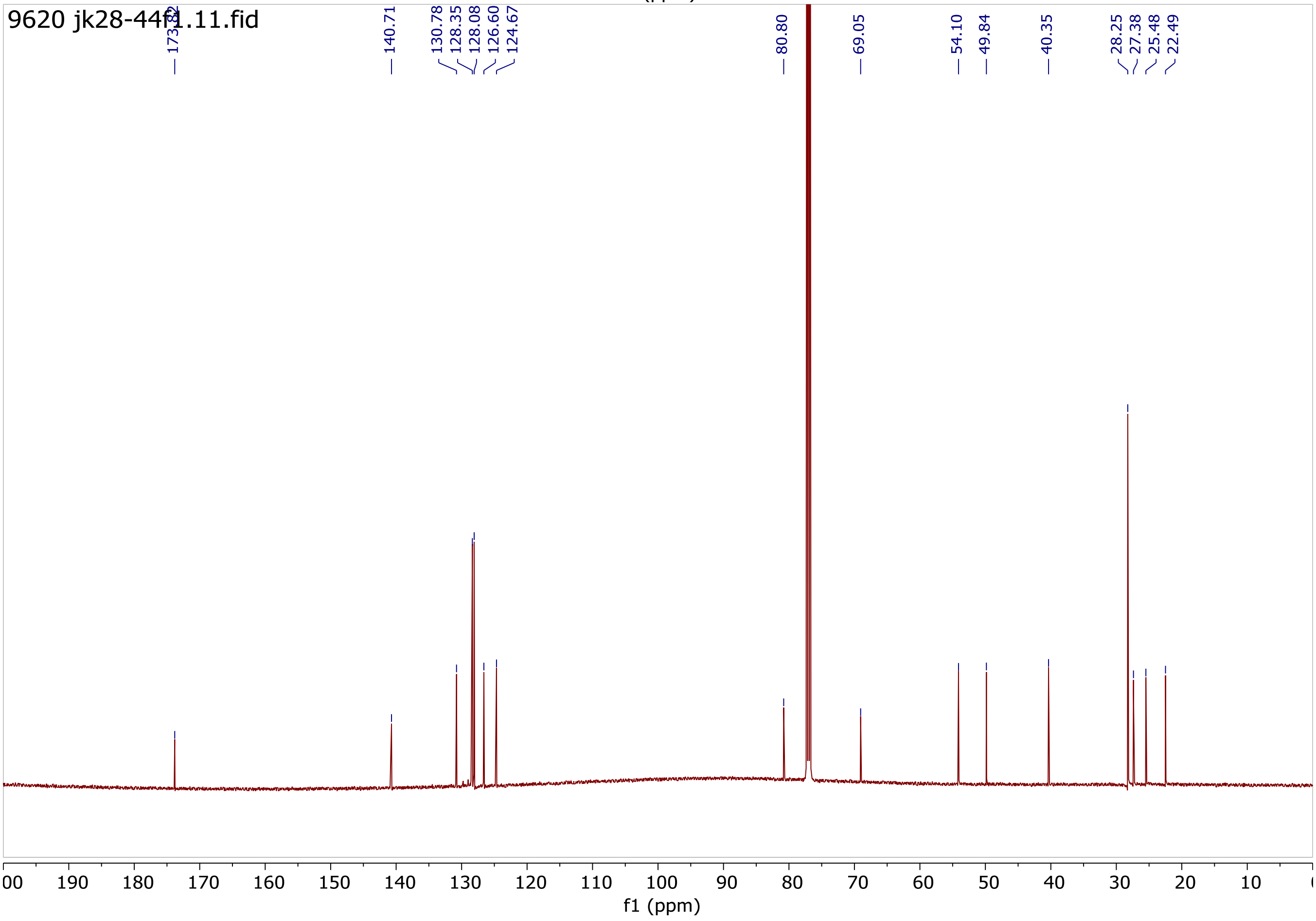
9620 jk28-44f1.11.fid
173.92
140.71
130.78
128.35
128.08
126.60
124.67
80.80
69.05
54.10
49.84
40.35
28.25
27.38
25.48
22.49
f1 (ppm)

$CDCl_3$

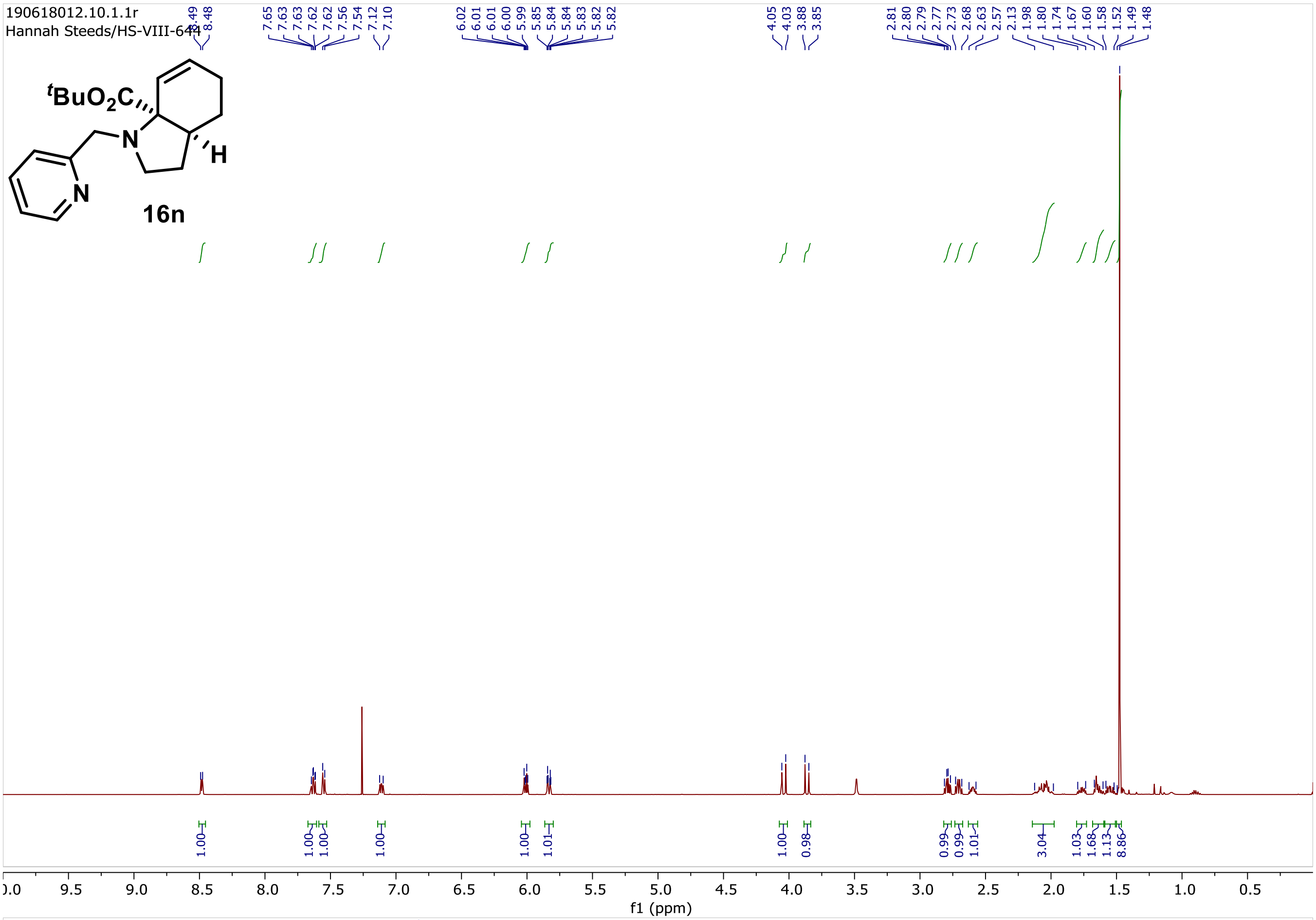

190618012.10.1.1r
Hannah Steeds/HS-VIII-644
8.49 8.48 7.65 7.63 7.63 7.62 7.62 7.56 7.54 7.12 7.10 6.02 6.01 6.01 6.00 5.99 5.85 5.84 5.84 5.83 5.82 5.82 4.05 4.03 3.88 3.85 2.81 2.80 2.79 2.77 2.73 2.68 2.63 2.57 2.13 1.98 1.80 1.74 1.67 1.60 1.58 1.52 1.49 1.48
tBuO2C
N
H
N
16n
1.00 1.00 1.00 1.00 1.00 1.01 1.00 0.98 0.99 0.99 1.01 3.04 1.03 1.68 1.13 8.86
f1 (ppm)

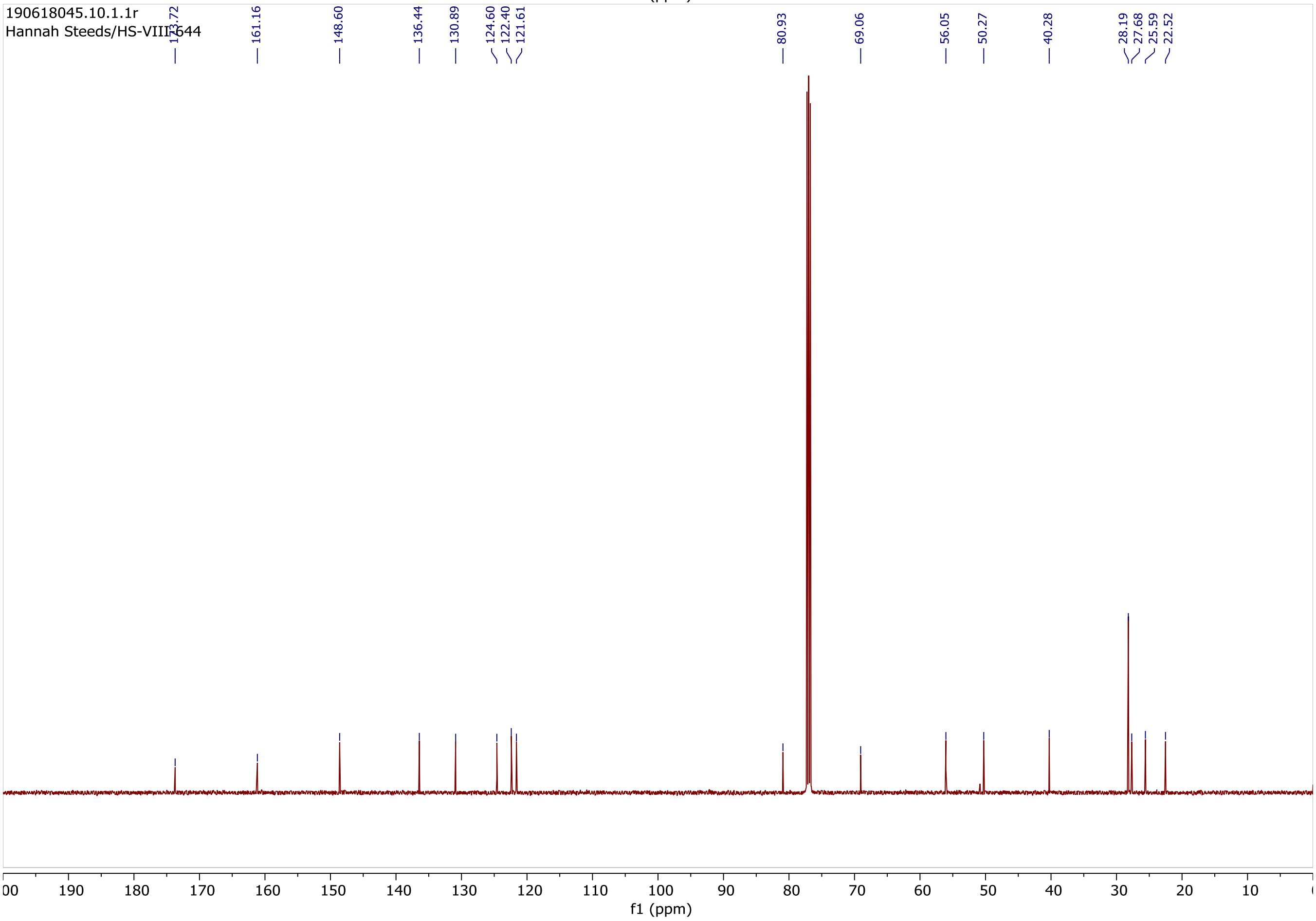

190618045.10.1.1r
Hannah Steeds/HS-VIII-644
173.72 161.16 148.60 136.44 130.89 124.60 122.40 121.61 80.93 69.06 56.05 50.27 40.28 28.19 27.68 25.59 22.52
f1 (ppm)

CDCl$_3$

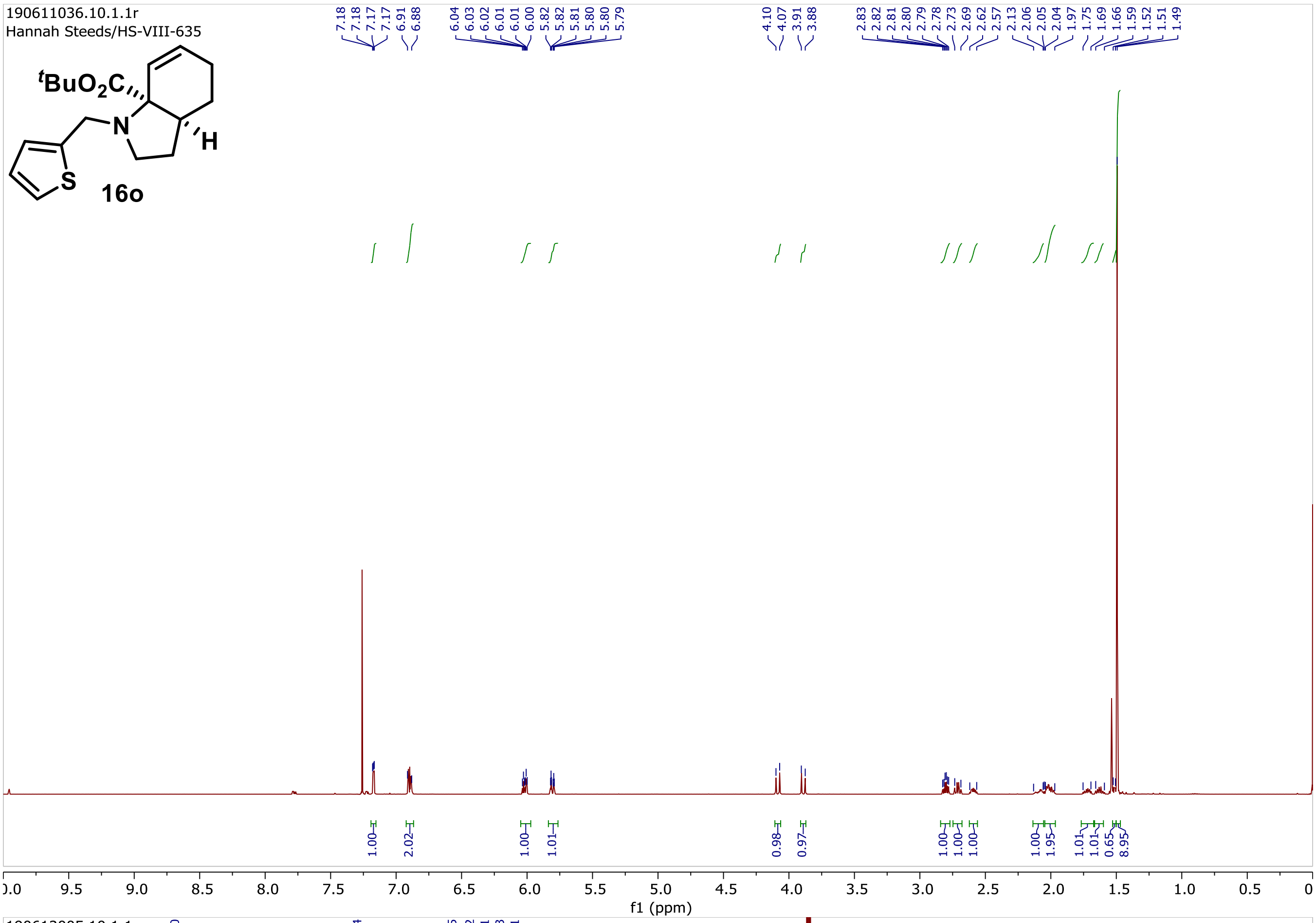
190611036.10.1.1r
Hannah Steeds/HS-VIII-635
^{t}BuO$_2$C
N
H
S
16o
f1 (ppm)

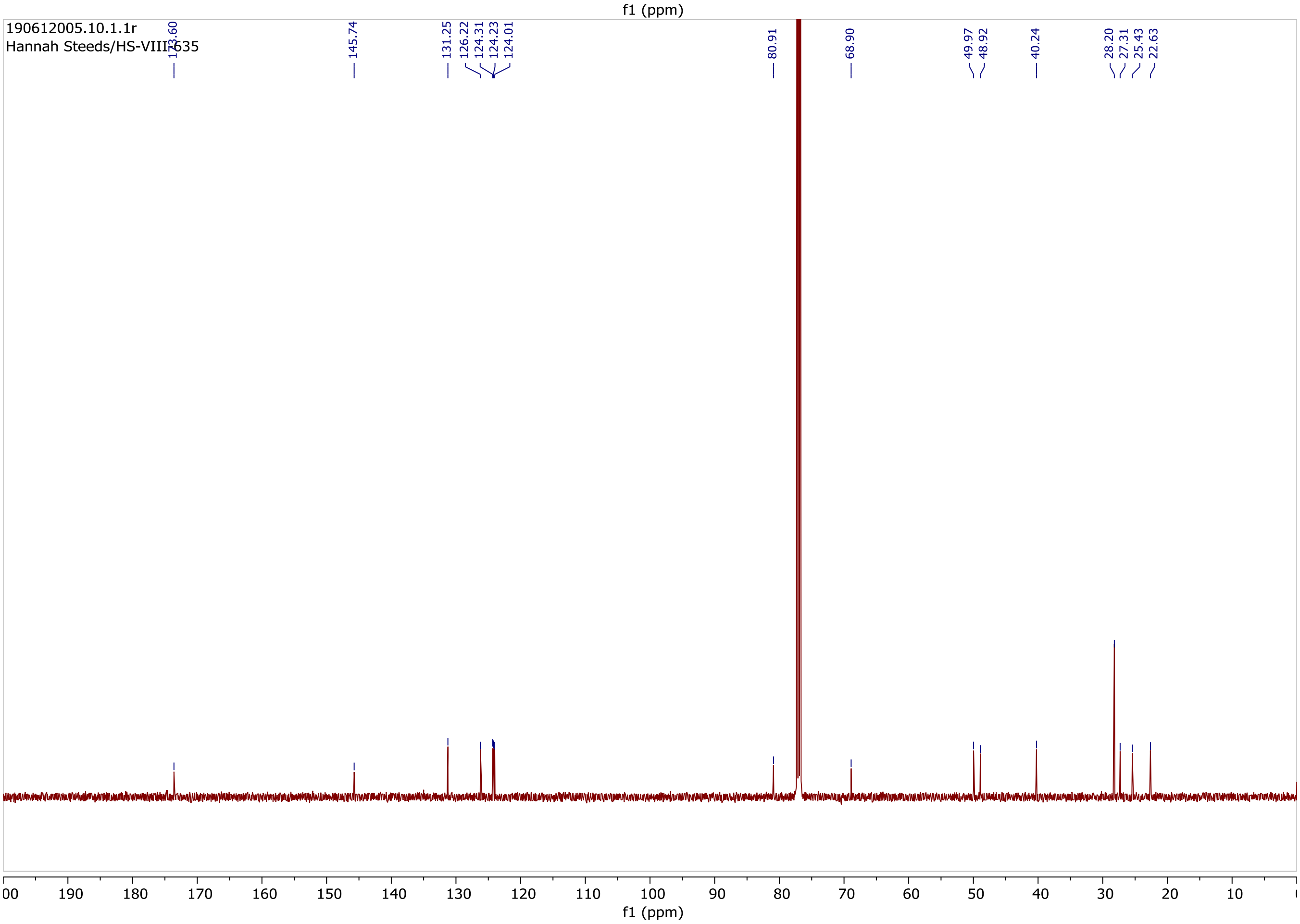
190612005.10.1.1r
Hannah Steeds/HS-VIII-635
f1 (ppm)

CDCl$_3$

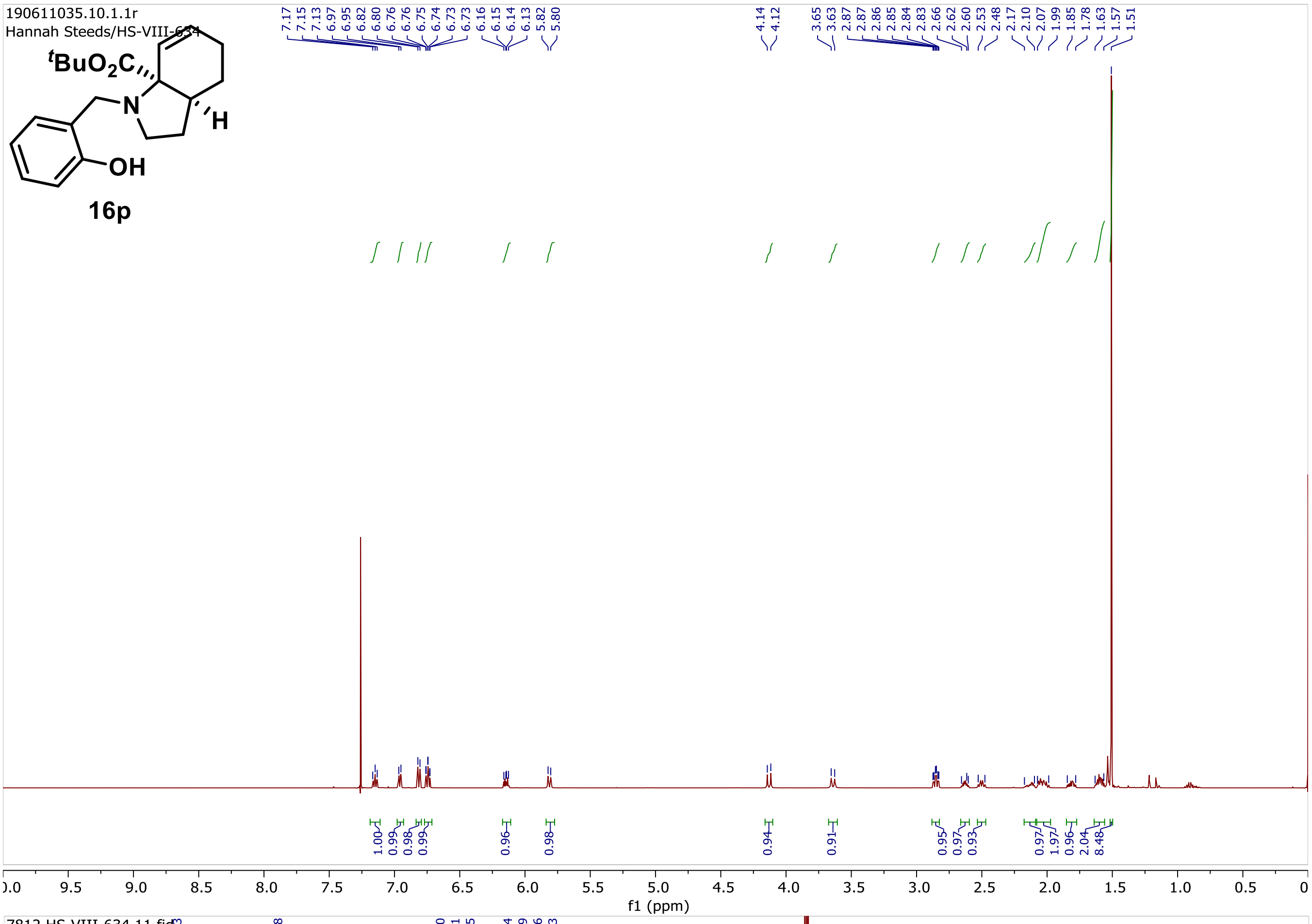

190611035.10.1.1r
Hannah Steeds/HS-VIII-634
^{t}BuO$_2$C
N
H
OH
16p
7.17 7.15 7.13 6.97 6.95 6.82 6.80 6.76 6.76 6.75 6.74 6.73 6.73 6.16 6.15 6.14 6.13 5.82 5.80
4.14 4.12
3.65 3.63 2.87 2.86 2.85 2.84 2.83 2.66 2.62 2.60 2.53 2.48 2.17 2.10 2.07 1.99 1.85 1.78 1.63 1.57 1.51
1.00 0.99 0.98 0.99 0.96 0.98 0.94 0.91 0.95 0.97 0.93 0.97 1.97 0.96 2.04 8.48
f1 (ppm)

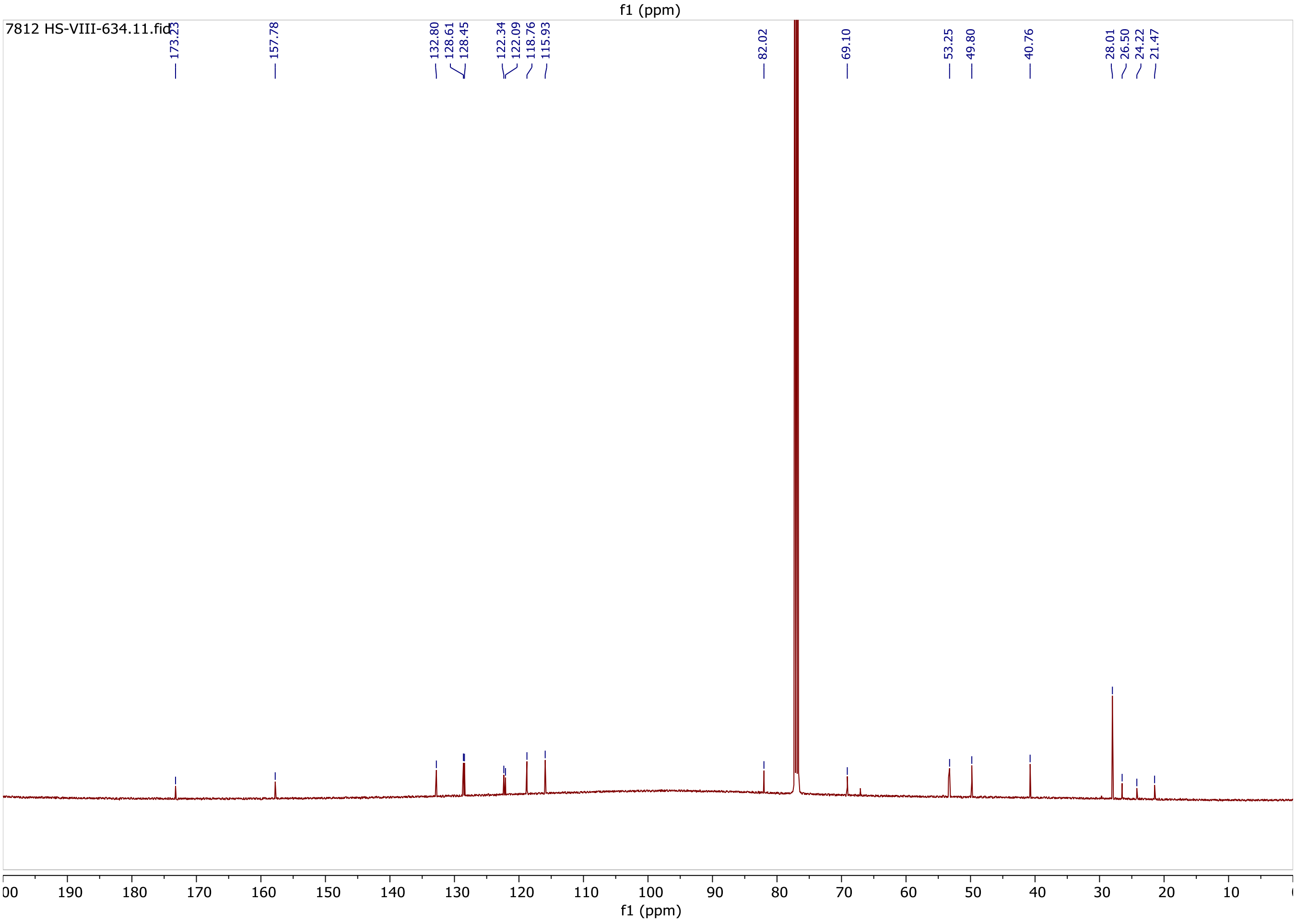

7812 HS-VIII-634.11.fid
173.23
157.78
132.80 128.61 128.45
122.34 122.09 118.76 115.93
82.02
69.10
53.25 49.80
40.76
28.01 26.50 24.22 21.47
f1 (ppm)

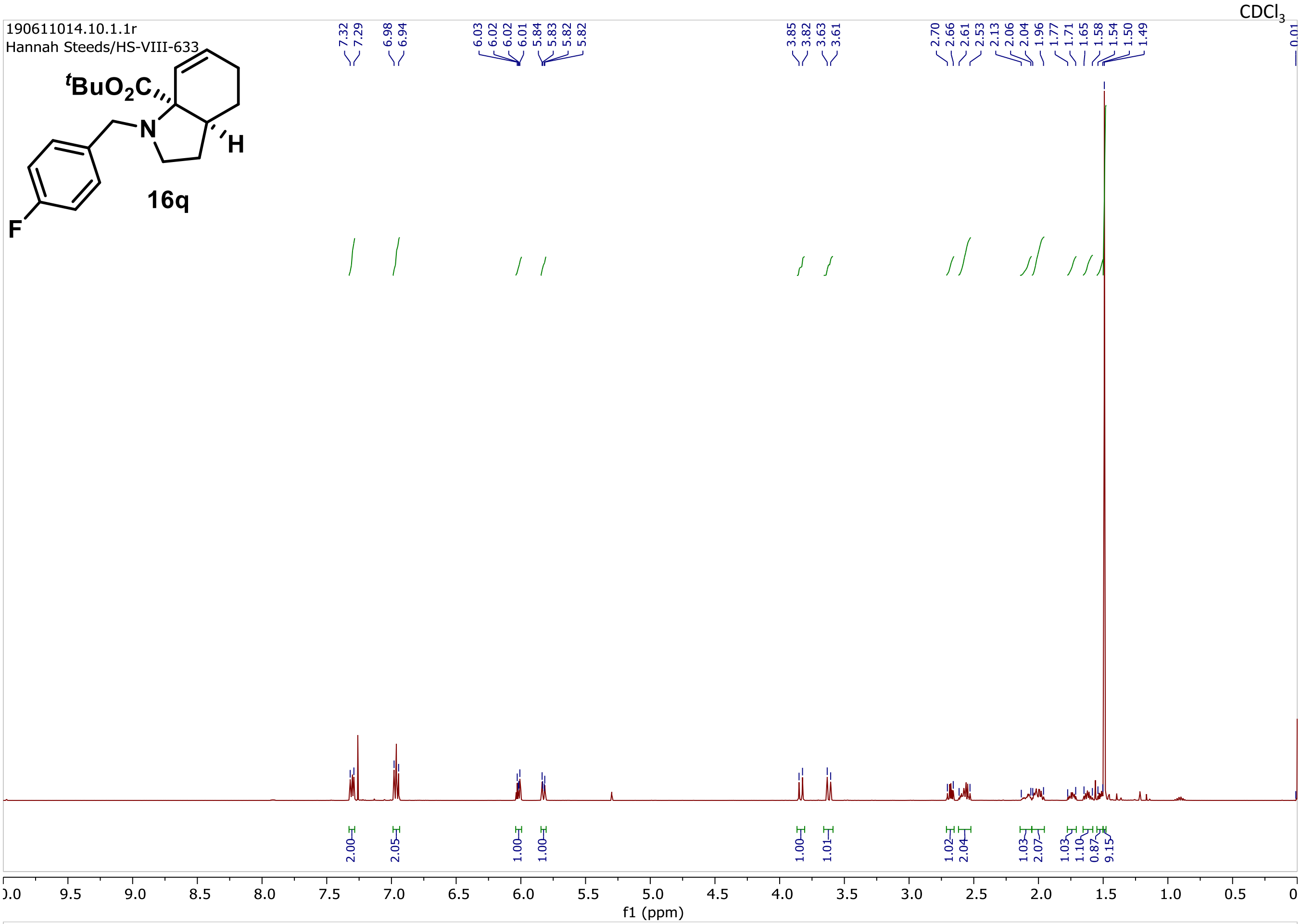

CDCl$_3$
190611014.10.1.1r
Hannah Steeds/HS-VIII-633
^{t}BuO$_2$C
N
H
F
16q
7.32 7.29 6.98 6.94 6.03 6.02 6.02 6.01 5.84 5.83 5.82 5.82 3.85 3.82 3.63 3.61 2.70 2.66 2.61 2.53 2.13 2.06 2.04 1.96 1.77 1.71 1.65 1.58 1.54 1.50 1.49
2.00 2.05 1.00 1.00 1.00 1.01 1.02 2.04 1.03 2.07 1.03 1.10 0.87 9.15
9.5 9.0 8.5 8.0 7.5 7.0 6.5 6.0 5.5 5.0 4.5 4.0 3.5 3.0 2.5 2.0 1.5 1.0 0.5
f1 (ppm)

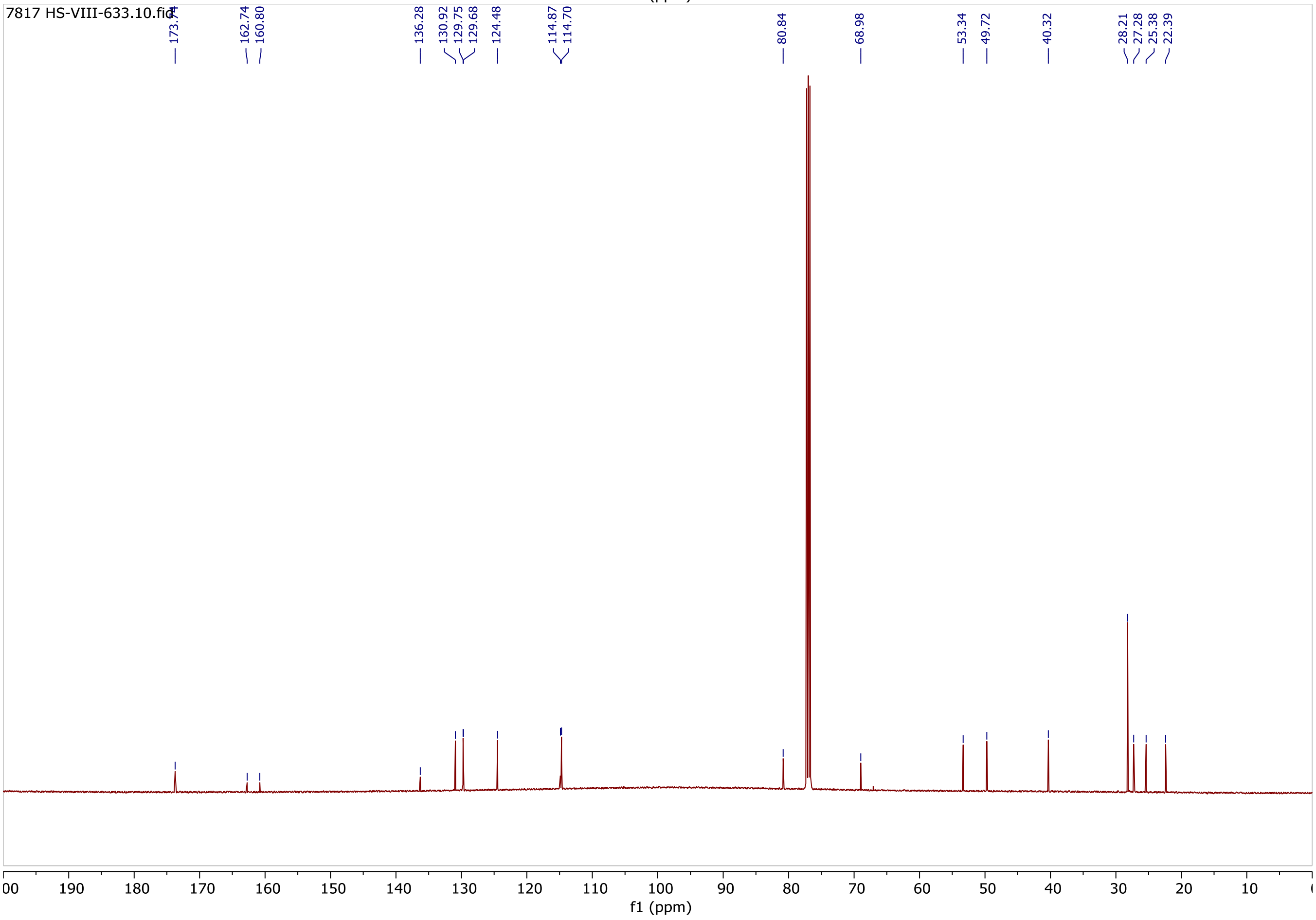

7817 HS-VIII-633.10.fid
162.74 160.80 136.28 130.92 129.75 129.68 124.48 114.87 114.70 80.84 68.98 53.34 49.72 40.32 28.21 27.28 25.38 22.39
190 180 170 160 150 140 130 120 110 100 90 80 70 60 50 40 30 20 10
f1 (ppm)

CDCl$_3$

kb/hs16963 HS-VIII-633

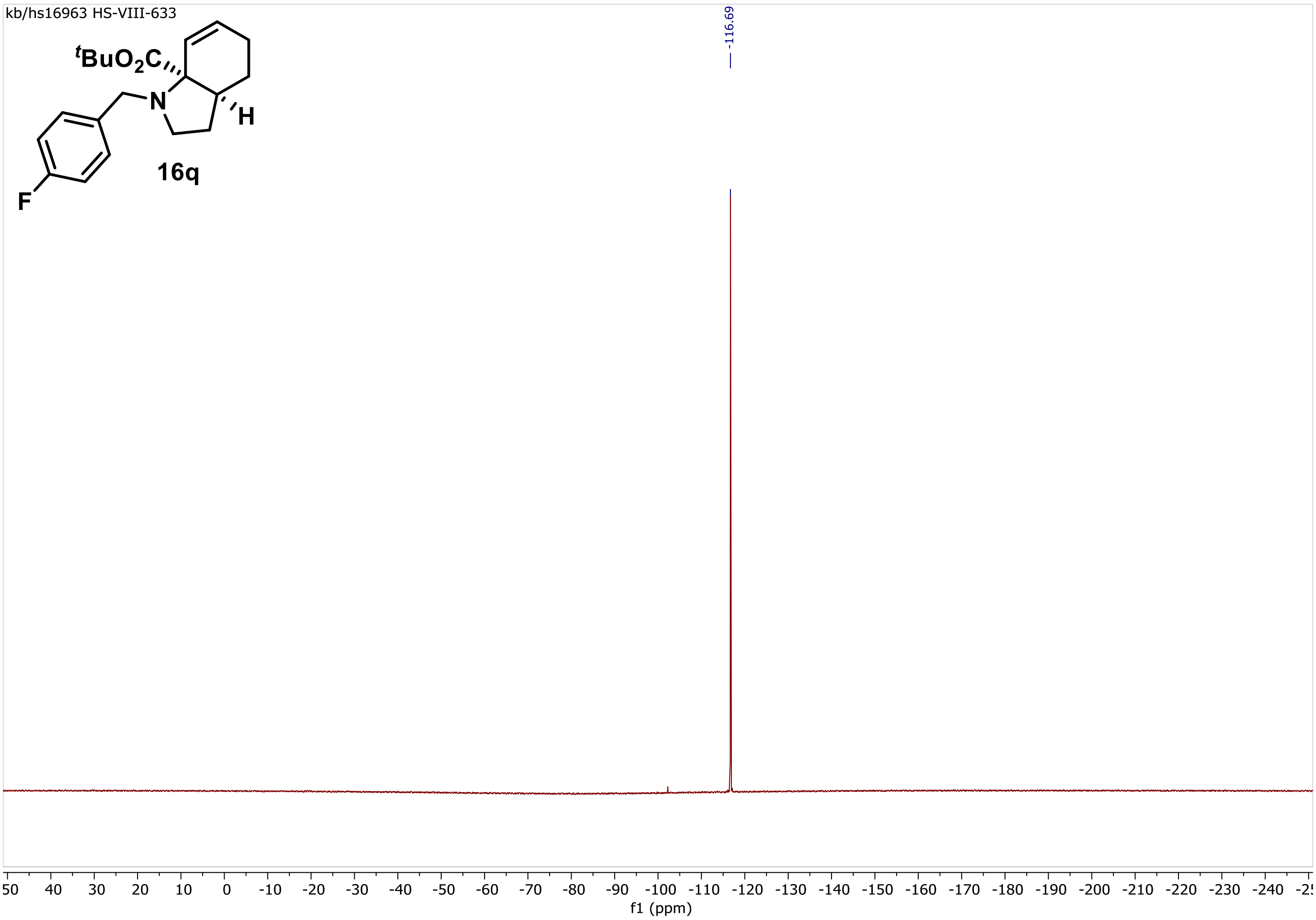

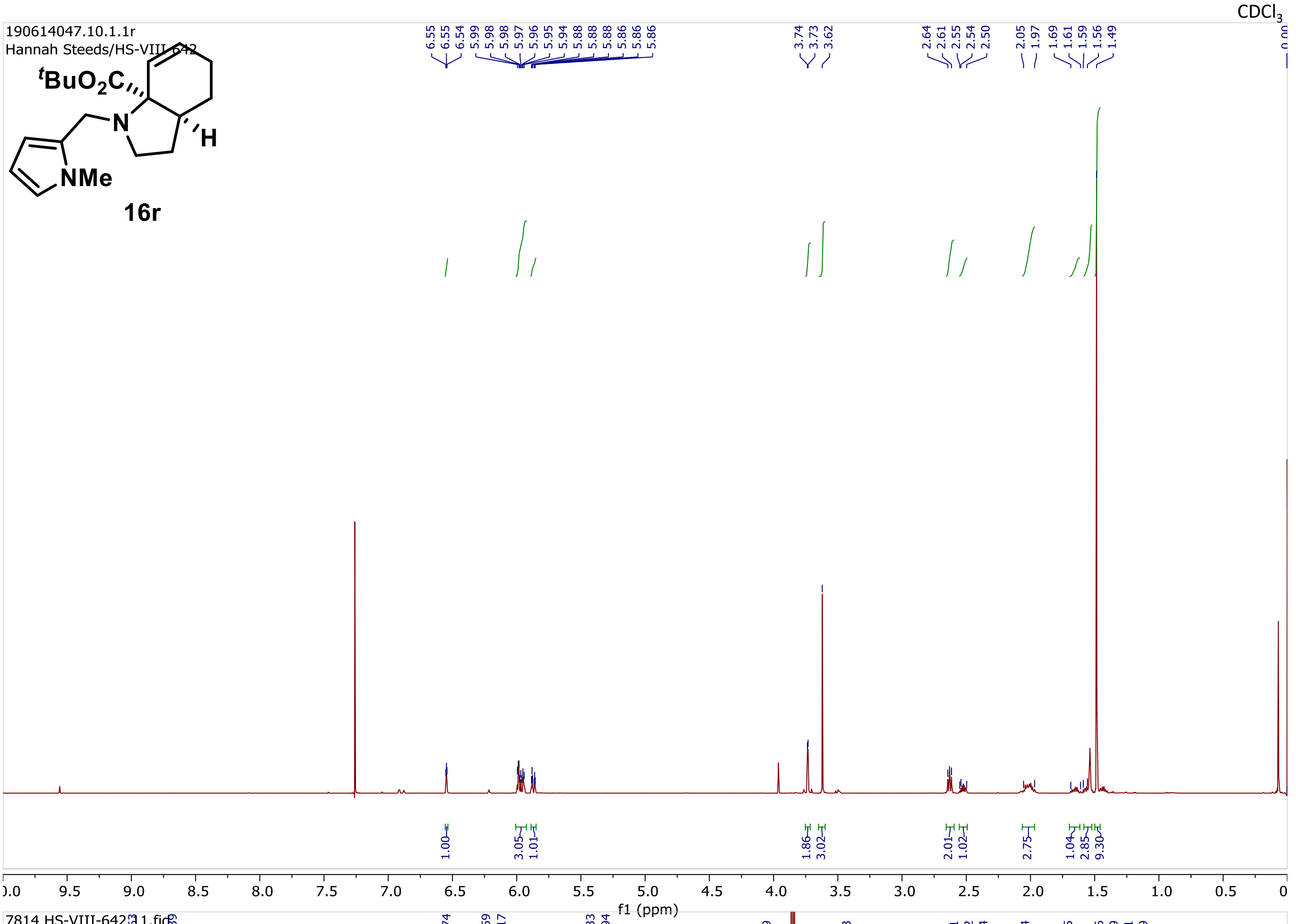
CDCl$_3$
190614047.10.1.1r
Hannah Steeds/HS-VIII-642
^{t}BuO$_2$C
N
NMe
H
16r
6.55 6.55 6.54 5.99 5.98 5.98 5.97 5.96 5.95 5.94 5.88 5.88 5.88 5.86 5.86 5.86
3.74 3.73 3.62
2.64 2.61 2.55 2.54 2.50
2.05 1.97 1.69 1.61 1.59 1.56 1.49
1.00 3.05 1.01 1.86 3.02 2.01 1.02 2.75 1.04 2.85 9.30
f1 (ppm)

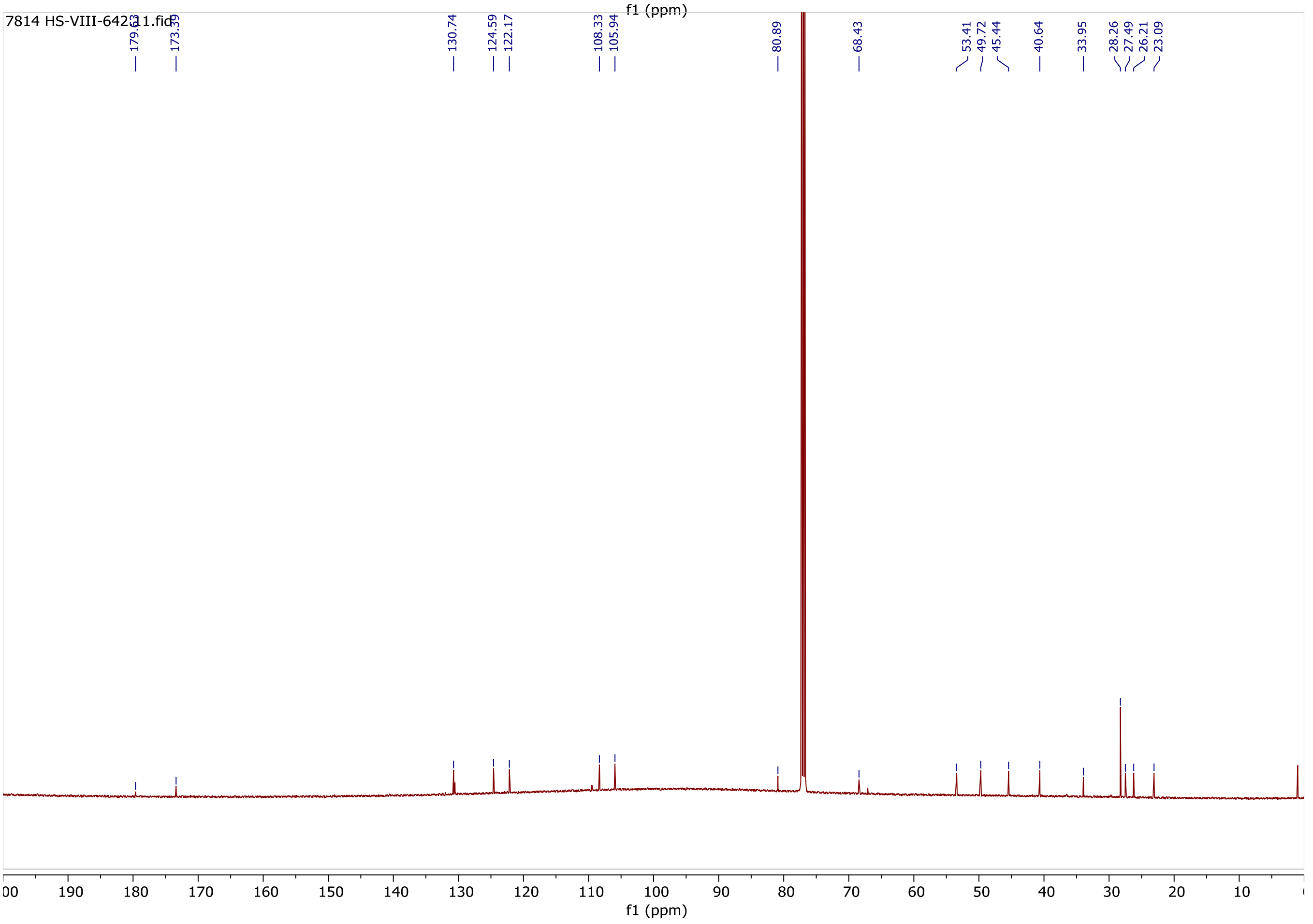
179.63 173.39
130.74 124.59 122.17
108.33 105.94
80.89 68.43
53.41 49.72 45.44 40.64 33.95 28.26 27.49 26.21 23.09
f1 (ppm)

CDCl$_3$

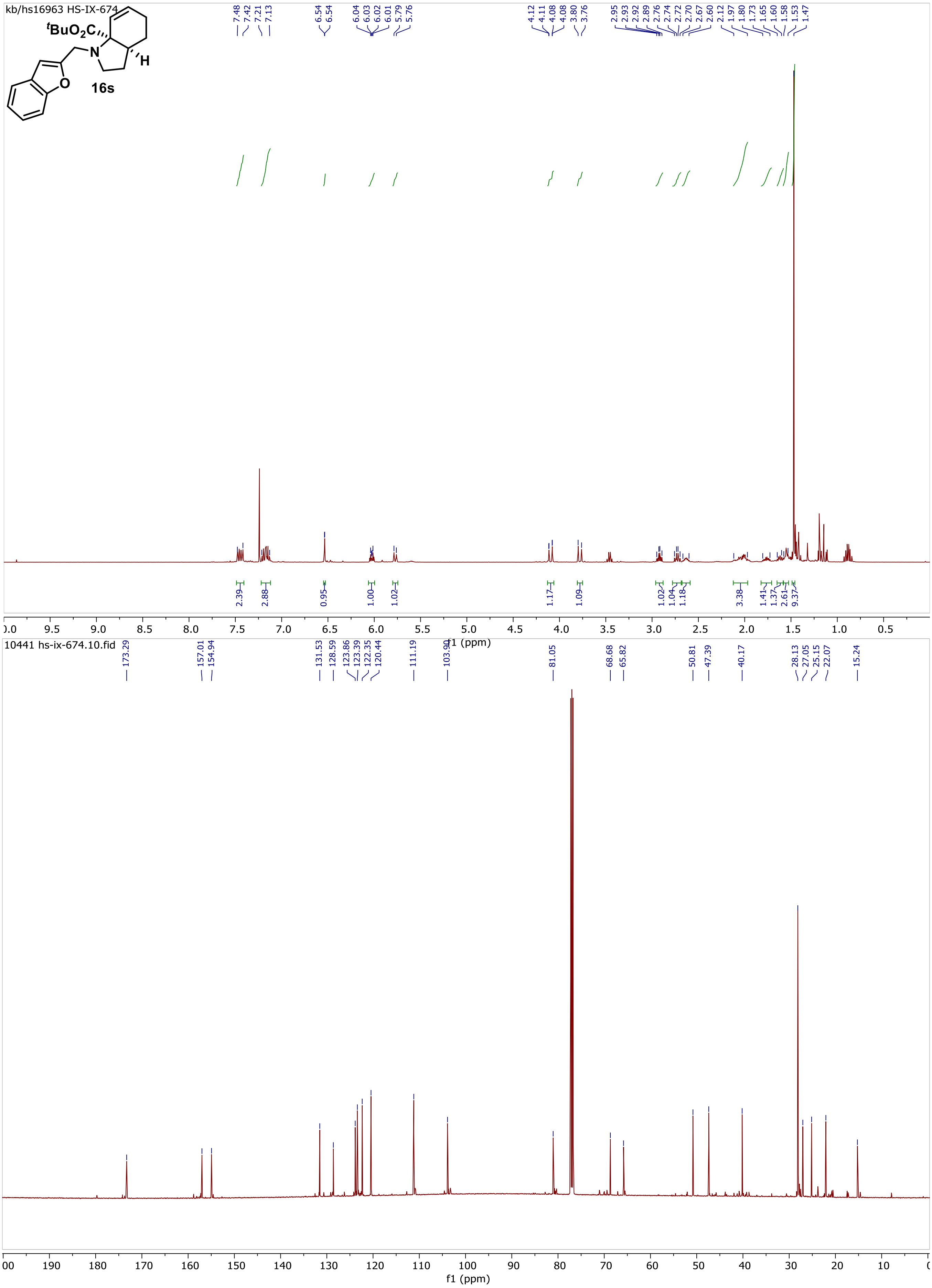
kb/hs16963 HS-IX-674
^{t}BuO$_2$C
N
H
O
16s
7.48 7.42 7.21 7.13 6.54 6.54 6.04 6.03 6.02 6.01 5.79 5.76 4.12 4.11 4.08 4.08 3.80 3.76 2.95 2.93 2.92 2.89 2.76 2.74 2.72 2.70 2.67 2.60 2.12 1.97 1.80 1.73 1.65 1.60 1.58 1.53 1.47
2.39 2.88 0.95 1.00 1.02 1.17 1.09 1.02 1.04 1.18 3.38 1.41 1.37 2.61 9.37
f1 (ppm)
10441 hs-ix-674.10.fid
173.29 157.01 154.94 131.53 128.59 123.86 123.39 122.35 120.44 111.19 103.90 81.05 68.68 65.82 50.81 47.39 40.17 28.13 27.05 25.15 22.07 15.24
f1 (ppm)

CDCl$_3$

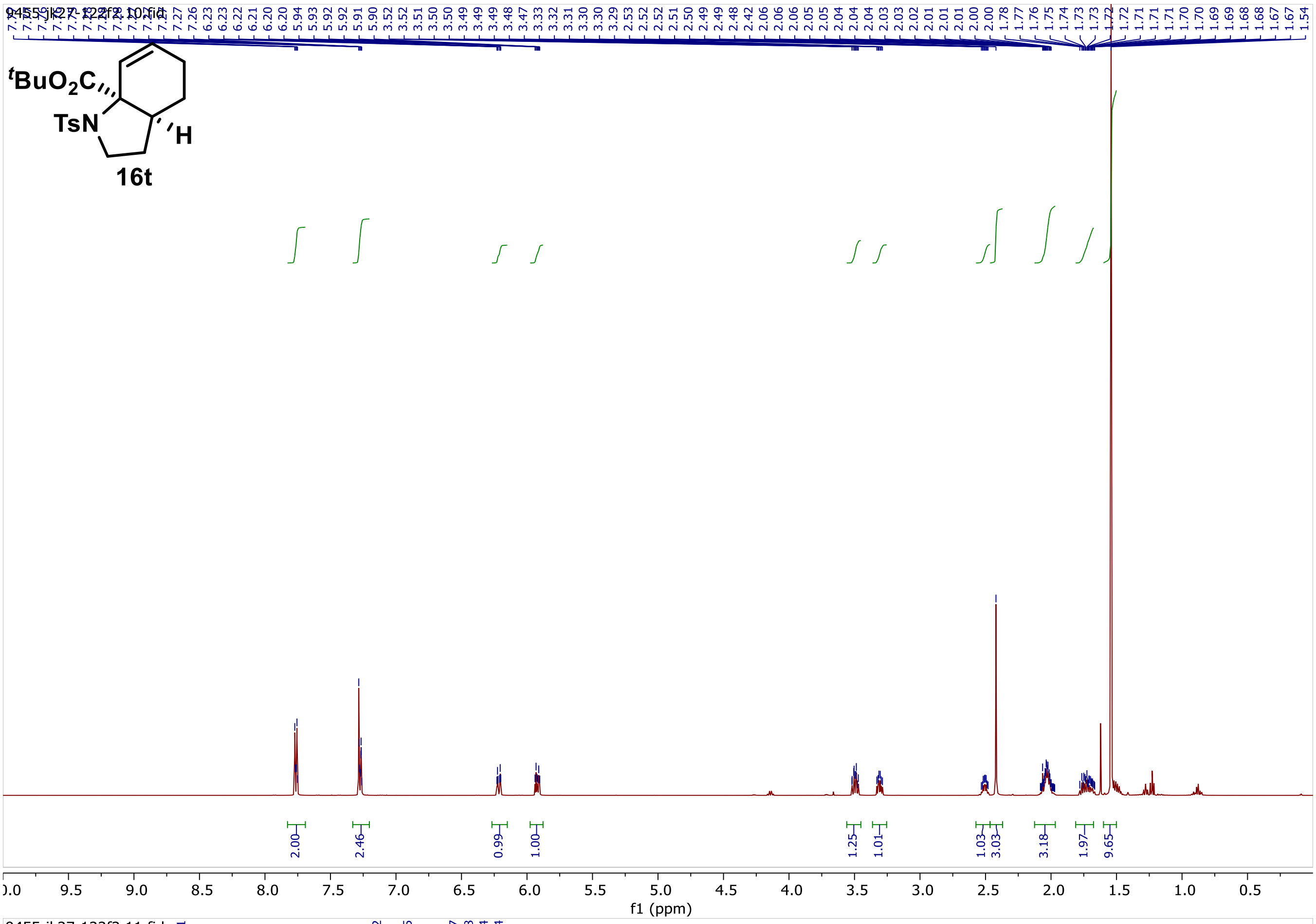
9455 jk27-122f2.10.fid
7.27
7.26
6.23
6.23
6.22
6.21
6.20
6.20
5.94
5.93
5.92
5.92
5.91
5.90
3.52
3.52
3.51
3.50
3.50
3.49
3.49
3.48
3.47
3.33
3.32
3.31
3.30
3.30
3.29
2.53
2.52
2.52
2.51
2.50
2.49
2.49
2.48
2.42
2.06
2.06
2.06
2.05
2.05
2.04
2.04
2.04
2.03
2.03
2.02
2.01
2.01
2.01
2.00
1.78
1.77
1.76
1.75
1.74
1.73
1.73
1.73
1.72
1.72
1.71
1.71
1.71
1.70
1.69
1.68
1.67
1.67
1.54
^{t}BuO$_2$C
TsN
H
16t
2.00
2.46
0.99
1.00
1.25
1.01
1.03
3.03
3.18
1.97
9.65
9.5
9.0
8.5
8.0
7.5
7.0
6.5
6.0
5.5
5.0
4.5
4.0
3.5
3.0
2.5
2.0
1.5
1.0
0.5
f1 (ppm)

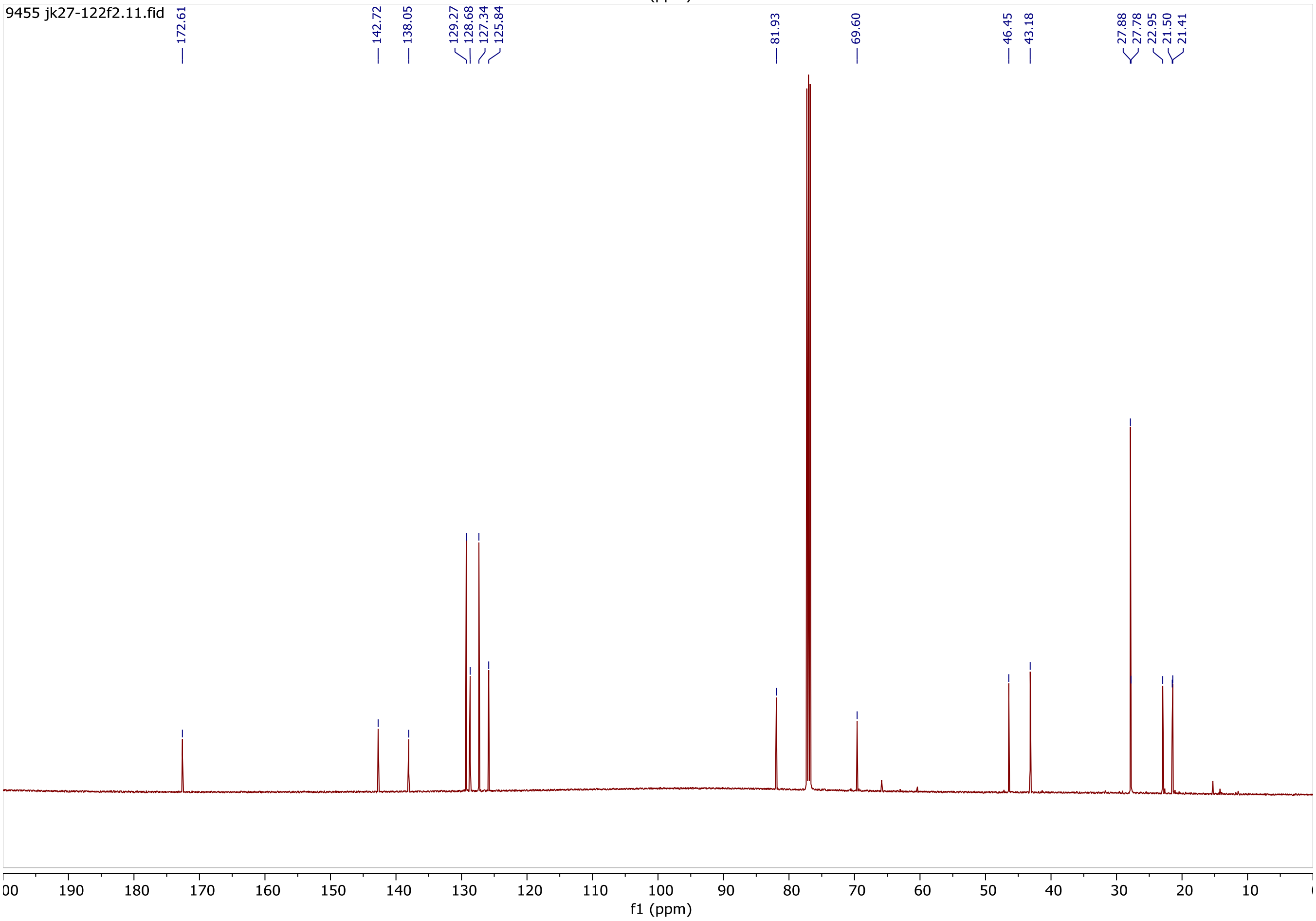
9455 jk27-122f2.11.fid
172.61
142.72
138.05
129.27
128.68
127.34
125.84
81.93
69.60
46.45
43.18
27.88
27.78
22.95
21.50
21.41
190
180
170
160
150
140
130
120
110
100
90
80
70
60
50
40
30
20
10
f1 (ppm)

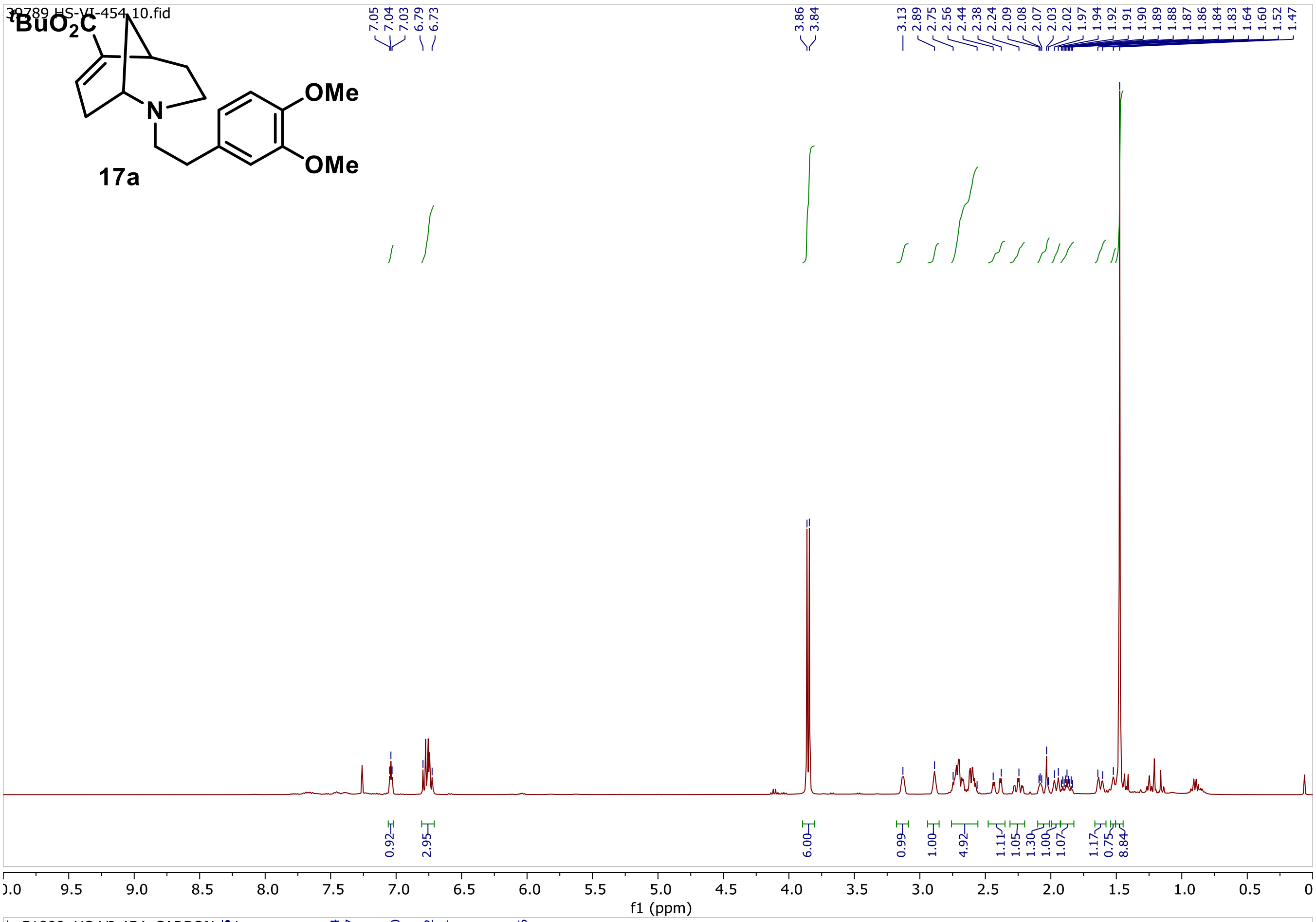
39789_HS-VI-454_10.fid
^{t}BuO$_2$C
N
OMe
OMe
17a
7.05 7.04 7.03 6.79 6.73
3.86 3.84
3.13 2.89 2.75 2.56 2.44 2.38 2.24 2.09 2.08 2.07 2.03 2.02 1.97 1.94 1.92 1.91 1.90 1.89 1.88 1.87 1.86 1.84 1.83 1.64 1.60 1.52 1.47
0.92 2.95 6.00 0.99 1.00 4.92 1.11 1.05 1.30 1.00 1.07 1.17 0.75 8.84
f1 (ppm)

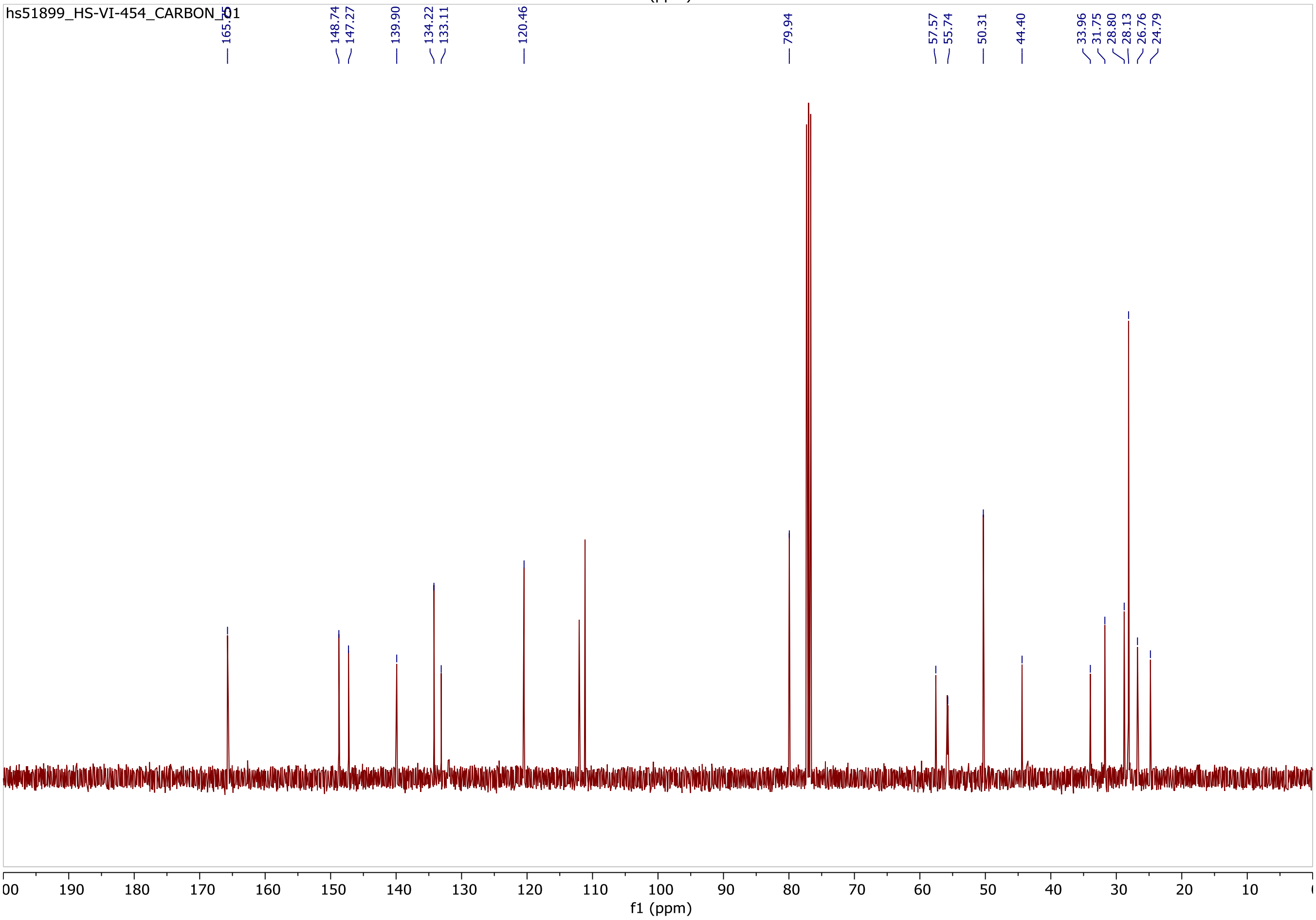
hs51899_HS-VI-454_CARBON_01
165.7
148.74 147.27
139.90
134.22 133.11
120.46
79.94
57.57 55.74
50.31
44.40
33.96 31.75 28.80 28.13 26.76 24.79
f1 (ppm)

CDCl$_3$

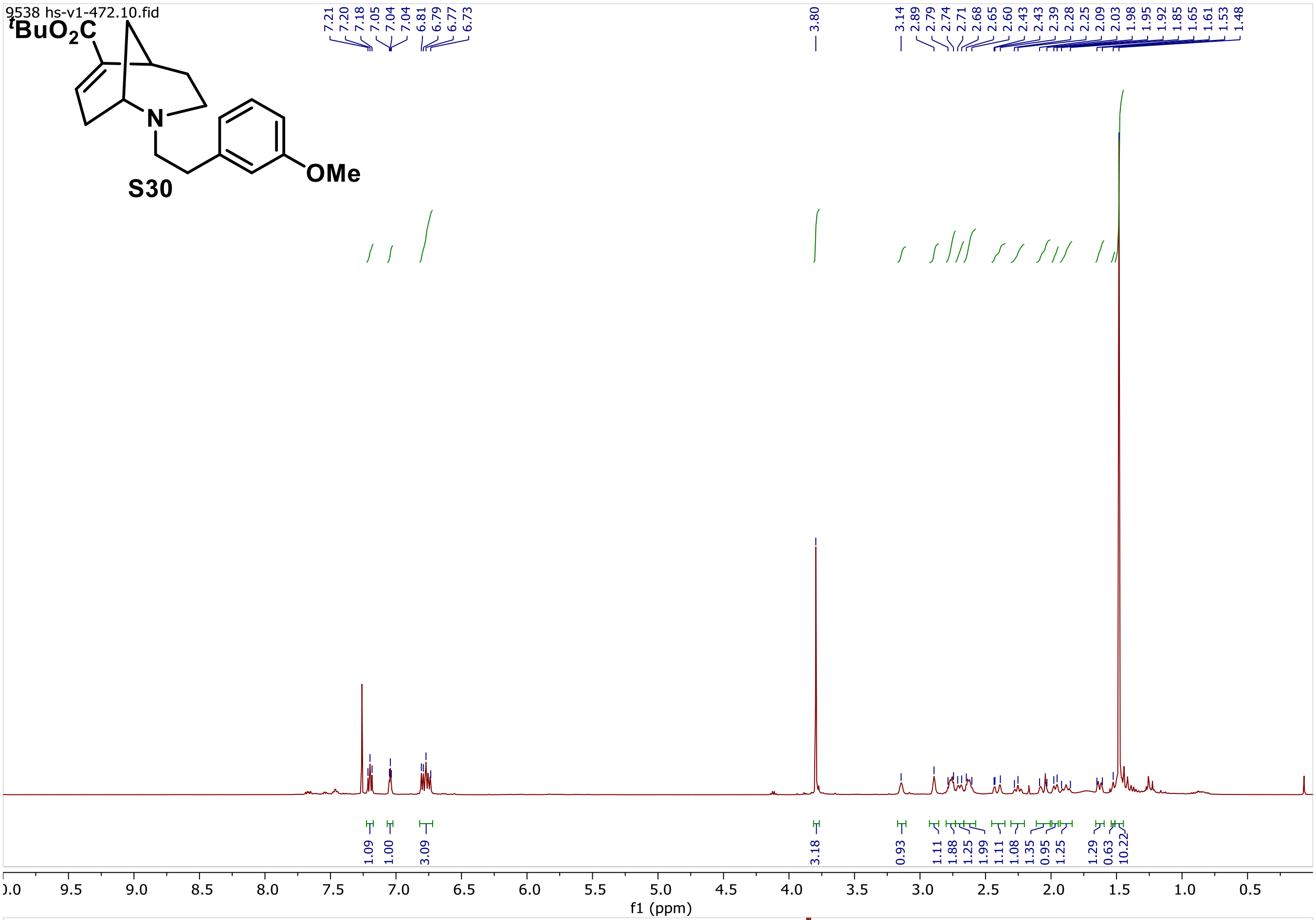
9538 hs-v1-472.10.fid
^{t}BuO$_2$C
N
OMe
S30
7.21 7.20 7.18 7.05 7.04 7.04 6.81 6.79 6.77 6.73
3.80
3.14 2.89 2.79 2.74 2.71 2.68 2.65 2.60 2.43 2.43 2.39 2.28 2.25 2.09 2.03 1.98 1.95 1.92 1.85 1.65 1.61 1.53 1.48
1.09 1.00 3.09 3.18 0.93 1.11 1.88 1.25 1.99 1.11 1.08 1.35 0.95 1.25 1.29 0.63 10.22
9.5 9.0 8.5 8.0 7.5 7.0 6.5 6.0 5.5 5.0 4.5 4.0 3.5 3.0 2.5 2.0 1.5 1.0 0.5
f1 (ppm)

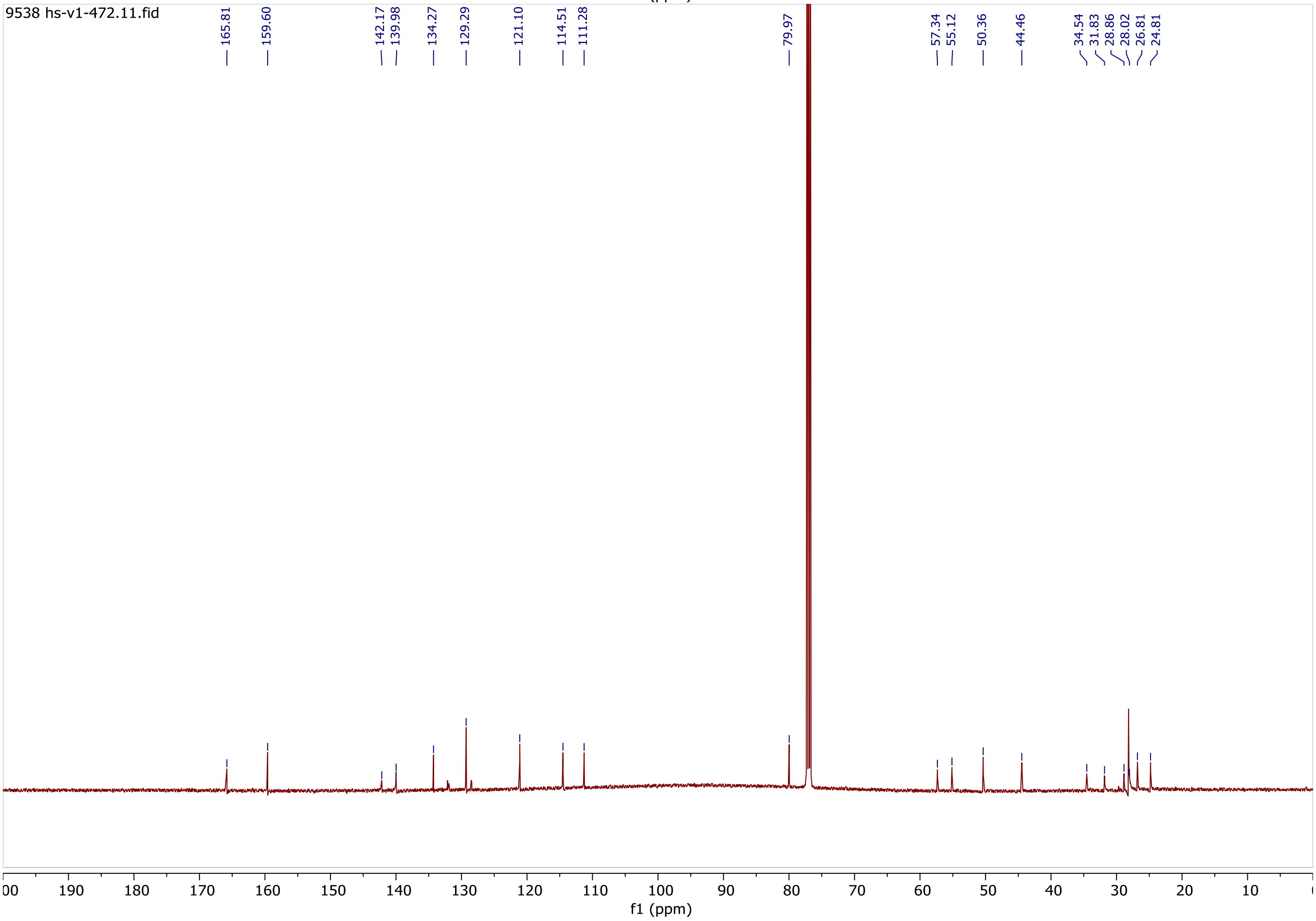
9538 hs-v1-472.11.fid
165.81 159.60 142.17 139.98 134.27 129.29 121.10 114.51 111.28 79.97 57.34 55.12 50.36 44.46 34.54 31.83 28.86 28.02 26.81 24.81
190 180 170 160 150 140 130 120 110 100 90 80 70 60 50 40 30 20 10
f1 (ppm)

CDCl$_3$

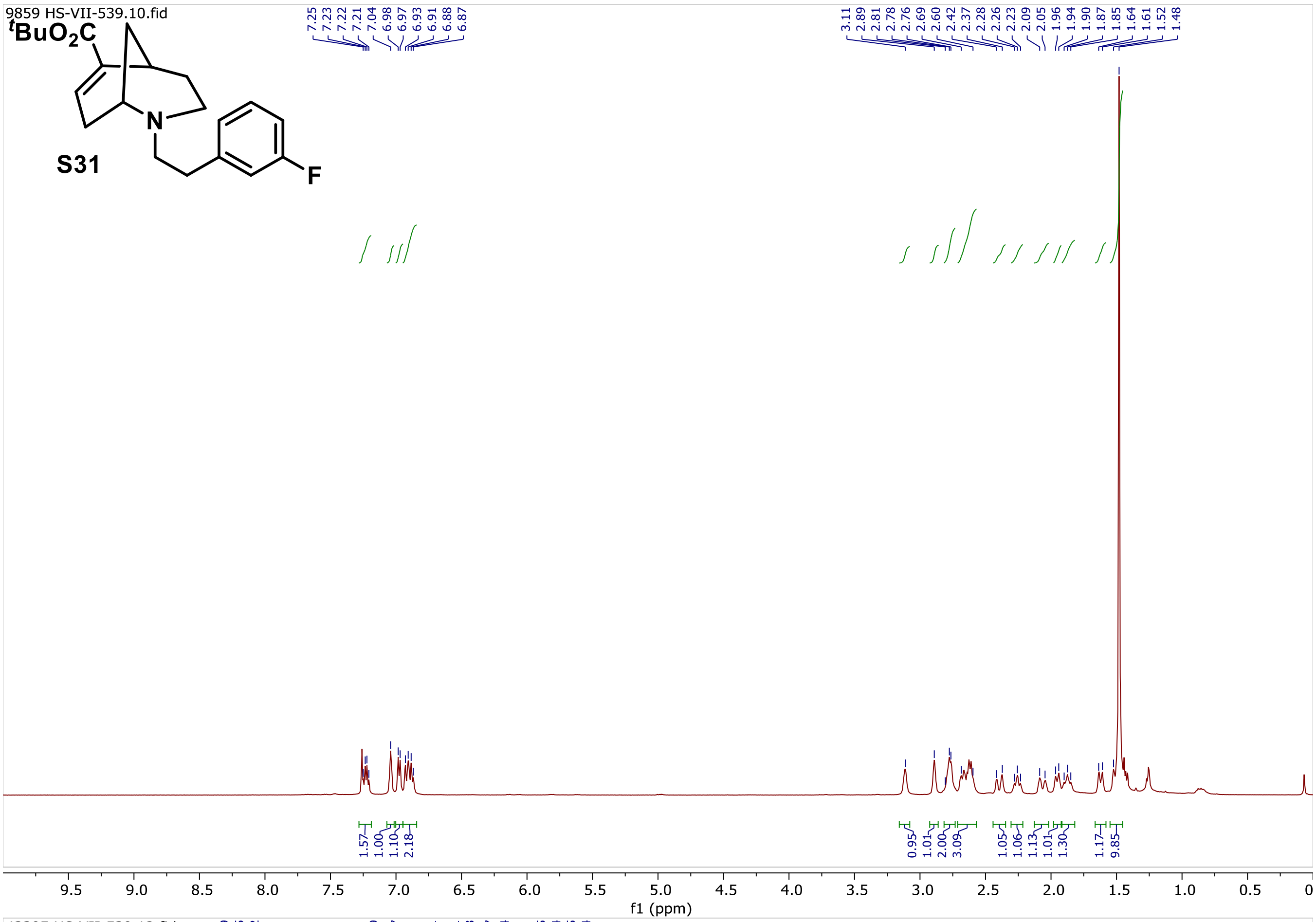
9859 HS-VII-539.10.fid
^{t}BuO$_2$C
N
F
S31
7.25 7.23 7.22 7.21 7.04 6.98 6.97 6.93 6.91 6.88 6.87
3.11 2.89 2.81 2.78 2.76 2.69 2.60 2.42 2.37 2.28 2.26 2.23 2.09 2.05 1.96 1.94 1.90 1.87 1.85 1.64 1.61 1.52 1.48
1.57 1.00 1.10 2.18
0.95 1.01 2.00 3.09 1.05 1.06 1.13 1.01 1.30 1.17 9.85
9.5 9.0 8.5 8.0 7.5 7.0 6.5 6.0 5.5 5.0 4.5 4.0 3.5 3.0 2.5 2.0 1.5 1.0 0.5 0
f1 (ppm)

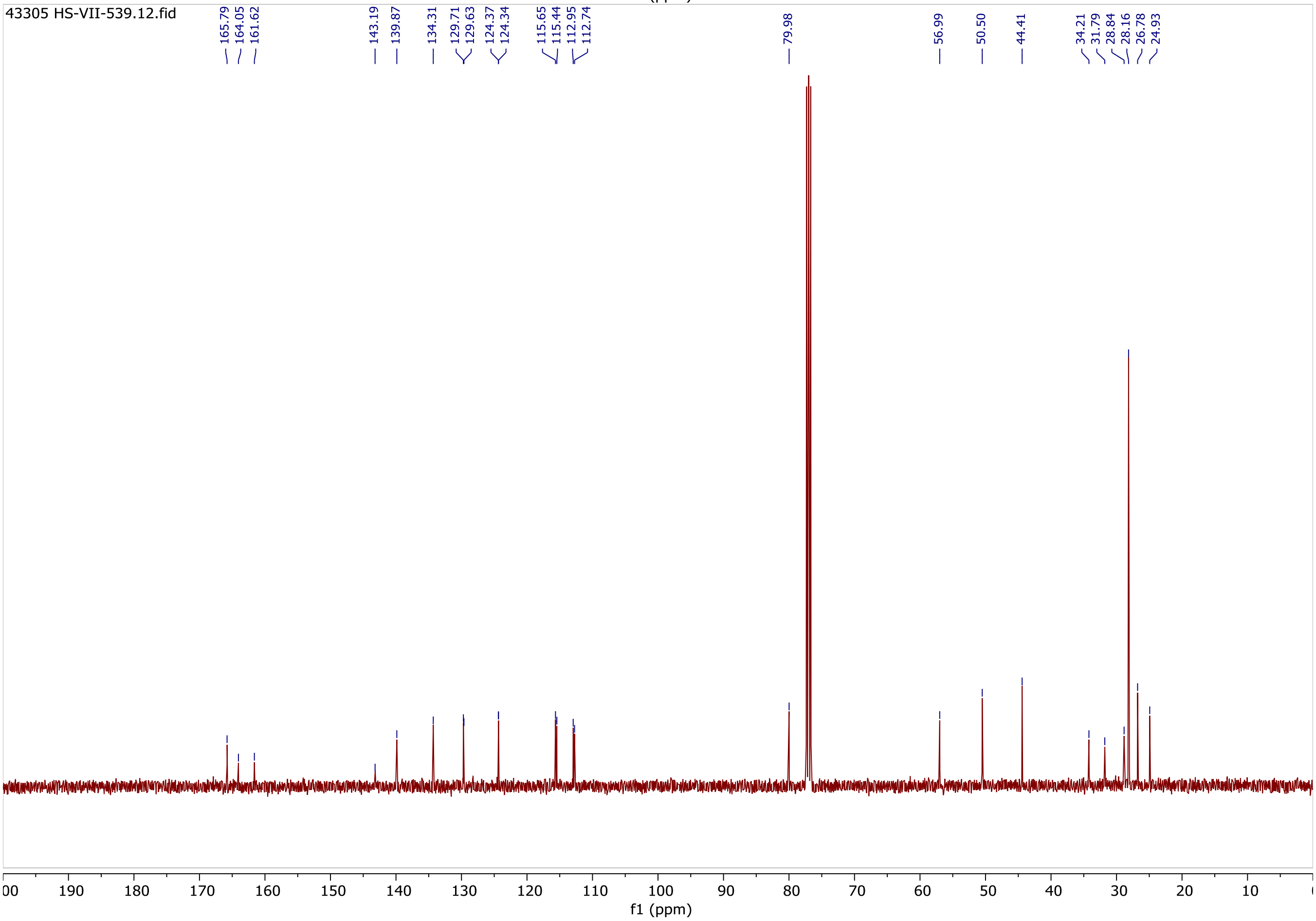
43305 HS-VII-539.12.fid
165.79 164.05 161.62
143.19 139.87 134.31 129.71 129.63 124.37 124.34
115.65 115.44 112.95 112.74
79.98
56.99 50.50 44.41
34.21 31.79 28.84 28.16 26.78 24.93
190 180 170 160 150 140 130 120 110 100 90 80 70 60 50 40 30 20 10
f1 (ppm)

CDCl$_3$

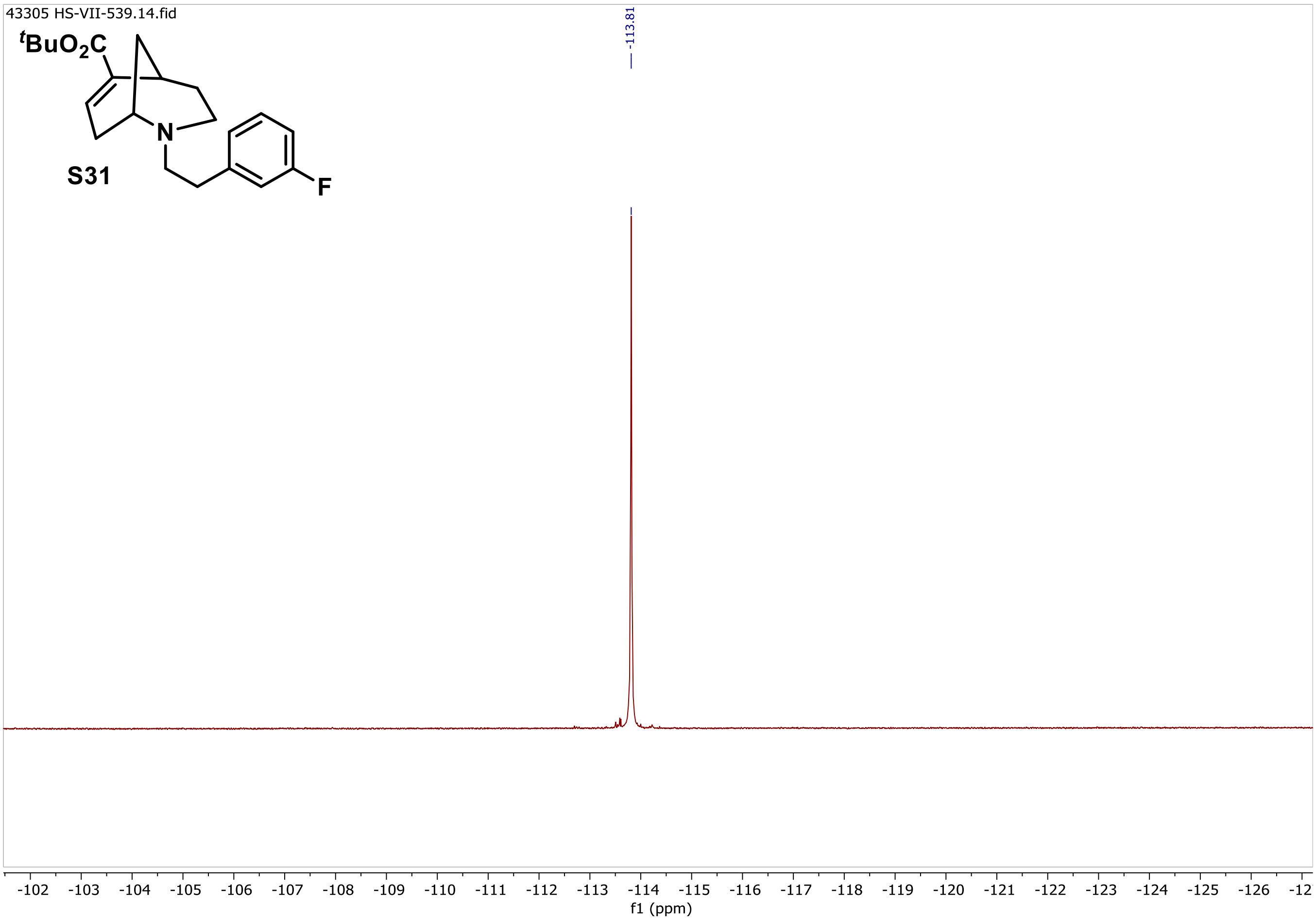
43305 HS-VII-539.14.fid
tBuO2C
N
F
S31
-113.81
-102 -103 -104 -105 -106 -107 -108 -109 -110 -111 -112 -113 -114 -115 -116 -117 -118 -119 -120 -121 -122 -123 -124 -125 -126 -12
f1 (ppm)

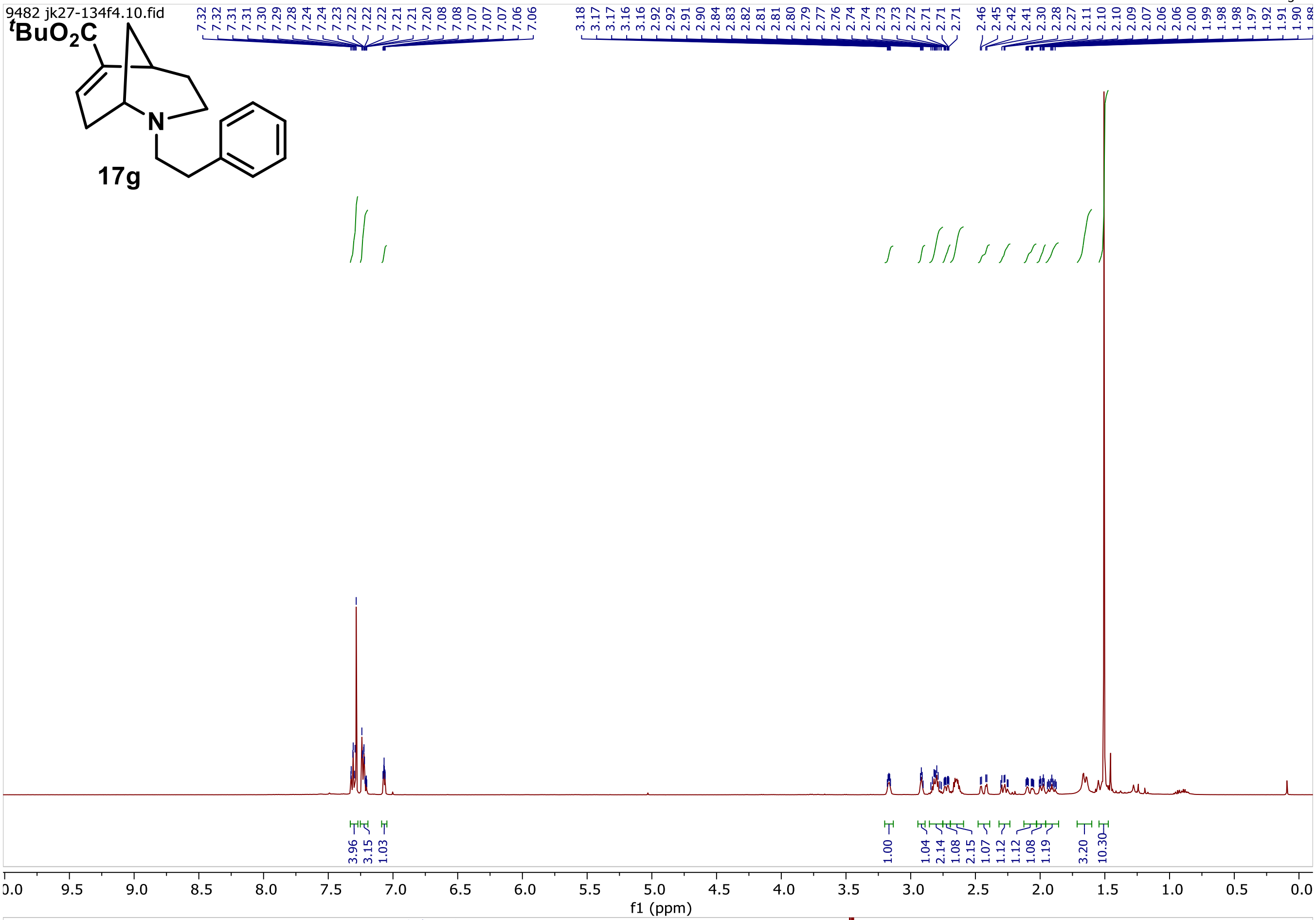

$^tBuO_2C$
N
17g
CDCl3
9482 jk27-134f4.10.fid
7.32 7.32 7.31 7.31 7.30 7.29 7.28 7.24 7.24 7.23 7.22 7.22 7.22 7.21 7.21 7.20 7.08 7.07 7.07 7.07 7.06 7.06
3.18 3.17 3.17 3.16 2.92 2.92 2.91 2.90 2.84 2.83 2.82 2.81 2.80 2.79 2.77 2.76 2.74 2.74 2.73 2.73 2.72 2.71 2.71 2.71
2.46 2.45 2.42 2.41 2.30 2.28 2.27 2.11 2.10 2.10 2.09 2.07 2.06 2.06 2.00 1.99 1.98 1.98 1.97 1.92 1.91 1.90
3.96 3.15 1.03 1.00 1.04 2.14 1.08 2.15 1.07 1.12 1.12 1.08 1.19 3.20 10.30
9.5 9.0 8.5 8.0 7.5 7.0 6.5 6.0 5.5 5.0 4.5 4.0 3.5 3.0 2.5 2.0 1.5 1.0 0.5 0.0
f1 (ppm)

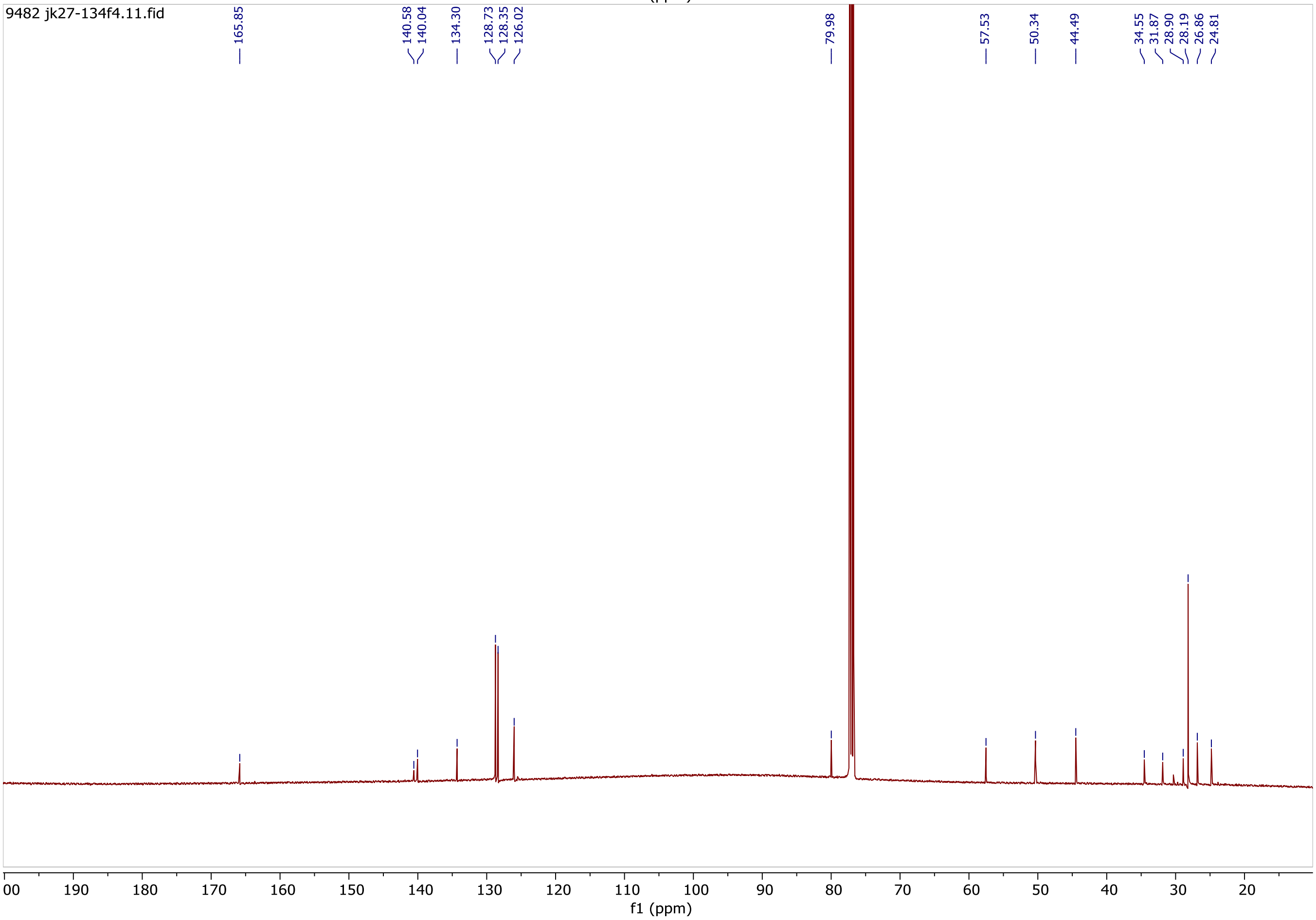

9482 jk27-134f4.11.fid
165.85
140.58 140.04
134.30
128.73 128.35 126.02
79.98
57.53
50.34
44.49
34.55 31.87 28.90 28.19 26.86 24.81
190 180 170 160 150 140 130 120 110 100 90 80 70 60 50 40 30 20
f1 (ppm)

CDCl$_3$

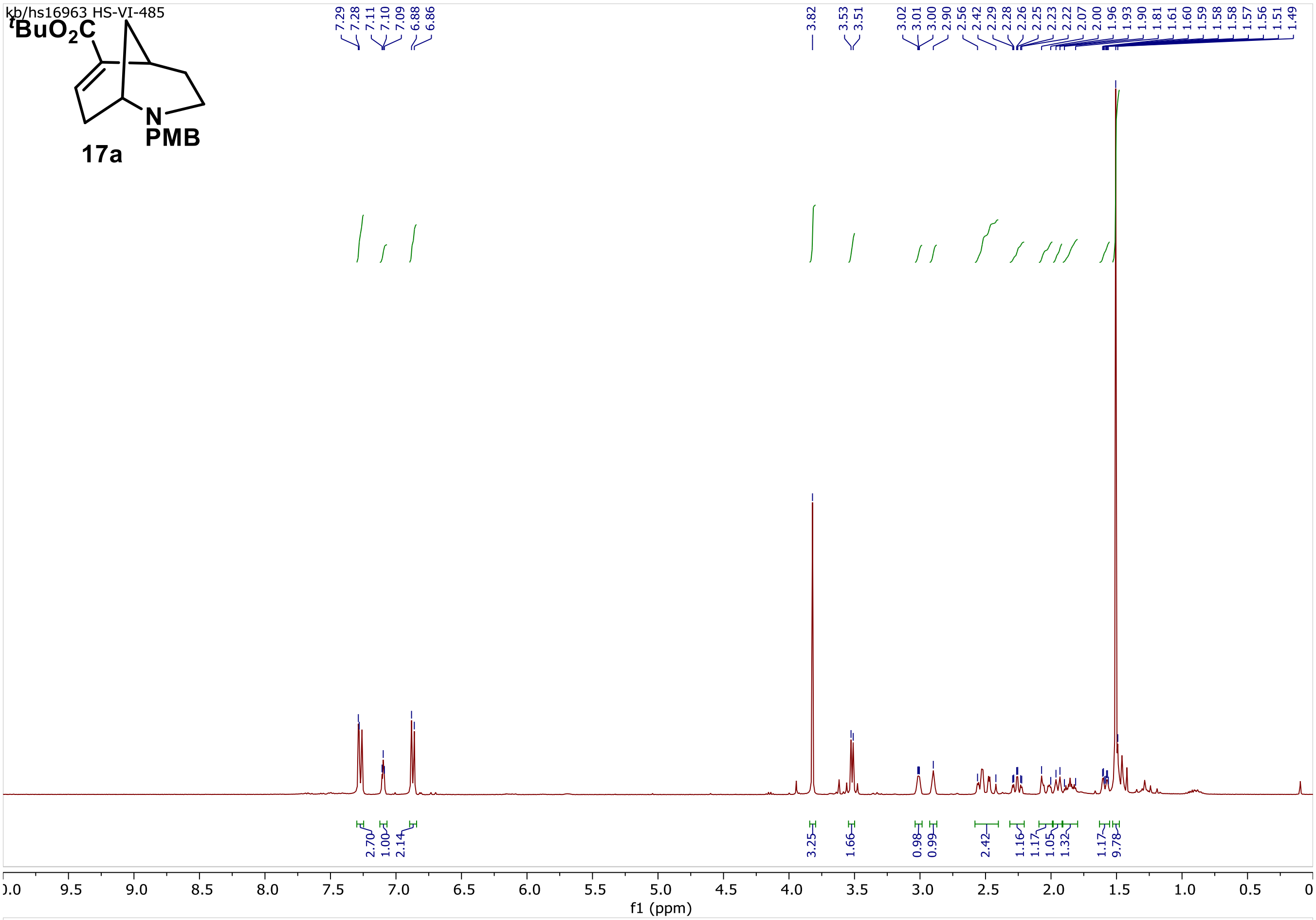

kb/hs16963 HS-VI-485
^{t}BuO$_2$C
N
PMB
17a
7.29 7.28 7.11 7.10 7.09 6.88 6.86 3.82 3.53 3.51 3.02 3.01 3.00 2.90 2.56 2.42 2.29 2.28 2.26 2.25 2.23 2.22 2.07 2.00 1.96 1.93 1.90 1.81 1.61 1.60 1.59 1.58 1.57 1.56 1.51 1.49
2.70 1.00 2.14 3.25 1.66 0.98 0.99 2.42 1.16 1.17 1.05 1.32 1.17 9.78
f1 (ppm)

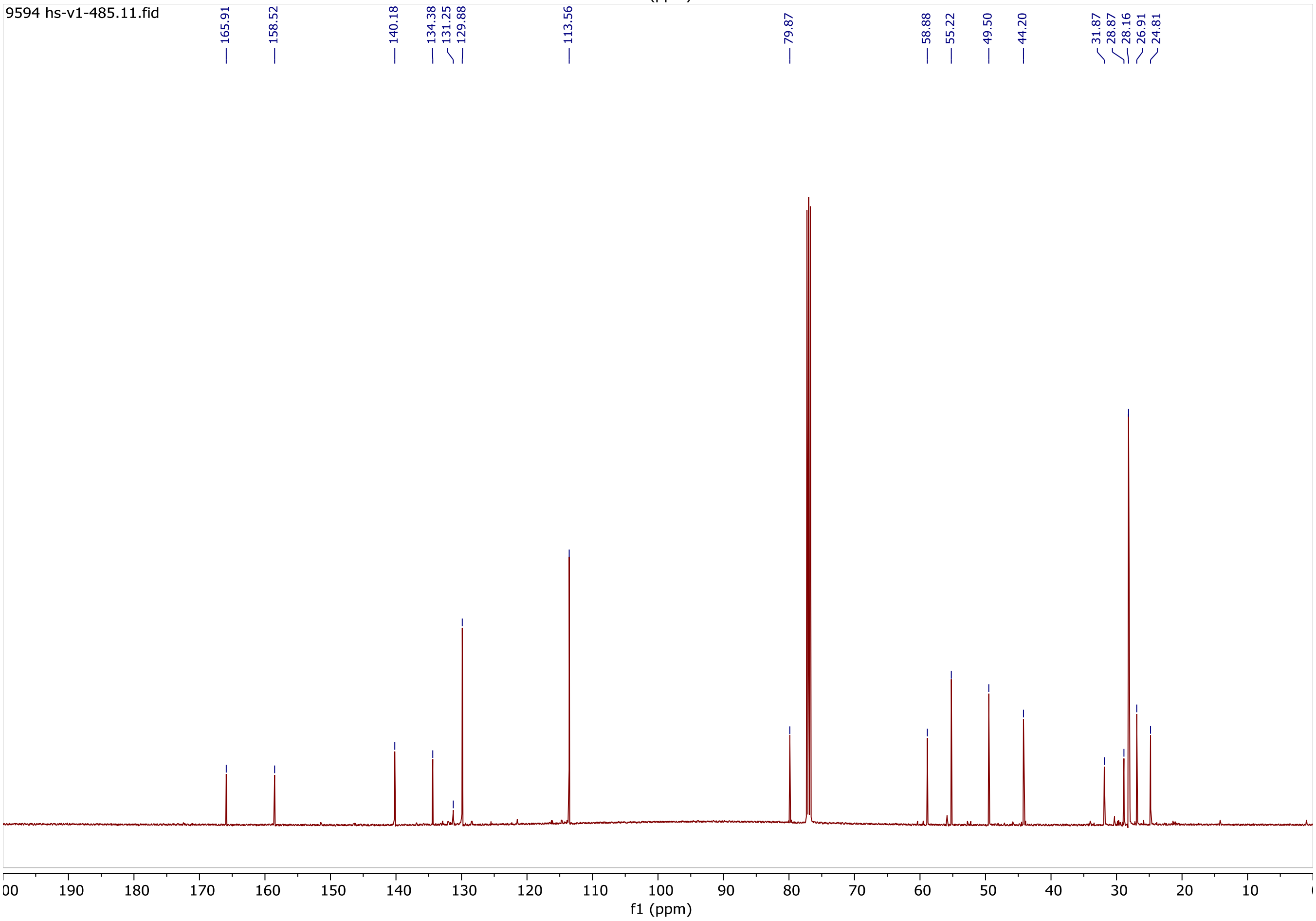

9594 hs-v1-485.11.fid
165.91 158.52 140.18 134.38 131.25 129.88 113.56 79.87 58.88 55.22 49.50 44.20 31.87 28.87 28.16 26.91 24.81
f1 (ppm)

CDCl$_3$

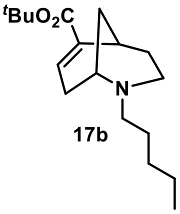

^{t}BuO$_2$C
N

17b

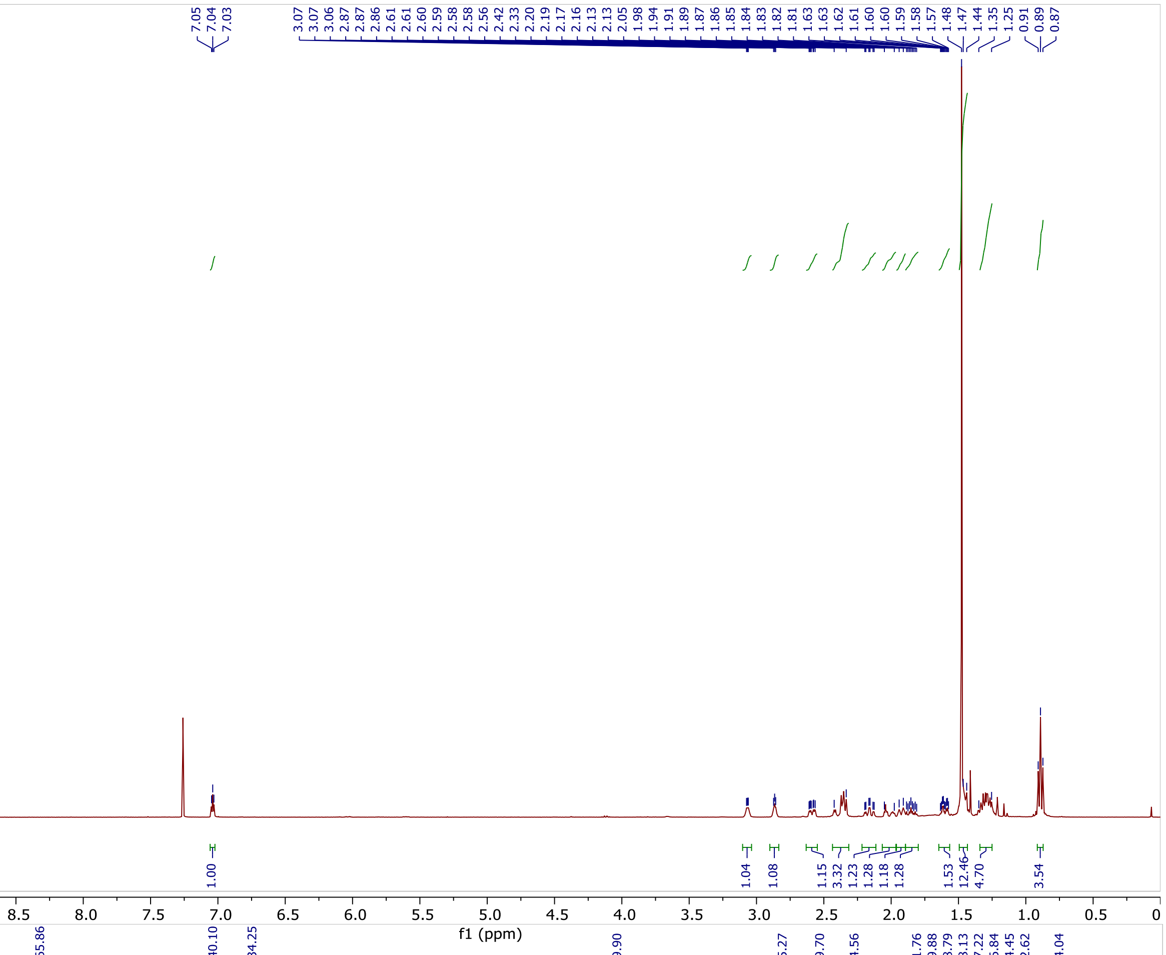

57600 HS-X-766.10.fid
7.05
7.04
7.03
3.07
3.07
3.06
2.87
2.87
2.86
2.61
2.61
2.60
2.59
2.58
2.58
2.56
2.42
2.33
2.20
2.19
2.17
2.16
2.13
2.13
2.05
1.98
1.94
1.91
1.89
1.87
1.86
1.85
1.84
1.83
1.82
1.81
1.63
1.63
1.62
1.61
1.60
1.60
1.59
1.58
1.57
1.48
1.47
1.44
1.35
1.25
0.91
0.89
0.87
1.00
1.04
1.08
1.15
3.32
1.23
1.28
1.18
1.28
1.53
12.46
4.70
3.54
0.0 9.5 9.0 8.5 8.0 7.5 7.0 6.5 6.0 5.5 5.0 4.5 4.0 3.5 3.0 2.5 2.0 1.5 1.0 0.5 0
f1 (ppm)

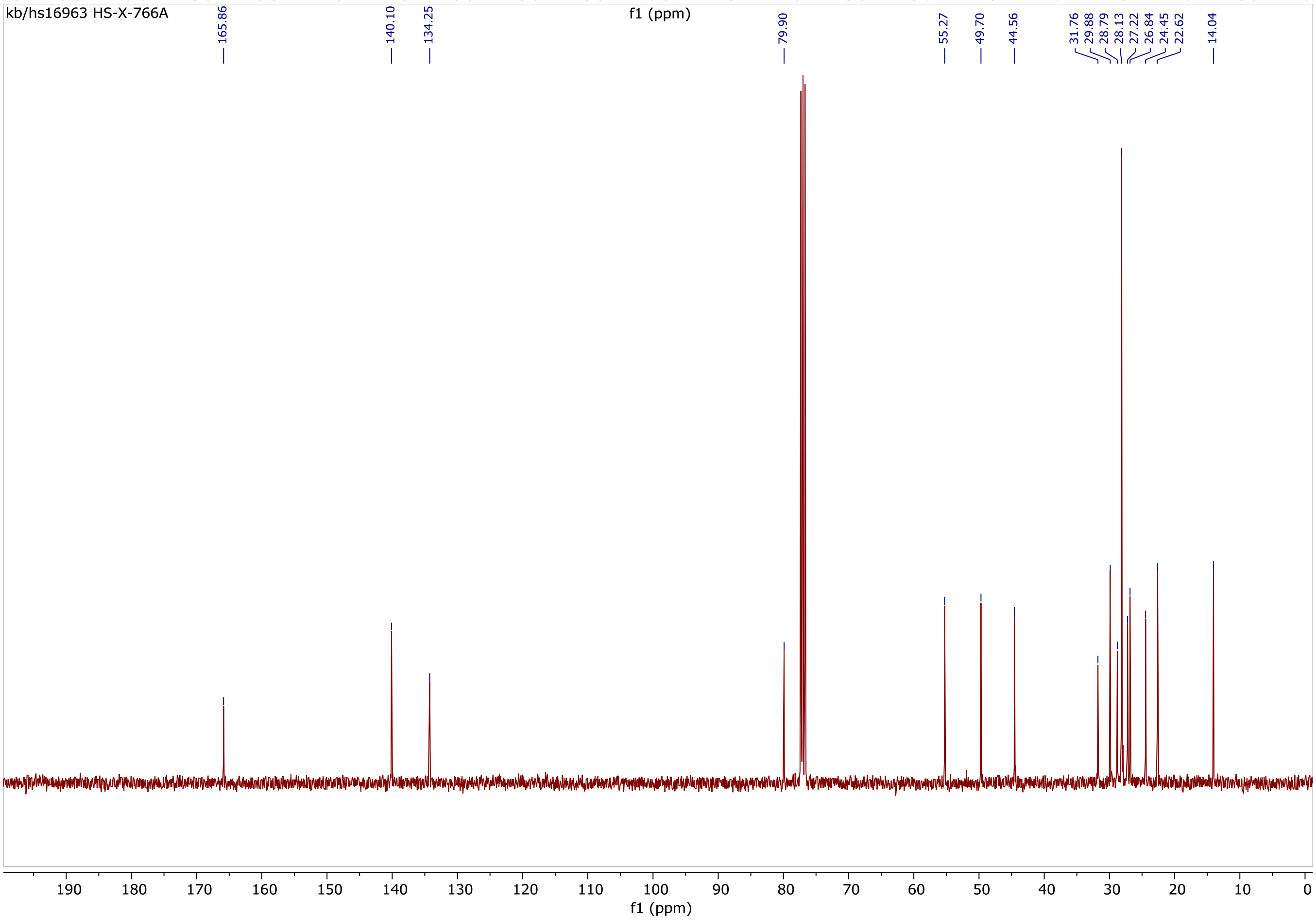

kb/hs16963 HS-X-766A
165.86
140.10
134.25
79.90
55.27
49.70
44.56
31.76
29.88
28.79
28.13
27.22
26.84
24.45
22.62
14.04
190 180 170 160 150 140 130 120 110 100 90 80 70 60 50 40 30 20 10 0
f1 (ppm)

CDCl$_3$

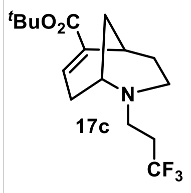

^{t}BuO$_2$C
N
CF$_3$

17c

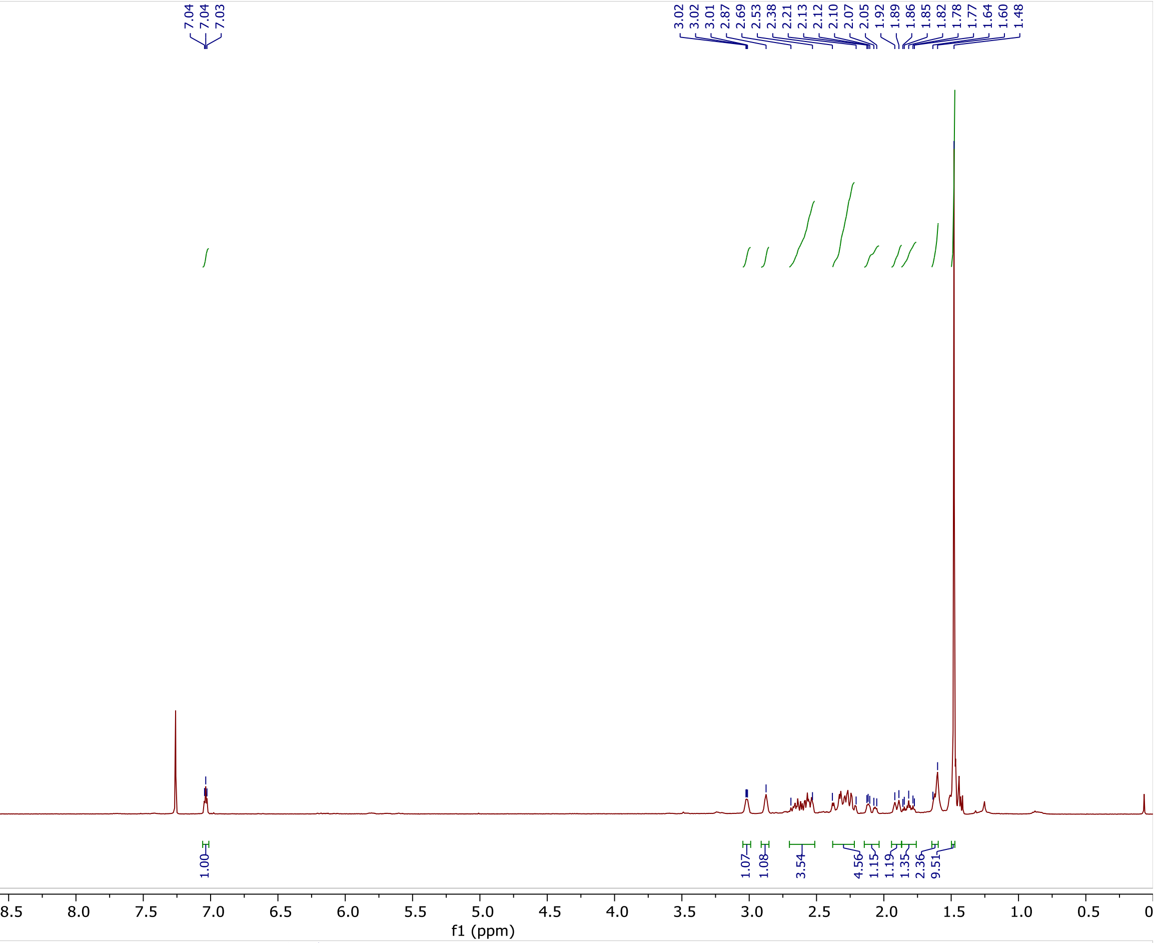

kb/hs16963 HS-X-765
7.04
7.04
7.03
3.02
3.02
3.01
2.87
2.69
2.53
2.38
2.21
2.13
2.12
2.10
2.07
2.05
1.92
1.89
1.86
1.85
1.82
1.78
1.77
1.64
1.60
1.48
1.00
1.07
1.08
3.54
4.56
1.15
1.19
1.35
2.36
9.51
f1 (ppm)

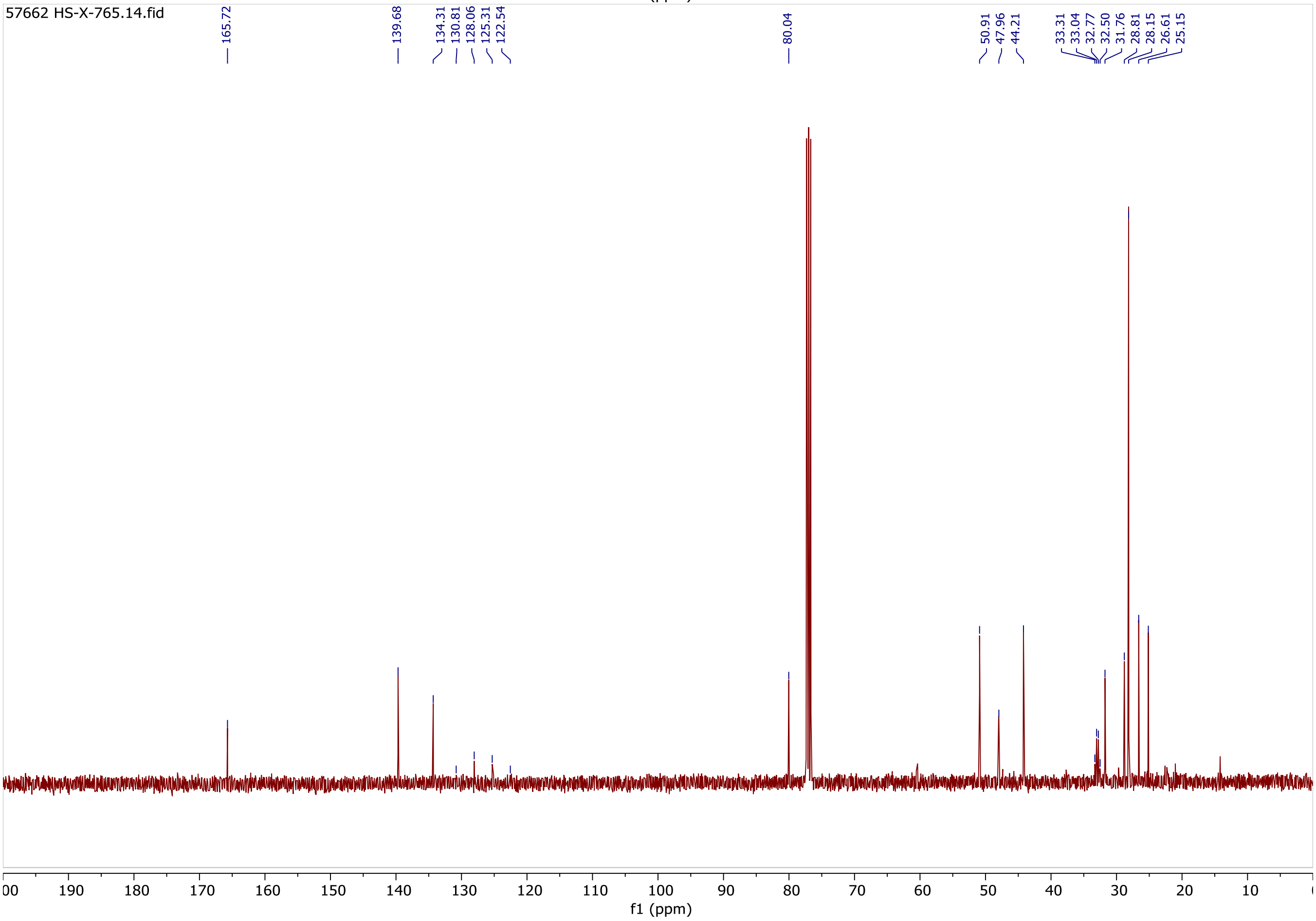

57662 HS-X-765.14.fid
165.72
139.68
134.31
130.81
128.06
125.31
122.54
80.04
50.91
47.96
44.21
33.31
33.04
32.77
32.50
31.76
28.81
28.15
26.61
25.15
f1 (ppm)

CDCl$_3$

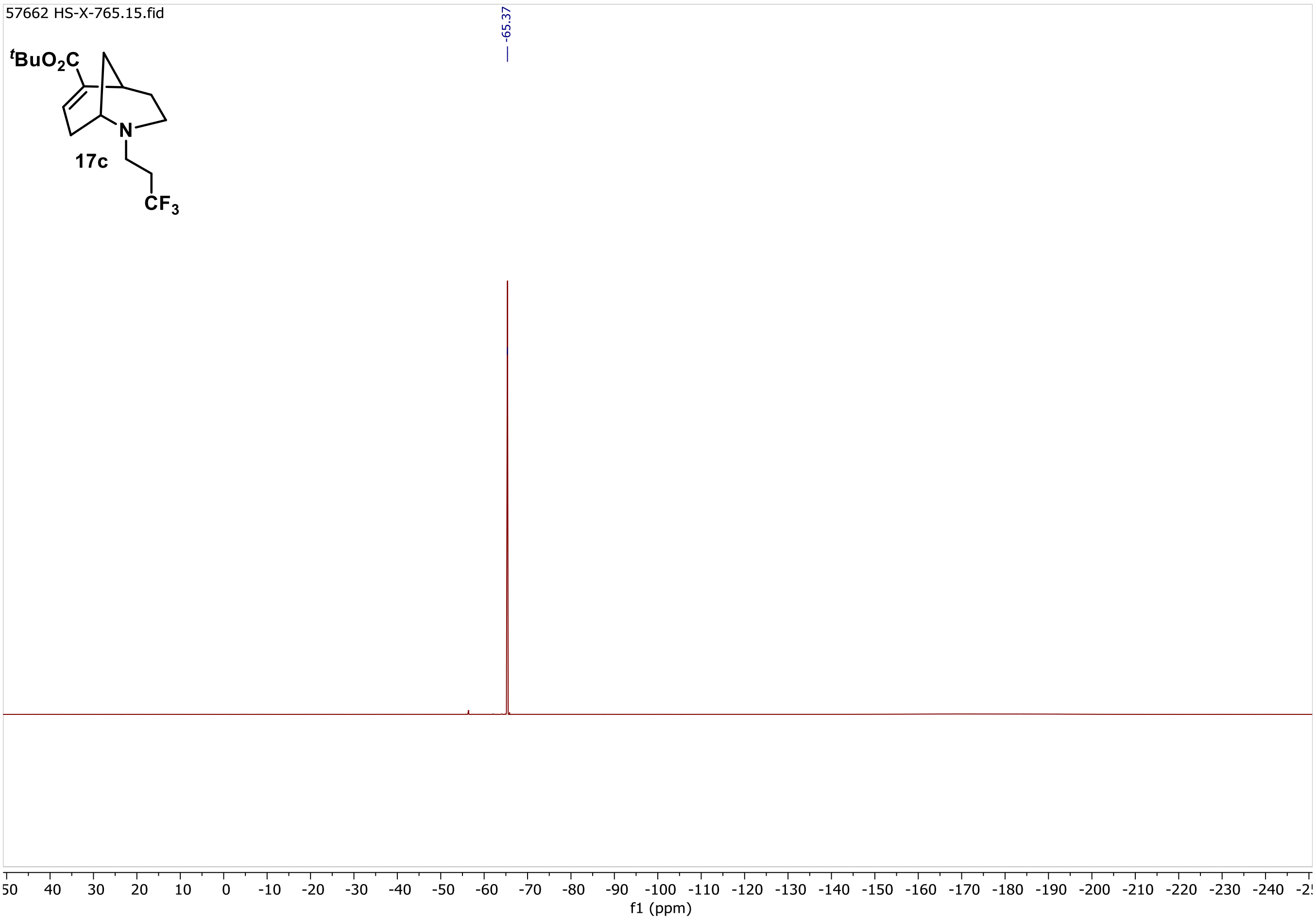

57662 HS-X-765.15.fid
-65.37
^{t}BuO$_2$C
N
17c
CF$_3$
50 40 30 20 10 0 -10 -20 -30 -40 -50 -60 -70 -80 -90 -100 -110 -120 -130 -140 -150 -160 -170 -180 -190 -200 -210 -220 -230 -240
f1 (ppm)

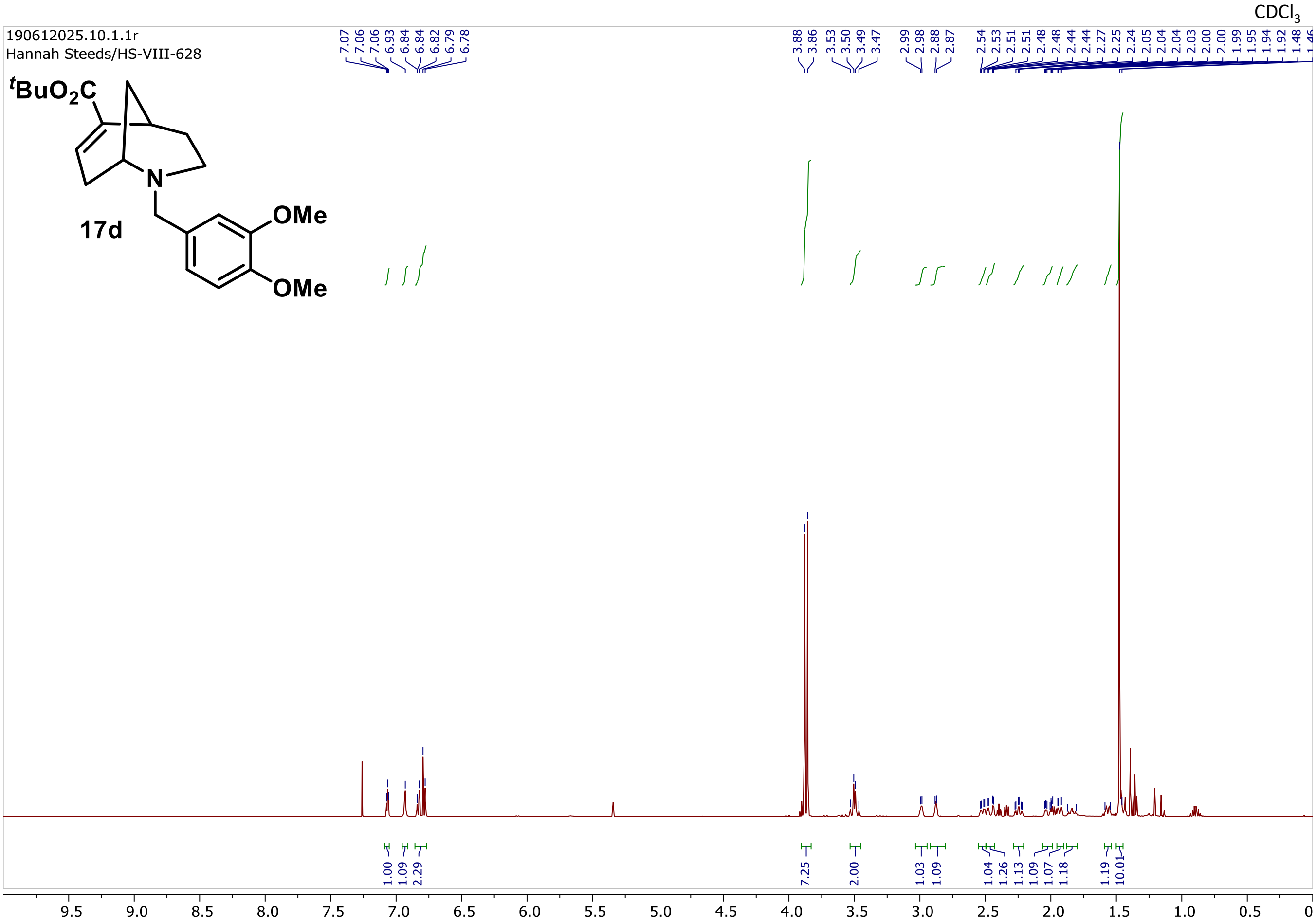
CDCl3
190612025.10.1.1r
Hannah Steeds/HS-VIII-628
tBuO2C
N
OMe
OMe
17d
7.07
7.06
7.06
6.93
6.84
6.84
6.82
6.79
6.78
3.88
3.86
3.53
3.50
3.49
3.47
2.99
2.98
2.88
2.87
2.54
2.53
2.51
2.51
2.48
2.48
2.44
2.44
2.27
2.25
2.24
2.05
2.04
2.04
2.03
2.00
2.00
1.99
1.95
1.94
1.92
1.48
1.00
1.09
2.29
7.25
2.00
1.03
1.09
1.04
1.26
1.13
1.09
1.07
1.18
1.19
10.01
9.5 9.0 8.5 8.0 7.5 7.0 6.5 6.0 5.5 5.0 4.5 4.0 3.5 3.0 2.5 2.0 1.5 1.0 0.5
f1 (ppm)
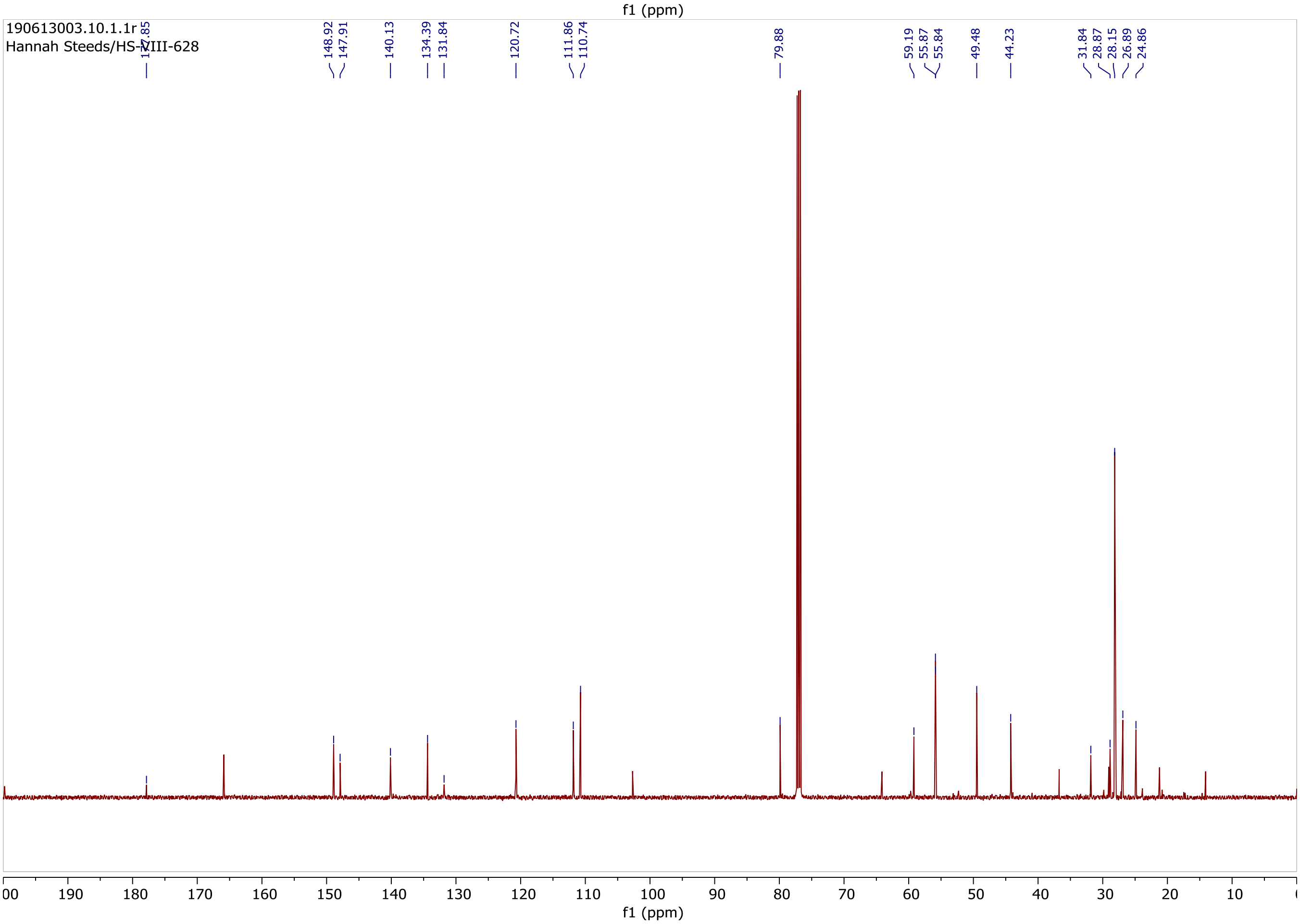
190613003.10.1.1r
Hannah Steeds/HS-VIII-628
148.92
147.91
140.13
134.39
131.84
120.72
111.86
110.74
79.88
59.19
55.87
55.84
49.48
44.23
31.84
28.87
28.15
26.89
24.86
190 180 170 160 150 140 130 120 110 100 90 80 70 60 50 40 30 20 10
f1 (ppm)

CDCl$_3$

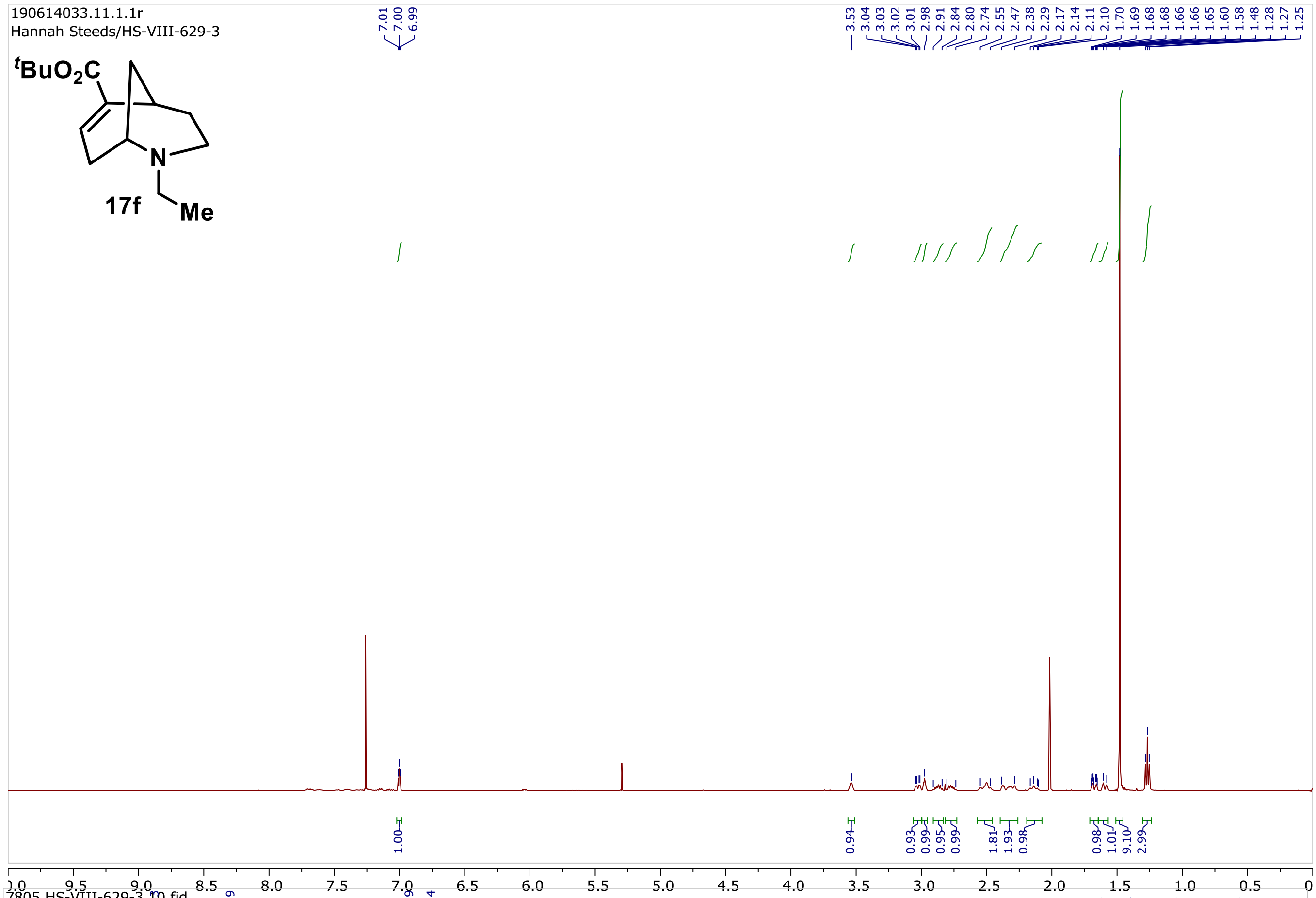
190614033.11.1.1r
Hannah Steeds/HS-VIII-629-3
^{t}BuO$_2$C
N
Me
17f
7.01
7.00
6.99
3.53
3.04
3.03
3.02
3.01
2.98
2.91
2.84
2.80
2.74
2.55
2.47
2.38
2.29
2.17
2.14
2.11
2.10
1.70
1.69
1.68
1.68
1.66
1.66
1.65
1.60
1.58
1.48
1.28
1.27
1.25
1.00
0.94
0.93
0.99
0.95
0.99
1.81
1.93
0.98
0.98
1.01
9.10
2.99
f1 (ppm)

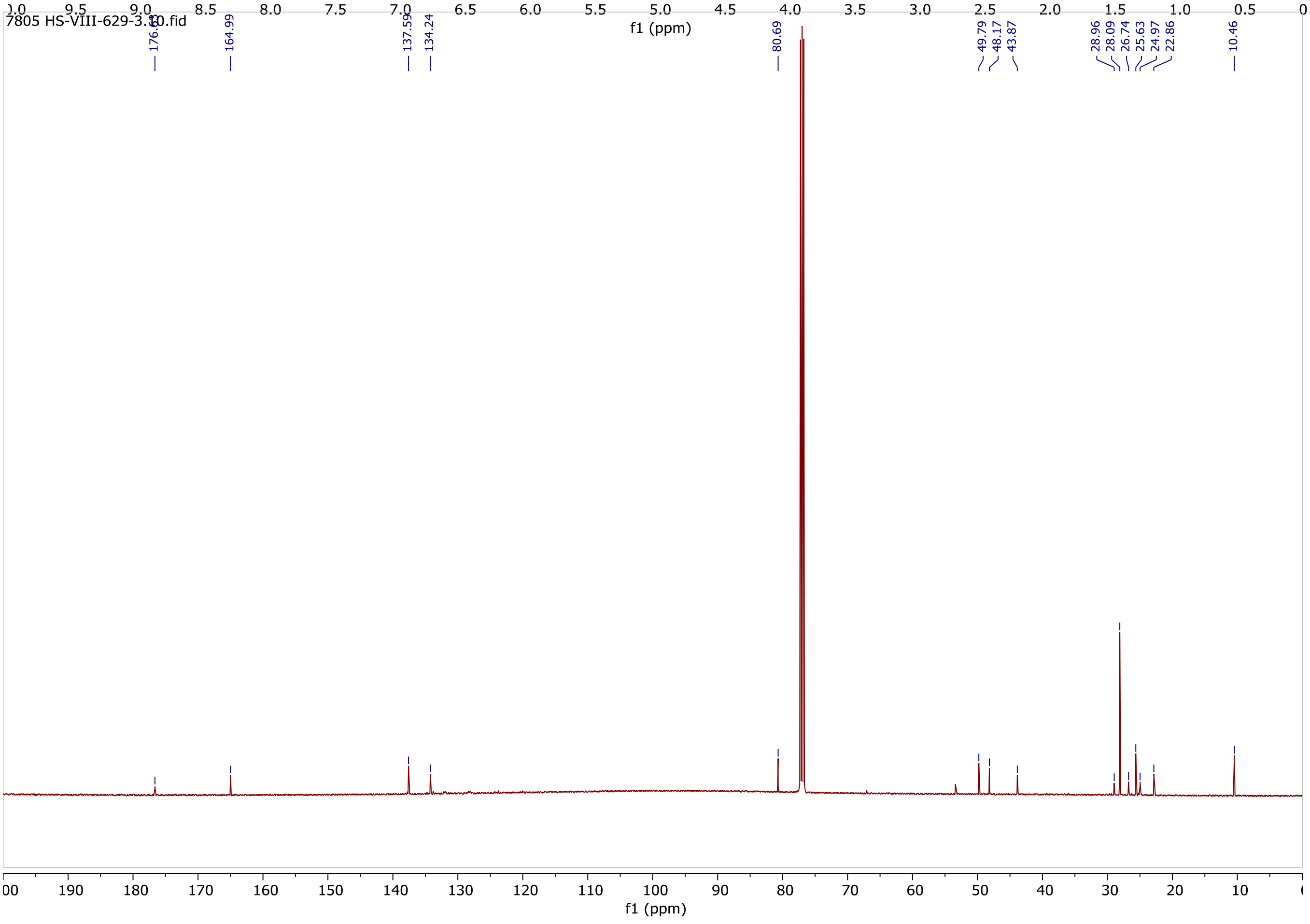
HS-VIII-629-3.10.fid
164.99
137.59
134.24
80.69
49.79
48.17
43.87
28.96
28.09
26.74
25.63
24.97
22.86
10.46
f1 (ppm)

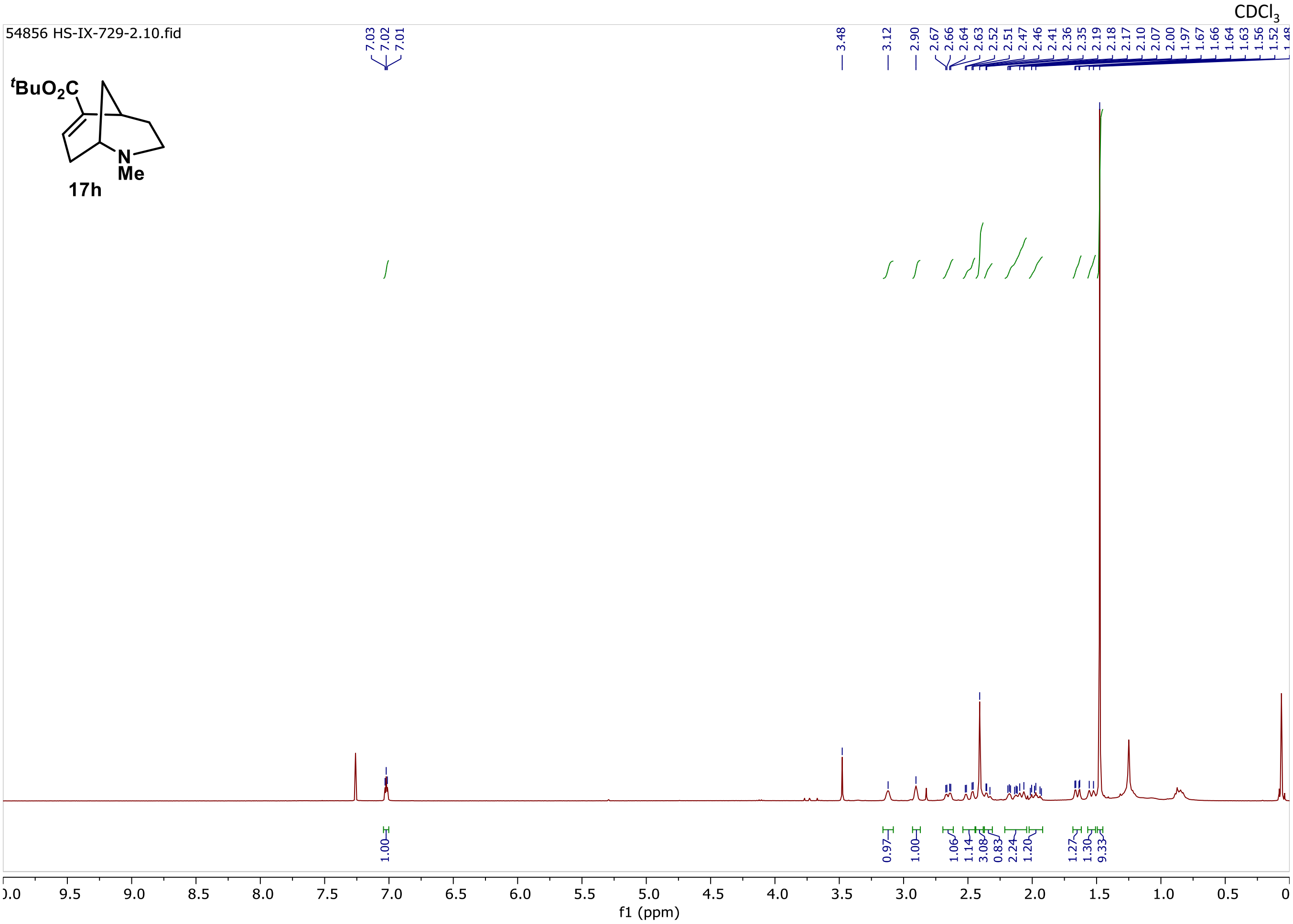
CDCl$_3$
54856 HS-IX-729-2.10.fid
^{t}BuO$_2$C
N
Me
17h
7.03
7.02
7.01
3.48
3.12
2.90
2.67
2.66
2.64
2.63
2.52
2.51
2.47
2.46
2.41
2.36
2.35
2.19
2.18
2.17
2.10
2.07
2.00
1.97
1.67
1.66
1.64
1.63
1.56
1.52
1.00
0.97
1.00
1.06
1.14
3.08
0.83
2.24
1.20
1.27
1.30
9.33
9.5
9.0
8.5
8.0
7.5
7.0
6.5
6.0
5.5
5.0
4.5
4.0
3.5
3.0
2.5
2.0
1.5
1.0
0.5
f1 (ppm)

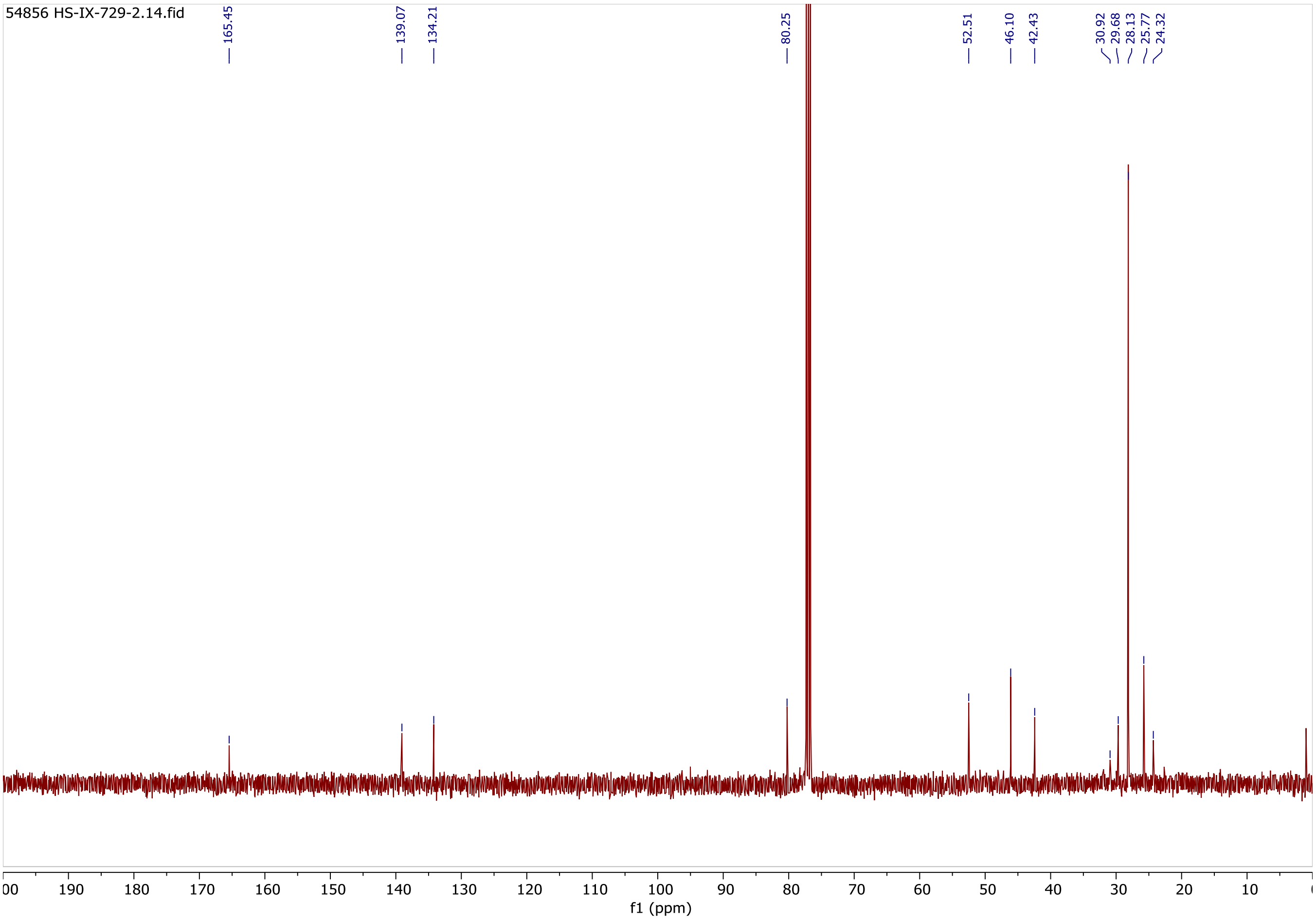
54856 HS-IX-729-2.14.fid
165.45
139.07
134.21
80.25
52.51
46.10
42.43
30.92
29.68
28.13
25.77
24.32
190
180
170
160
150
140
130
120
110
100
90
80
70
60
50
40
30
20
10
f1 (ppm)

CDCl3

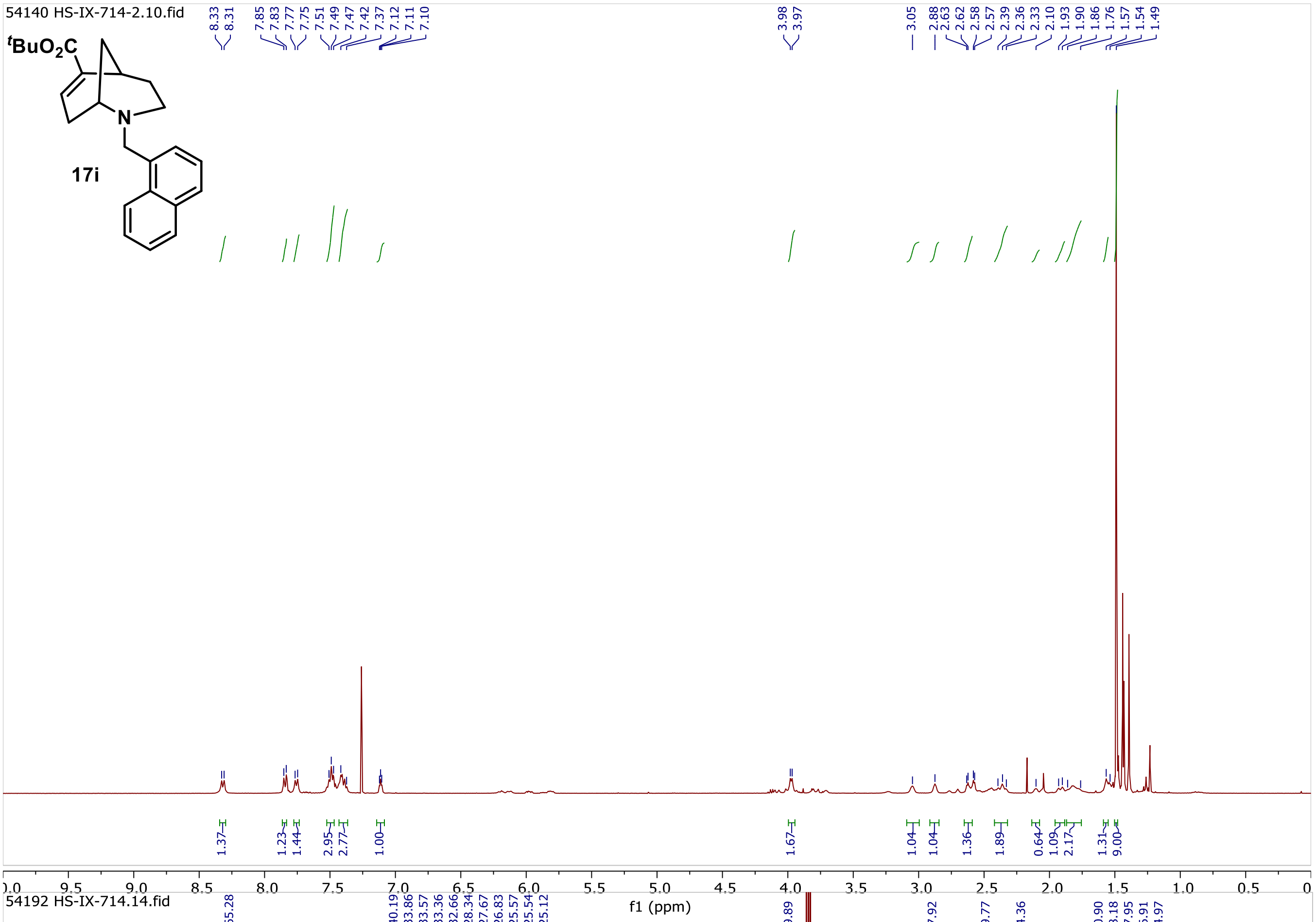
54140 HS-IX-714-2.10.fid
tBuO2C
N
17i
8.33 8.31 7.85 7.83 7.77 7.75 7.51 7.49 7.47 7.42 7.37 7.12 7.11 7.10
3.98 3.97
3.05 2.88 2.63 2.62 2.58 2.57 2.39 2.36 2.33 2.10 1.93 1.90 1.86 1.76 1.57 1.54 1.49
1.37 1.23 1.44 2.95 2.77 1.00 1.67 1.04 1.04 1.36 1.89 0.64 1.09 2.17 1.31 9.00
f1 (ppm)

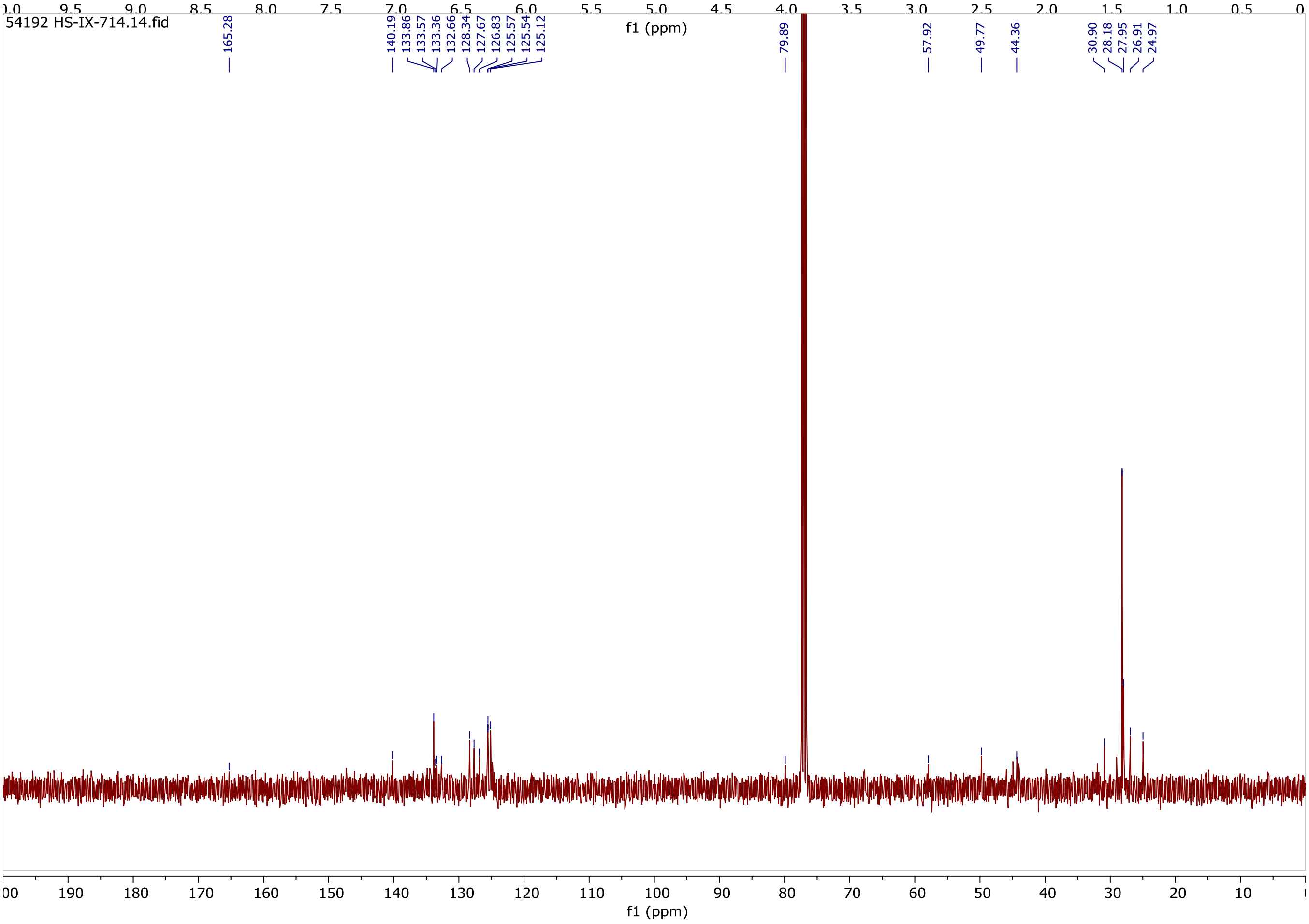
54192 HS-IX-714.14.fid
165.28
140.19 133.86 133.57 133.36 132.66 128.34 127.67 126.83 125.57 125.54 125.12
79.89
57.92 49.77 44.36
30.90 28.18 27.95 26.91 24.97
f1 (ppm)

CDCl$_3$

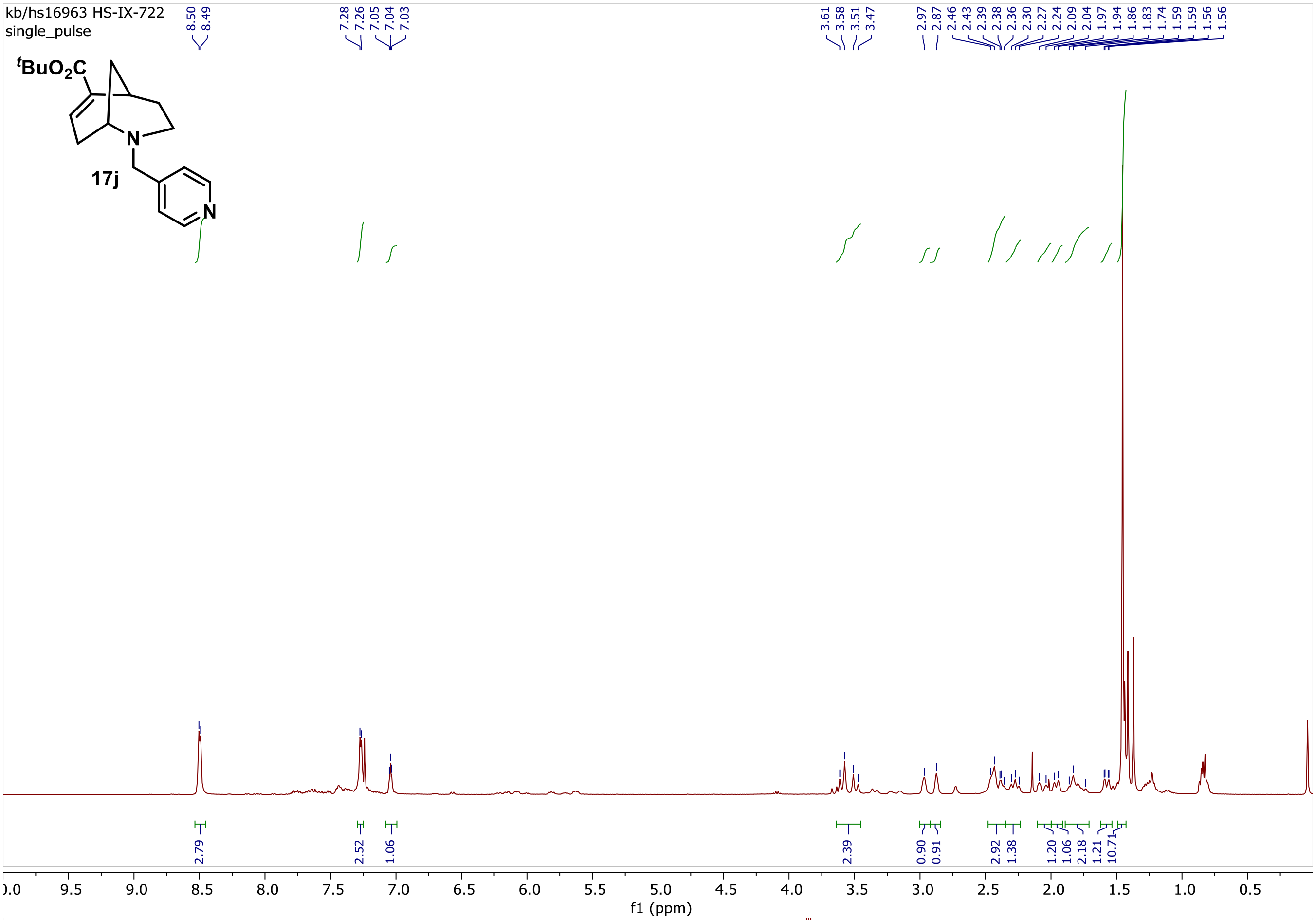
kb/hs16963 HS-IX-722
single_pulse
^{t}BuO$_2$C
N
17j
N
8.50 8.49
7.28 7.26 7.05 7.04 7.03
3.61 3.58 3.51 3.47
2.97 2.87 2.46 2.43 2.39 2.38 2.36 2.30 2.27 2.24 2.09 2.04 1.97 1.94 1.86 1.83 1.74 1.59 1.59 1.56 1.56
2.79
2.52
1.06
2.39
0.90 0.91
2.92 1.38
1.20 1.06 2.18 1.21 10.71
f1 (ppm)

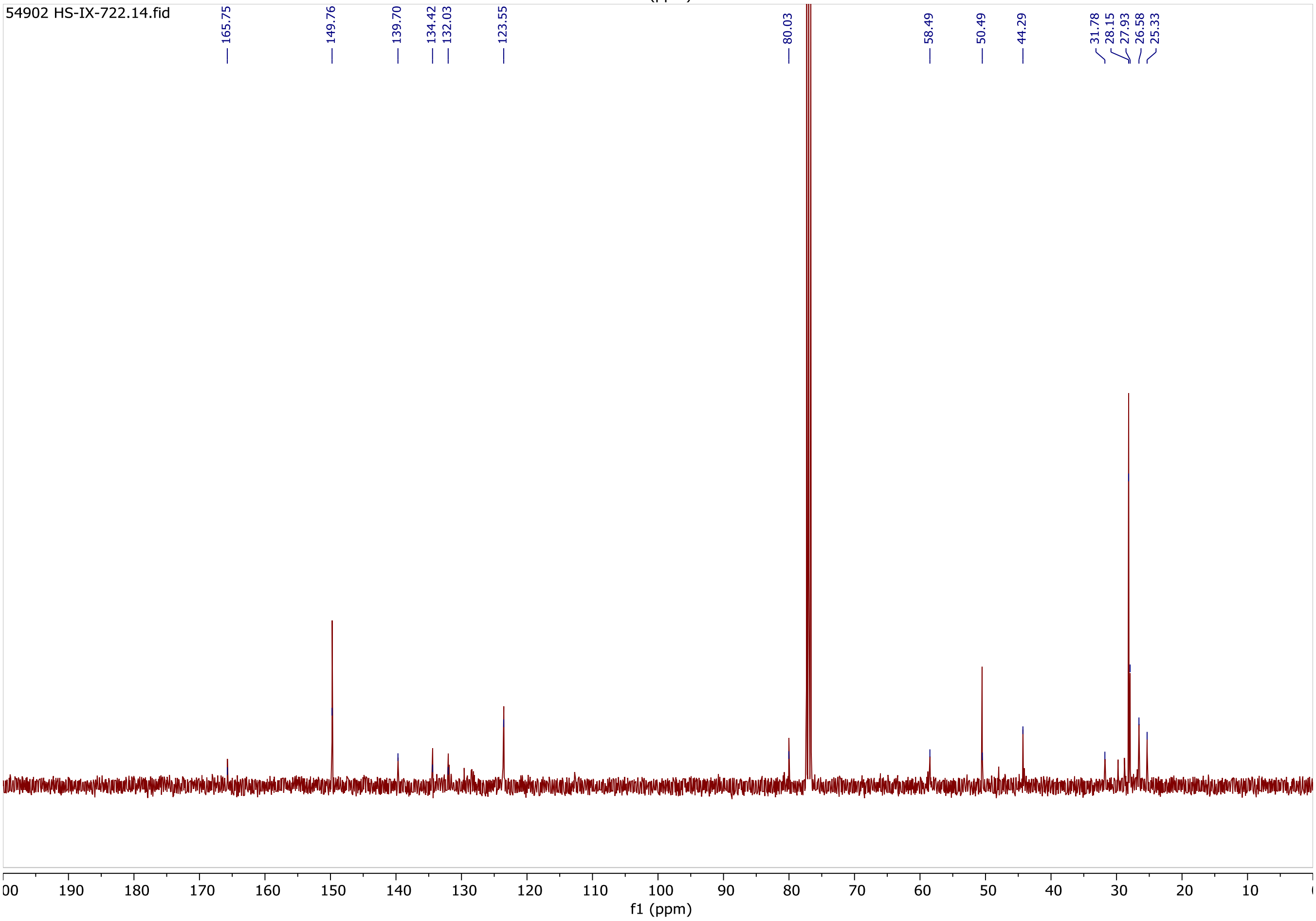
54902 HS-IX-722.14.fid
165.75
149.76
139.70 134.42 132.03
123.55
80.03
58.49
50.49
44.29
31.78 28.15 27.93 26.58 25.33
f1 (ppm)

CDCl$_3$

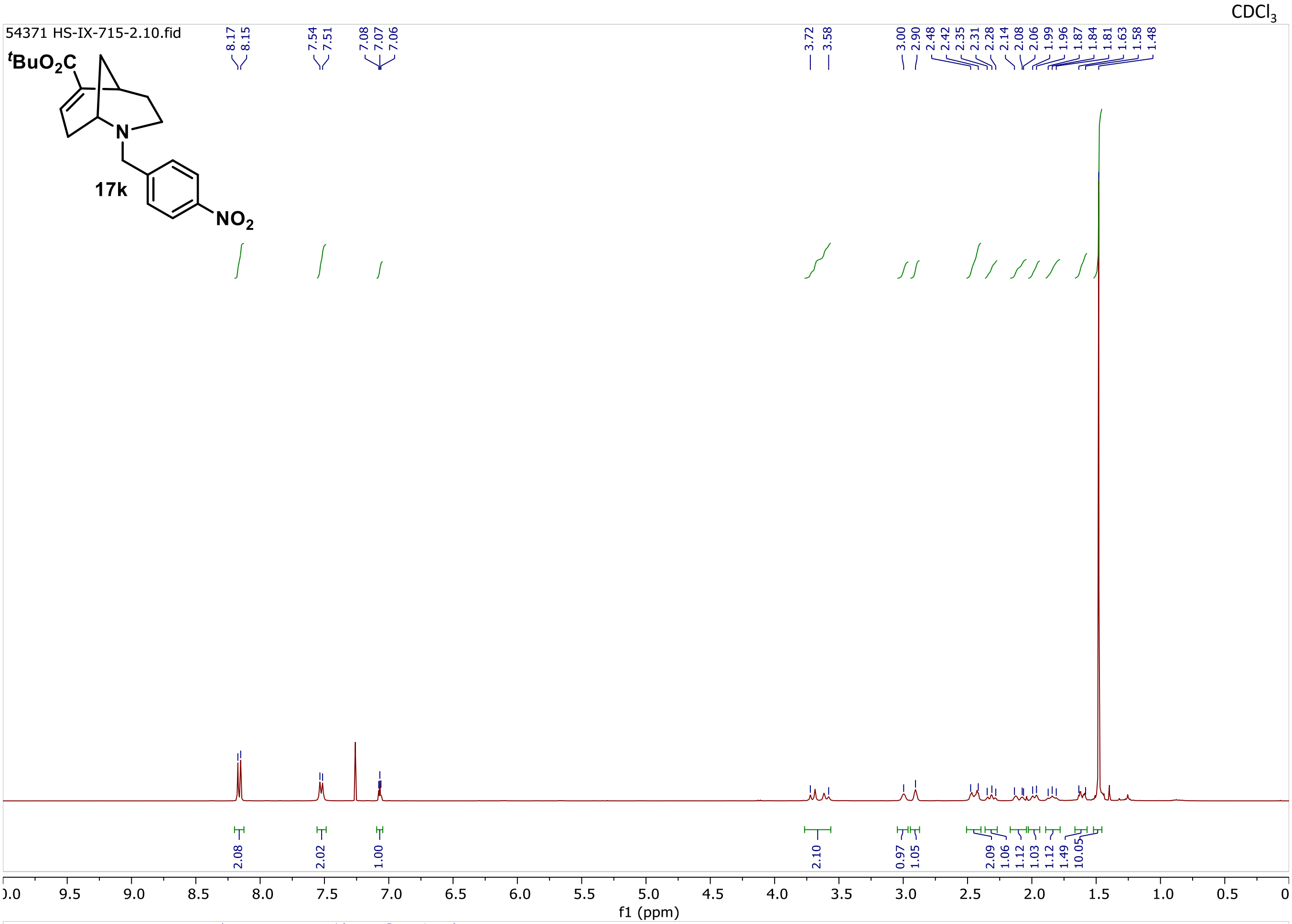
54371 HS-IX-715-2.10.fid
8.17
8.15
7.54
7.51
7.08
7.07
7.06
3.72
3.58
3.00
2.90
2.48
2.42
2.35
2.31
2.28
2.14
2.08
2.06
1.99
1.96
1.87
1.84
1.81
1.63
1.58
1.48
tBuO2C
N
17k
NO2
2.08
2.02
1.00
2.10
0.97
1.05
2.09
1.06
1.12
1.03
1.12
1.49
10.05
f1 (ppm)

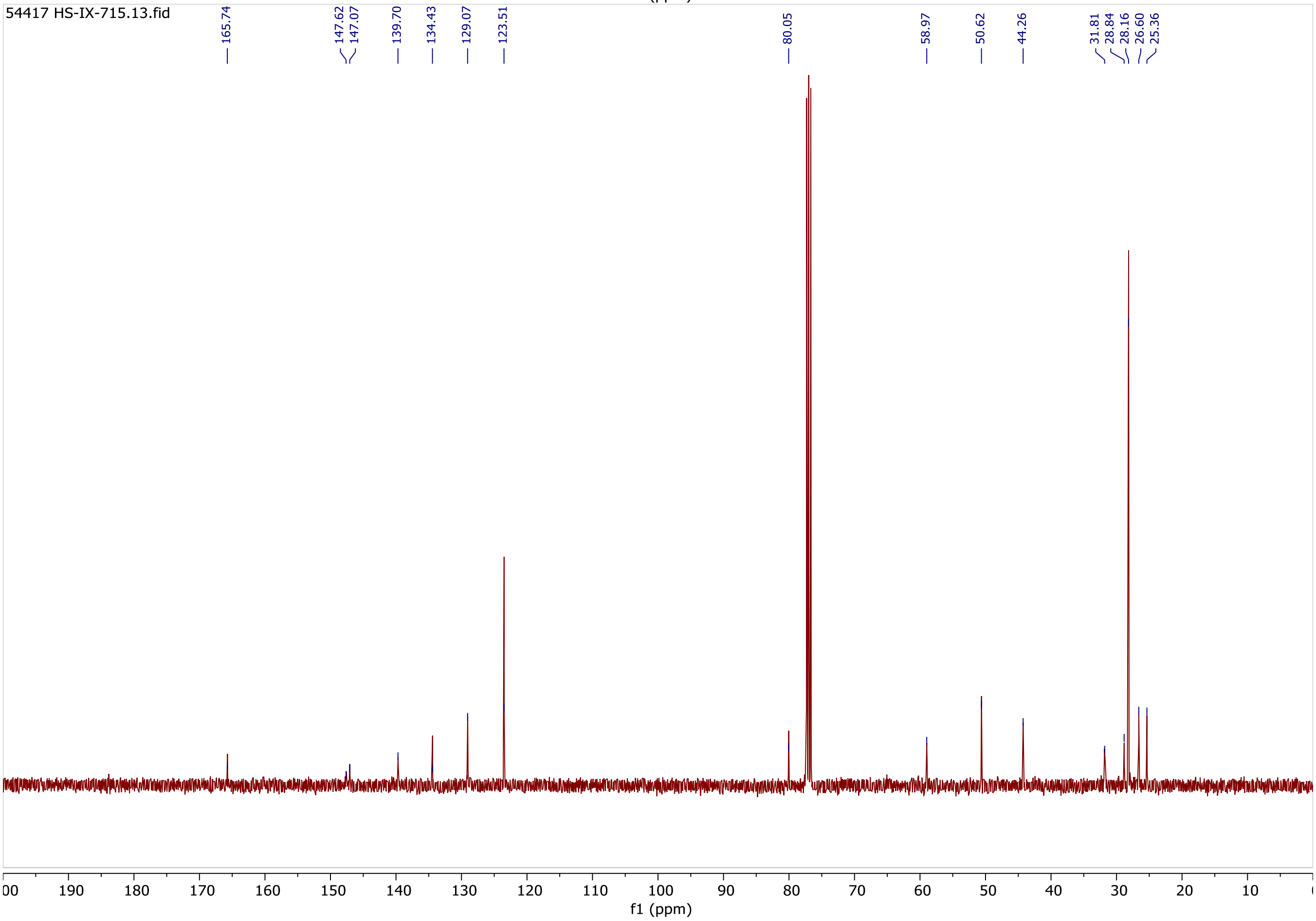
54417 HS-IX-715.13.fid
165.74
147.62
147.07
139.70
134.43
129.07
123.51
80.05
58.97
50.62
44.26
31.81
28.84
28.16
26.60
25.36
f1 (ppm)

CDCl3

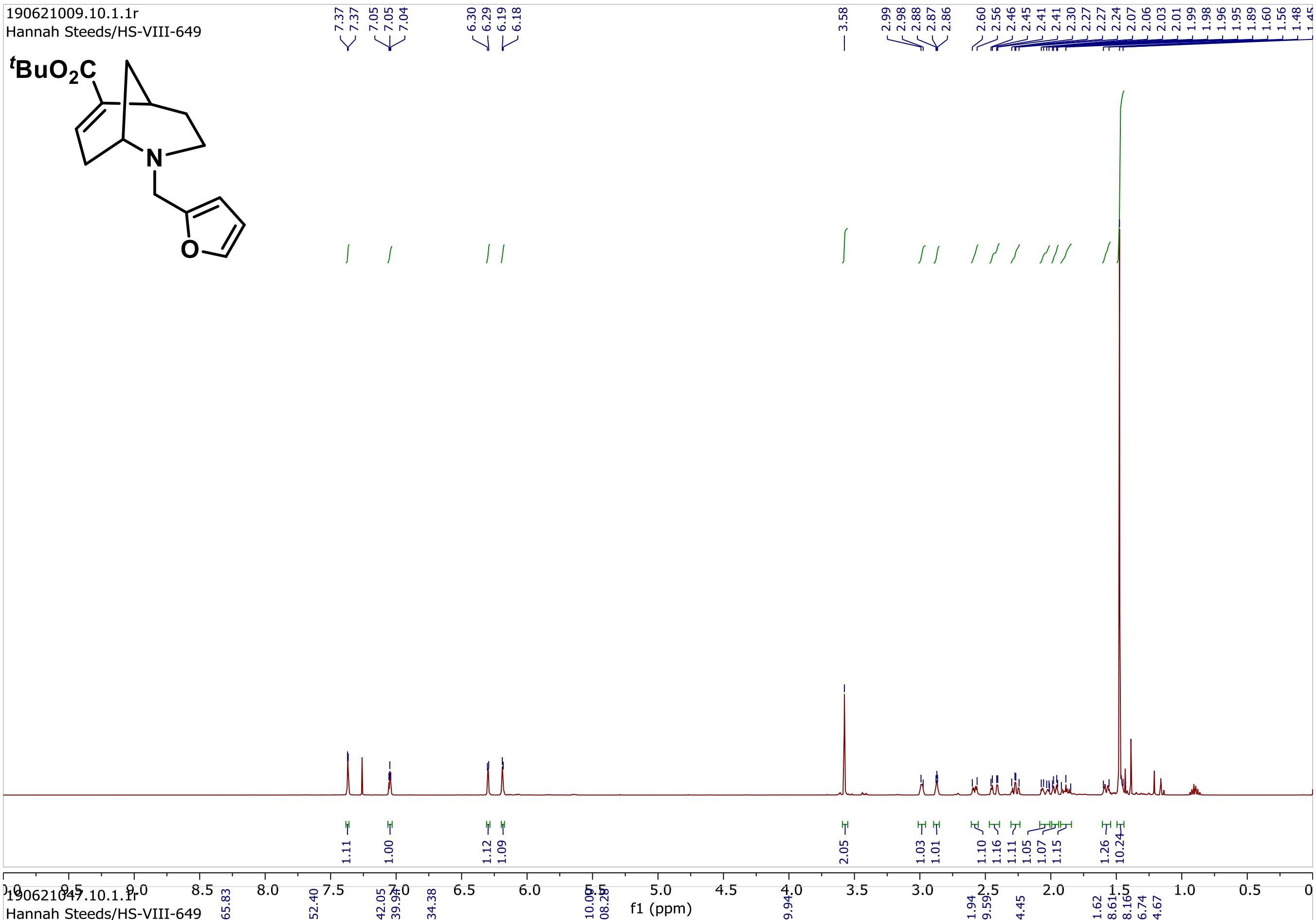

190621009.10.1.1r
Hannah Steeds/HS-VIII-649
tBuO2C
N
O
7.37
7.37
7.05
7.05
7.04
6.30
6.29
6.19
6.18
3.58
2.99
2.98
2.88
2.87
2.86
2.60
2.56
2.46
2.45
2.41
2.41
2.30
2.27
2.27
2.24
2.07
2.06
2.03
2.01
1.99
1.98
1.96
1.95
1.89
1.60
1.56
1.48
1.11
1.00
1.12
1.09
2.05
1.03
1.01
1.10
1.16
1.11
1.05
1.07
1.15
1.26
10.24
f1 (ppm)

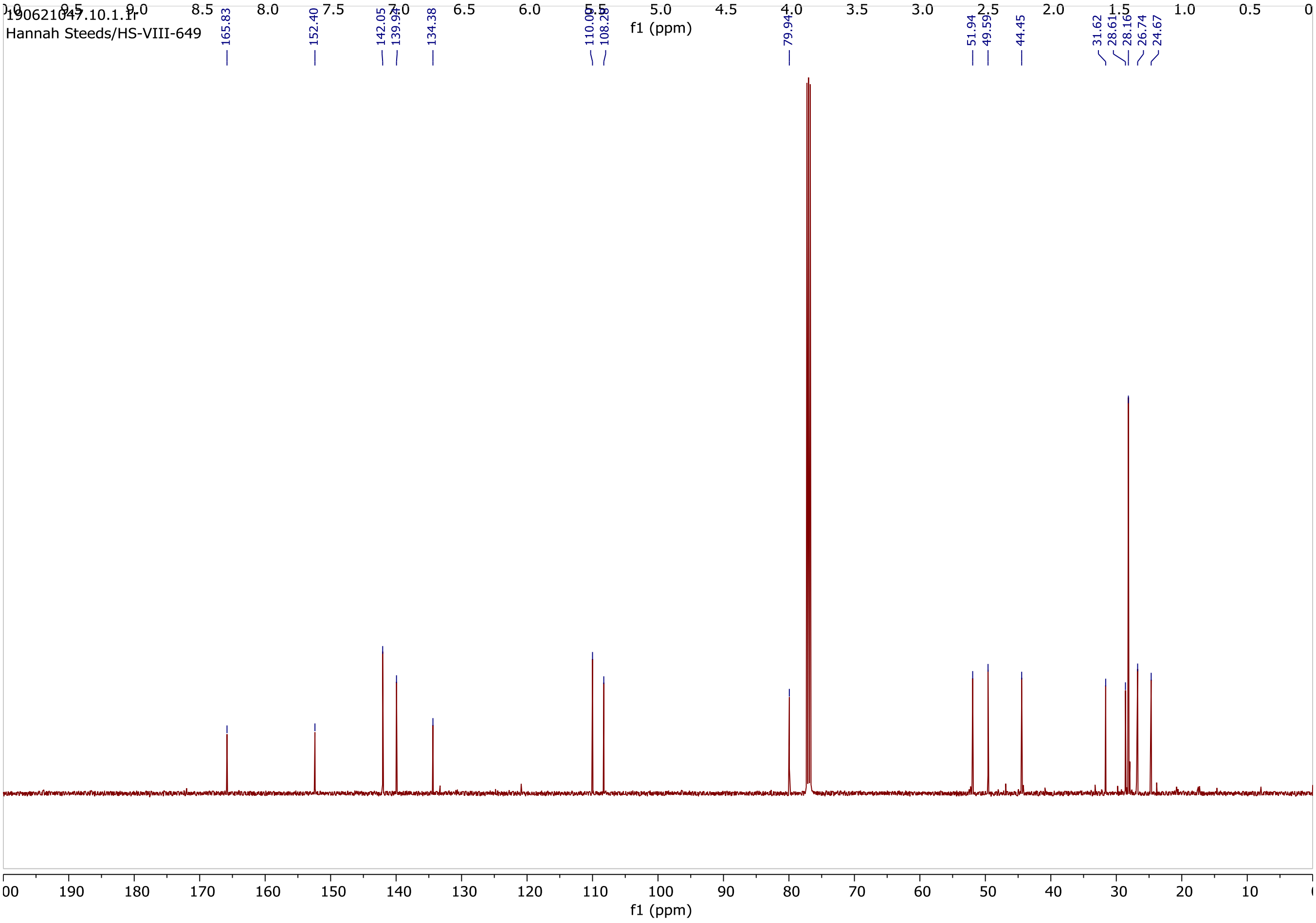

190621047.10.1.1r
Hannah Steeds/HS-VIII-649
165.83
152.40
142.05
139.94
134.38
110.09
108.28
79.94
51.94
49.59
44.45
31.62
28.61
28.16
26.74
24.67
f1 (ppm)

$CDCl_3$

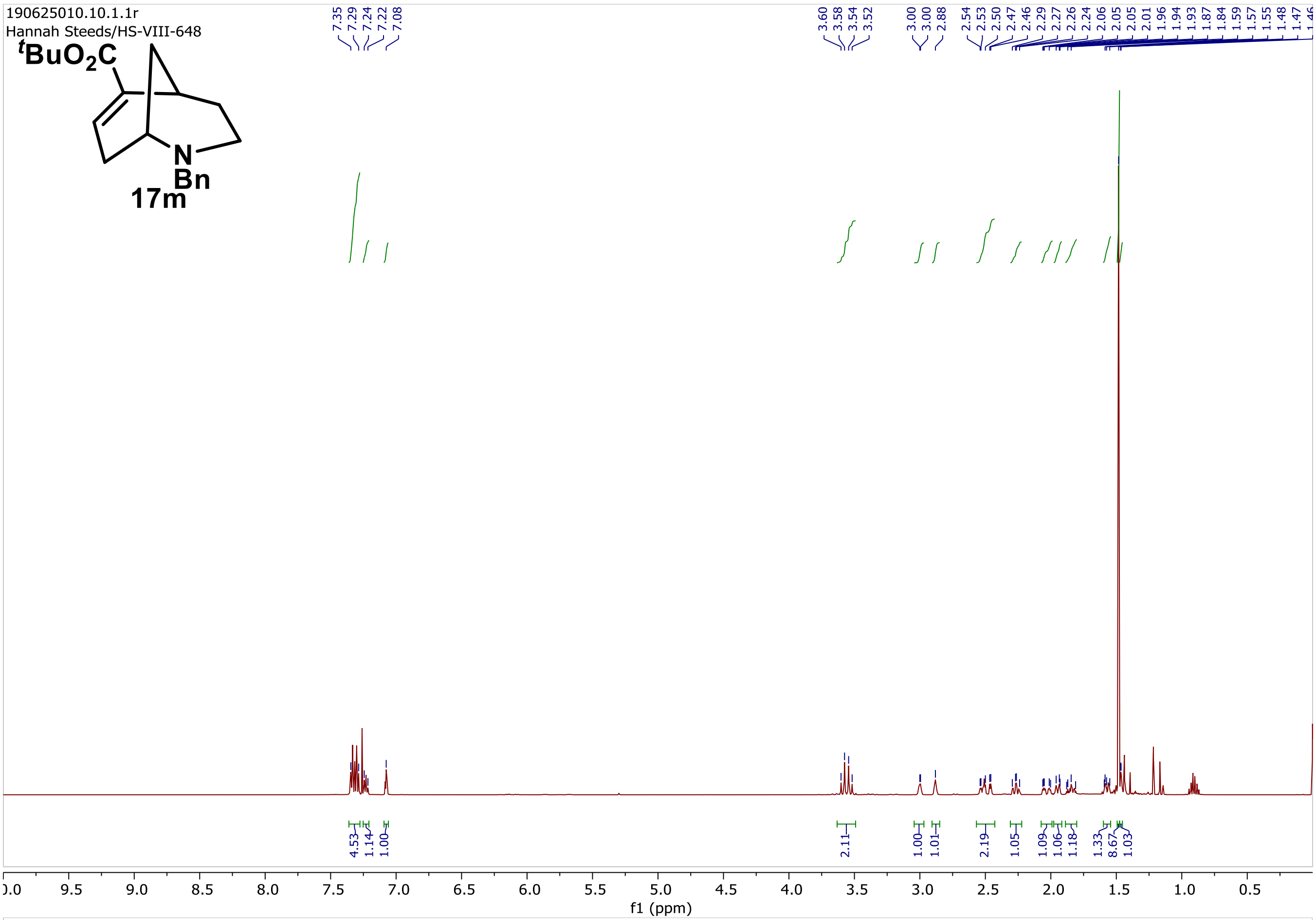
190625010.10.1.1r
Hannah Steeds/HS-VIII-648
$^{t}BuO_2C$
N
Bn
17m
7.35
7.29
7.24
7.22
7.08
3.60
3.58
3.54
3.52
3.00
3.00
2.88
2.54
2.53
2.50
2.47
2.46
2.29
2.27
2.26
2.24
2.06
2.05
2.05
2.01
1.96
1.94
1.93
1.87
1.84
1.59
1.57
1.55
1.48
1.47
4.53
1.14
1.00
2.11
1.00
1.01
2.19
1.05
1.09
1.06
1.18
1.33
8.67
1.03
f1 (ppm)

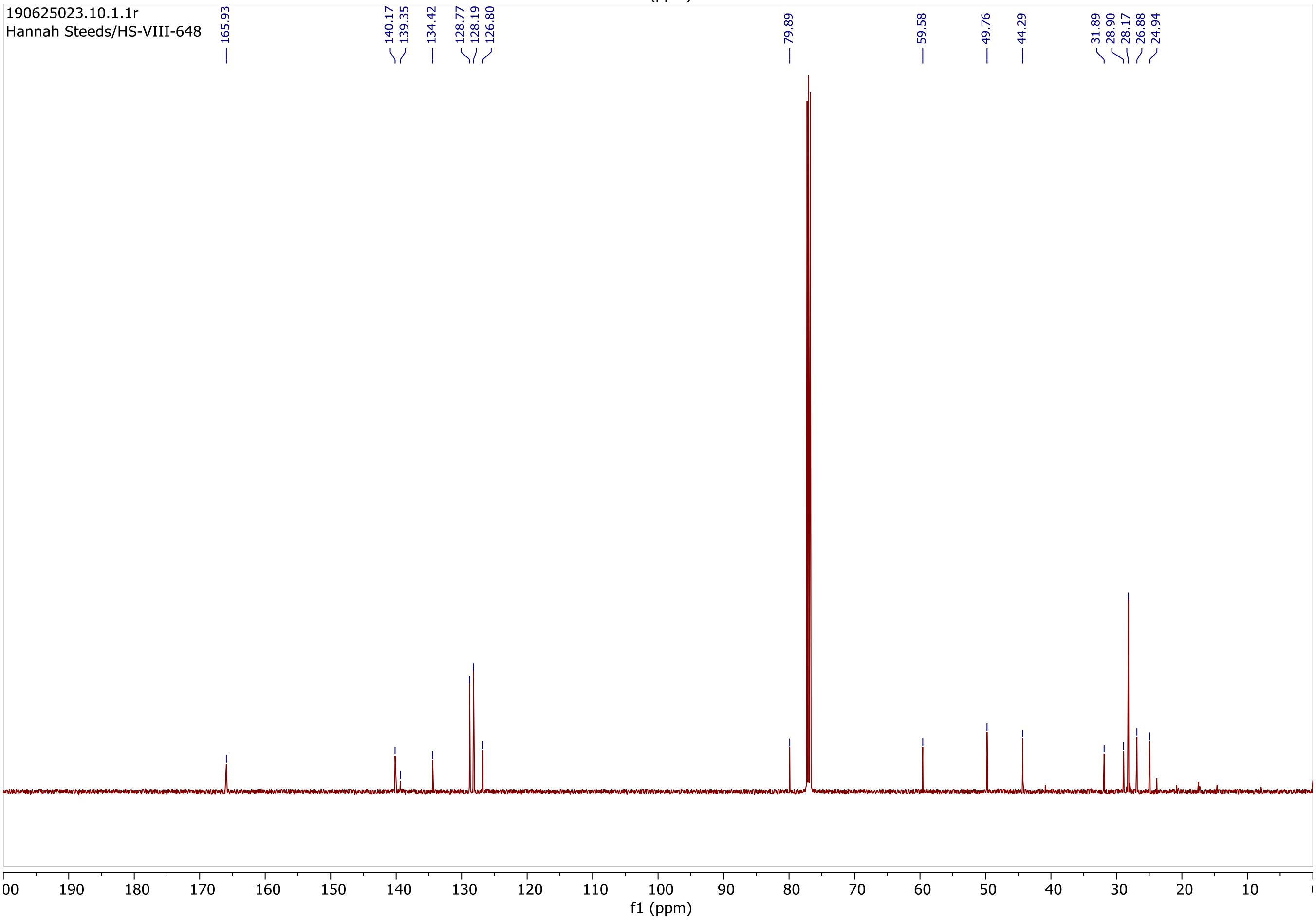
190625023.10.1.1r
Hannah Steeds/HS-VIII-648
165.93
140.17
139.35
134.42
128.77
128.19
126.80
79.89
59.58
49.76
44.29
31.89
28.90
28.17
26.88
24.94
f1 (ppm)

CDCl$_3$

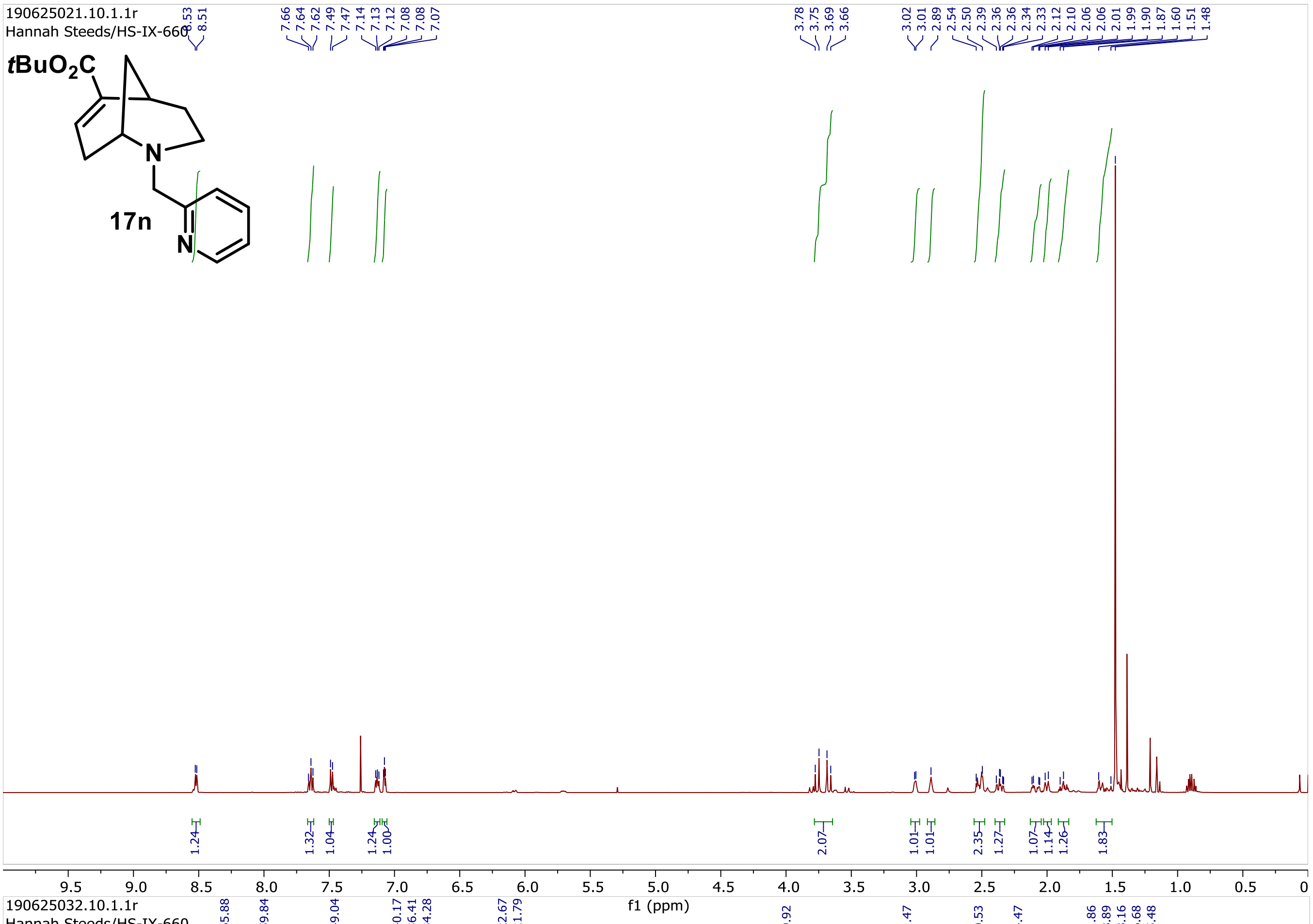

190625021.10.1.1r
Hannah Steeds/HS-IX-660
tBuO2C
N
N
17n
8.53
8.51
7.66
7.64
7.62
7.49
7.47
7.14
7.13
7.12
7.08
7.08
7.07
3.78
3.75
3.69
3.66
3.02
3.01
2.89
2.54
2.50
2.39
2.36
2.36
2.34
2.33
2.12
2.10
2.06
2.01
1.99
1.90
1.87
1.60
1.51
1.48
1.24
1.32
1.04
1.24
1.00
2.07
1.01
1.01
2.35
1.27
1.07
1.14
1.26
1.83
f1 (ppm)

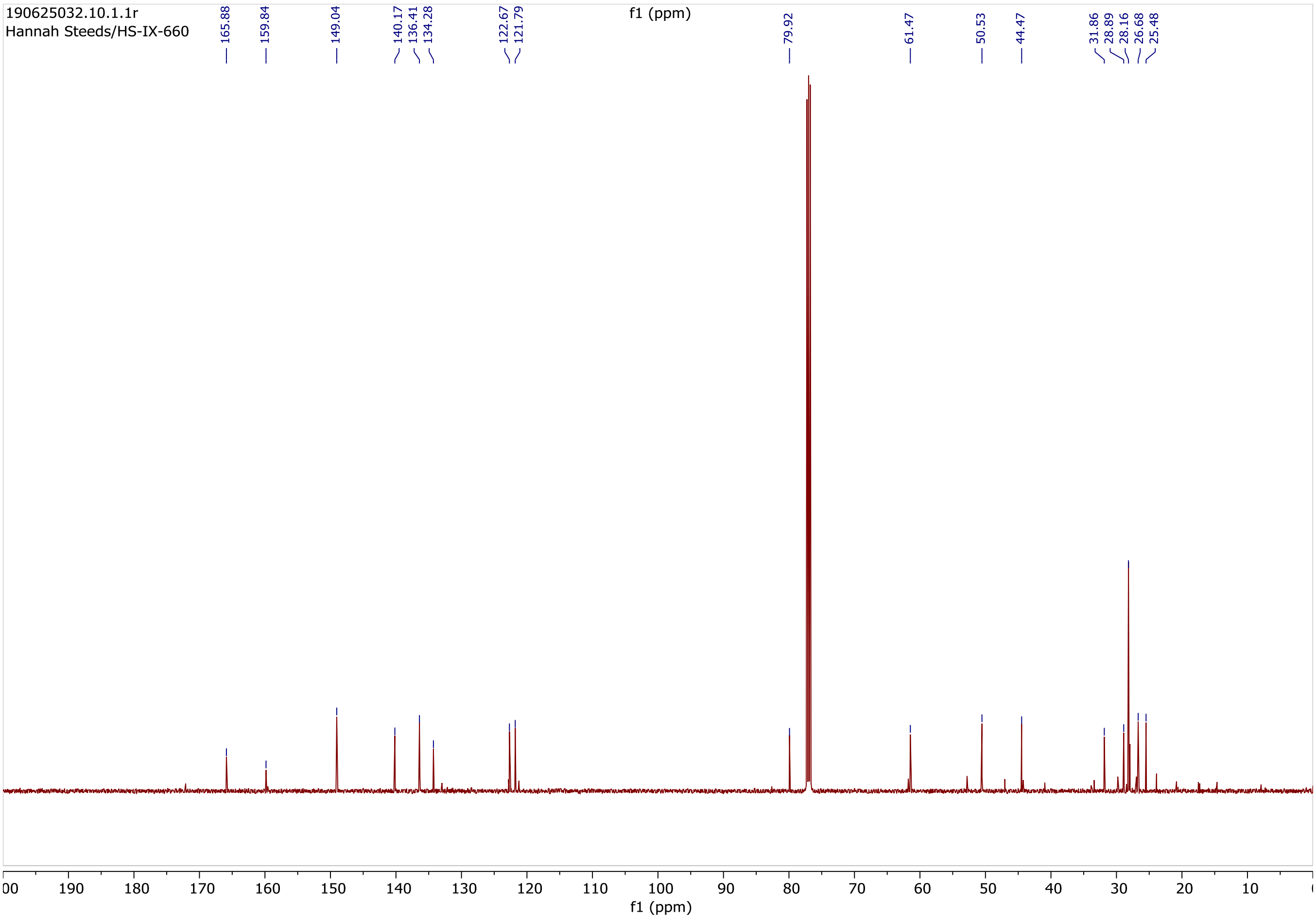

190625032.10.1.1r
Hannah Steeds/HS-IX-660
165.88
159.84
149.04
140.17
136.41
134.28
122.67
121.79
79.92
61.47
50.53
44.47
31.86
28.89
28.16
26.68
25.48
f1 (ppm)

CDCl$_3$

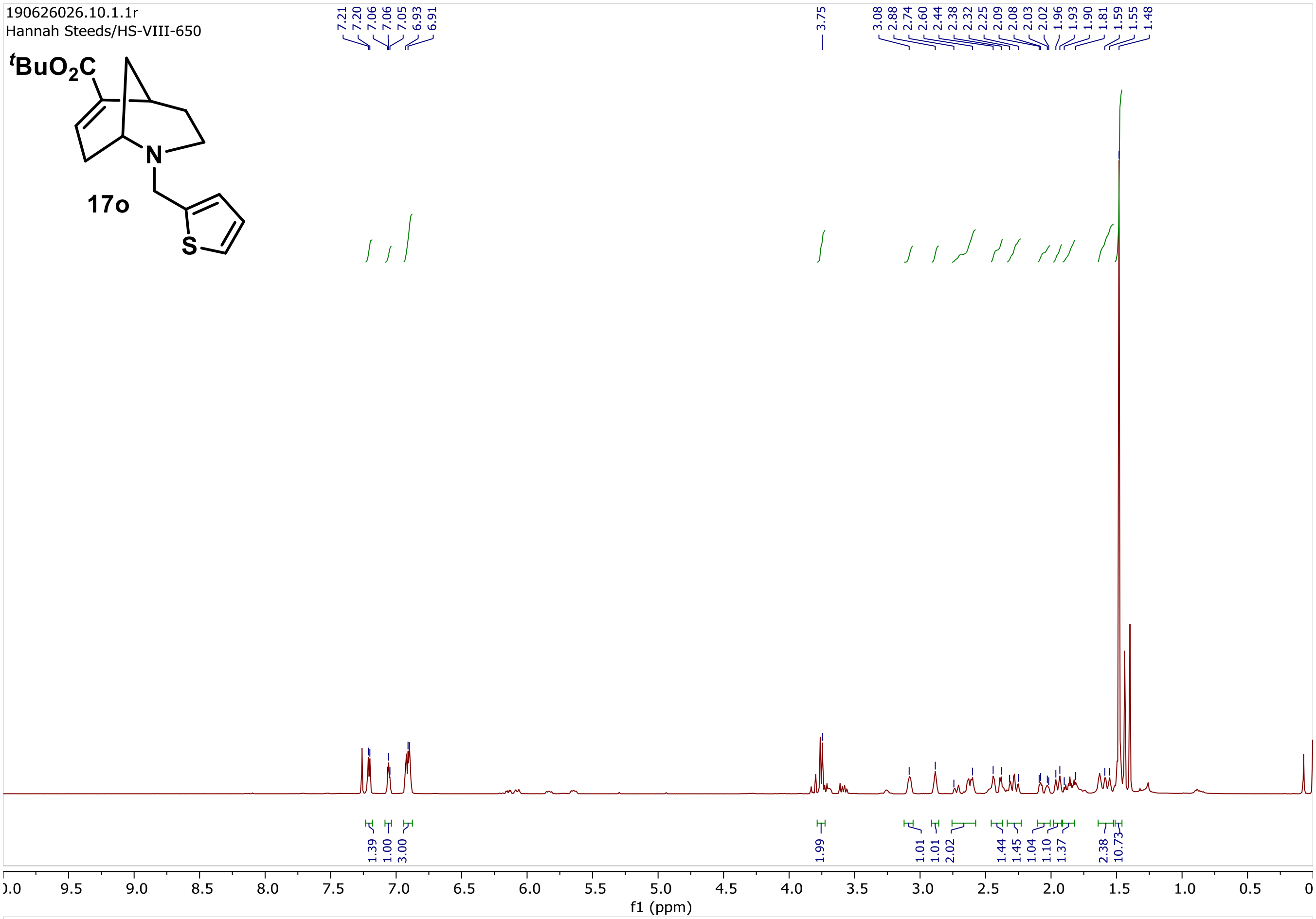
190626026.10.1.1r
Hannah Steeds/HS-VIII-650
tBuO2C
N
S
17o
7.21
7.20
7.06
7.06
7.05
6.93
6.91
3.75
3.08
2.88
2.74
2.60
2.44
2.38
2.32
2.25
2.09
2.08
2.03
2.02
1.96
1.93
1.90
1.81
1.59
1.55
1.48
1.39
1.00
3.00
1.99
1.01
1.01
2.02
1.44
1.45
1.04
1.10
1.37
2.38
10.73
f1 (ppm)

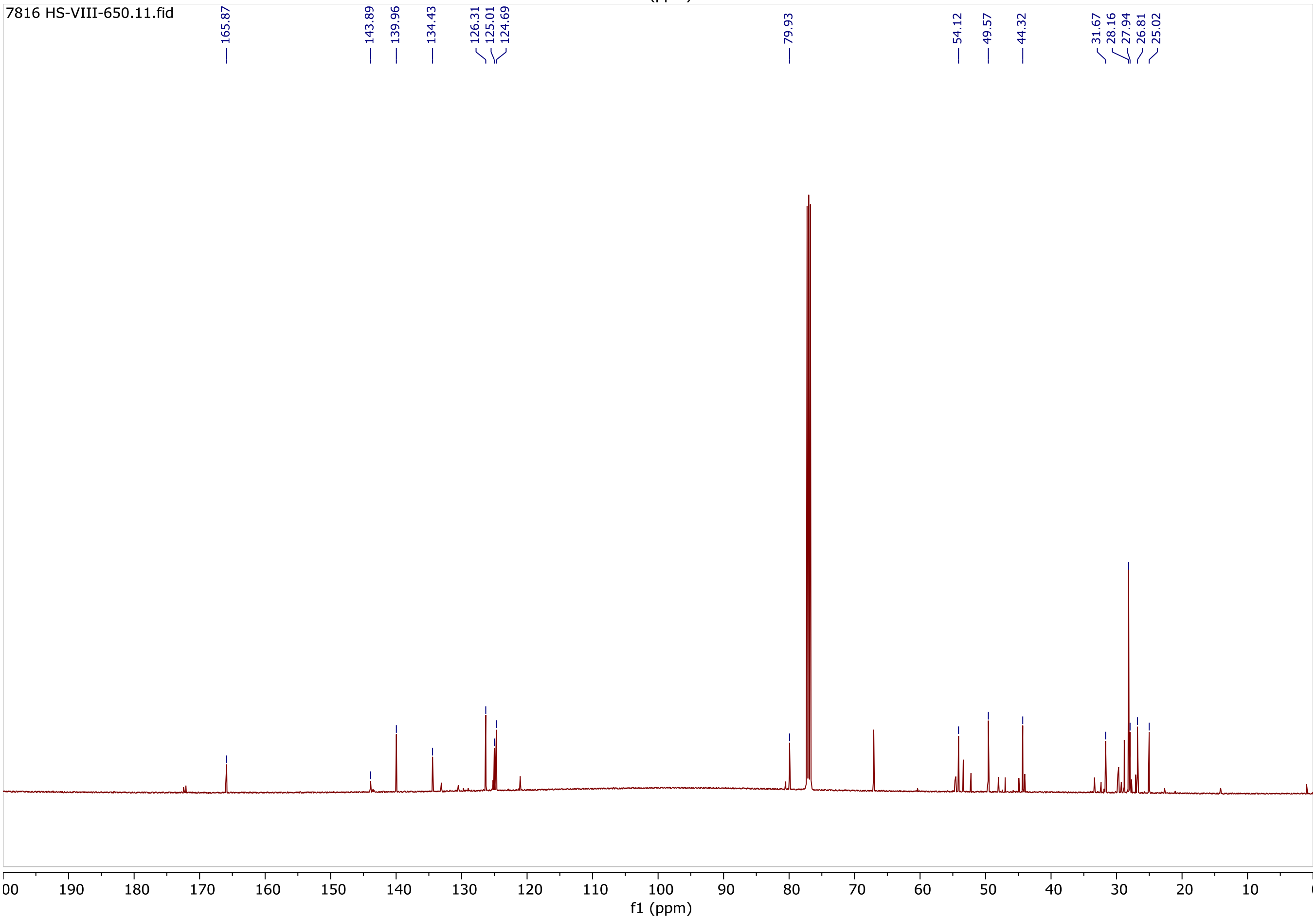
7816 HS-VIII-650.11.fid
165.87
143.89
139.96
134.43
126.31
125.01
124.69
79.93
54.12
49.57
44.32
31.67
28.16
27.94
26.81
25.02
f1 (ppm)

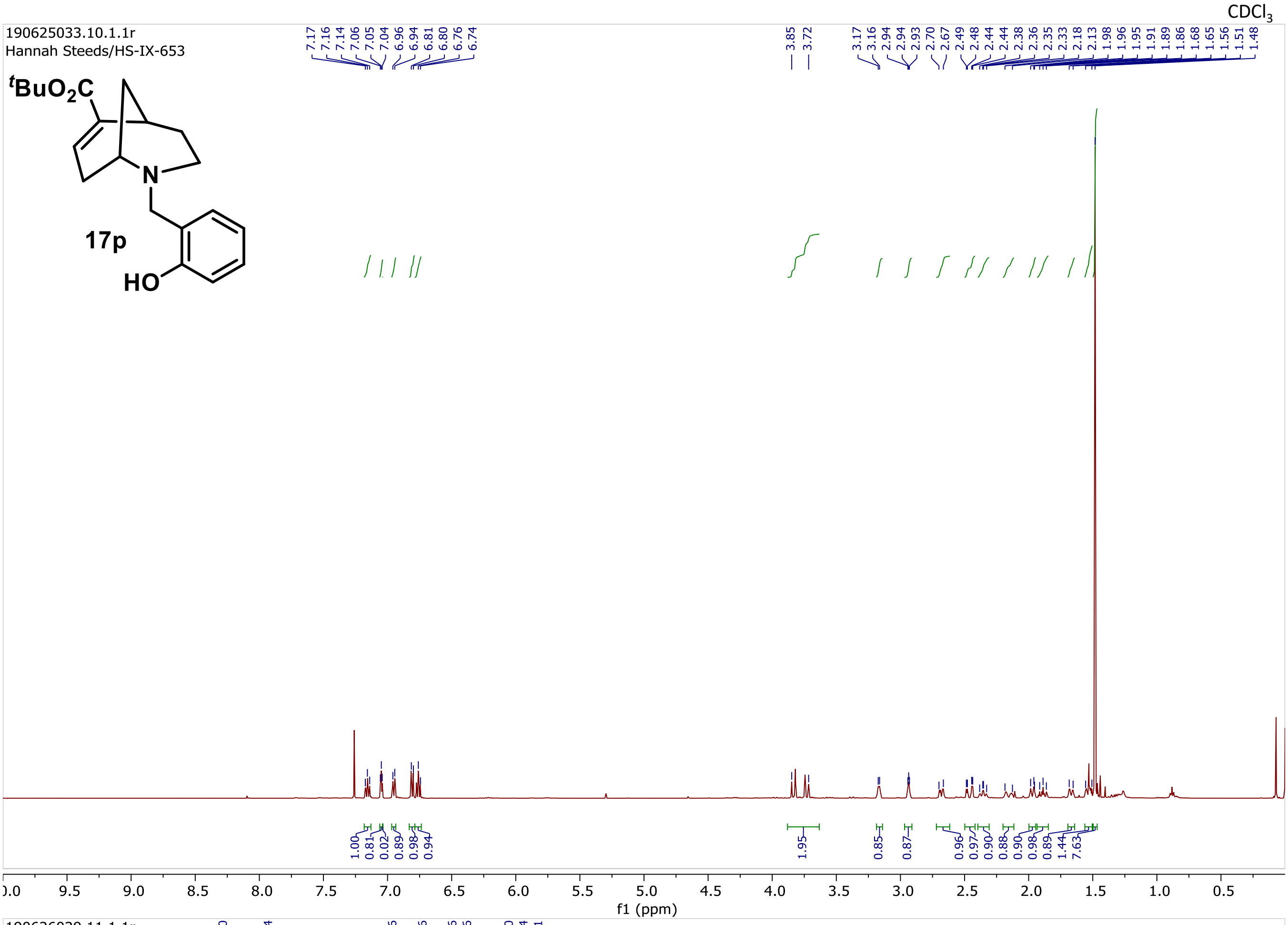
CDCl3
190625033.10.1.1r
Hannah Steeds/HS-IX-653
tBuO2C
N
17p
HO
7.17 7.16 7.14 7.06 7.05 7.04 6.96 6.94 6.81 6.80 6.76 6.74
3.85 3.72
3.17 3.16 2.94 2.94 2.93 2.70 2.67 2.49 2.48 2.44 2.44 2.38 2.36 2.35 2.33 2.18 2.13 1.98 1.96 1.95 1.91 1.89 1.86 1.68 1.65 1.56 1.51 1.48
1.00 0.81 0.02 0.89 0.98 0.94 1.95 0.85 0.87 0.96 0.97 0.90 0.88 0.90 0.98 0.89 1.44 7.63
f1 (ppm)
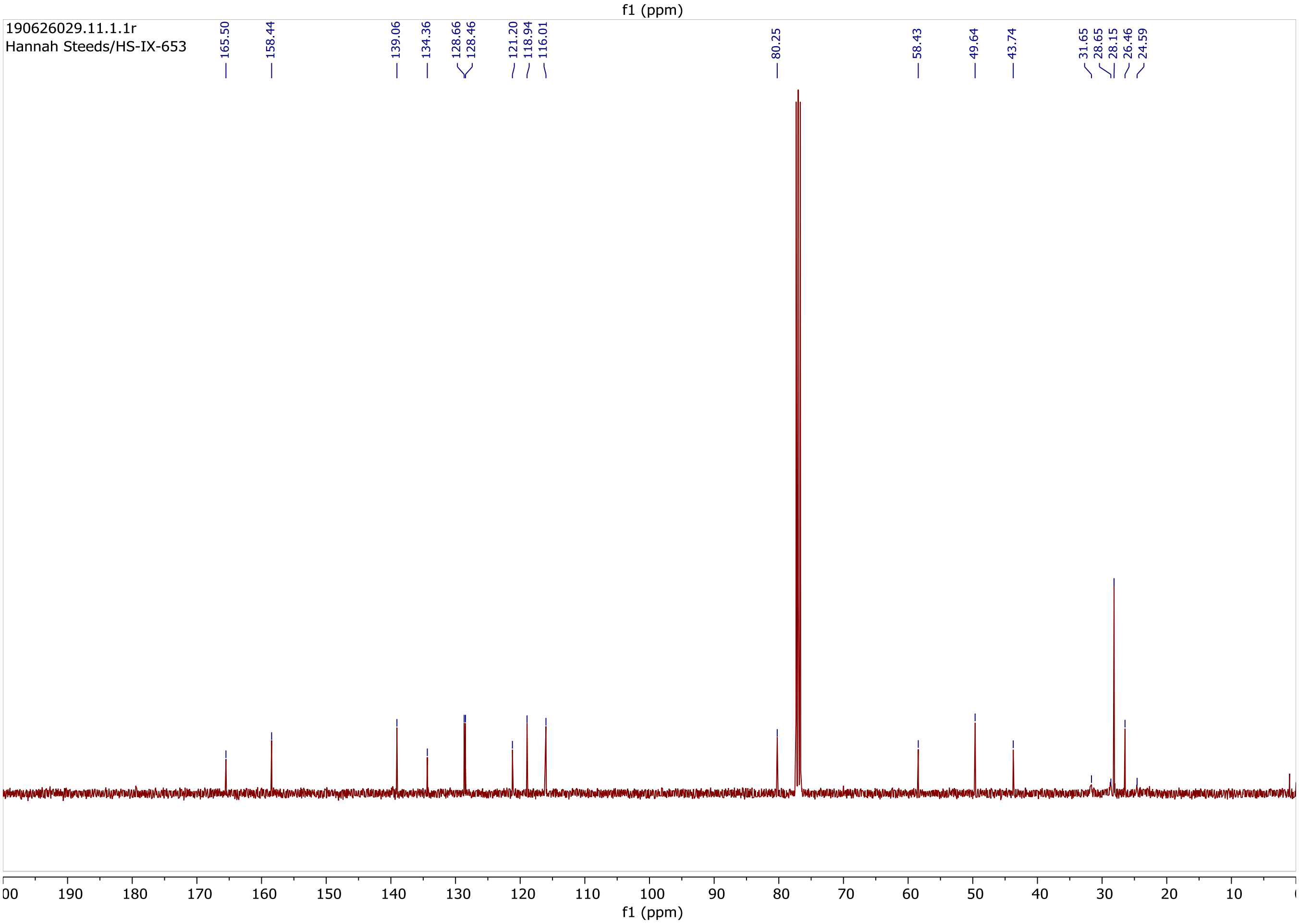
190626029.11.1.1r
Hannah Steeds/HS-IX-653
165.50 158.44 139.06 134.36 128.66 128.46 121.20 118.94 116.01 80.25 58.43 49.64 43.74 31.65 28.65 28.15 26.46 24.59
f1 (ppm)

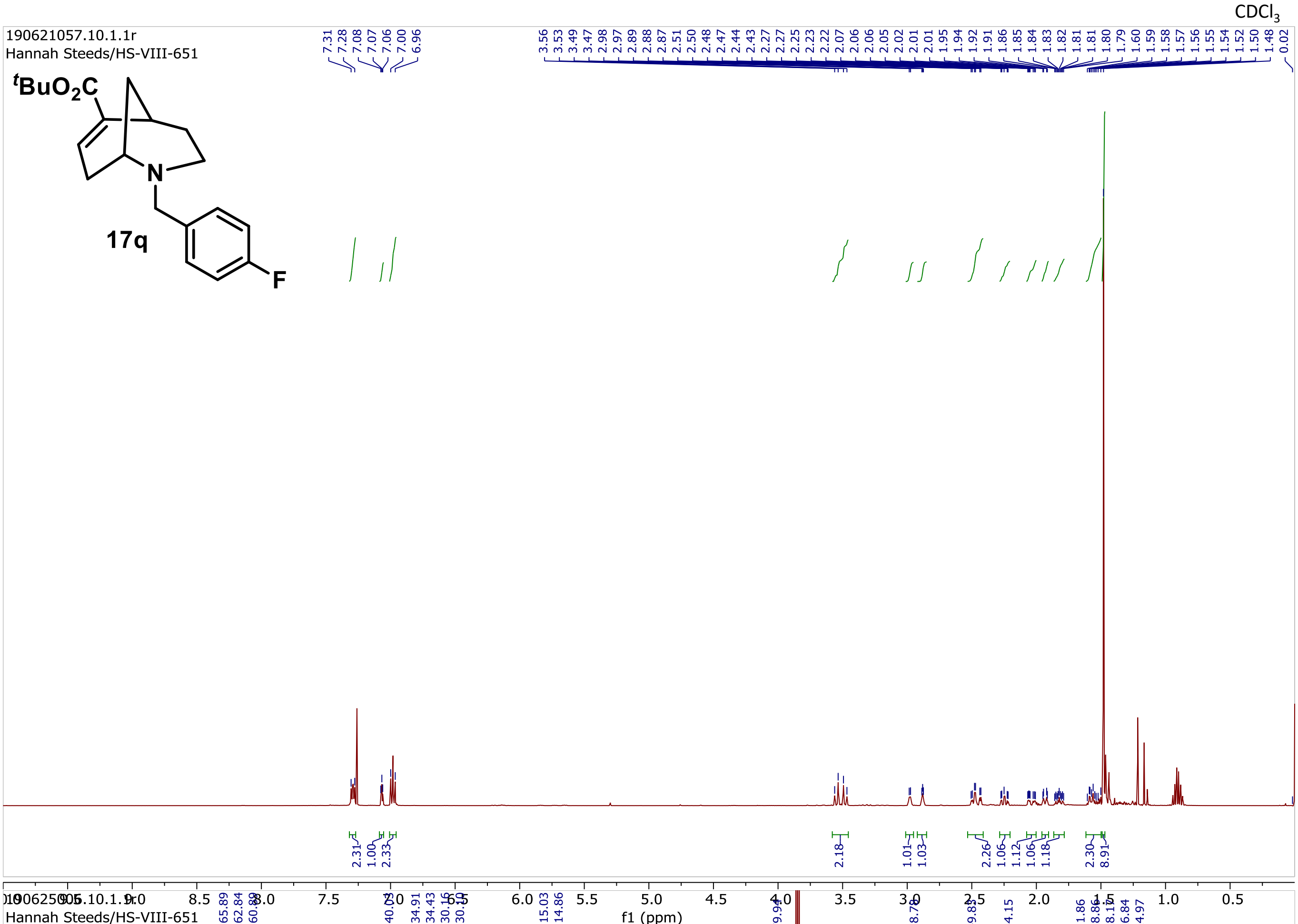
CDCl$_3$
190621057.10.1.1r
Hannah Steeds/HS-VIII-651
17q
f1 (ppm)

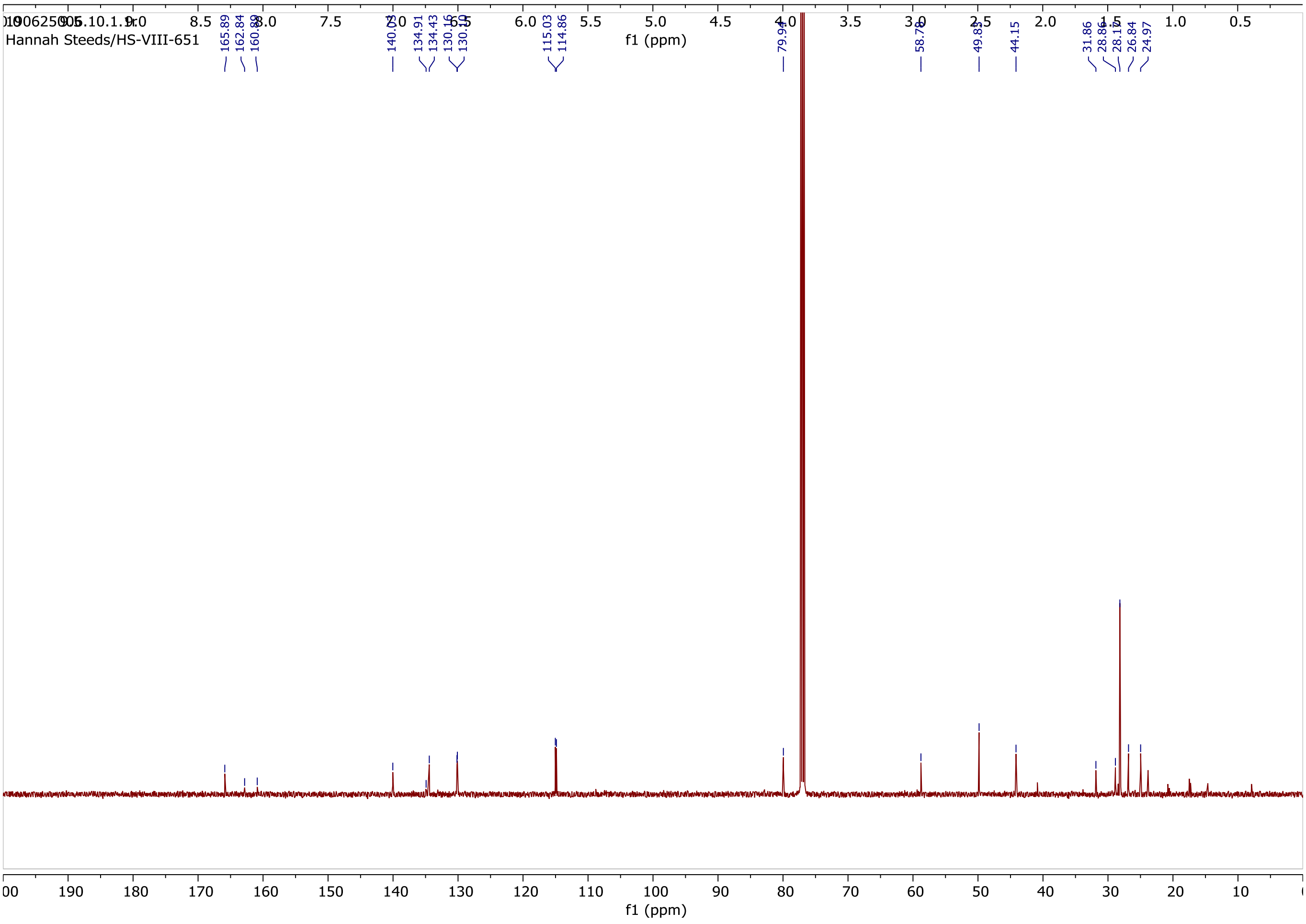
Hannah Steeds/HS-VIII-651
f1 (ppm)

CDCl$_3$

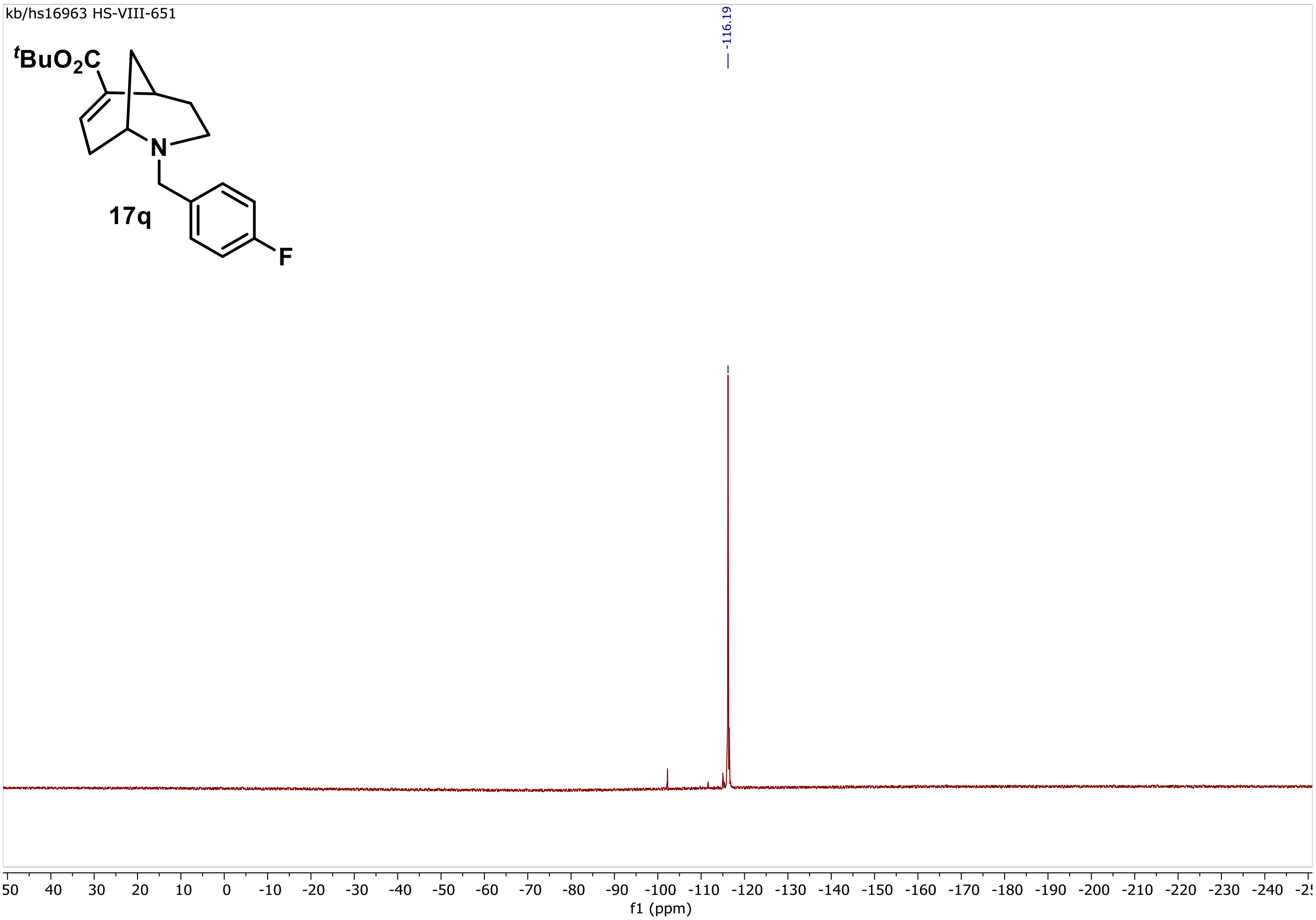
kb/hs16963 HS-VIII-651
^{t}BuO$_2$C
N
17q
F
-116.19
50 40 30 20 10 0 -10 -20 -30 -40 -50 -60 -70 -80 -90 -100 -110 -120 -130 -140 -150 -160 -170 -180 -190 -200 -210 -220 -230 -240
f1 (ppm)

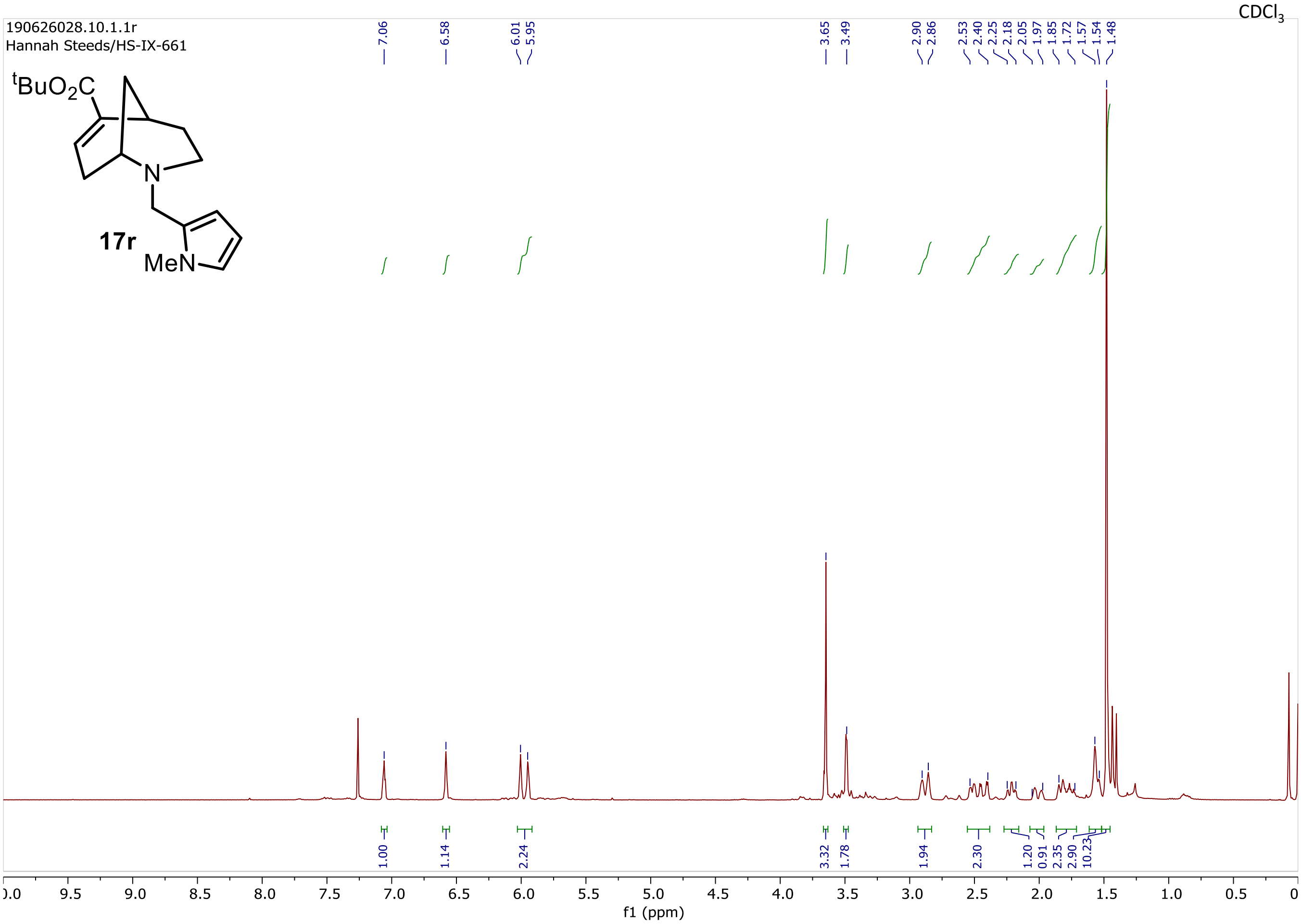
CDCl$_3$
190626028.10.1.1r
Hannah Steeds/HS-IX-661
7.06
6.58
6.01
5.95
3.65
3.49
2.90
2.86
2.53
2.40
2.25
2.18
2.05
1.97
1.85
1.72
1.57
1.54
1.48
^{t}BuO$_2$C
N
17r
MeN
1.00
1.14
2.24
3.32
1.78
1.94
2.30
1.20
0.91
2.35
2.90
10.23
f1 (ppm)

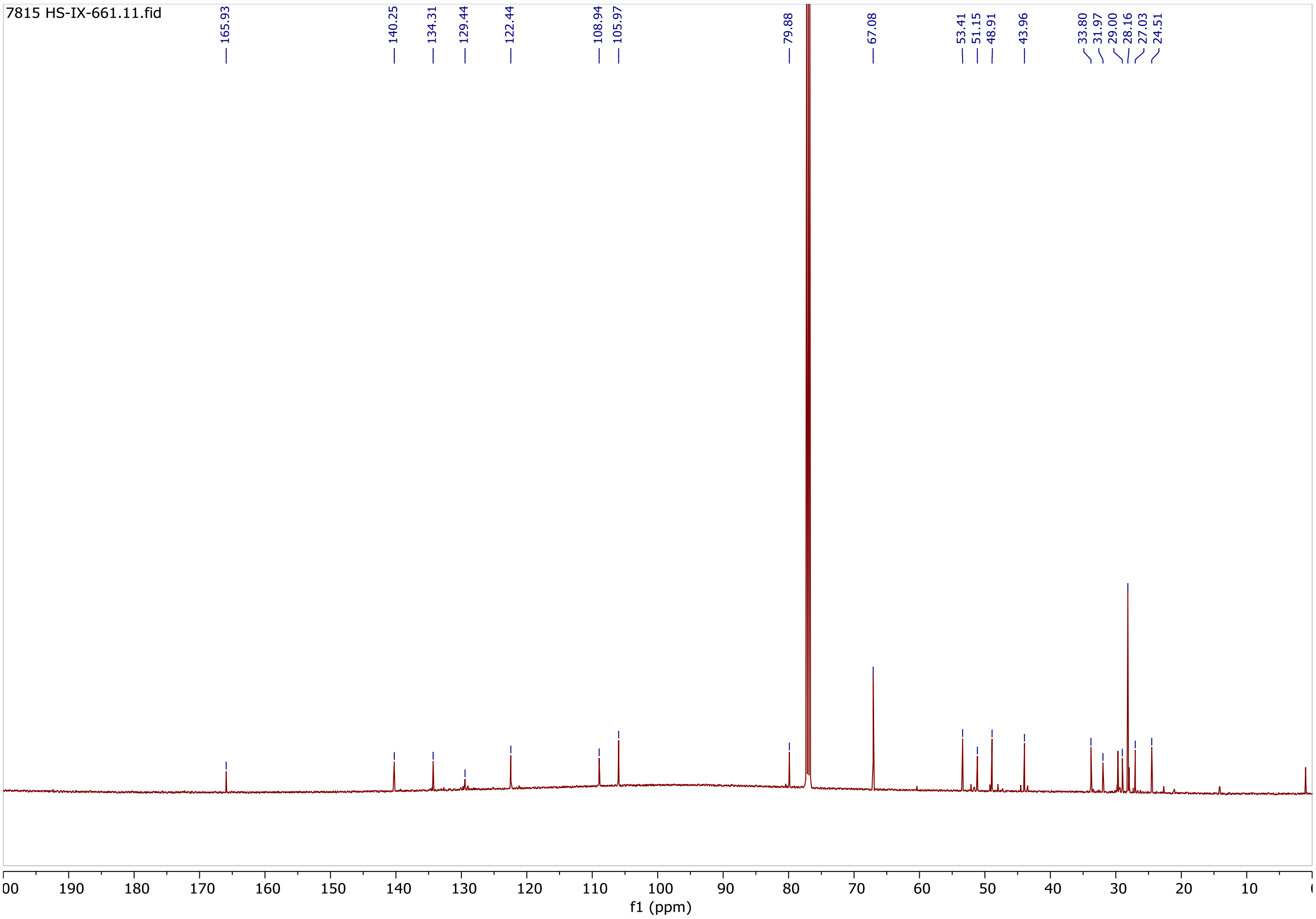
7815 HS-IX-661.11.fid
165.93
140.25
134.31
129.44
122.44
108.94
105.97
79.88
67.08
53.41
51.15
48.91
43.96
33.80
31.97
29.00
28.16
27.03
24.51
f1 (ppm)

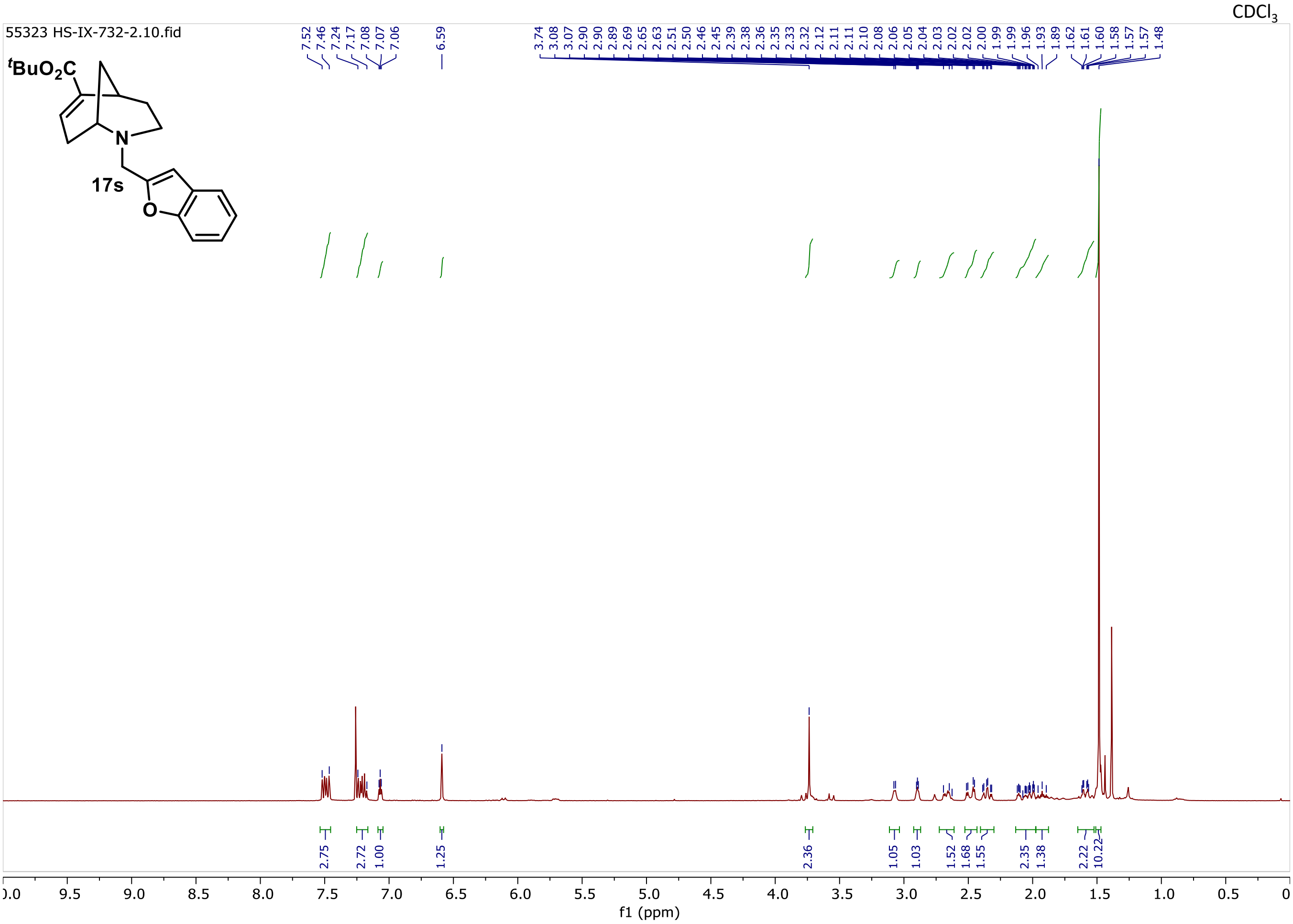
CDCl$_3$
55323 HS-IX-732-2.10.fid
^{t}BuO$_2$C
N
O
17s
7.52 7.46 7.24 7.17 7.08 7.07 7.06
6.59
3.74 3.08 3.07 2.90 2.89 2.69 2.65 2.63 2.51 2.50 2.46 2.45 2.39 2.38 2.36 2.35 2.33 2.32 2.12 2.11 2.11 2.10 2.08 2.06 2.05 2.04 2.03 2.02 2.02 2.00 1.99 1.99 1.96 1.93 1.89 1.62 1.61 1.60 1.58 1.57 1.57 1.48
2.75 2.72 1.00 1.25 2.36 1.05 1.03 1.52 1.68 1.55 2.35 1.38 2.22 10.22
f1 (ppm)

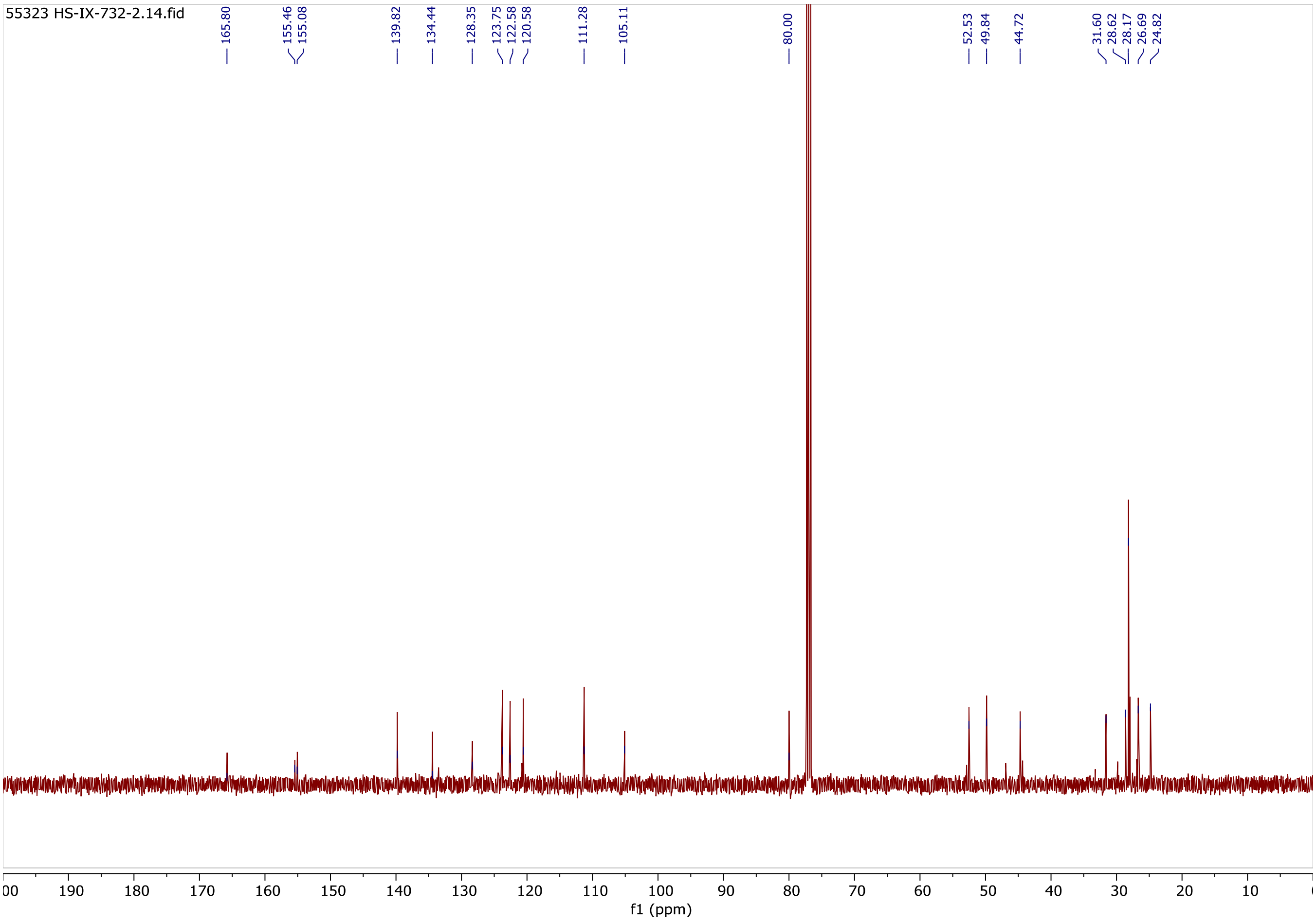
55323 HS-IX-732-2.14.fid
165.80
155.46 155.08
139.82
134.44
128.35
123.75 122.58 120.58
111.28
105.11
80.00
52.53 49.84
44.72
31.60 28.62 28.17 26.69 24.82
f1 (ppm)

CDCl$_3$

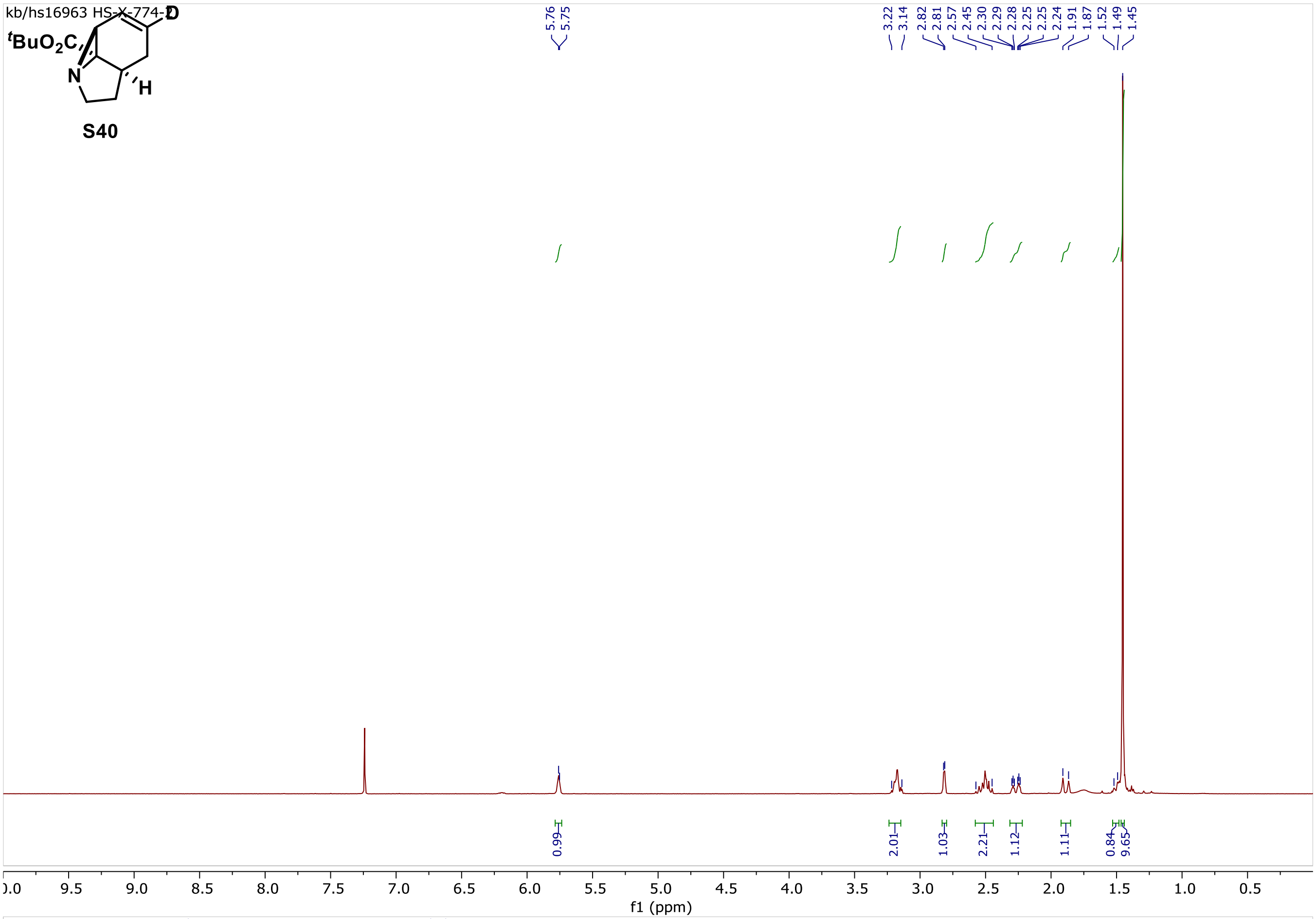

kb/hs16963 HS-X-774-D
^{t}BuO$_2$C
N
D
H
S40
5.76
5.75
3.22
3.14
2.82
2.81
2.57
2.45
2.30
2.29
2.28
2.25
2.25
2.24
1.91
1.87
1.52
1.49
1.45
0.99
2.01
1.03
2.21
1.12
1.11
0.84
9.65
f1 (ppm)

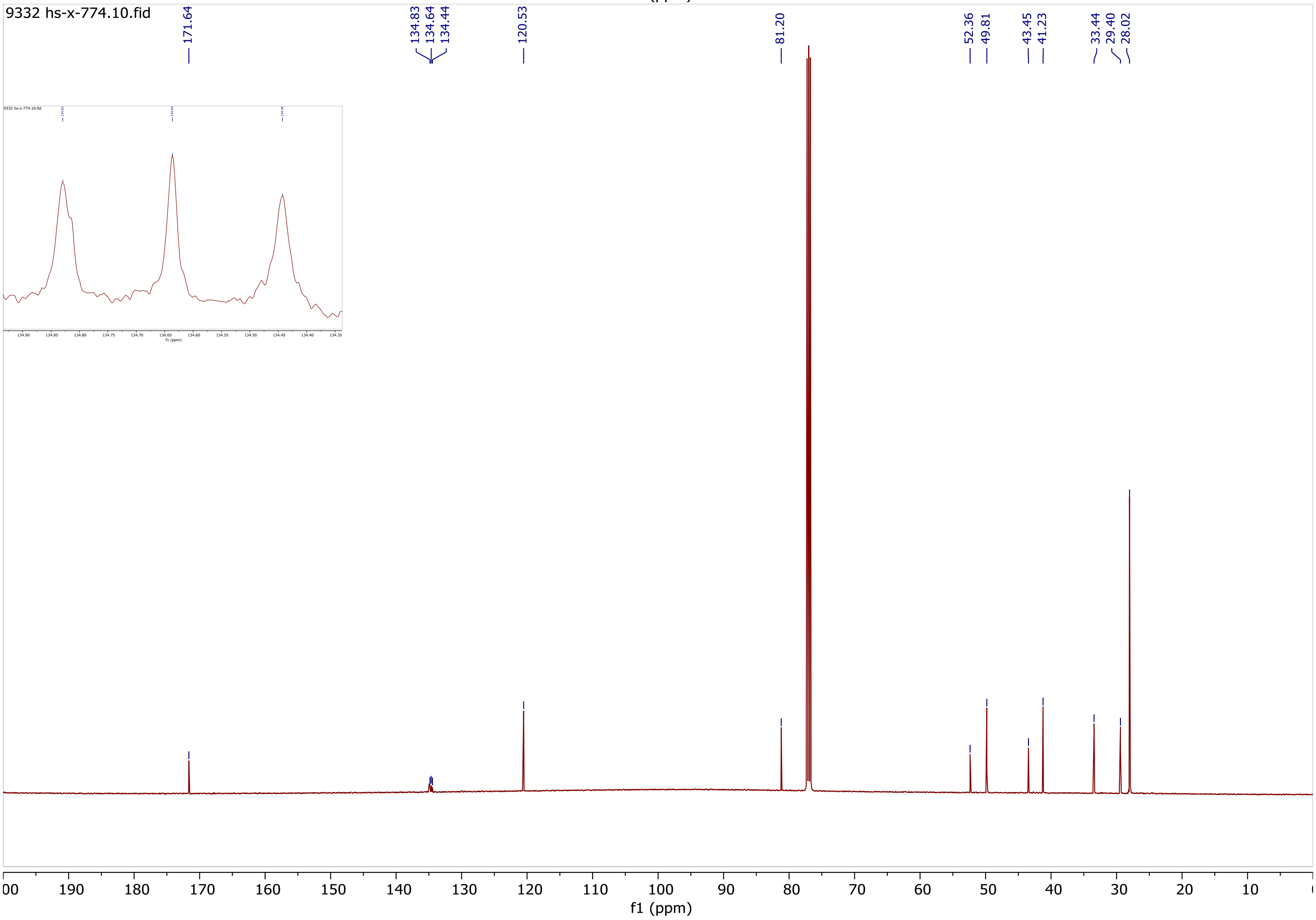

9332 hs-x-774.10.fid
171.64
134.83
134.64
134.44
120.53
81.20
52.36
49.81
43.45
41.23
33.44
29.40
28.02
f1 (ppm)

CDCl$_3$

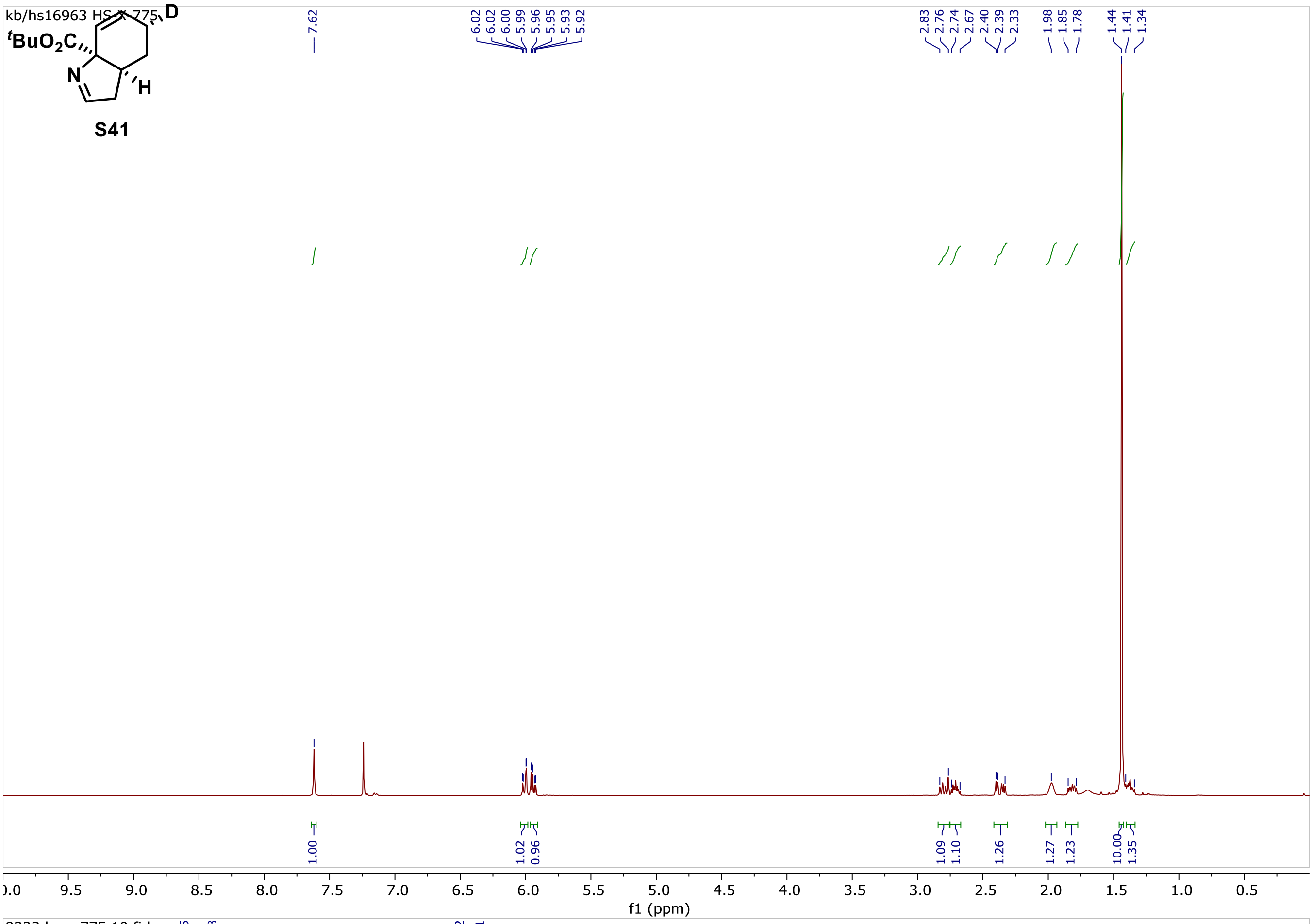
kb/hs16963 HS-X-775
^{t}BuO$_2$C
N
D
H
S41
7.62
6.02 6.02 6.00 5.99 5.96 5.95 5.93 5.92
2.83 2.76 2.74 2.67 2.40 2.39 2.33 1.98 1.85 1.78 1.44 1.41 1.34
1.00
1.02 0.96
1.09 1.10 1.26 1.27 1.23 10.00 1.35
f1 (ppm)

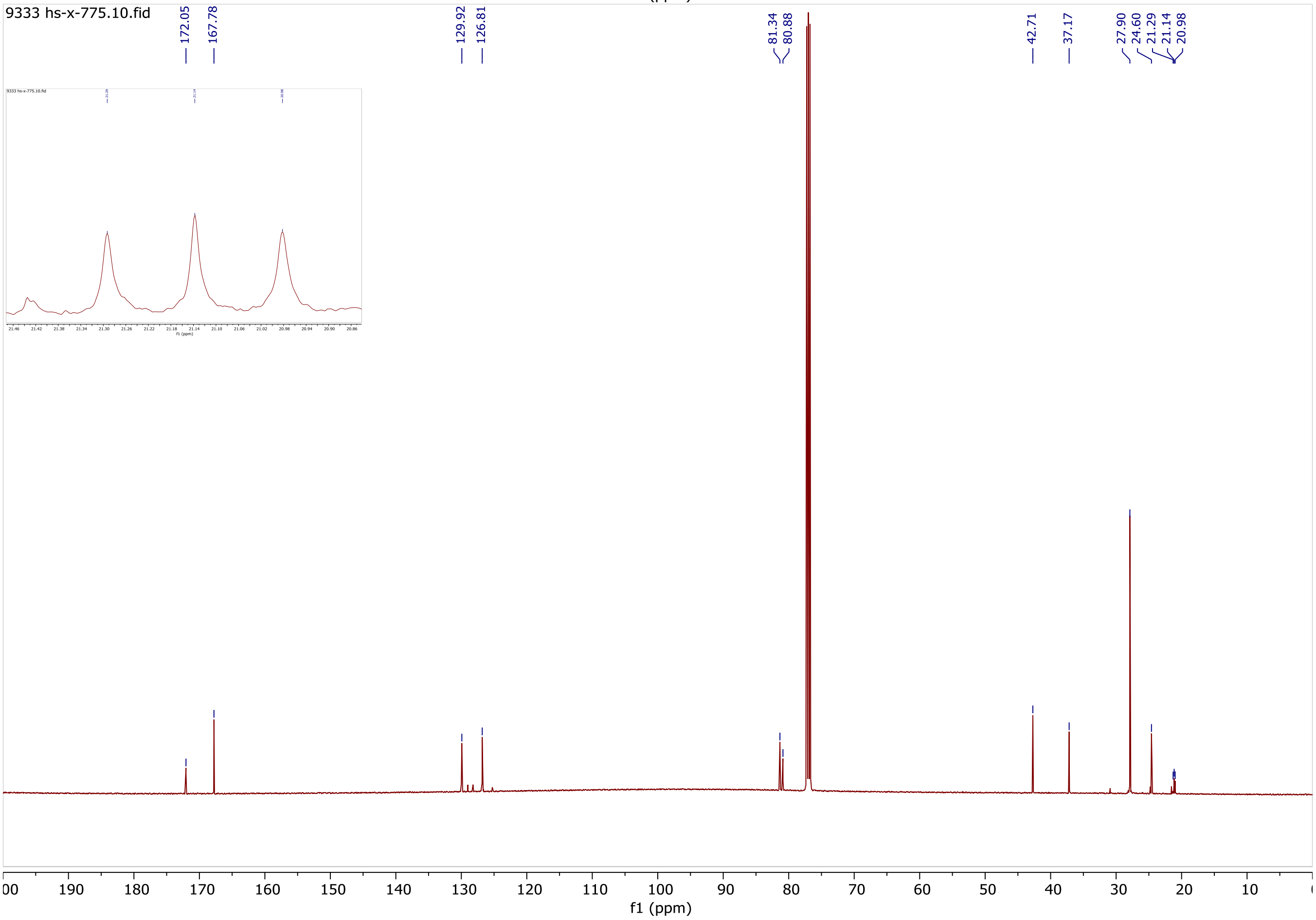
9333 hs-x-775.10.fid
172.05 167.78
129.92 126.81
81.34 80.88
42.71 37.17
27.90 24.60 21.29 21.14 20.98
f1 (ppm)

CDCl$_3$

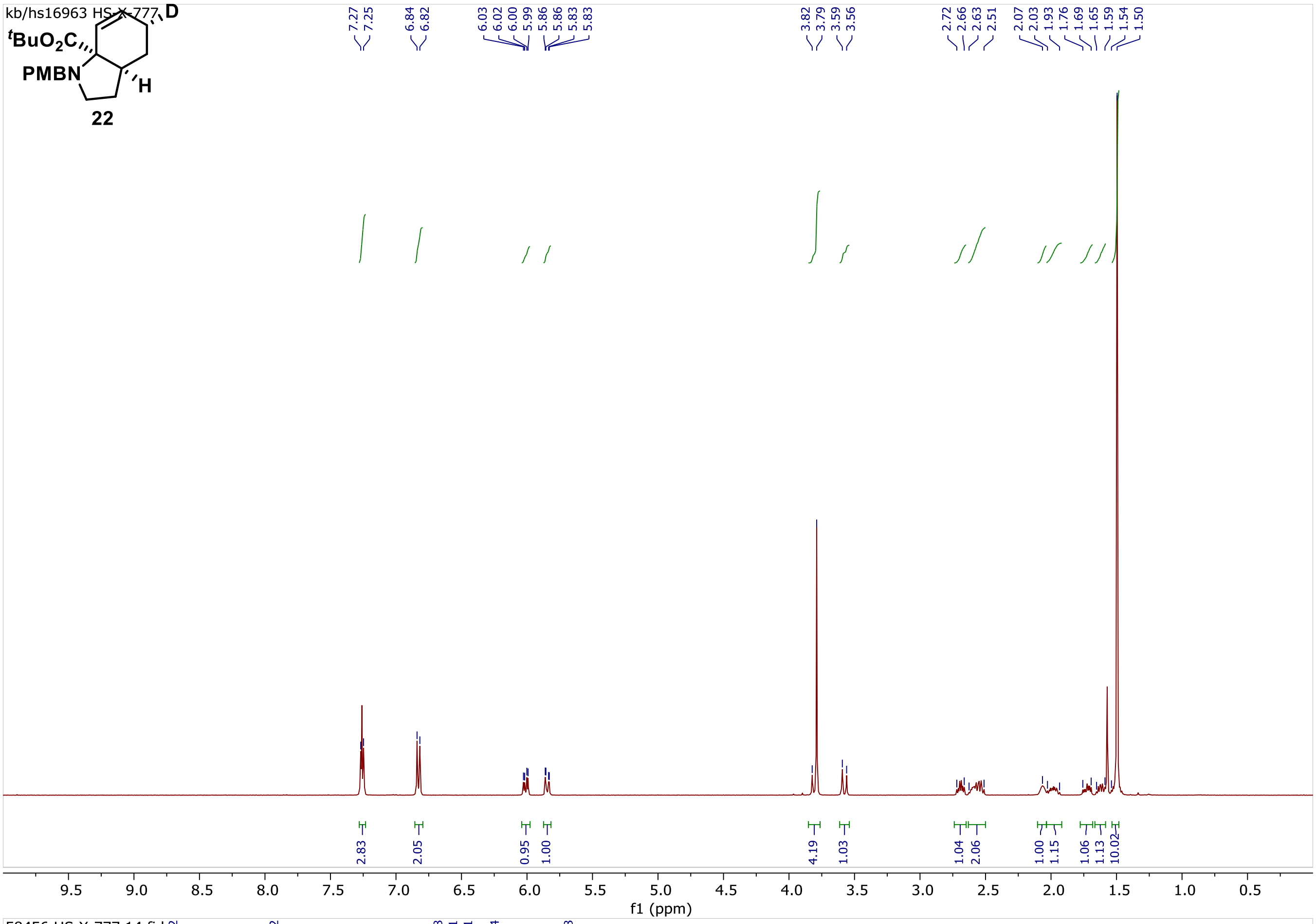

kb/hs16963 HS-X-777
D
^{t}BuO$_2$C
PMBN
H
22
7.27 7.25 6.84 6.82 6.03 6.02 6.00 5.99 5.86 5.86 5.83 5.83 3.82 3.79 3.59 3.56 2.72 2.66 2.63 2.51 2.07 2.03 1.93 1.76 1.69 1.65 1.59 1.54 1.50
2.83 2.05 0.95 1.00 4.19 1.03 1.04 2.06 1.00 1.15 1.06 1.13 10.02
9.5 9.0 8.5 8.0 7.5 7.0 6.5 6.0 5.5 5.0 4.5 4.0 3.5 3.0 2.5 2.0 1.5 1.0 0.5
f1 (ppm)

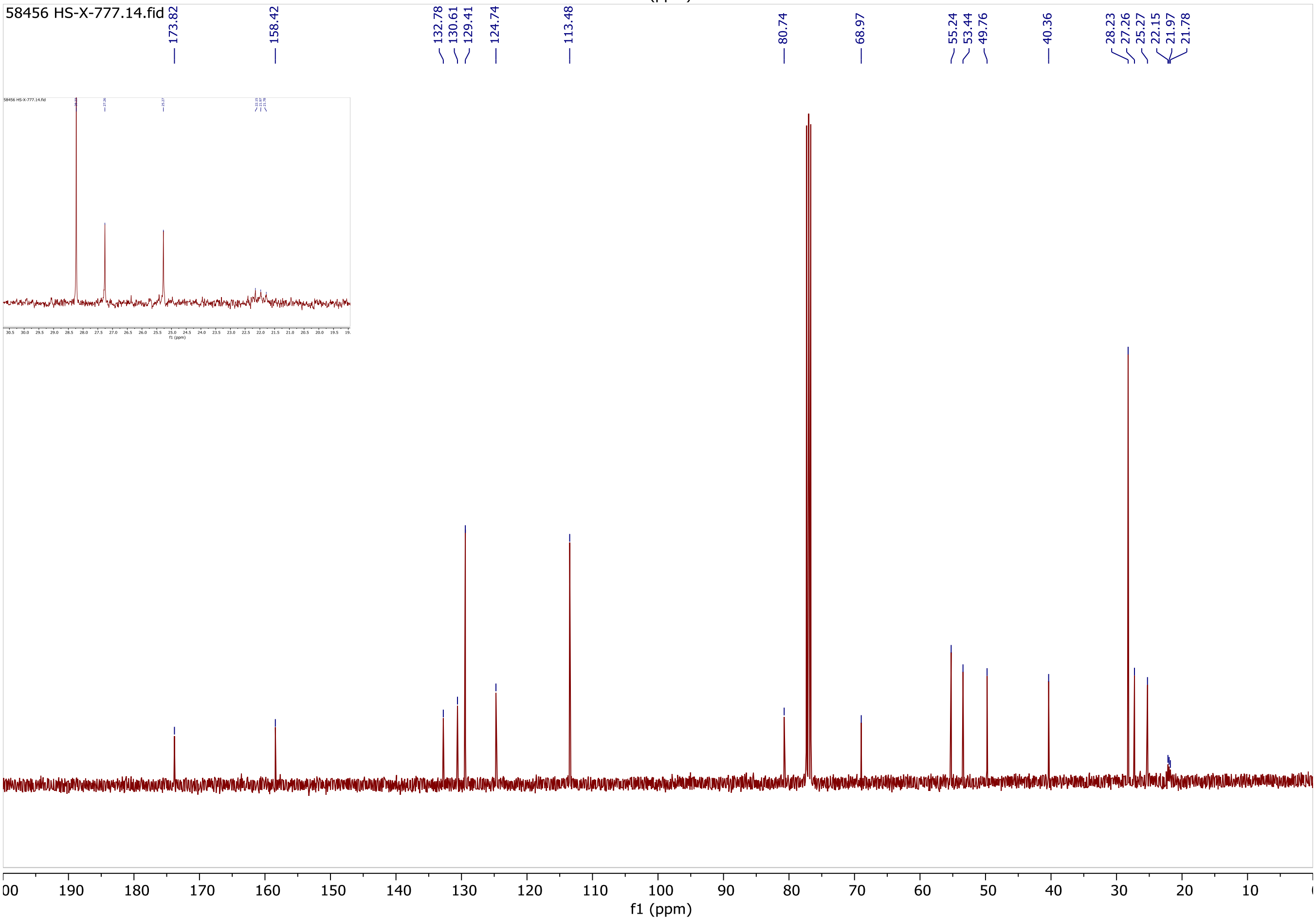

58456 HS-X-777.14.fid
173.82 158.42 132.78 130.61 129.41 124.74 113.48 80.74 68.97 55.24 53.44 49.76 40.36 28.23 27.26 25.27 22.15 21.97 21.78
00 190 180 170 160 150 140 130 120 110 100 90 80 70 60 50 40 30 20 10
f1 (ppm)

CDCl$_3$

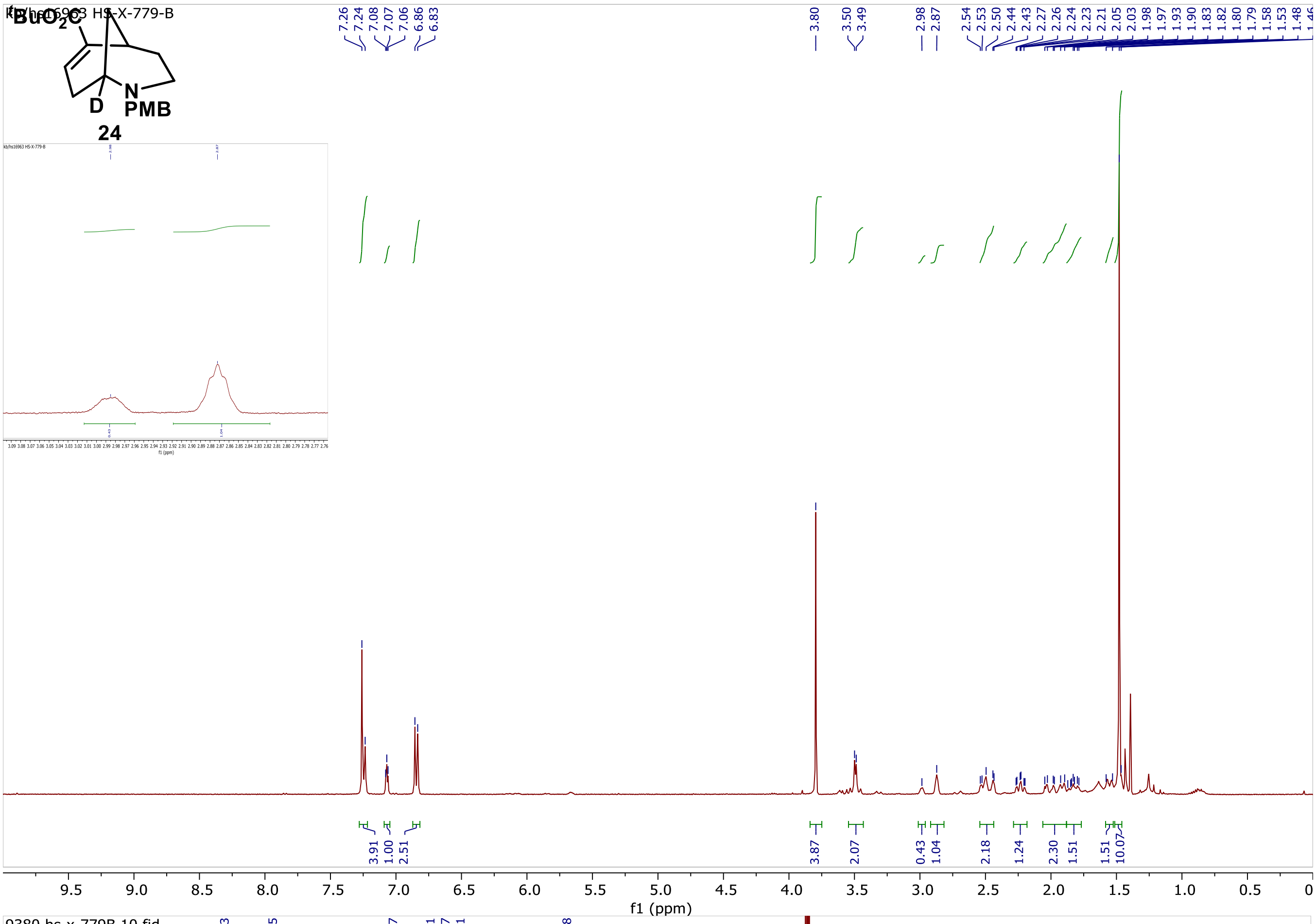
kb/hs16963 HS-X-779-B
7.26
7.24
7.08
7.07
7.06
6.86
6.83
3.80
3.50
3.49
2.98
2.87
2.54
2.53
2.50
2.44
2.43
2.27
2.26
2.24
2.23
2.21
2.05
2.03
1.98
1.97
1.93
1.90
1.83
1.82
1.80
1.79
1.58
1.53
1.48
3.91
1.00
2.51
3.87
2.07
0.43
1.04
2.18
1.24
2.30
1.51
1.51
10.07
9.5
9.0
8.5
8.0
7.5
7.0
6.5
6.0
5.5
5.0
4.5
4.0
3.5
3.0
2.5
2.0
1.5
1.0
0.5
f1 (ppm)

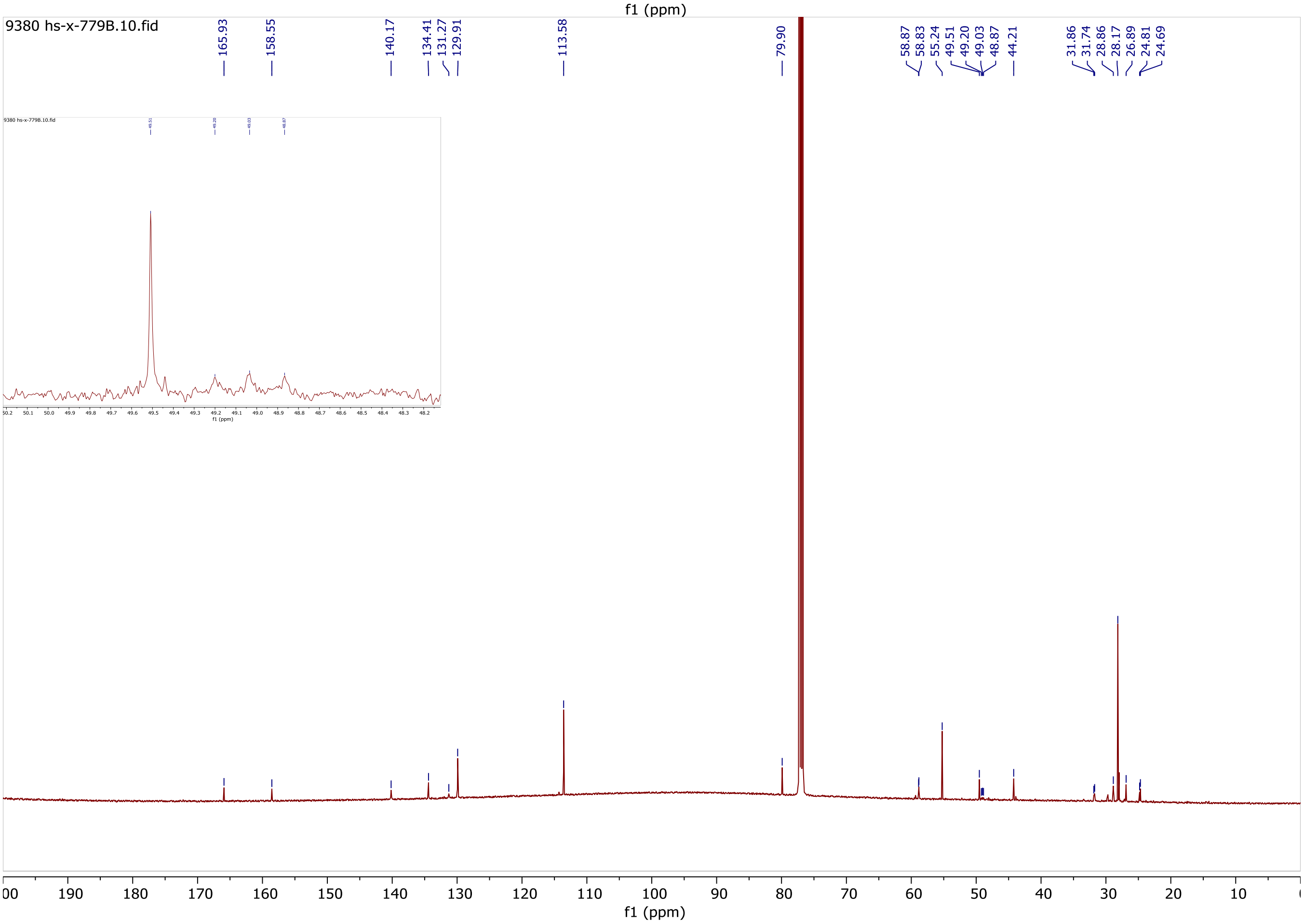
9380 hs-x-779B.10.fid
165.93
158.55
140.17
134.41
131.27
129.91
113.58
79.90
58.87
58.83
55.24
49.51
49.20
49.03
48.87
44.21
31.86
31.74
28.86
28.17
26.89
24.81
24.69
190
180
170
160
150
140
130
120
110
100
90
80
70
60
50
40
30
20
10
f1 (ppm)

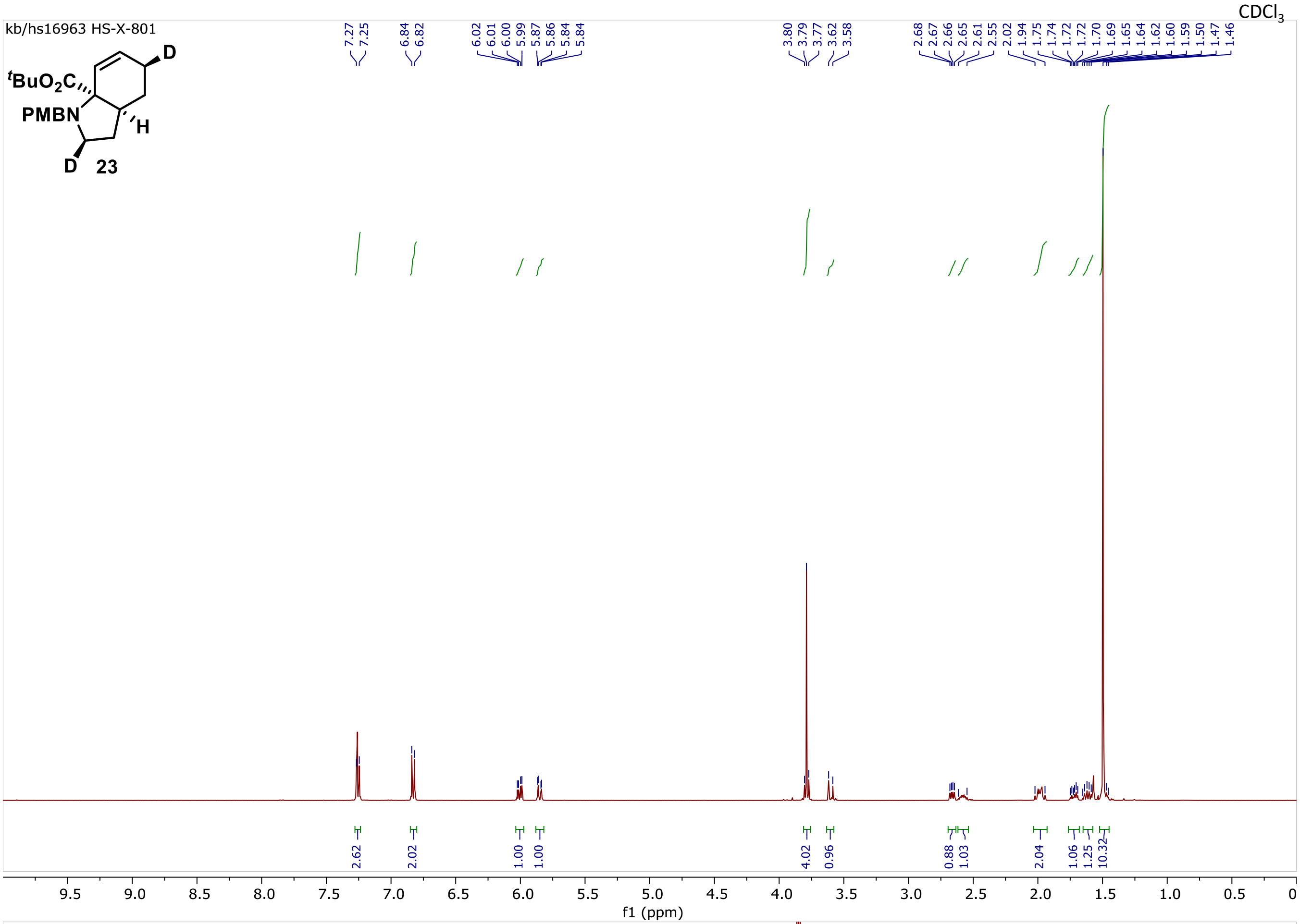
CDCl3
kb/hs16963 HS-X-801
tBuO2C
PMBN
D
H
D
23
7.27
7.25
6.84
6.82
6.02
6.01
6.00
5.99
5.87
5.86
5.84
5.84
3.80
3.79
3.77
3.62
3.58
2.68
2.67
2.66
2.65
2.61
2.55
2.02
1.94
1.75
1.74
1.72
1.70
1.69
1.65
1.64
1.62
1.60
1.59
1.50
1.47
1.46
2.62
2.02
1.00
1.00
4.02
0.96
0.88
1.03
2.04
1.06
1.25
10.32
f1 (ppm)

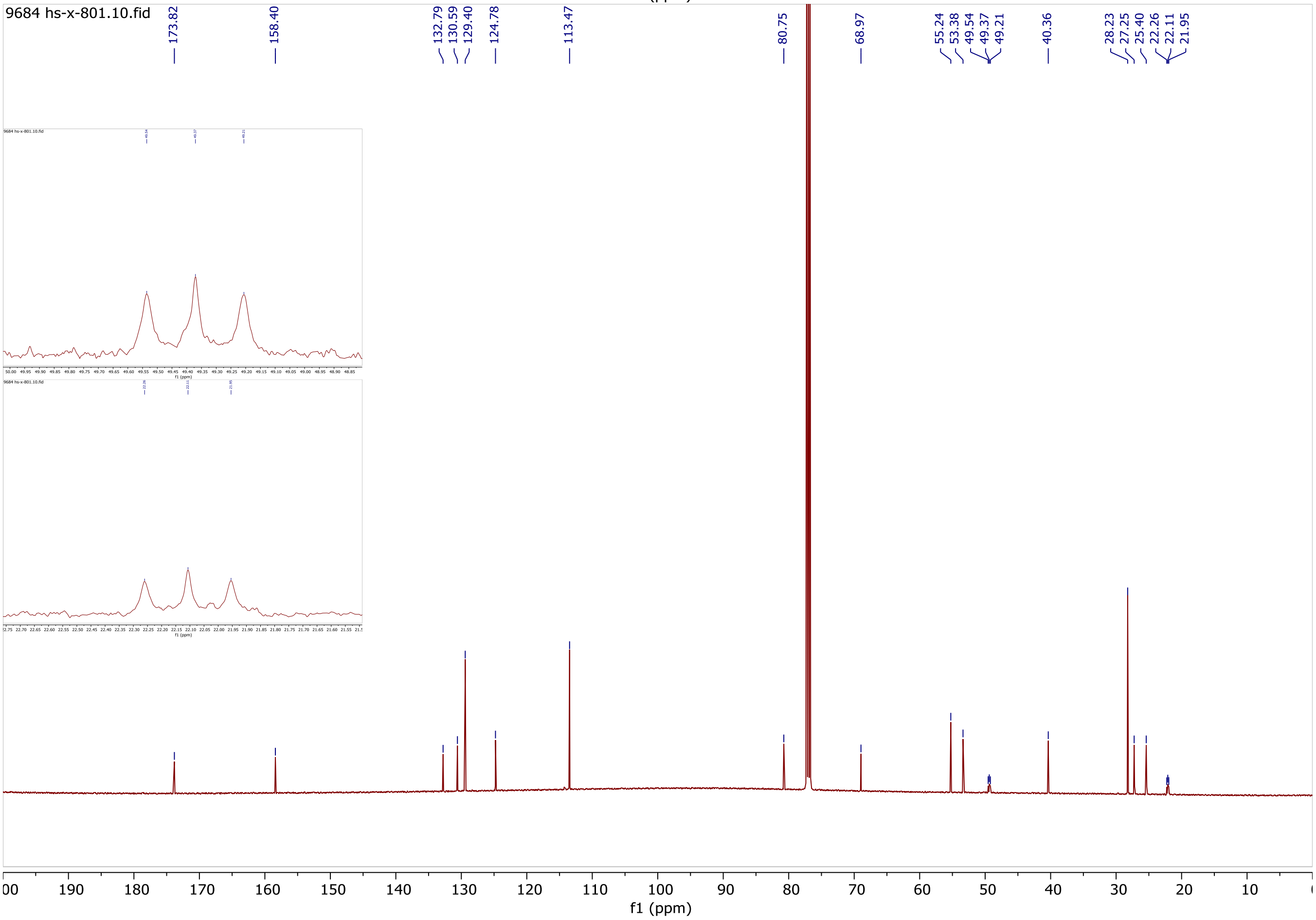
9684 hs-x-801.10.fid
173.82
158.40
132.79
130.59
129.40
124.78
113.47
80.75
68.97
55.24
53.38
49.54
49.37
49.21
40.36
28.23
27.25
25.40
22.26
22.11
21.95
f1 (ppm)

CDCl$_3$

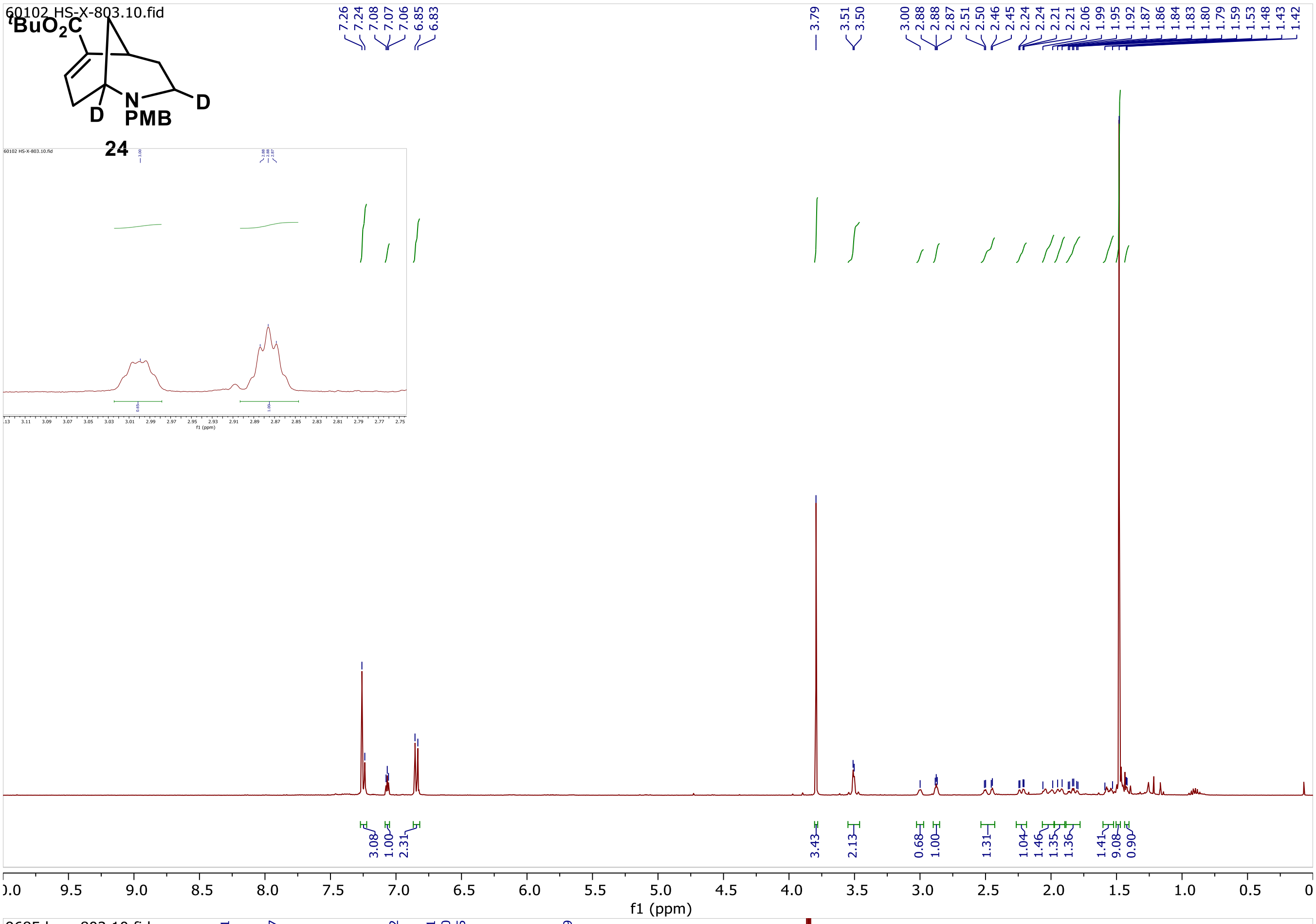

60102 HS-X-803.10.fid
^{t}BuO$_2$C
N
PMB
D
D
24
7.26 7.24 7.08 7.07 7.06 6.85 6.83
3.79
3.51 3.50
3.00 2.88 2.88 2.87 2.51 2.50 2.46 2.45 2.24 2.24 2.21 2.06 1.99 1.95 1.92 1.87 1.86 1.84 1.83 1.80 1.79 1.59 1.53 1.48 1.43 1.42
3.08 1.00 2.31 3.43 2.13 0.68 1.00 1.31 1.04 1.46 1.35 1.36 1.41 9.08 0.90
f1 (ppm)

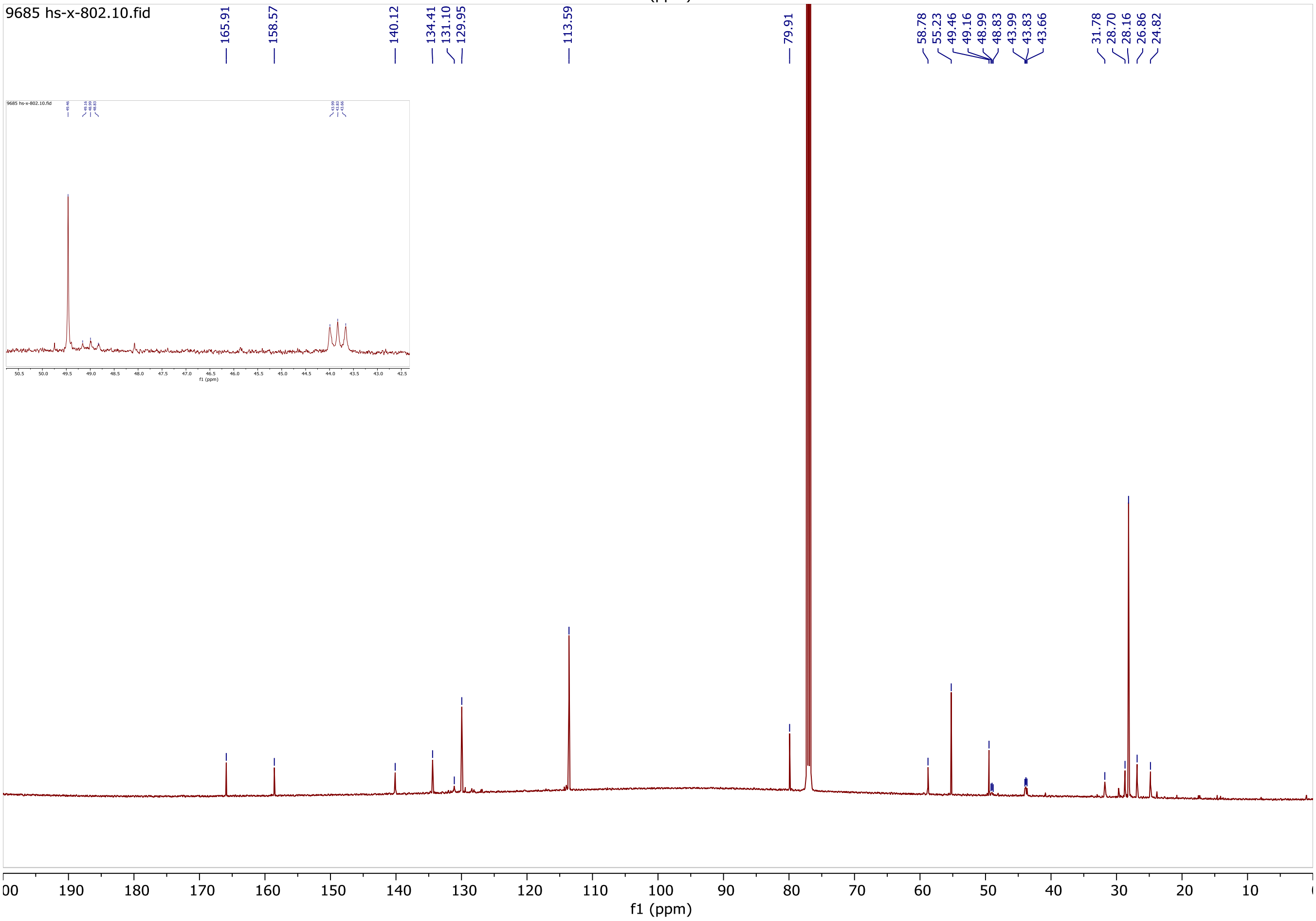

9685 hs-x-802.10.fid
165.91
158.57
140.12
134.41 131.10 129.95
113.59
79.91
58.78 55.23 49.46 49.16 48.99 48.83 43.99 43.83 43.66
31.78 28.70 28.16 26.86 24.82
f1 (ppm)

CDCl$_3$

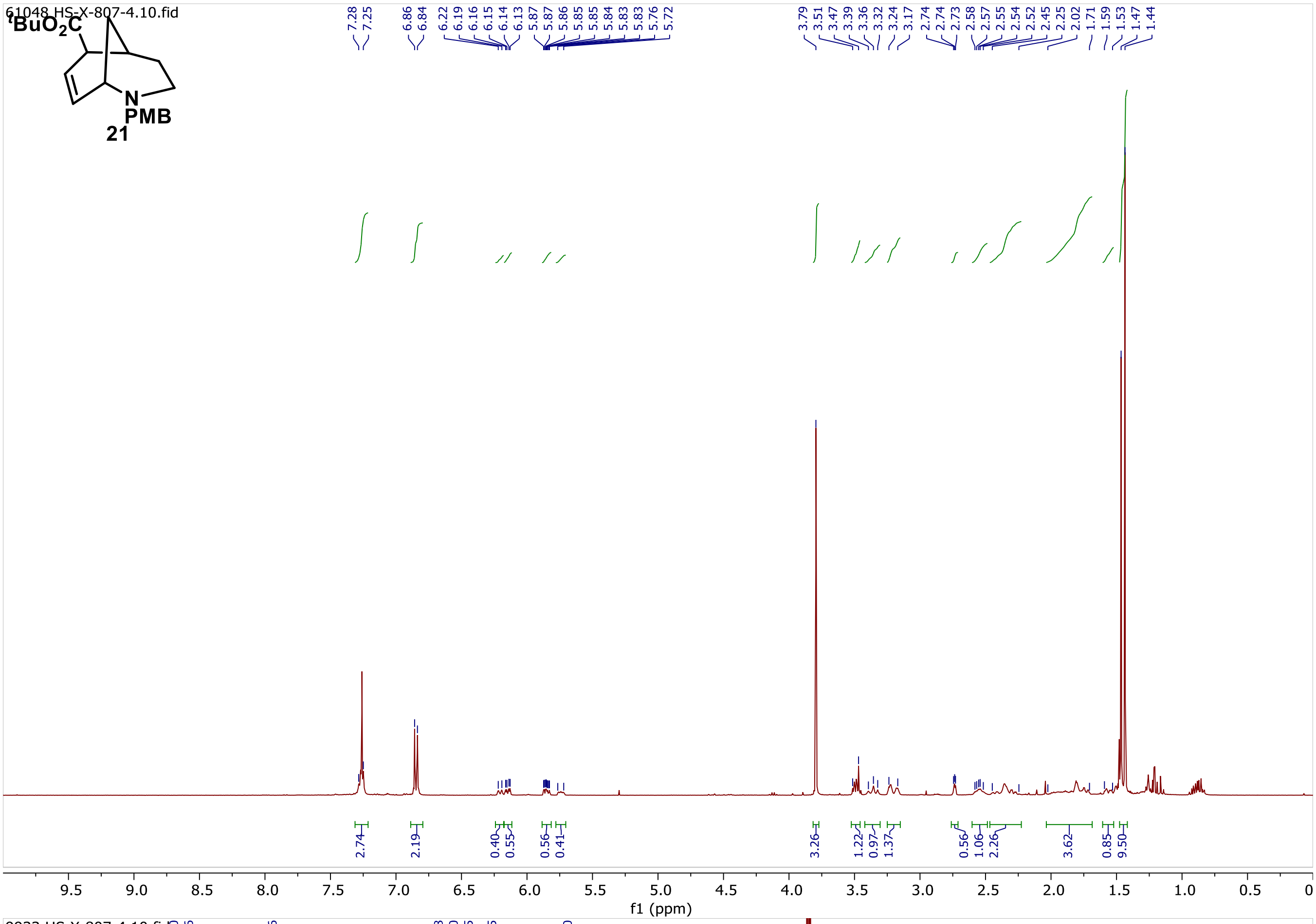

61048 HS-X-807-4.10.fid
^{t}BuO$_2$C
N
PMB
21
f1 (ppm)

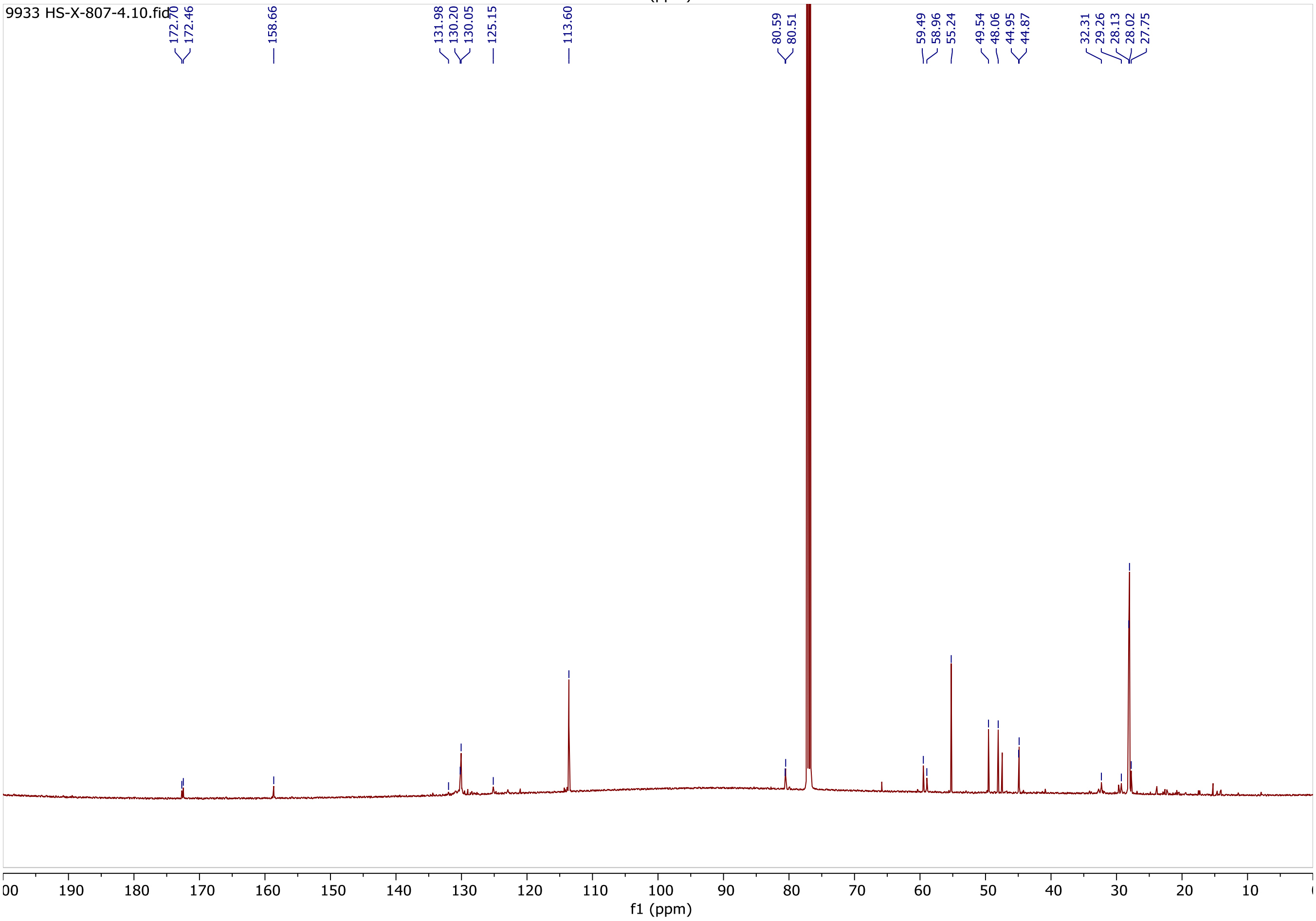

9933 HS-X-807-4.10.fid
f1 (ppm)

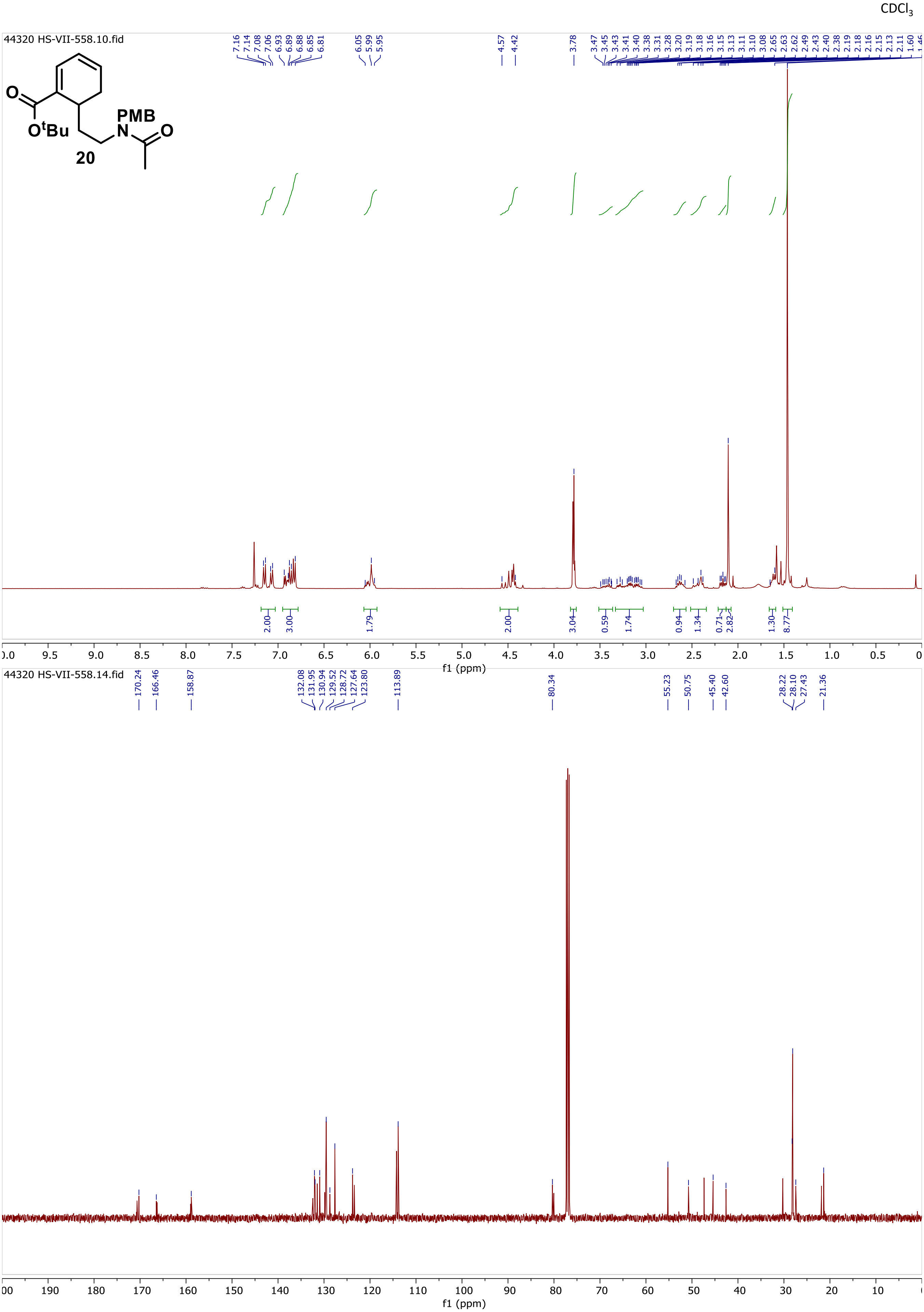
CDCl$_3$
44320 HS-VII-558.10.fid
O
O^{t}Bu
PMB
N
O
20
7.16 7.14 7.08 7.06 6.93 6.89 6.88 6.85 6.81
6.05 5.99 5.95
4.57 4.42
3.78
3.47 3.45 3.43 3.41 3.40 3.38 3.31 3.28 3.20 3.19 3.18 3.16 3.15 3.13 3.11 3.10 3.08 2.65 2.63 2.62 2.49 2.43 2.40 2.38 2.19 2.18 2.16 2.15 2.13 2.11 1.60
2.00 3.00 1.79 2.00 3.04 0.59 1.74 0.94 1.34 0.71 2.82 1.30 8.77
9.5 9.0 8.5 8.0 7.5 7.0 6.5 6.0 5.5 5.0 4.5 4.0 3.5 3.0 2.5 2.0 1.5 1.0 0.5
f1 (ppm)
44320 HS-VII-558.14.fid
170.24 166.46 158.87
132.08 131.95 130.94 129.52 128.72 127.64 123.80 113.89
80.34
55.23 50.75 45.40 42.60
28.22 28.10 27.43 21.36
190 180 170 160 150 140 130 120 110 100 90 80 70 60 50 40 30 20 10
f1 (ppm)

CDCl$_3$

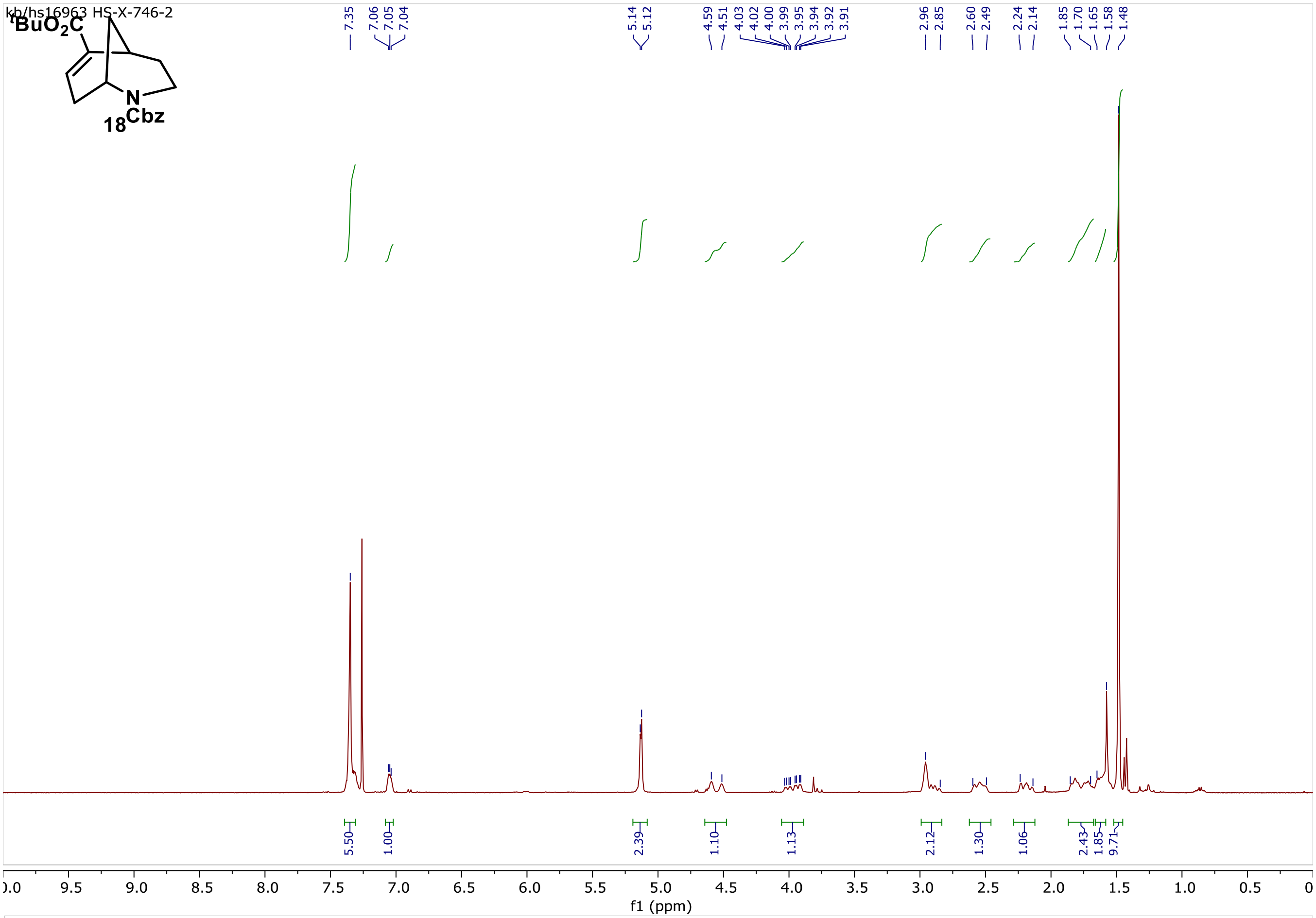

kb/hs16963 HS-X-746-2
tBuO2C
N
18
Cbz
7.35
7.06
7.05
7.04
5.14
5.12
4.59
4.51
4.03
4.02
4.00
3.99
3.95
3.94
3.92
3.91
2.96
2.85
2.60
2.49
2.24
2.14
1.85
1.70
1.65
1.58
1.48
5.50
1.00
2.39
1.10
1.13
2.12
1.30
1.06
2.43
1.85
9.71
f1 (ppm)

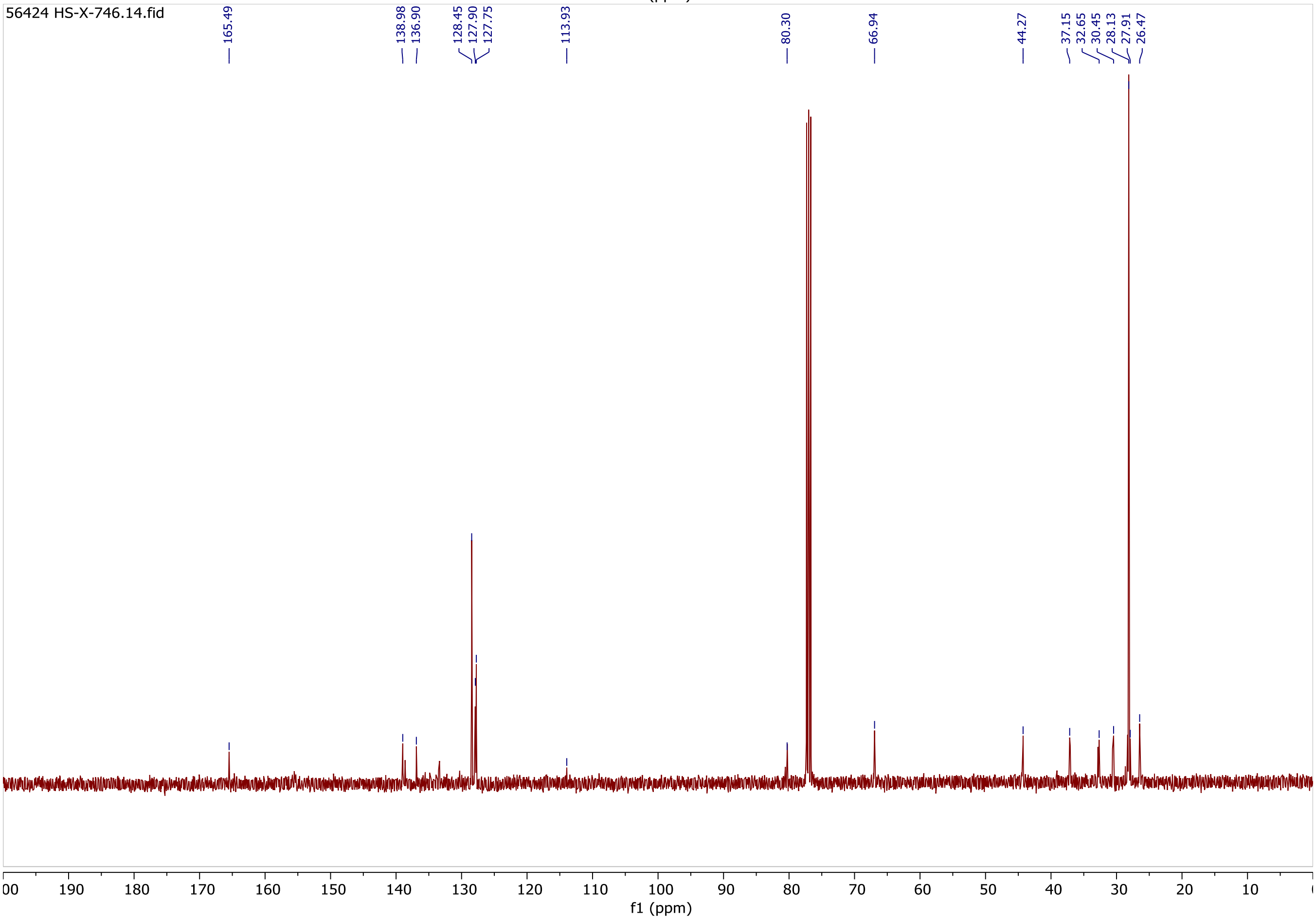

56424 HS-X-746.14.fid
165.49
138.98
136.90
128.45
127.90
127.75
113.93
80.30
66.94
44.27
37.15
32.65
30.45
28.13
27.91
26.47
f1 (ppm)

CDCl$_3$

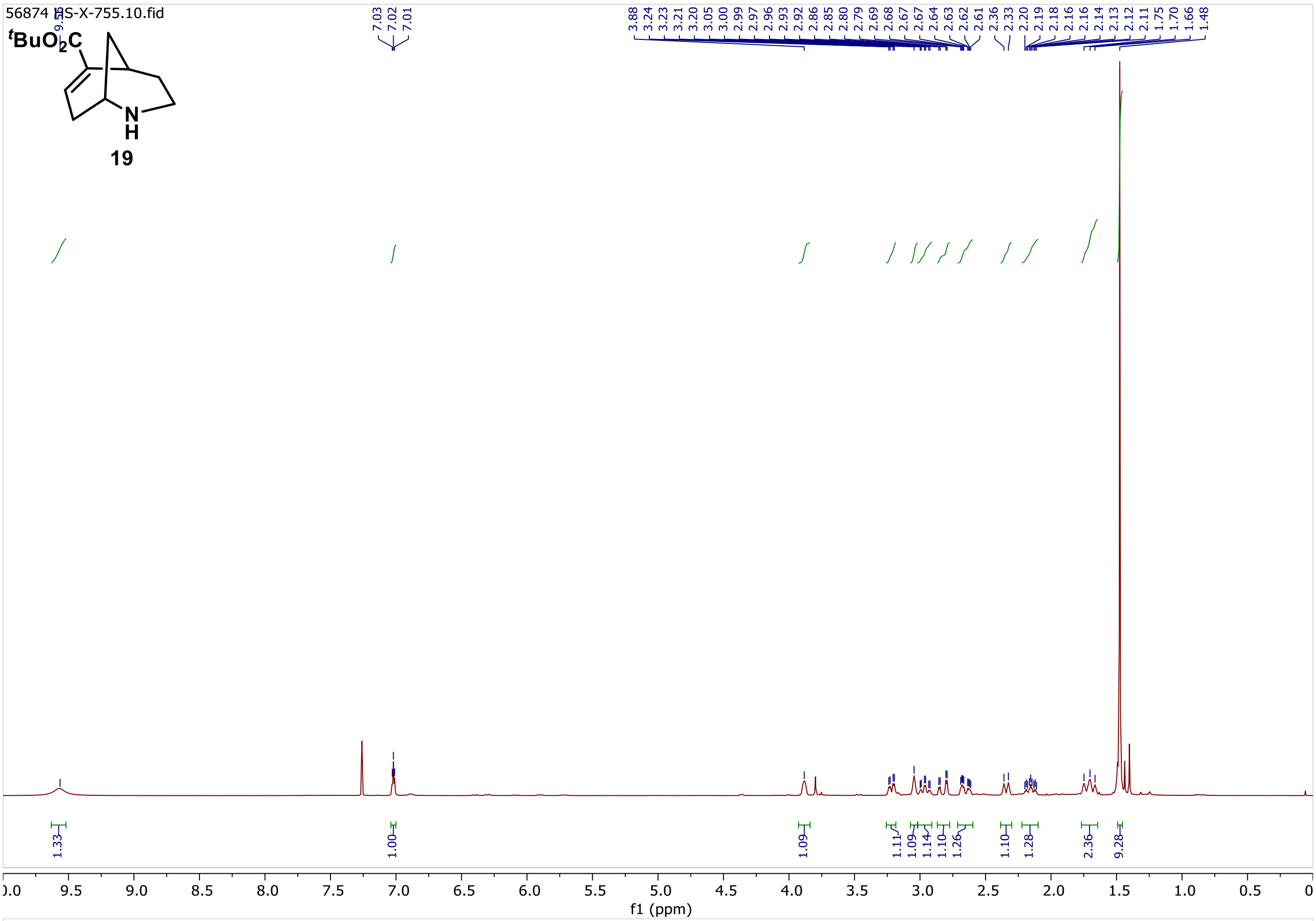

56874 HS-X-755.10.fid
^{t}BuO$_2$C
N
H
19
7.03
7.02
7.01
3.88 3.24 3.23 3.21 3.20 3.05 3.00 2.99 2.97 2.96 2.93 2.92 2.86 2.85 2.80 2.79 2.69 2.68 2.67 2.67 2.64 2.63 2.62 2.61 2.36 2.33 2.20 2.19 2.18 2.16 2.14 2.13 2.12 2.11 1.75 1.70 1.66 1.48
1.33
1.00
1.09
1.11
1.09
1.14
1.10
1.26
1.10
1.28
2.36
9.28
9.5 9.0 8.5 8.0 7.5 7.0 6.5 6.0 5.5 5.0 4.5 4.0 3.5 3.0 2.5 2.0 1.5 1.0 0.5 0
f1 (ppm)

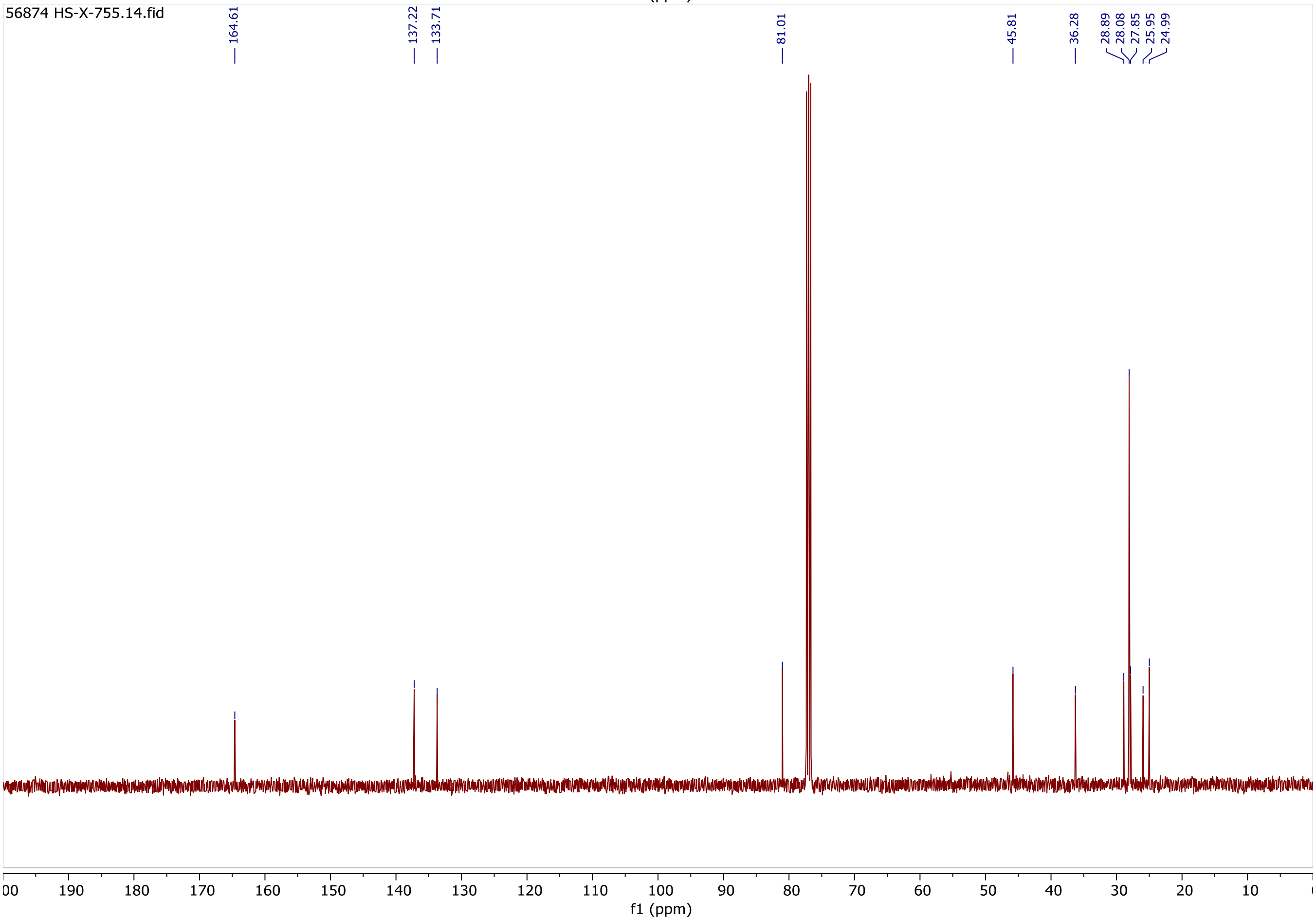

56874 HS-X-755.14.fid
164.61
137.22
133.71
81.01
45.81
36.28
28.89 28.08 27.85 25.95 24.99
190 180 170 160 150 140 130 120 110 100 90 80 70 60 50 40 30 20 10
f1 (ppm)
